# Novel macrocyclic peptidomimetics targeting the insulin-regulated aminopeptidase (IRAP): design, synthesis and evaluation

**DOI:** 10.1039/d5md00438a

**Published:** 2025-08-06

**Authors:** Esther Olaniran Håkansson, Lorenzo J. I. Balestri, Sharathna Puthiyaparambath, Sebastian Moes, Henning Henschel, Christian Sköld, Mathias Hallberg, Mats Larhed, Bobo Skillinghaug, Luke R. Odell

**Affiliations:** a Department of Medicinal Chemistry, BMC Uppsala University P.O. Box 574 SE-751 23 Uppsala Sweden luke.odell@ilk.uu.se; b The Beijer Laboratory, Department of Pharmaceutical Biosciences, Neuropharmacology and Addiction Research, Biomedical Centre, Uppsala University P.O. Box 591 SE-751 24 Uppsala Sweden; c The Beijer Laboratory, Science for Life Laboratory, Department of Medicinal Chemistry, Biomedical Centre, Uppsala University P.O. Box 574 SE-751 23 Uppsala Sweden

## Abstract

Inhibition of the insulin-regulated aminopeptidase (IRAP) is a promising therapeutic strategy for neurodegenerative disorders such as Alzheimer's disease, due to its role in cognitive processes. HA08, a macrocyclic peptidomimetic derived from angiotensin IV, is among the most potent known IRAP inhibitors (IC_50_ = 18 nM). However, detailed structure–activity relationship (SAR) studies at its C-terminus have been limited by synthetic constraints. Herein, we report the design, synthesis, and biological evaluation of a focused series of HA08 analogues to explore the impact of C-terminal modifications on IRAP inhibition. An improved divergent synthetic route was established *via* a common macrocyclic intermediate, enabling late-stage diversification through coupling with non-natural amino acids which led to the synthesis of twelve novel peptidomimetic scaffolds. Several analogues retained high potency, with one-carbon elongation next to the carboxylic acid moiety or secondary amine being well tolerated. In contrast, aliphatic analogues exhibited markedly reduced potency, highlighting the importance of π–π interactions, while the low activity of phenoxyacetic acid derivatives likely reflects altered geometry within the binding pocket. The most potent inhibitor in the series featured a C-terminal benzyl alcohol (IC_50_ = 59 nM), approaching the activity of HA08. To rationalise these SAR trends, molecular dynamics simulations were performed based on the IRAP–HA08 co-crystal structure. Partial least squares analysis of protein–ligand contact patterns revealed that sustained interactions between the C-terminal carboxylate and Arg929 correlated with lower potency, whereas interaction with Arg439 was associated with enhanced activity. These findings suggest that subtle shifts in C-terminal positioning influence binding mode and potency and provides valuable insights for the design of future IRAP inhibitors.

## Abstract 2

A new synthetic platform enables late-stage macrocycle diversification, revealing how subtle C-terminal changes affect potency and guide IRAP inhibitor design.

## Graphical abstract

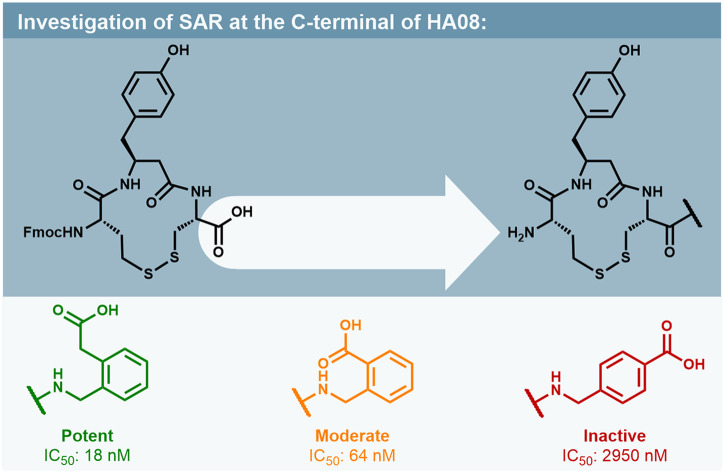

## Introduction

As the global population ages, the prevalence of age-related diseases is on the rise.^1,2^ Among these, Alzheimer's disease (AD) is the most prevalent and devastating, currently ranking as the third leading cause of death among the elderly. Despite extensive research, the underlying mechanisms of AD pathology remains elusive, and no disease-modifying treatments are available.^3^ Approved therapies (cholinesterase inhibitors^4,5^ and NMDA receptor antagonists^6^) offer only symptomatic relief,^7^ underscoring the urgent need for novel therapeutic approaches.^7^ Clinically, AD is marked by progressive loss of memory and cognitive decline.^3^ Therefore, the discovery of compounds that enhance memory and learning in animal models of AD has sparked considerable interest, both for their therapeutic potential and for what they may reveal about the mechanisms underlying cognitive decline.

**Fig. 1:**
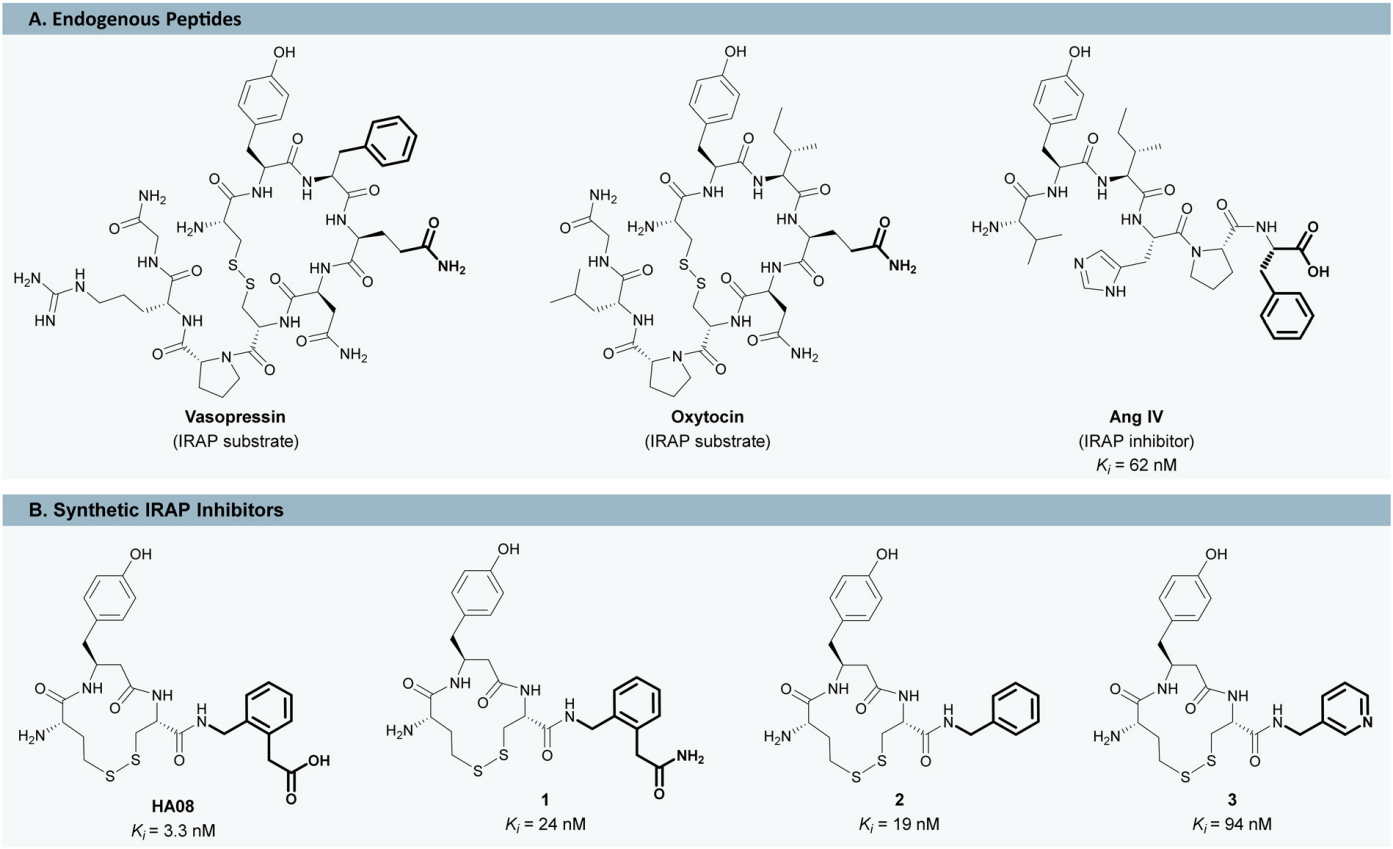
Endogenous peptides (A) and synthetic macrocyclic inhibitors (B) interacting with the insulin-regulated aminopeptidase (IRAP).

One such compound is angiotensin IV (Ang IV, Val–Tyr–Ile–His–Pro–Phe, Fig. 1), a hexapeptide fragment of the renin–angiotensin system that improves performance in rodent models of cognitive impairment as was first demonstrated by Braszko *et al.* in 1988.^8–11^Ang IV was initially thought to act *via* a distinct AT_4_ receptor but subsequent research identified this target as a zinc-dependent aminopeptidase with broad physiological roles. This enzyme is now most commonly referred to as the insulin-regulated aminopeptidase (IRAP, EC 3.4.11.3), although it is also known as oxytocinase and placental leucine aminopeptidase in reproductive and endocrine contexts.^12,13^ In healthy rat brains, IRAP is highly expressed in regions central to memory and learning such as the hippocampus and the neocortex.^8,14,15^ Since IRAP inhibition has been linked to cognitive enhancement, it is a promising molecular target in the context of AD.^16,17^

Ang IV is an inhibitor with moderate affinity but is rapidly degraded, limiting its therapeutic potential.^18^ This limitation has spurred the development of peptidomimetic IRAP inhibitors with higher metabolic stability,^12,19,20^ among which the macrocyclic compound HA08 (IC_50_ = 18 nM, determined in this study; *K*_i_ = 3.3 nM (ref. 21)) has emerged as a lead scaffold. HA08 contains a C-terminal γ-turn mimetic (2-(aminomethyl)phenylacetic acid, AMPA) and a disulfide-linked macrocyclic core, structural features shared with the endogenous IRAP substrates vasopressin and oxytocin (Fig. 1).^21,22^

Although the precise mechanism by which IRAP inhibition improves cognitive function remains to be elucidated, several hypotheses have been proposed.^10,11,23^ One hypothesis is that IRAP inhibitors increase the half-life of memory-promoting neuropeptides (such as vasopressin and oxytocin) by preventing their IRAP-mediated degradation.^24^ Another is that IRAP impacts neuronal glucose uptake *via* the GLUT4 transporter, indirectly promoting dendritic spine density, a marker of synaptic plasticity.^25^ Indeed, administration of HA08 has been shown to increase dendritic spine density in the hippocampus,^26^ suggesting a possible link to the synaptic degeneration observed in AD and other neurodegenerative conditions.^27,28^ Despite its potency, HA08 is susceptible to proteolytic degradation *in vivo*, making it best suited as a chemical biology probe in dementia models rather than a therapeutic lead.^29,30^

Small molecule inhibitors of IRAP have been pursued since 2008, when the first series of benzopyran-based inhibitors were identified by virtual screening of a homology model of the catalytic domain.^17,31^ Subsequent efforts, including conventional screening^32^ and our own high-throughput screening protocols,^33^ have yielded several different types of inhibitors including aryl sulfonamides,^34,35^ quinazolones,^36^ imidazo[1,5-*α*]pyridines^37^ and hydroxamic acids,^38^ all displaying varying potencies. Nevertheless, HA08 remains the most potent inhibitor reported to date. This motivated us to further examine the scaffold of HA08 in more detail.

To advance the pharmacological potential of the HA08 scaffold, novel analogues with improved properties are needed. While extensive structure–activity relationship (SAR) studies have been conducted around the macrocyclic core and N-terminal region of HA08,^21,39^ the C-terminal region remains largely unexplored. This is likely due to the constraints of the traditional C-terminal to N-terminal linear solid-phase peptide synthesis (SPPS) approach, which limits diversification at the C-terminus.^39^ For example, the IRAP inhibitors 1–3 (Fig. 1) can currently only be accessed by varying the resin and/or the first amino acid in the sequence.^21,39^

Recently, the crystal structure of IRAP in complex with HA08 (PDB ID: 6YDX) has shed new light on the flexibility and plasticity of the binding site.^40^ From the crystal structure two distinct binding modes were identified: one involving electrostatic interactions between the C-terminal carboxylate and Arg929/Arg439 (Fig. S6A), and another featuring π–π stacking between the phenyl ring and Tyr961 (Fig. S6B). These findings suggest that C-terminal modifications may significantly influence binding pose and affinity, and that novel analogues with altered geometry and electronics could be accommodated by the IRAP active site. Moreover, the minimal loss of activity observed with C-terminal modifications in compounds 1–3 implies that the C-terminal region can tolerate structural variation.^21,39^

Herein, we report the design, synthesis, and biological evaluation of a series of C-terminally modified HA08 analogues. To overcome previous synthetic limitations, we developed a divergent strategy centred around a macrocyclic intermediate, allowing for late-stage diversification at the C-terminal position. Furthermore, novel rigid and elongated aromatic amino acid motifs were designed and incorporated. This approach enabled a focused SAR investigation in this region, using biochemical assays and molecular dynamics (MD) simulations to explore the impact of specific C-terminal modifications on IRAP binding and inhibition.

## Results and discussion

### Chemistry

The reported synthesis of HA08 employs an azide-functionalized AMPA moiety as a masked amine to prevent intramolecular cyclization to the *δ*-lactam 1,4-dihydroisoquinolin-3(2*H*)-one.^19,21,41^ This strategy relies on a tandem Staudinger reduction and aza-Wittig amide formation, wherein the resin-bound azide is reduced *in situ* and coupled to a carboxylic acid in the presence of a phosphine. However, we encountered issues during our attempts to reproduce this synthesis due to incomplete amide bond formation during the Staudinger reaction. Attempts to circumvent this issue by performing the reduction of the azide in solution were unsuccessful (Table S1). Under all investigated conditions the *δ*-lactam was formed as either the sole product or the major component of a complex mixture. This side reaction likely accounts for the low reproducibility observed during our attempts to synthesize HA08*via* SPPS.

**Scheme 1:**
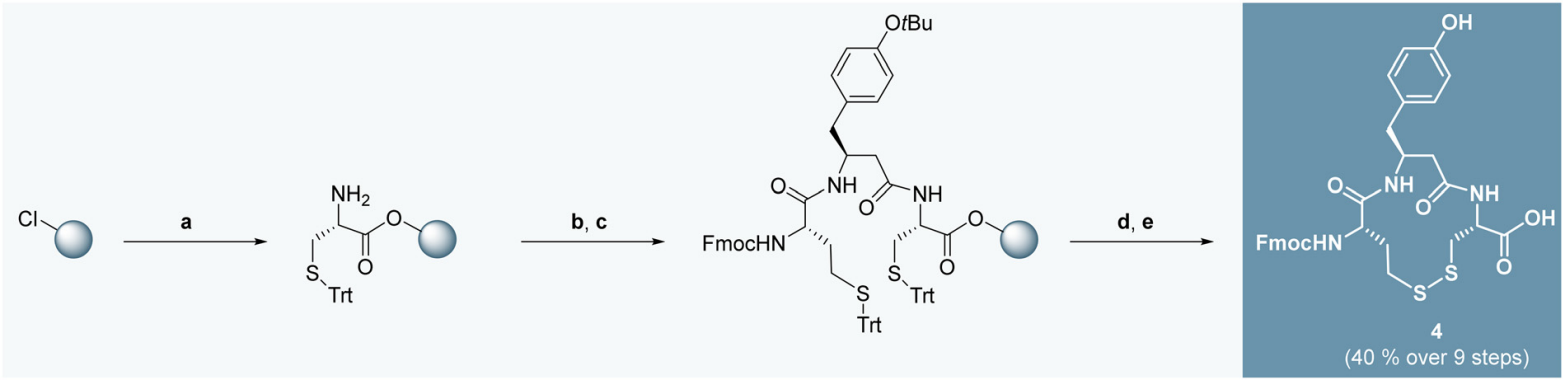
Synthesis of Fmoc-protected key intermediate 4 by SPPS using 2-chlorotrityl chloride resin (2-CTC resin). Reagents and conditions: a) i. 2-CTC resin (1 equiv.), SOCl_2_ (1.2 equiv.), pyridine (2.4 equiv.), DCM, rt, 6 h; ii. Fmoc-Cys(Trt)-OH (2 equiv.), DIPEA (7 equiv.), DCM, rt, ON; iii. DCM/MeOH/DIPEA (86 : 10 : 4), rt, 1 h; iv. 20% piperidine in DMF, rt, (3 × 10 min); b) i. Fmoc-βhTyr(*t*Bu)-OH (2 equiv.), oxyma (4 equiv.), DIC (4 equiv.), DIPEA (7 equiv.), DMF, rt, ON; ii. 20% piperidine in DMF, rt, (3 × 10 min); c) Fmoc-hCys(Trt)-OH (2 equiv.), oxyma (4 equiv.), DIC (4 equiv.), DIPEA (7 equiv.), DMF, rt, 4 h; d) TFA/H_2_O/TIS/1,2-ethanedithiol (92.5 : 2.5 : 2.5 : 2.5), rt, 2 h; e) TFA : acetonitrile (1 : 1), rt, 3 d.

To address this issue, we devised a novel divergent synthetic strategy that enables the efficient synthesis of peptidomimetic IRAP inhibitors. This approach leverages a key macrocyclic intermediate (compound 4, Scheme 1) that can be diversified in the final step by coupling with a series of non-natural amino acids. This strategy not only avoids *δ*-lactam formation and epimerization,^21^ but also provides late-stage flexibility for C-terminal modification, thereby facilitating rapid SAR exploration.

Compound 4 was synthesized by standard Fmoc-based SPPS on 2-chlorotrityl chloride (2-CTC) resin (Scheme 1). Activation of the resin was performed using thionyl chloride, pyridine and DCM, followed by sequential coupling of Fmoc-Cys(Trt)-OH, Fmoc-βhTyr(*t*Bu)-OH and Fmoc-hCys(Trt)-OH using Oxyma Pure and DIC as coupling reagents. The *N*-Fmoc-protected tripeptide was cleaved from the resin and cyclized in TFA/acetonitrile, affording 4 in 40% overall yield with negligible epimerization and minimal side-product formation, representing a notable improvement over the previous linear route.^21^

**Scheme 2:**
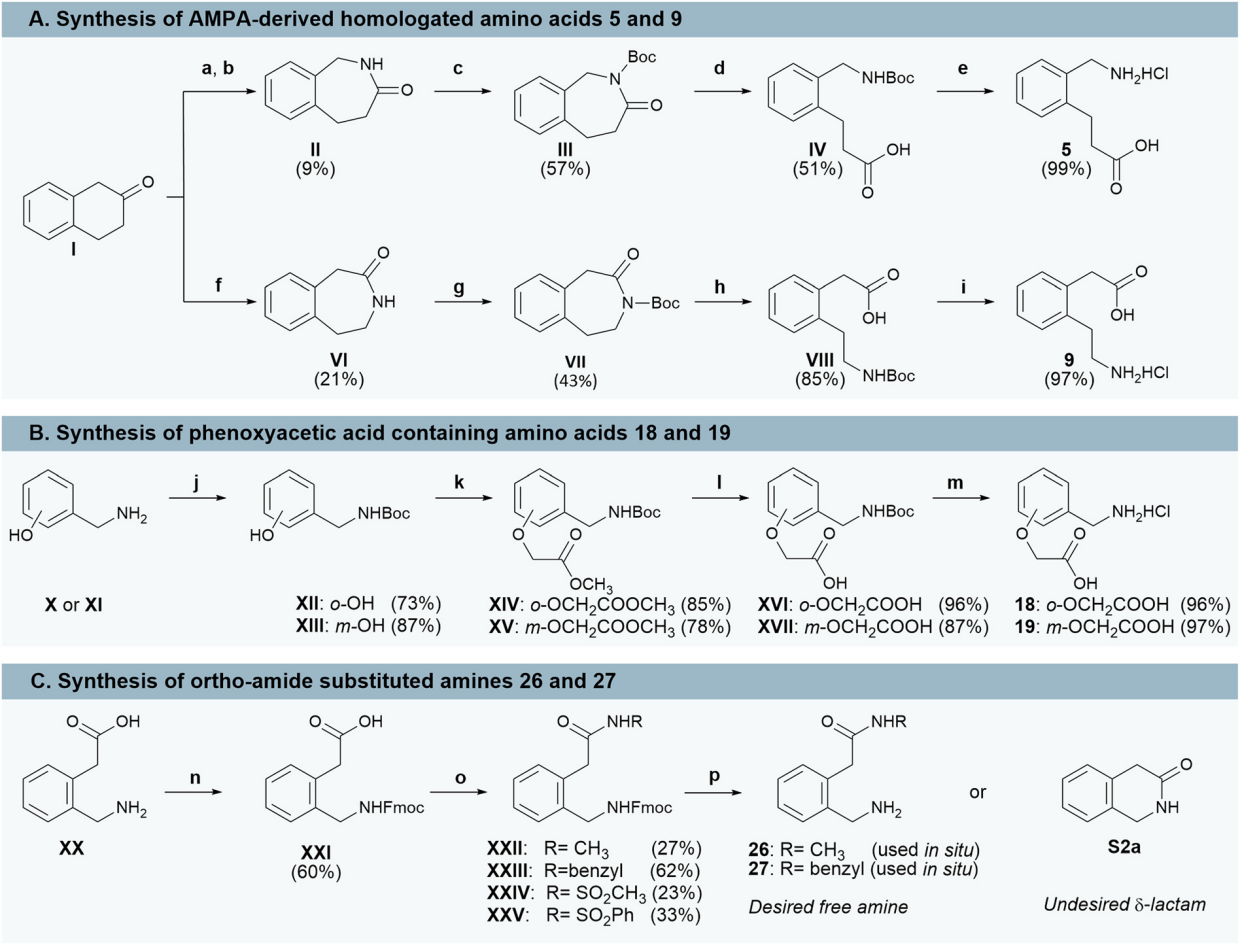
Synthesis of non-natural amino acid building blocks. Reagents and conditions: a) NH_3_OH·HCl (1.22 equiv.), H_2_O : EtOH (2 : 3), NaOAc (1.62 equiv.), rt, 2 h; b) SOCl_2_ (6.6 equiv., DCM, rt, ON; c) Boc_2_O (2.00 equiv.), DCM, Et_3_N (1.00 equiv.), DMAP (1.00 equiv.) rt, ON; d) 1 N LiOH (6 equiv.), THF, 50 °C, ON; e) 4 M HCl in dioxane, rt, ON; f) MsOH (1.62 equiv.), NaN_3_ ((1.30 equiv.), 0 °C to rt, ON; g) Boc_2_O (2.00 equiv.), DCM, Et_3_N (1.00 equiv.), DMAP (1.00 equiv.), rt, ON; h) 1 N LiOH (6 equiv.), THF, 50 °C, ON; i) 4 M HCl in dioxane, rt, ON; j) Boc_2_O (1.00 equiv.), H_2_O : THF (1 : 2), rt, ON; k) anhydrous Cs_2_CO_3_ (1.20 equiv.) in acetone, methyl bromoacetate (1.2 equiv.), rt, 2.5 h; l) K_2_CO_3_ in acetonitrile : H_2_O (2 : 1), MW, 100 °C, 20 min; m) 4 M HCl in dioxane, rt, ON; n) Fmoc-Cl (1.20 equiv.), 1,4-dioxane, 10 wt% Na_2_CO_3_, rt, 4 h; o) EDCI·HCl (2.15 equiv.), DMAP (2.15 equiv.), DCM, rt, 2–12 h, p) 20% piperidine in DMF, rt, 30 min.

To enable C-terminal diversification, a variety of novel non-natural amino acids (compounds 5, 9, 18–19, 26–27) were synthesized (Scheme 2). The homologated analogues 5 and 9 were prepared from β-tetralone (Scheme 2A). Treatment of I (Roman numerals denote intermediates) with sodium azide in concentrated methansulfonic acid produced regioisomers II and VI in approximately a 1 : 1 ratio (as determined by ^1^H-NMR spectroscopic analysis). Regioisomer VI readily precipitated during workup, affording a 21% yield upon trituration with isohexane/DCM. Regioisomer II could be selectively generated by conversion of I to the corresponding ketoxime (1 : 1 mixture of *E* and *Z* isomers) followed by thionyl chloride-catalysed Beckmann rearrangement, albeit in low yield. Lactams III and VII were obtained *via* Boc-protection of II and VI, respectively, and subsequent base-mediated hydrolysis gave intermediates IV and VIII. Final deprotection with 4 M HCl in dioxane yielded the desired novel non-natural amino acids as the HCl salts 5 and 9.

Oxygen-atom extended analogues 18 and 19 were synthesized in four steps from commercially available 2-(aminomethyl)phenol (X) and 3-(aminomethyl)phenol (XI), respectively (Scheme 2B). Boc-protection afforded carbamates XII and XIII, which were alkylated with methyl bromoacetate to give ethers XIV and XV. Hydrolysis under microwave-assisted heating furnished the carboxylic acids XVI and XVII, which were finally deprotected to yield the target amino acids 18 and 19 as their corresponding HCl salts.

Amide (XXII–XXIII) and acyl sulfonamide (XXIV–XXV) intermediates were prepared according to Scheme 2C. To minimize lactam formation, the amino group of 2-(aminomethyl)phenylacetic acid (XX) was first protected as the Fmoc-derivative XXI. After optimization (see Table S2 in SI), XXI was coupled with the appropriate nucleophile using EDCI·HCI and DMAP in DCM to yield 23–62% of XXII–XXV. Treatment of intermediates XXII and XXIII with piperidine proceeded to give the free amines 26–27 which were used without purification. However, Fmoc deprotection of acylsulfonamides XXIV and XXV proved challenging as they readily cyclized to the corresponding *δ*-lactam S2a under mildly basic conditions.

The pronounced cyclization of acyl sulfonamides is likely due to the enhanced leaving group ability of the sulfonamide (p*K*_a_ ≈ 10) compared to the corresponding amide (p*K*_a_ ≈ 35). Due to the instability of the acyl sulfonamide intermediates under the deprotection conditions, their incorporation into final analogues was not pursued in this study. Nevertheless, the divergent synthetic strategy developed herein supports late-stage C-terminal diversification and may enable access to such derivatives using alternative deprotection or protecting groups.

**Scheme 3:**
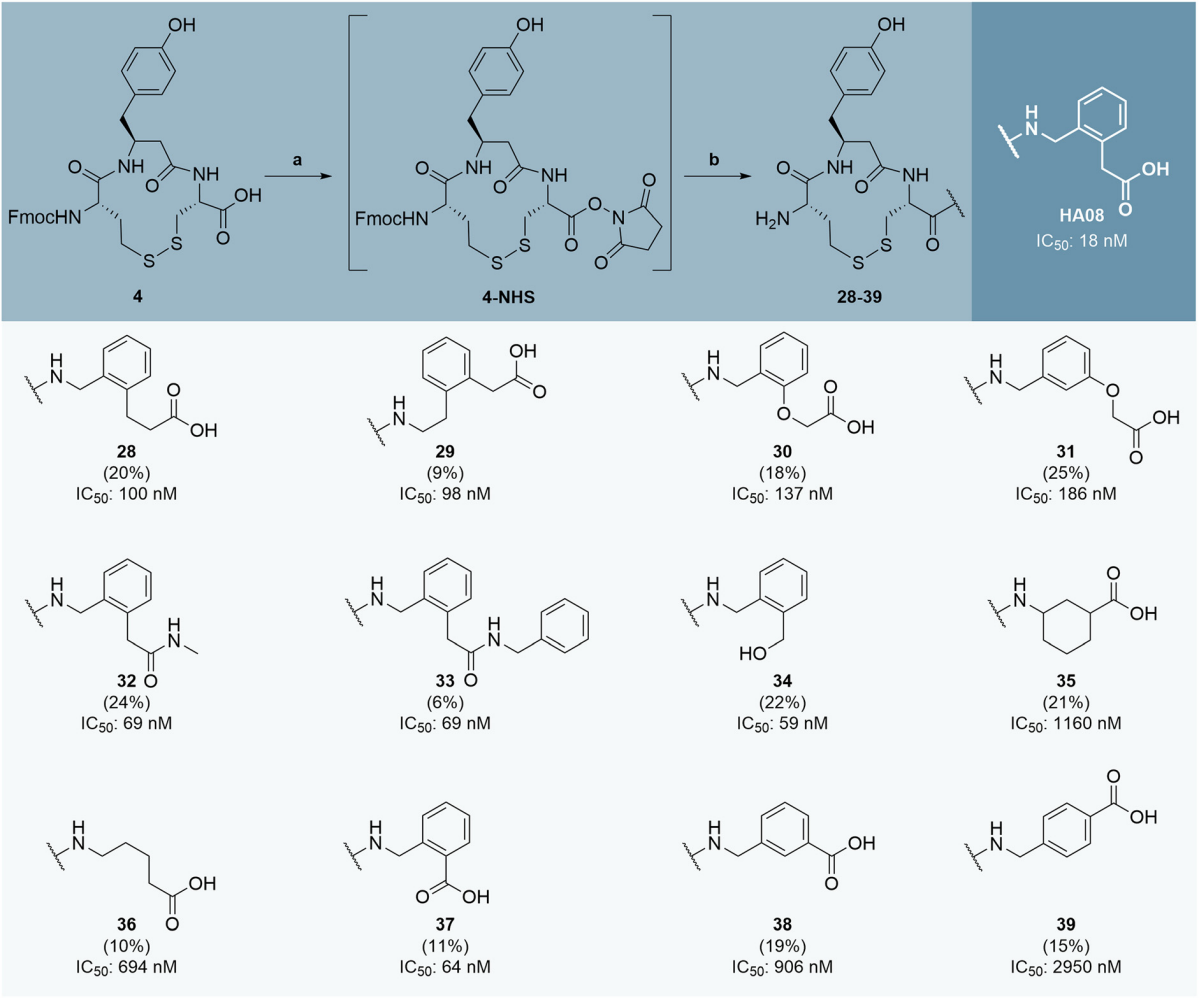
Synthesis and *in vitro* evaluation of novel HA08 analogues with various non-natural amino acids at the C-terminal. Reagents and conditions: a) *N*-hydroxysuccinimide (1.2 equiv.), DCC (1.2 equiv.), DMF, rt, 4 h–ON, b) i. amino acid (1.5–3.5 equiv.), rt, 2 h–ON, ii. piperidine (3 equiv.), rt, 30 min.

The final peptidomimetic IRAP inhibitors 28–39 were prepared as outlined in Scheme 3, which also includes the inhibitory potency of the analogues and HA08 against IRAP. Key intermediate 4 was activated with *N*-hydroxysuccinimide (NHS) and DCC in DMF to give NHS-ester 4-NHS, which was subsequently coupled with synthesized (5, 9, 18–19, 26–27) and commercially available non-natural amino acids to afford the target compounds. Crude products were precipitated in cold diethyl ether and purified by HPLC to yield the final target peptides 28–39 as TFA salts in 6–25% isolated yield.

### *In vitro* evaluation

IRAP inhibition was quantified using a fluorescence-based enzymatic assay, measuring the hydrolysis of l-leucine-7-amido-4-methylcoumarin, as previously described by Gising *et al.*^33^ Dose-dependent inhibition of IRAP by synthesized peptidomimetics 28–39 and HA08 as a positive control was assessed in 384-well plates using excitation and emission wavelengths of 355 nm and 460 nm, respectively.

Compounds 28 and 29 (Scheme 3), featuring a one carbon elongation adjacent to the C-terminal carboxylic acid or the secondary amine, respectively, exhibited similar activity and were approximately six times less potent than HA08. This modest decline suggests that minor lengthening of the terminal side chain is tolerated but suboptimal for binding.

In contrast, phenoxyacetic acid derivatives 30 and 31 demonstrated a more pronounced loss of activity, with IC_50_ values 8–10 times higher than HA08. These results imply that although the aromatic character is preserved, the additional ether oxygen or altered geometry may disrupt key interactions within the binding pocket.

The secondary amides 32 and 33 retained significant inhibitory activity, with only 4-fold reduction in potency (compared to HA08). The relative potency of these substituted amides is only slightly reduced compared to 1,^39^ indicating that the charged carboxylic acid is not strictly required for IRAP inhibition and that a neutral amide can serve as a viable C-terminal replacement. Notably, no clear trend was observed regarding the size of the N-substituent, suggesting limited steric sensitivity at this position. Analogue 34, featuring a C-terminal benzyl alcohol, was the most potent compound in this series (IC_50_ = 59 nM), suggesting that a hydroxyl group can effectively mimic the carboxylate through hydrogen bonding or polar interactions.

Truncation of the C-terminal side chain was better tolerated than extension. However, both aliphatic analogues 35 and 36 exhibited weak inhibition, with IC_50_ values in the micromolar range. These findings underscore the critical role of aromaticity in the C-terminal region, likely contributing through π-stacking or other non-covalent interactions essential for high-affinity binding. Notably, the type of aromaticity also matters as it has been previously reported that substitution of phenyl in compound 2 with a pyridyl (compound 3) resulted in a 5-fold decrease in potency.^21^ Analogue 37, which features a benzoic acid instead of the phenylacetic acid of HA08, maintained high potency (IC_50_ = 64 nM), indicating that the methylene linker is not essential for IRAP inhibition and *ortho* substitution can increase potency (*cf.* compound 2). Interestingly, relocation of the carboxylic acid to the *meta* or *para* position, as in compounds 38 and 39 led to a dramatic loss of activity, with IC_50_ values 14-fold and 46-fold higher than compound 37, respectively. This illustrates the importance of correct spatial orientation of the C-terminal carboxylate for potent IRAP inhibitors.

### Computational investigation

The positional isomers 37–39 in which the C-terminal carboxylate is positioned at the *ortho*-, *meta*-, and *para* position, respectively, varied greatly in their potency (IC_50_ = 64 nM, 906 nM, and 2950 nM). Compared to HA08 with a methylene linker, these analogues have more rigid conformational properties as well as altered position of the carboxylic acid, which could potentially interact with different positively charged residue side-chains. To better understand the striking differences in potency, we performed MD simulations to probe their binding modes using HA08 as a reference. Initial ligand poses were derived from the co-crystallized HA08–IRAP complex (PDB ID: 6YDX), and all simulations were conducted in triplicate for 500 ns, corresponding to 1.5 μs of sampling for each ligand. Independent starting conformations for the replicates were extracted from the initial trajectory of each ligand to ensure diverse sampling.

**Fig. 2:**
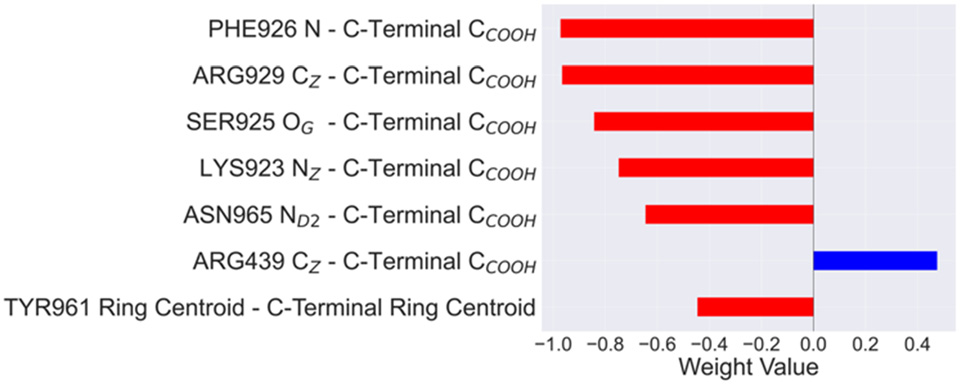
Contact weights to the ligand C-terminal from PLS analysis as a bar graph with relative importance of specific contacts.

Throughout the simulations, the N-terminal interactions of HA08 and analogues 37–39 remained largely conserved and were consistent with the crystal structure.^40^ These were characterized by stable coordination of the ligand's amide oxygen to the catalytic Zn^2+^ and salt bridges between the terminal amine and glutamic acid residues in the active site. To investigate the role of C-terminal interactions in modulating potency, we performed partial least squares (PLS) regression to analyze the frequency of protein–ligand contacts during the simulations. The bar graph in Fig. 2 illustrates the relative importance (weights) of specific contacts between the C-terminal of the ligand and protein residues as determined by the PLS regression model. A graph of model weights with all contacts including those not shown here can be found in Fig. S8 and coefficients of the model can be found in Table S7.

The analysis identified two residues, Arg439 and Arg929, which correlate to binding affinity. These two residues were previously observed to interact with the C-terminal carboxylate of HA08 in the crystal structure. However, the PLS model revealed a negative correlation between binding affinity and the occupancy of the contact between the ligands' C-terminal carboxylate and Arg929, despite this interaction being present in the HA08 crystal structure.

**Fig. 3:**
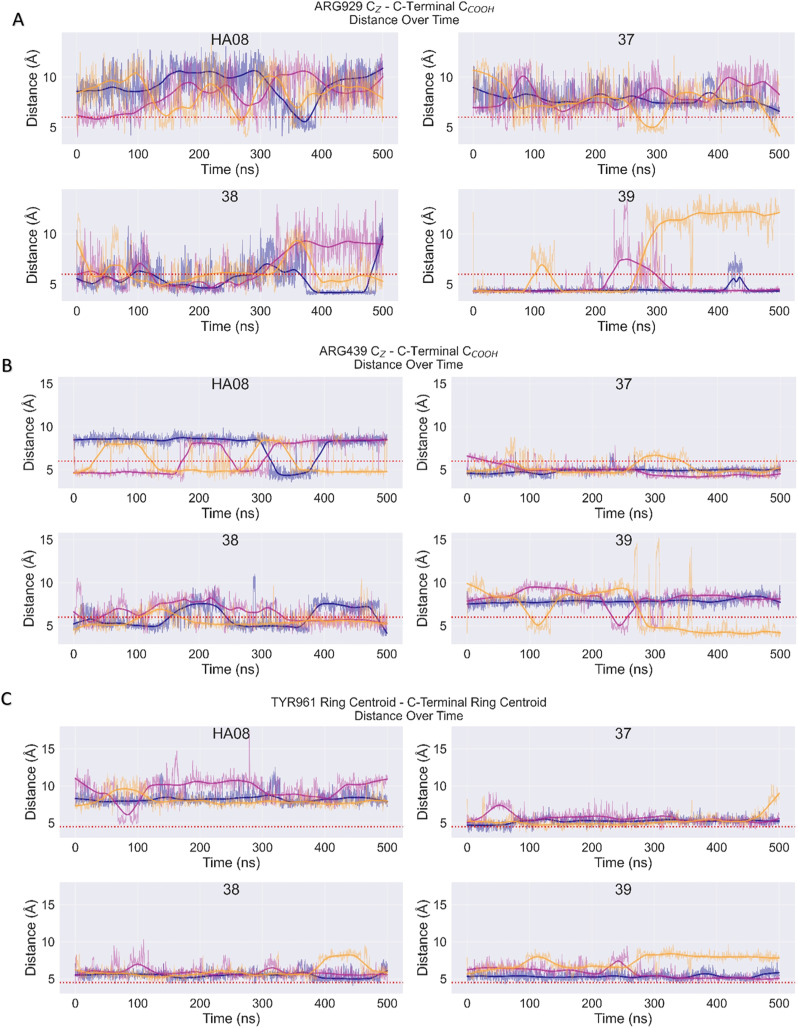
Time evolution of distances between C-terminal carboxylate (carbon) of the inhibitor and the guanidinium group (carbon) of A) Arg929 and B) Arg439 as well as C) between the aromatic rings of inhibitor in the C-terminal and Tyr961. Solid lines represent smoothed trends, shaded regions show fluctuations, and the colours represent each separate simulation. The red dashed line shows threshold distance for contact occupancy.

As visualized in Fig. 3A, the lower affinity compounds 38 and 39 exhibited persistent interactions with Arg929 across all simulation replicates. In contrast, the more potent HA08 and 37 rarely formed this contact. Instead, both HA08 and 37 preferentially engaged Arg439 with their C-terminal carboxylate as shown in Fig. 3B. In the case of 37, this interaction is maintained almost continuously throughout all simulations. HA08, while also forming Arg439 contacts in all replicates, exhibited dynamic switching between two binding modes involving this residue.

However, the π–π interaction between Tyr961 and the HA08 C-terminal, which is present in one of the crystal structure conformations, was not observed as a stable interaction in the MD simulations. For HA08, these rings were only close to each other for a short period in one of the simulation replicates, as shown by the plots of the ring centroid distances in Fig. 3C. On the other hand, the C-terminal phenyl groups of 37–39 were close to Tyr961 during most of the simulated time, indicating that this interaction may be important for these compounds. Nevertheless, this modelling investigation does not allow for a clear conclusion about how differences in the C-terminal affect inhibitor potency. Both the compounds with high inhibitory effect and the compounds with low inhibitory effects obtain well-matched interactions with their carboxylic acid moiety to arginine residues, albeit different ones. Although the specific energetic consequences of these interactions are difficult to quantify from this dataset alone, the correlation between the frequency of contact with Arg929 and decreased potency suggests that this residue may contribute to a less favourable binding mode. It is possible that the *para*- and *meta*-substituted analogues may orient the carboxylate in a geometry that promotes interaction with Arg929, potentially compromising more optimal contacts elsewhere in the binding pocket. Additionally, subtle differences in C-terminal length and angle may influence ligand positioning and perturb favourable contacts in other regions of the molecule.

## Conclusions

Preclinical studies have demonstrated the potential of IRAP inhibitors as cognitive enhancers and for treating neurodegenerative conditions such as Alzheimer's disease, which are characterized by progressive cognitive and memory decline. Among these, HA08, a macrocyclic peptidomimetic derived from angiotensin IV, has emerged as one of the most potent IRAP inhibitors reported to date. However, its susceptibility to proteolytic degradation has limited its utility as a therapeutic compound.

In this study, we developed a divergent and modular synthetic strategy that enables efficient late-stage diversification of HA08 analogues, with particular focus on the C-terminal region. This approach addressed key synthetic challenges, including intramolecular cyclization, and facilitated the preparation of a structurally diverse set of macrocyclic peptidomimetics. As part of this strategy, we also synthesized and characterized various non-natural amino acids that were incorporated into the C-terminal of HA08. These building blocks are not only valuable for this series but hold broader application in the synthesis of other bioactive compounds.

*In vitro* evaluation of these compounds identified several potent IRAP inhibitors and elucidated key structure–activity relationships at the C-terminal moiety. Molecular dynamics (MD) simulations supported these findings by providing mechanistic insights into how subtle geometric variations influence critical protein–ligand interactions. Collectively, these results expand the chemical space of IRAP inhibitors and highlight the importance of C-terminal geometry in modulating biological activity. The most promising analogues identified in this study warrant further mechanistic and *in vivo* evaluation to assess their therapeutic potential for enhancing cognitive function in neurodegenerative diseases.

## Experimental

### General information

Analytical thin-layer chromatography (TLC) was performed on silica gel 60 F-254 plates and visualized with UV light. Flash column chromatography was performed using silica gel 60 (40–63 μm). ^1^H NMR spectra were recorded at 400 or 500 MHz. ^13^C{^1^H} NMR spectra were recorded at 101 or 126 MHz. Chemical shifts (*δ*_H_) are quoted in parts per million (ppm). Analytical HPLC/ESI-MS was performed using electrospray ionization (ESI) and a Penomenex Kinetex C18 column (50 × 3.0 mm, 2.6 μm particle size, 100 Å pore size) with CH_3_CN/H_2_O in 0.05% aqueous HCOOH as mobile phase at a flow rate of 1.5 ml min^−1^. Preparative reversed-phase high-performance liquid chromatography (RP-HPLC) was performed on a Glison HPLC system with UV-triggered (214 nm) fraction collection using a Machery-Nagel NUCLEODUR C18 HTec column (21 × 125 mm, particle size 5 μm) with a gradient of H_2_O/CH_3_CN/0.1% TFA as mobile phase at a flow rate of 25 mL min^−1^. High-resolution molecular masses (HRMS) were determined on a mass spectrometer equipped with an ESI source and a time-of-flight (TOF) mass analyzer. All chemicals and solvents were purchased from Sigma Aldrich, Fisher Scientific, or VWR, and used without further purification.

### General procedure A: synthesis of peptidomimetics 28–39

The Fmoc-protected macrocyclic tripeptide intermediate 4 (1.0 equiv.) was dissolved in anhydrous DMF (1 mL) and treated with *N*-hydroxysuccinimide (NHS, 1.2 equiv.) and dicyclohexylcarbodiimide (DCC, 1.2 equiv.) in a sealed microwave vial. The reaction mixture was stirred at room temperature for 4 h, after which formation of the desired NHS ester 4-NHS was confirmed by LC-MS. The cap was removed and the appropriate non-natural amino acid (1.5–3.5 equiv.) was added. The reaction mixture was stirred at room temperature until complete conversion as determined by LC-MS (2 h to overnight). The Fmoc group was then removed by addition of piperidine (3 equiv.) and stirring at room temperature for 30 minutes. The crude product was precipitated in cold diethyl ether, purified by RP-HPLC and lyophilized to yield final IRAP inhibitors as their corresponding trifluoroacetate (TFA) salts.

### Synthesis and characterization of peptidomimetics

#### Compound 28

Peptidomimetic 28 was synthesized according to general procedure A using 3-(2-(aminomethyl)phenyl)propanoic acid hydrochloride (3.5 equiv.) in the second step (11 mg, 20%). HRMS (ESI/TOF) *m*/*z*: [M + H]^+^ calcd for C_27_H_35_N_4_O_6_S_2_ 575.1998; found 575.1982. ^1^H NMR (400 MHz, DMSO-*d*_6_) *δ* 12.15 (s, 1H), 9.22 (d, *J* = 5.0 Hz, 1H), 8.55 (d, *J* = 7.4 Hz, 1H), 8.43 (m, 2H), 8.12 (s, 3H), 7.16 (m, 4H), 7.07 (m, 2H), 6.68 (m, 2H), 4.42 (m, 1H), 4.28 (dd, *J* = 5.8, 2.4 Hz, 2H), 4.06 (m, 1H), 3.76 (s, 1H), 2.99 (d, *J* = 7.3 Hz, 2H), 2.83 (t, *J* = 7.7 Hz, 2H), 2.72 (m, 2H), 2.53 (d, *J* = 6.3 Hz, 2H), 2.22 (dd, *J* = 15.2, 4.0 Hz, 1H), 2.13 (s, 1H), 1.89 (d, *J* = 11.4 Hz, 1H).

#### Compound 29

Peptidomimetic 29 was synthesized according to general procedure A using 2-(2-(2-aminoethyl)phenyl)acetic acid hydrochloride (1.5 equiv.) in the second step (6.0 mg, 9%). HRMS (ESI/TOF) *m*/*z*: [M + H]^+^ calcd for C_27_H_35_N_4_O_6_S_2_ 575.1998; found 575.1984.

#### Compound 30

Peptidomimetic 30 was synthesized according to general procedure A using 2-(2-(aminomethyl)phenoxy)acetic acid hydrochloride (2.0 equiv.) and DIPEA (1.2 equiv.) in the second step (9.9 mg, 18%). HRMS (ESI/TOF) *m*/*z*: [M + H]^+^ calcd for C_26_H_33_N_4_O_7_S_2_ 571.1791; found 577.1782. ^1^H NMR (400 MHz, DMSO-*d*_6_) *δ* 13.03 (s, 1H), 9.22 (s, 1H), 8.56 (d, *J* = 7.3 Hz, 1H), 8.47 (d, *J* = 7.9 Hz, 1H), 8.32 (t, *J* = 6.0 Hz, 1H), 8.13 (m, 3H), 7.20 (td, *J* = 7.8, 1.7 Hz, 1H), 7.15 (dd, *J* = 7.5, 1.7 Hz, 1H), 7.07 (m, 2H), 6.91 (t, *J* = 7.5 Hz, 1H), 6.87 (d, *J* = 8.3 Hz, 1H), 6.68 (m, 2H), 6.54 (s, 1H), 4.71 (s, 2H), 4.43 (m, 1H), 4.28 (m, 2H), 4.07 (m, 1H), 3.76 (s, 1H), 3.00 (d, *J* = 7.3 Hz, 2H), 2.74 (m, 2H), 2.62 (m, 1H), 2.54 (s, 1H), 2.23 (dd, *J* = 15.2, 4.1 Hz, 1H), 1.92 (m, 1H).

#### Compound 31

Peptidomimetic 31 was synthesized according to general procedure A using 2-(3-(aminomethyl)phenoxy)acetic acid hydrochloride (2.0 equiv.) and DIPEA (1.2 equiv.) in the second step (14 mg, 25%). HRMS (ESI/TOF) *m*/*z*: [M + H]^+^ calcd for C_26_H_33_N_4_O_7_S_2_ 577.1791; found 577.1792. ^1^H NMR (400 MHz, DMSO-*d*_6_) *δ* 9.24 (s, 1H), 8.54 (d, *J* = 7.3 Hz, 1H), 8.49 (m, 2H), 7.21 (m, 1H), 7.07 (m, 2H), 6.82 (d, *J* = 7.6 Hz, 1H), 6.77 (d, *J* = 7.6 Hz, 2H), 6.68 (d, *J* = 8.4 Hz, 1H), 4.63 (s, 2H), 4.42 (t, *J* = 10.5 Hz, 1H), 4.24 (d, *J* = 6.0 Hz, 2H), 4.09 (m, 1H), 3.76 (d, *J* = 7.7 Hz, 1H), 2.98 (m, 2H), 2.73 (m, 2H), 2.68 (p, *J* = 1.8 Hz, 1H), 2.63 (m, 1H), 2.55 (s, 1H), 2.24 (dd, *J* = 15.1, 4.2 Hz, 1H), 2.10 (m, 1H), 1.90 (m, 1H).

#### Compound 32

Peptidomimetic 32 was synthesized according to general procedure A using 2-(2-(aminomethyl)phenyl)-*N*-methylacetamide (2.0 equiv.) in the second step (6.5 mg, 24%). HRMS (ESI/TOF) *m*/*z*: [M + H]^+^ calcd for C_27_H_36_N_5_O_5_S_2_ 574.2158; found 574.2142. ^1^H NMR (400 MHz, DMSO-*d*_6_) *δ* 9.21 (s, 1H), 8.54 (d, *J* = 7.3 Hz, 1H), 8.46 (m, 2H), 8.09 (m, 3H), 8.01 (d, *J* = 4.8 Hz, 1H), 7.19 (m, 3H), 7.06 (d, *J* = 8.4 Hz, 2H), 6.67 (m, 2H), 4.40 (m, 1H), 4.31 (d, *J* = 5.8 Hz, 2H), 4.08 (m, 1H), 3.75 (m, 1H), 3.48 (s, 2H), 2.98 (d, *J* = 7.3 Hz, 2H), 2.73 (m, 2H), 2.55 (m, 5H), 2.26 (dd, *J* = 15.3, 4.3 Hz, 1H), 2.11 (m, 2H), 1.89 (m, 1H), 1.23 (s, 1H).

#### Compound 33

Peptidomimetic 33 was synthesized according to general procedure A using 2-(2-(aminomethyl)phenyl)-*N*-benzylacetamide (2.0 equiv.) in the second step (1.7 mg, 6%). HRMS (ESI/TOF) *m*/*z*: [M + H]^+^ calcd for C_33_H_40_N_5_O_5_S_2_ 650.2471; found 650.2473.

#### Compound 34

Peptidomimetic 34 was synthesized according to general procedure A using (2-(aminomethyl)phenyl)methanol (2.0 equiv.) in the second step (12 mg, 22%). HRMS (ESI/TOF) *m*/*z*: [M + H]^+^ calcd for C_25_H_33_N_4_O_5_S_2_ 533.1892; found 533.1885. ^1^H NMR (400 MHz, DMSO-*d*_6_) *δ* 9.22 (q, *J* = 2.0 Hz, 1H), 8.55 (d, *J* = 7.3 Hz, 1H), 8.46 (d, *J* = 8.0 Hz, 1H), 8.37 (t, *J* = 5.9 Hz, 1H), 8.12 (s, 4H), 7.39 (m, 1H), 7.23 (m, 3H), 7.08 (m, 2H), 6.69 (m, 2H), 5.14 (t, *J* = 5.3 Hz, 1H), 4.53 (d, *J* = 5.2 Hz, 2H), 4.42 (t, *J* = 10.0 Hz, 1H), 4.30 (d, *J* = 6.0 Hz, 2H), 4.08 (q, *J* = 5.7, 5.3 Hz, 1H), 3.76 (s, 1H), 3.00 (d, *J* = 7.3 Hz, 2H), 2.75 (m, 2H), 2.23 (dd, *J* = 15.2, 4.1 Hz, 1H), 2.11 (m, 1H), 1.92 (m, 1H).

#### Compound 35

Peptidomimetic 35 was synthesized according to general procedure A using *cis*-3-aminocyclohexanecarboxylic acid (2.0 equiv.) in the second step (11 mg, 21%). HRMS (ESI/TOF) *m*/*z*: [M + H]^+^ calcd for C_24_H_35_N_4_O_6_S_2_ 539.1998; found 539.1993. ^1^H NMR (400 MHz, DMSO-*d*_6_) *δ* 9.22 (s, 1H), 8.50 (d, *J* = 7.4 Hz, 1H), 8.35 (d, *J* = 8.3 Hz, 1H), 7.92 (dd, *J* = 7.8, 3.9 Hz, 1H), 7.06 (m, 2H), 7.00 (m, 1H), 6.69 (m, 3H), 4.33 (t, *J* = 8.0 Hz, 1H), 4.07 (d, *J* = 7.4 Hz, 1H), 3.77 (s, 1H), 3.52 (d, *J* = 8.9 Hz, 2H), 2.99 (tt, *J* = 13.8, 6.9 Hz, 2H), 2.55 (s, 5H), 2.28 (m, 4H), 1.82 (m, 7H), 1.21 (m, 4H), 1.04 (d, *J* = 6.1 Hz, 1H).

#### Compound 36

Peptidomimetic 36 was synthesized according to general procedure A using 5-aminopentanoic acid (2.0 equiv.) in the second step (5.0 mg, 10%). HRMS (ESI/TOF) *m*/*z*: [M + H]^+^ calcd for C_22_H_33_N_4_O_6_S_2_ 513.1842; found 513.1821. ^1^H NMR (400 MHz, DMSO-*d*_6_) *δ* 9.22 (d, *J* = 11.7 Hz, 1H), 8.47 (d, *J* = 7.3 Hz, 1H), 8.36 (d, *J* = 8.1 Hz, 1H), 7.94 (t, *J* = 5.7 Hz, 1H), 7.05 (m, 2H), 6.68 (m, 2H), 6.53 (s, 1H), 4.32 (dd, *J* = 11.2, 7.4 Hz, 1H), 4.06 (d, *J* = 6.5 Hz, 1H), 3.72 (s, 1H), 3.00 (m, 4H), 2.73 (dd, *J* = 15.1, 5.3 Hz, 1H), 2.64 (m, 1H), 2.54 (s, 1H), 2.21 (m, 3H), 2.11 (s, 1H), 1.88 (t, *J* = 11.5 Hz, 1H), 1.42 (m, 4H), 1.23 (s, 1H).

#### Compound 37

Peptidomimetic 37 was synthesized according to general procedure A using 2-(aminomethyl)benzoic acid (2.0 equiv.) in the second step (5.5 mg, 11%). HRMS (ESI/TOF) *m*/*z*: [M + H]^+^ calcd for C_25_H_31_N_4_O_6_S_2_ 547.1685; found 547.1693. ^1^H NMR (400 MHz, DMSO-*d*_6_) *δ* 9.22 (s, 1H), 8.52 (m, 2H), 8.40 (m, 1H), 7.87 (dd, *J* = 8.1, 1.5 Hz, 1H), 7.51 (m, 1H), 7.36 (m, 2H), 7.07 (m, 2H), 6.67 (m, 2H), 6.53 (d, *J* = 4.5 Hz, 2H), 4.60 (m, 2H), 4.42 (m, 1H), 4.07 (m, 1H), 3.74 (m, 1H), 2.99 (d, *J* = 7.4 Hz, 2H), 2.74 (m, 2H), 2.54 (s, 3H), 2.23 (dd, *J* = 15.1, 4.0 Hz, 1H), 2.12 (m, 2H), 1.89 (m, 1H).

#### Compound 38

Peptidomimetic 38 was synthesized according to general procedure A using 3-(aminomethyl)benzoic acid (2.0 equiv.) in the second step (4.9 mg, 19%). HRMS (ESI/TOF) *m*/*z*: [M + H]^+^ calcd for C_25_H_31_N_4_O_6_S_2_ 547.1685; found 547.1680.

#### Compound 39

Peptidomimetic 39 was synthesized according to general procedure A using 4-(aminomethyl)benzoic acid (2.0 equiv.) in the second step (4.0 mg, 15%). HRMS (ESI/TOF) *m*/*z*: [M + H]^+^ calcd for C_25_H_31_N_4_O_6_S_2_ 547.1685; found 547.1671. ^1^H NMR (400 MHz, DMSO-*d*_6_) *δ* 9.22 (s, 1H), 8.59 (t, *J* = 6.1 Hz, 1H), 8.53 (d, *J* = 7.2 Hz, 1H), 8.48 (d, *J* = 7.8 Hz, 1H), 7.89 (d, *J* = 8.2 Hz, 2H), 7.35 (d, *J* = 8.1 Hz, 2H), 7.08 (d, *J* = 8.4 Hz, 2H), 6.68 (m, 2H), 4.42 (t, *J* = 10.1 Hz, 1H), 4.34 (d, *J* = 6.0 Hz, 2H), 4.08 (d, *J* = 6.7 Hz, 1H), 3.73 (d, *J* = 7.9 Hz, 1H), 2.99 (d, *J* = 7.0 Hz, 2H), 2.74 (m, 2H), 2.66 (m, 1H), 2.55 (s, 2H), 2.23 (dd, *J* = 15.1, 4.1 Hz, 1H), 2.10 (s, 1H), 1.90 (m, 1H).

### Fluorescence IRAP assay

IRAP inhibition was evaluated using a fluorescence-based assay, adapted from the method described by Gising *et al.*^33^ Briefly, the assay measured the inhibition of IRAP-catalyzed hydrolysis of l-leucine-7-amido-4-methylcoumarin in 384-well black microplates. Fluorescence was recorded with an excitation wavelength of 355 nm and an emission wavelength of 460 nm to quantify enzymatic activity. Dose–response curves were generated to assess compound potency.

### Computational methods

The crystal structure with PDB ID 6YDX containing the ligand HA08 was used as the starting point for the molecular dynamic simulations. The protein–ligand complexes were prepared for analysis using protein preparation in Maestro 2024-3.^42–44^ Molecular dynamic simulations were performed using Desmond using the OPLS4 force field.^45,46^ The simulation time was 500 ns at 310 K with standard pressure. The complexes of 37–39 were built from the HA08 crystal structure and minimized in place. The complexes of all ligands were solvated with TIP3P water, neutralized with sodium ions, and 0.15 M NaCl added with an exclusion volume 15 Å from the ligand. The integration time was adjusted to 1 fs, and the cut-off range for coulombic interactions was increased to 10 Å. Additional MD simulation details are available in SI in the MD simulation and PLS modelling details section including RMSD plots (Fig. S22–S25) for the movement of ligand and protein, and simulation quality statistics (Tables S3–S6).

We employed PLS regression as implemented in scikit learn to correlate protein–ligand contact patterns with binding affinities. All potential residue contacts between ligand and protein were extracted from all trajectory snapshots taken every 100 ns. Potential residue contacts were defined by distances of 4 Å between ligand and residue heavy atoms. The list was filtered to consist only of potential hydrogen bonds and π-stacking interactions of aromatic rings. The two distances involving carboxylate oxygens were replaced with distances to *C*_COOH_ atom in order to generate one distance to these moieties. Similarly, distances involving the nitrogen atoms of the guanidinium group of arginine residues were replaced with one distance, to the *C*_Z_ carbon. All final distances included after filtering can be found in SI (Table S7). The distances were used to calculate the occupancy time in contact as a percentage of the trajectory, as defined by a cut-off distance for each type of contact (see Table S8). The occupancy percentages were used as the *X* variables in the PLS model with the negative logarithm of experimental binding affinity as the *Y* variable. The complete time evolution plots of the distances with largest coefficients in the PLS model (absolute value over 0.06) can be found in Fig. S9. Regular snapshots of the MD trajectories are shown in Fig. S10–S21.

## Author contributions

Conceptualization, E. O. H., M. L. and L. R. O; methodology, E. O. H., L. J. I. B., S. P., S. M., H. H., C. S., B. S. and L. R. O.; validation, E. O. H., L. J. I. B., S. P. and S. M.; formal analysis, E. O. H., S. M., H. H., C. S.; investigation, E. O. H., L. J. I. B., S. P. and S. M.; data curation, E. O. H., S. M. and C. S.; writing – original draft preparation E. O. H., S. M., C. S., B. S., L. R. O., writing – review and editing, E. O. H., S. M., H. H., C. S., M. H., M. L., B. S. and L. R. O.; visualization, E. O. H., S. M., C. S., B. S. and L. R. O.; supervision, H. H., C. S., M. H., M. L., B. S. and L. R. O.; project administration, E. O. H., M. L., B. S. and L. R. O.; funding acquisition, M. L. and L. R. O.

## Conflicts of interest

There are no conflicts to declare.

## Supplementary Material

MD-016-D5MD00438A-s001

## Data Availability

Supplementary Information available: Tables S1–S8. Fig. S1–S25, synthesis procedures, characterisation data, dose-response curves, computational details. See DOI: https://doi.org/10.1039/D5MD00438A. Data for this article, including details on experimental procedures and characterization data of all compounds are available as part of the SI.
