# Supplementary material for: Novel macrocyclic peptidomimetics targeting the insulin-regulated aminopeptidase (IRAP): design, synthesis and evaluation
Source: RSC Med Chem. 2025 Aug 6;16(10):5059–69. doi: 10.1039/d5md00438a (PMC12378631; doi:10.1039/d5md00438a)
Supplement: MD-016-D5MD00438A-s001 [file MD-016-D5MD00438A-s001.pdf]

Supplementary Information (SI) for RSC Medicinal Chemistry.
This journal is © The Royal Society of Chemistry 2025

SUPPORTING INFORMATION

Novel Macrocyclic Peptidomimetics Targeting the Insulin-Regulated Aminopeptidase (IRAP): Design, Synthesis and Evaluation

Esther Olaniran Håkansson, [a] Lorenzo J.I. Balestri,[a] Sharathna Puthiyaparambath,[a] Sebastian Moes,[a] Henning Henschel,[a] Christian Sköld,[a] Mathias Hallberg,[b] Mats Larhed,[c] Bobo Skillinghaug[a], and Luke R. Odell[a]*

[a.] *Department of Medicinal Chemistry, BMC Uppsala University, P.O. Box 574, SE-751 23 Uppsala, Sweden *Email: luke.odell@ilk.uu.se*

[b.] *The Beijer Laboratory, Department of Pharmaceutical Biosciences, Neuropharmacology and Addiction Research, Biomedical Centre, Uppsala University, P.O. Box 591, SE-751 24 Uppsala, Sweden;*

[c.] *The Beijer Laboratory, Science for Life Laboratory, Department of Medicinal Chemistry, Biomedical Centre, Uppsala University, P.O. Box 574, SE-751 23 Uppsala, Sweden*

Contents

Synthesis of Intermediates and Non-Natural Amino Acid Building Blocks 5
Synthesis of macrocyclic tripeptide intermediate 4 5
Synthesis of non-natural amino acids 5
Synthesis of building blocks towards macrocyclic analogue 28. 6
Synthesis of building blocks towards macrocyclic analogue 29 7
Synthesis of building blocks towards macrocyclic analogues 30 and 31. 8
Synthesis of building blocks towards macrocyclic analogues 32 and 33 and acyl sulfonamide building block 9

Supplementary Tables 11
Investigation of conditions for reduction of azides 11
Optimization of conditions for synthesis of acyl sulfonamide-substituted benzylamine 12

Characterization of macrocyclic tripeptide 4 (LC-MS and ^{1}H NMR) 17
Compound 4, LC-MS 17
Compound 4, ^{1}H NMR (400 MHz, DMSO-d_6) spectrum 18

Characterization of Non-Natural Amino Acids 5, 9, 18-19, XXII-XXV and their building blocks II-IV, VI-VIII, XII-XVII and XXI (LC-MS, ^{1}H and ^{13}C NMR) 19
Compound S4, ^{1}H NMR (400 MHz, $CDCl_3$) spectrum 20
Compound S4, ^{13}C{1H} NMR (101 MHz, $CDCl_3$) spectrum 20
Compound II, LC-MS 21
Compound II, ^{1}H NMR (400 MHz, $CDCl_3$) spectrum 22
Compound II, ^{13}C{1H} NMR (101 MHz, $CDCl_3$) spectrum 22
Compound III, ^{1}H NMR (400 MHz, $CDCl_3$) spectrum 23
Compound III, ^{13}C{1H} NMR (101 MHz, $CDCl_3$) spectrum 23
Compound IV, ^{1}H NMR (400 MHz, $CDCl_3$) spectrum 24
Compound IV, ^{13}C{1H} NMR (101 MHz, $CDCl_3$) spectrum 24
Compound 5, LC-MS 25
Compound 5, ^{1}H NMR (400 MHz, MeOD) spectrum 26
Compound 5, ^{13}C{1H} NMR (101 MHz, MeOD) spectrum 26
Compound VI, ^{1}H NMR (400 MHz, $CDCl_3$) spectrum 27
Compound VI, ^{13}C{1H} NMR (101 MHz, $CDCl_3$) spectrum 27
Compound VII, LC-MS 28
Compound VII, ^{1}H NMR (400 MHz, $CDCl_3$) spectrum 29
Compound VII, ^{13}C{1H} NMR (101 MHz, $CDCl_3$) spectrum 29
Compound VIII, LC-MS 30
Compound VIII, ^{1}H NMR (400 MHz, $CDCl_3$) spectrum 31
Compound VIII, ^{13}C{1H} NMR (126 MHz, $CDCl_3$) spectrum 31
Compound 9, LC-MS 32
Compound 9, ^{1}H NMR (400 MHz, DMSO-d_6) spectrum 33
Compound 9, ^{13}C{1H} NMR (126 MHz, DMSO-d_6) spectrum 33
Compound XII, ^{1}H NMR (400 MHz, $CDCl_3$) spectrum 34
Compound XII, ^{13}C{1H} NMR (126 MHz, $CDCl_3$) spectrum 34
Compound XIII, ^{1}H NMR (400 MHz, $CDCl_3$) spectrum 35
Compound XIII, ^{13}C{1H} NMR (126 MHz, $CDCl_3$) spectrum 35
Compound XIV, LC-MS 36
Compound XIV, ^{1}H NMR (400 MHz, $CDCl_3$) spectrum 37
Compound XIV, ^{13}C{1H} NMR (126 MHz, $CDCl_3$) spectrum 37
Compound XV, LC-MS 38
Compound XV, ^{1}H NMR (400 MHz, $CDCl_3$) spectrum 39
Compound XV, ^{13}C{1H} NMR (126 MHz, $CDCl_3$) spectrum 39
Compound XVI, LC-MS 40

Compound XVI, ^{1}H NMR (400 MHz, $CDCl_3$) spectrum 41
Compound XVI, $^{13}C\{1H\}$ NMR (126 MHz, $CDCl_3$) spectrum 41
Compound XVII, LC-MS 42
Compound XVII, ^{1}H NMR (400 MHz, $CDCl_3$) spectrum 43
Compound XVII, $^{13}C\{1H\}$ NMR (101 MHz, $CDCl_3$) spectrum 43
Compound 18, LC-MS 44
Compound 18, ^{1}H NMR (400 MHz, DMSO-d_6) spectrum 45
Compound 18, $^{13}C\{1H\}$ NMR (101 MHz, DMSO-d_6) spectrum 45
Compound 19, LC-MS 46
Compound 19, ^{1}H NMR (400 MHz, DMSO-d_6) spectrum 46
Compound 19, $^{13}C\{1H\}$ NMR (101 MHz, DMSO-d_6) spectrum 47
Compound XXI, LC-MS 48
Compound XXI, ^{1}H NMR (400 MHz, DMSO-d_6) spectrum 49
Compound XXI, $^{13}C\{1H\}$ NMR (101 MHz, DMSO-d_6) spectrum 49
Compound XXII, LC-MS 50
Compound XXII, ^{1}H NMR (500 MHz, $CDCl_3$) spectrum 51
Compound XXII, $^{13}C\{1H\}$ NMR (126 MHz, $CDCl_3$) spectrum 51
Compound XXIII, LC-MS 52
Compound XXIII, ^{1}H NMR (500 MHz, $CDCl_3$) spectrum 53
Compound XXIII, $^{13}C\{1H\}$ NMR (126 MHz, $CDCl_3$) spectrum 53
Compound XXIV, LC-MS 54
Compound XXIV, ^{1}H NMR (500 MHz, $CDCl_3$) spectrum 55
Compound XXIV, $^{13}C\{1H\}$ NMR (126 MHz, $CDCl_3$) spectrum 55
Compound XXV, LC-MS 56
Compound XXV, ^{1}H NMR (500 MHz, $CDCl_3$) spectrum 57
Compound XXV, $^{13}C\{1H\}$ NMR (126 MHz, $CDCl_3$) spectrum 57

Characterization of Peptidomimetics 28-39 (LC-MS and ^{1}H NMR) 58
Compound 28, LC-MS 58
Compound 28, HRMS (ESI/TOF) 59
Compound 28, ^{1}H NMR (400 MHz, DMSO-d_6) spectrum 59
Compound 28, ^{19}F NMR (376 MHz, DMSO-d_6) spectrum 60
Compound 29, LC-MS 61
Compound 29, HRMS (ESI/TOF) 62
Compound 30, LC-MS 63
Compound 30, HRMS (ESI/TOF) 64
Compound 30, ^{1}H NMR (400 MHz, DMSO-d_6) spectrum 64
Compound 31, LC-MS 65
Compound 31, HRMS (ESI/TOF) 66
Compound 31, ^{1}H NMR (400 MHz, DMSO-d_6) spectrum 66
Compound 32, LC-MS 67
Compound 32, HRMS (ESI/TOF) 68
Compound 32, ^{1}H NMR (400 MHz, DMSO-d_6) spectrum 68
Compound 33, LC-MS 69
Compound 33, HRMS (ESI/TOF) 70
Compound 34, LC-MS 71
Compound 34, HRMS (ESI/TOF) 72
Compound 34, ^{1}H NMR (400 MHz, DMSO-d_6) spectrum 72
Compound 35, LC-MS 73
Compound 35, HRMS (ESI/TOF) 74
Compound 35, ^{1}H NMR (400 MHz, DMSO-d_6) spectrum 74
Compound 36, LC-MS 75
Compound 36, HRMS (ESI/TOF) 76
Compound 36, ^{1}H NMR (400 MHz, DMSO-d_6) spectrum 76

Compound 37, LC-MS 77
Compound 37, HRMS (ESI/TOF) 78
Compound 37, ^{1}H NMR (400 MHz, DMSO-d_6) spectrum 78
Compound 38, LC-MS 79
Compound 38, HRMS (ESI/TOF) 80
Compound 38, ^{1}H NMR (400 MHz, DMSO-d_6) spectrum 80
Compound 39, LC-MS 81
Compound 39, HRMS (ESI/TOF) 82
Compound 39, ^{1}H NMR (400 MHz, DMSO-d_6) spectrum 82

Dose-response curves for Peptidomimetics 28-39 83
Compound 28 84
Compound 29 84
Compound 30 84
Compound 31 85
Compound 32 85
Compound 33 85
Compound 34 86
Compound 35 86
Compound 36 86
Compound 37 87
Compound 38 87
Compound 39 87

MD simulation and PLS modelling details 88

References 117

Synthesis of Intermediates and Non-Natural Amino Acid Building Blocks

Synthesis of macrocyclic tripeptide intermediate 4

Compound **4** was prepared *via* standard Fmoc-based solid phase peptide synthesis (SPPS) on 2-chlorotrityl chloride (2-CTC) resin (loading: 1.51 mmol/g). The resin (199 mg, 0.300 mmol) was activated with $SOCl_2$ (26 µL, 0.36 mmol, 1.2 equiv.) and pyridine (58 µL, 0.72 mmol, 2.4 equiv.) in dry dichloromethane (DCM, 2 mL) for 6h at room temperature. The resin was then filtered and washed with dry DCM (6 × 1 min).

Fmoc-Cys(Trt)-OH (0.599 mmol) in dry DCM (2 mL) and DIPEA (2.10 mmol) were added, and the mixture was agitated at room temperature for 18 h. The resin was washed with DCM (6 × 1 min), and residual unreacted sites were capped using 2 mL of DCM/MeOH/DIPEA (86:10:4) for 1 h. The capping solution was removed, the reactor was flushed with nitrogen and the resin was washed with DCM (2 x 1 min) and dry DMF (2 x 1 min).

Fmoc deprotection was performed using 20% piperidine in DMF (3 x 10 min) followed by washing with DMF (6 x 1 min). Fmoc-β-homoTyr(*t*Bu)-OH and Fmoc-homoCys(Trt)-OH were coupled sequentially. Each coupling used the Fmoc-protected-amino acid (0.599 mmol), Oxyma (1.20 mmol), DIC (1.20 mmol) and DIPEA (2.10 mmol) in DMF (2 mL), pre-activated for five minutes before addition to the resin. Each reaction was allowed to proceed overnight at room temperature. The solution was emptied and the resin was washed with DMF (6 × 1 min) and DCM (3 × 1 min).

The protected linear peptide was cleaved and side chain protecting groups were removed by treating the resin with 2-3 mL of TFA/H_2O/TIS/1,2-Ethanedithiol (92.5:2.5:2.5:2.5) for 2 h at room temperature. The filtrate was concentrated under a stream of nitrogen, and the crude product was precipitated in cold diethyl ether. The solid was collected by centrifugation, washed twice with cold ether, and dried *in vacuo* to afford 138 mg of the Fmoc-protected linear tripeptide as a pale-yellow solid.

The crude linear tripeptide (138 mg) was dissolved in a 1:1 mixture of trifluoroacetic acid and acetonitrile (14 mL of each) and was stirred at room temperature for 72 h to promote cyclization. The reaction mixture was concentrated *in vacuo*, and the crude cyclic tripeptide was precipitated in cold diethyl ether, purified by preparative reversed-phase high-performance liquid chromatography (RP-HPLC), and lyophilized to give the macrocyclic tripeptide intermediate **4** in 40% total yield (across all steps). The identity and purity of the product were confirmed by analytical liquid chromatography-mass spectrometry (LC-MS). HRMS (ESI/TOF) m/z: $[M + H]^+$ Calcd for $C_{32}H_{34}N_3O_7S_2$ 636.1838; Found 636.1859. ^1H NMR (400 MHz, DMSO-d_6) δ 9.19 (s, 1H), 8.37 (d, *J* = 7.5 Hz, 1H), 8.09 (d, *J* = 7.3 Hz, 1H), 7.88 (d, *J* = 7.5 Hz, 2H), 7.73 (t, *J* = 7.7 Hz, 2H), 7.41 (t, *J* = 7.4 Hz, 2H), 7.33 (q, *J* = 8.0, 7.3 Hz, 3H), 7.04 (d, *J* = 8.1 Hz, 2H), 6.67 (d, *J* = 8.3 Hz, 2H), 4.24 (m, 4H), 4.08 (p, *J* = 7.0, 6.6 Hz, 2H), 2.90 (dd, *J* = 13.5, 8.4 Hz, 1H), 2.81 (m, 2H), 2.54 (m, 4H), 2.08 (m, 1H), 1.98 (s, 1H), 1.85 (m, 1H).

Synthesis of non-natural amino acids

General procedure B: Boc protection of lactams

The lactam (1.00 equiv.) was dissolved in dry DCM and stirred at 40 °C under nitrogen. Boc anhydride (2.00 equiv.) in DCM, triethylamine (1.00 equiv.) and DMAP (1.00 equiv.) were added. The reaction mixture was stirred overnight and then poured into a saturated $CuSO_4$ solution. The organic layer was separated, and the aqueous layer was extracted with ethyl acetate. The combined organic layers were dried with sodium sulfate, filtered and concentrated *in vacuo*.

General procedure C: Ring opening of Boc protected lactams

A solution of 1 N LiOH was added dropwise to a stirred solution of Boc-protected lactam in THF. The mixture was stirred at 50 °C overnight. Additional LiOH was added if necessary. After completion, the solvent was evaporated. The aqueous layer was acidified to pH 6 with 10% acetic acid (AcOH) and extracted with diethyl ether. The combined organic layers were dried over sodium sulfate, filtered and concentrated. The crude product was purified by flash column chromatography.

General procedure D: Boc deprotection

To a solution of the Boc-protected substrate, 4 M HCl in dioxane was added. The mixture was stirred at room temperature overnight and concentrated. Remaining solvent in the crude product was removed by co-evaporating with DCM and drying *in vacuo* overnight.

General procedure E: Boc protection of benzylamines

Boc anhydride (0.886 g, 4.06 mmol, 1.00 equiv.) was added to a solution of benzylamine (500 mg, 4.06 mmol, 1.00 equiv.) in water (5 mL) and tetrahydrofuran (THF, 10 mL). The mixture was stirred at room temperature overnight. THF was removed by rotary evaporation and the aqueous residue was extracted with ethyl acetate (3 × 20 mL). The combined organic layers were dried over sodium sulfate and concentrated *in vacuo*. The residue was purified by column chromatography (isohexane: ethyl acetate 5:1).

General procedure F: Alkylation of phenols with methyl bromoacetate

The phenol (0.664 g, 2.98 mmol, 1.00 equiv.) was added to a suspension of anhydrous Cs_2CO_3 (1.16 g, 3.57 mmol, 1.20 equiv.) in acetone (15 mL). After 15 min, methyl bromoacetate (0.34 mL, 3.57mmol, 1.2 equiv.) was added and the mixture was stirred for 2.5 h. The solids were removed by filtration, washed with acetone and the filtrate was concentrated *in vacuo*. The crude product was purified by column chromatography (isohexane: ethyl acetate 3:7).

General procedure G: Ester hydrolysis

The Boc-protected ester and potassium carbonate were suspended in a 2:1 mixture of acetonitrile and water, then irradiated in a microwave reactor at 100 °C for 20-25 min. After complete conversion, the mixture was acidified to pH 1 with 1 N HCl solution. The aqueous layer was extracted with ethyl acetate, the combined organic layers were dried over sodium sulfate and concentrated *in vacuo* to afford the carboxylic acid product.

General procedure H: amide coupling with EDCI*HCl and DMAP

To a solution of 2-(2-(((((9*H*-fluoren-9-yl)methoxy)carbonyl)amino)methyl)phenyl)acetic acid **XVII** in a round-bottom flask (25 mL), *N,N*-dimethylpyridin-4-amine (DMAP), and 1-ethyl-3-(3-dimethylaminopropyl)carbodiimide hydrochloride (EDCl*HCl) in DCM, the appropriate amine or sulfonamide was added. The resulting mixture was stirred at room temperature for 2-12 h, then cooled to 0 °C, and acidified to pH 1 with the 10% aqueous HCl. The mixture was extracted with DCM/MeOH (9:1, 3 × 50 mL). The combined organic layers were washed with water and brine, dried over sodium sulfate, and concentrated *in vacuo*.

Synthesis of building blocks towards macrocyclic analogue 28.

1,3,4,5-tetrahydro-2*H*-benzo[*c*]azepin-2-one (II)

A solution of β-tetralone I (1.81 mL, 13.7 mmol, 1.00 equiv.) in ethanol (12 mL) was treated with hydroxylamine hydrochloride (1.160 g, 16.69 mmol, 1.22 equiv.), sodium acetate (1.818 g, 22.16 mmol, 1.62 equiv.) and water (8 mL) and the mixture was stirred at room temperature. After 2h, water was added and the mixture was extracted with diethyl ether (3 x 30 mL). The combined organic phases were washed with brine, dried with $MgSO_4$, filtered and concentrated to yield the intermediate oxime **S4** as a yellow/brown oil in 94% yield. ^1H NMR (400 MHz, Chloroform-*d*) δ 9.38 (m, 1H), 7.29 (m, 4H), 3.97 (d, *J* = 4.6 Hz, 1H), 3.66 (d, *J* = 4.6 Hz, 1H), 2.99 (dq, *J* = 12.6, 6.4, 5.6 Hz, 2H), 2.86 (q, *J* = 6.8, 5.9 Hz, 1H), 2.71 (q, *J* = 6.5, 5.6 Hz, 1H). ^{13}C NMR (101 MHz, $CDCl_3$) δ 176.5, 159.7, 159.1, 138.3, 137.1, 134.7, 133.2, 129.0, 128.3, 127.8, 127.4, 126.8, 126.8, 126.3, 77.5, 77.2, 76.8, 35.0, 29.1, 28.9, 27.6, 24.5.

To the crude intermediate was added 10 mL DCM and thionyl chloride (6.6 mL, 90 mmol, 6.6 equiv.). The mixture was stirred at room temperature overnight after which it was poured into water and extracted with DCM (3 x 30 mL), washed with brine, dried with $MgSO_4$ and concentrated in vacuo. When subjected to isohexane, the crude formed a yellow precipitate. The suspension was centrifuged and the supernatant was decanted off. The yellow solid was dried in vacuo and the product **II** was obtained in a 9% yield. LRMS (ESI) m/z: $[M + H]^+$ Calcd for $C_{10}H_{12}NO$ 162.09; Found 162.1. ^1H NMR (400 MHz, Chloroform-*d*) δ 7.25 (m, 1H), 7.17 (m, 2H), 7.11 (dd, *J* = 7.5, 1.5 Hz, 1H), 4.36 (d, *J* = 4.6 Hz, 2H), 3.09 (m, 2H), 2.80 (m, 2H). ^{13}C NMR (101 MHz, $CDCl_3$) δ 175.7, 139.2, 136.2, 129.6, 128.4, 126.6, 45.9, 34.5, 28.6. Data in accordance with reported literature.[1]

tert-butyl 3-oxo-1,3,4,5-tetrahydro-2*H*-benzo[*c*]azepine-2-carboxylate (III)

General procedure B was followed using compound **II** (190 mg, 1.18 mmol, 1.00 equiv.), dry DCM (6 mL), Boc_2O (514 mg, 2.36 mmol, 2.00 equiv.) in DCM (1 mL), triethylamine (164 µL, 1.18 mmol, 1.00 equiv.) and DMAP (144 mg, 1.18 mmol, 1.00 equiv.). The reaction mixture was poured into saturated aqueous $CuSO_4$ solution (5 mL) and extracted with ethyl acetate (3 × 15 mL) to obtain the title compound **IV** as a white solid in 57% yield (176 mg). ^1H NMR (400 MHz, Chloroform-*d*) δ 7.25 (m, 1H), 7.15 (m, 3H), 4.94 (s, 2H), 3.26 (m, 2H), 3.06 (dd, *J* = 7.7, 5.8 Hz, 2H), 1.50 (s, 9H). ^{13}C NMR (101 MHz, $CDCl_3$) δ 173.9, 151.9, 136.8, 134.1, 130.3, 129.7, 128.4, 126.4, 83.3, 49.1, 36.5, 29.2, 28.2.

3-(2-(((tert-butoxycarbonyl)amino)methyl)phenyl)propanoic acid (IV)

General procedure C was followed using 1 N LiOH (4.0 mL, 4.0 mmol) and boc protected lactam **III** (176 mg, 0.674 mmol, 1.00 equiv.) in THF (3 mL). After stirring overnight at 50 °C, the reaction was still incomplete and 48 mg of LiOH was added. After 30 minutes the reaction was pushed towards completion. The aqueous phase was extracted with diethyl ether (3 × 15 mL) and the crude was purified by flash column chromatography (isohexane:diethyl ether 3:1 + 0.5% AcOH) to yield title compound **VI** (95 mg, 51%). ^{1}H NMR (400 MHz, Chloroform-*d*) δ 7.22 (m, 4H), 4.92 (s, 1H), 4.32 (m, 2H), 2.99 (t, *J* = 7.9 Hz, 2H), 2.66 (t, 2H), 1.45 (s, 9H). ^{13}C NMR (101 MHz, $CDCl_3$) δ 178.0, 156.0, 138.5, 136.4, 129.2, 129.0, 128.0, 127.0, 79.9, 42.3, 35.0, 28.5, 27.1.

3-(2-(aminomethyl)phenyl)propanoic acid hydrochloride (5)

General procedure D was followed using amide **IV** (98.0 mg, 0.351 mmol, 1.00 equiv.) and 4 M HCl in dioxane (3 mL) at room temperature overnight. The mixture was concentrated to afford a yellow/transparent oil which was the HCl salt of compound **5** (75 mg, 99%). LRMS (ESI) m/z: $[M + H]^+$ Calcd for $C_{10}H_{14}NO_2$ 180.10; Found 180.4. ^{1}H NMR (400 MHz, Methanol-*d*$_4$) δ 7.37 (m, 4H), 4.25 (d, *J* = 4.9 Hz, 2H), 3.01 (q, *J* = 6.6 Hz, 2H), 2.76 (p, *J* = 7.1 Hz, 2H). ^{13}C NMR (101 MHz, MeOD) δ 176.9, 141.3, 132.4, 131.1, 130.8, 130.6, 128.2, 41.5, 35.6, 27.4.

Synthesis of building blocks towards macrocyclic analogue 29.

1,3,4,5-tetrahydro-2*H*-benzo[*d*]azepin-2-one (VI)

β-tetralone **I** (2.70 mL, 20.5 mmol, 1.00 equiv.) was dissolved in methanesulfonic acid (2.15 mL, 33.1 mmol, 1.62 equiv.) and cooled to 0 °C. Thereafter, NaN_3 (1.73 g, 26.6 mmol, 1.30 equiv.) was added over 15 minutes. The reaction mixture was allowed to warm to room temperature and stirred overnight. The reaction mixture/cake was cooled on ice bath and sat. $NaHCO_3$ was added until slightly basic then extracted with DCM (3 x 50 mL). The organic phase was dried with sodium sulfate, filtered and concentrated *in vacuo*. The crude mixture was triturated in a mixture of DCM and isohexane leading to the precipitation of the title compound **VI** as a yellow solid (678 mg, 21%). The crude product was used in the next step without further purification. ^{1}H NMR (400 MHz, Chloroform-*d*) δ 7.15 (m, 4H), 6.57 (m, 1H), 3.84 (s, 2H), 3.57 (m, 2H), 3.12 (m, 2H). ^{13}C NMR (101 MHz, $CDCl_3$) δ 173.9, 137.0, 131.9, 130.5, 130.0, 127.4, 126.9, 42.5, 41.5, 33.4. Data in accordance with reported literature.[2]

tert-butyl 2-*oxo*-1,2,4,5-tetrahydro-3*H*-benzo[*d*]azepine-3-carboxylate (VII)

General procedure B was followed using **VI** (614 mg, 3.81 mmol, 1.00 equiv.), dry DCM (18 mL), Boc_2O (1.663 g, 7.618 mmol, 2.00 equiv.) in DCM (1 mL), triethylamine (531 µL, 3.81 mmol, 1.00 equiv.) and DMAP (465 mg, 3.81 mmol, 1.00 equiv.). 10 mL of saturated aqueous $CuSO_4$ solution was used and the reaction mixture was extracted with 3 × 50 mL ethyl acetate to give 429mg (43%) of the title compound **VII** as a white solid. LRMS (ESI) m/z: $[M - C_4H_9 + H]^+$ Calcd for $C_{11}H_{12}NO_3$ 206.08; Found 206.2. ^{1}H NMR (400 MHz, Chloroform-*d*) δ 7.15 (m, 4H), 4.20 (m, 2H), 4.02 (s, 2H), 3.22 (m, 2H), 1.53 (s, 9H). ^{13}C NMR (101 MHz, $CDCl_3$) δ 171.5, 152.2, 135.3, 131.5, 130.7, 130.2, 127.7, 126.8, 83.4, 46.0, 43.5, 33.3, 28.2.

2-[2-[2-(tert-butoxycarbonylamino)ethyl]phenyl]acetic acid (VIII)

General procedure C was followed using Boc-protected lactam **VII** (416 mg, 1.59 mmol, 1.00 equiv.), LiOH (343 mg, 14,3 mmol, 9.00 equiv.), THF (8 mL) and water (4.8 mL) was heated at 80 °C for 5h. Then the solvent was exaporated and the remaining aqueous phase was acidified with 10% AcOH to pH6 and extracted with ether (3 x 30 mL and crude was purified by flash column chromatography (isohexane:diethyl ether 3:1 + 0.5% AcOH) to yield 376 mg (85%) of compound **VIII**. LRMS (ESI) m/z: $[2M + H]^+$ Calcd for $C_{30}H_{43}N_2O_8$ 559.31; Found 558.8. ^{1}H NMR (400 MHz, Chloroform-*d*) δ 7.22 (ddd, *J* = 14.5, 11.3, 5.3 Hz, 5H), 3.73 (s, 2H), 3.34 (s, 2H), 2.84 (t, *J* = 7.3 Hz, 2H), 1.43 (s, 9H). ^{13}C NMR (126 MHz, $CDCl_3$) δ 176.9, 176.7, 137.7, 132.3, 131.0, 130.1, 128.0, 127.1, 41.3, 38.5, 33.4, 28.5, 20.8.

2-[2-(2-aminoethyl)phenyl]acetic acid hydrochloride (9)

General procedure D was followed using amide **VIII** (339 mg, 1.21 mmol, 1.00 equiv.) and 4 M HCl in dioxane solution (12 mL) at room temperature overnight. The solvent was evaporated to afford the title compound **9** as yellow crystals (255 mg, 97%). LRMS (ESI) m/z: $[M + H]^+$ Calcd for $C_{10}H_{14}NO_2$ 180.10; Found 180.2. ^{1}H NMR (500 MHz, DMSO-*d*$_6$) δ 12.46 (s, 1H), 8.15 (s, 3H), 7.22 (m, 4H), 3.66 (s, 2H), 2.91 (m, 4H). ^{13}C NMR (126 MHz, DMSO) δ 172.8, 136.3, 133.8, 130.9, 129.5, 127.3, 126.9, 38.0, 30.2.

Synthesis of building blocks towards macrocyclic analogues 30 and 31.

tert-butyl (2-hydroxybenzyl)carbamate (XII)

General procedure E was followed using 2-(aminomethyl) phenol **X** to afford the product **XII** as white crystalline solid (664 mg, 73%). ^{1}H NMR (400 MHz, Chloroform-*d*) δ 8.88 (s, 1H), 7.21 (ddd, *J* = 8.1, 7.3, 1.7 Hz, 1H), 7.06 (dd, *J* = 7.3, 1.7 Hz, 1H), 6.94 (dd, *J* = 8.1, 1.2 Hz, 1H), 6.82 (td, *J* = 7.3, 1.2 Hz, 1H), 5.24 (s, 1H), 4.23 (d, *J* = 6.8 Hz, 2H), 1.45 (s, 9H). ^{13}C NMR (126 MHz, $CDCl_3$) δ 157.4, 154.8, 129.6, 128.8, 123.8, 118.8, 116.7, 80.3, 76.3, 76.0, 75.7, 40.3, 27.3. Data in accordance with reported literature.[3]

tert-butyl (3-hydroxybenzyl)carbamate (XIII)

General procedure E was followed using 3-(aminomethyl) phenol **XI** to afford the product **XIII** as white crystalline solid (786 mg, 87%). ^{1}H NMR (400 MHz, Chloroform-*d*) δ 7.15 (m, 1H), 6.74 (m, 3H), 4.96 (s, 1H), 4.24 (m, 2H), 1.46 (s, 9H). ^{13}C NMR (126 MHz, $CDCl_3$) δ 156.6, 156.4, 140.4, 129.9, 119.3, 114.7, 114.4, 80.1, 44.6, 28.5. Data in accordance with reported literature.[4]

methyl 2-(2-(((tert-butoxycarbonyl)amino)methyl)phenoxy)acetate (XIV)

General procedure F was followed using **XII** (664 mg, 2.98 mmol, 1.00 equiv.), anhydrous Cs_2CO_3 (1.16 g, 3.57 mmol, 1.20 equiv.), acetone (15 mL) and methyl bromoacetate (34 µL, 3.57mmol, 1.20 equiv.) to afford the product **XIV** as an oily liquid (746 mg, 85%). LRMS (ESI) m/z: $[M + H]^+$ Calcd for $C_{15}H_{22}NO_5$ 296.15; Found 296.1, $[2M + H]^+$ Calcd for $C_{30}H_{43}N_2O_{10}$ 591.29; Found 590.9. ^{1}H NMR (400 MHz, Chloroform-*d*) δ 7.31 (d, *J* = 7.4 Hz, 1H), 7.22 (td, *J* = 7.9, 1.7 Hz, 1H), 6.96 (td, *J* = 7.4, 1.1 Hz, 1H), 6.75 (dd, *J* = 8.2, 1.1 Hz, 1H), 5.47 (s, 1H), 4.69 (s, 2H), 4.36 (d, *J* = 6.2 Hz, 2H), 3.81 (s, 3H), 1.44 (s, 9H). ^{13}C NMR (126 MHz, $CDCl_3$) δ 169.5, 156.1, 156.0, 130.1, 128.8, 128.1, 122.0, 111.3, 79.2, 65.4, 52.5, 40.7, 28.6.

methyl 2-(3-(((tert-butoxycarbonyl)amino)methyl)phenoxy)acetate (XV)

General procedure F was followed using **XIII** (786 mg, 3.52 mmol, 1.00 equiv.), anhydrous Cs_2CO_3 (1.4 g, 4.23mmol, 1.20 equiv.), acetone (15 mL) and methyl bromoacetate (400 µL, 4.23mmol, 1.20 equiv.) to afford the product **XIII** as oily liquid (799 mg, 78%). LRMS (ESI) m/z: $[2M + H]^+$ Calcd for $C_{30}H_{43}N_2O_{10}$ 591.29; Found 590.9. ^{1}H NMR (400 MHz, Chloroform-*d*) δ 7.17 (m, 1H), 6.84 (d, *J* = 7.6 Hz, 1H), 6.78 (t, *J* = 2.0 Hz, 1H), 6.72 (m, 1H), 4.76 (d, *J* = 13.1 Hz, 1H), 4.56 (s, 2H), 4.22 (d, *J* = 5.9 Hz, 2H), 3.74 (s, 3H), 1.39 (s, 9H). ^{13}C NMR (126 MHz, $CDCl_3$) δ 169.5, 158.1, 156.0, 141.0, 129.9, 120.9, 113.9, 113.3, 79.7, 65.3, 52.4, 44.6, 28.5.

2-(2-(((tert-butoxycarbonyl)amino)methyl)phenoxy)acetic acid (XVI)

General procedure G was followed using ester **XIV** (695 mg, 2.35 mmol, 1.00 equiv.), potassium carbonate (488mg, 5.88 mmol, 2.50 equiv.) and acetonitrile/H_2O (15 mL) to afford the acid **XVI** as white amorphous solid (637 mg, 96%). LRMS (ESI) m/z: $[M - H]^-$ Calcd for $C_{14}H_{18}NO_5$ 280.12; Found 280.2. ^{1}H NMR (400 MHz, Chloroform-*d*) δ 7.29 (m, 1H), 7.22 (m, 1H), 6.98 (m, 1H), 6.80 (d, *J* = 8.2 Hz, 1H), 5.14 (s, 1H), 4.65 (s, 2H), 4.39 (s, 2H), 1.43 (s, 9H). ^{13}C NMR (126 MHz, $CDCl_3$) δ 170.6, 156.8, 155.3, 130.6, 129.5, 127.5, 122.1, 111.6, 80.5, 65.3, 40.6, 28.5.

2-(3-(((tert-butoxycarbonyl)amino)methyl)phenoxy)acetic acid (XVII)

General procedure G was followed using ester **XV** (799 mg, 2.71 mmol, 1.00 equiv.), potassium carbonate (0.488, 4.06mmol, 1.50 equiv.) and acetonitrile/H_2O (15 mL) to afford the acid **XVII** as white amorphous solid (680 mg, 87%). LRMS (ESI) m/z: $[M - H]^-$ Calcd for $C_{14}H_{18}NO_5$ 280.12; Found 280.1. ^{1}H NMR (400 MHz, Chloroform-*d*) δ 7.16 (d, *J* = 8.0 Hz, 1H), 6.78 (m, 3H), 4.57 (s, 2H), 4.22 (m, 2H), 1.39 (s, 9H). ^{13}C NMR (126 MHz, $CDCl_3$) δ 172.4, 157.9, 156.2, 140.9, 130.0, 129.8, 121.0, 120.8, 113.9, 113.4, 80.0, 65.0, 44.6, 28.5.

2-(2-(aminomethyl)phenoxy)acetic acid hydrochloride (18)

General procedure D was followed using Boc-protected amine **XVI** (200 mg, 0.711 mmol) and 4 M HCl in dioxane (6 mL) to afford compound **18** as white solid (158 mg, 96%). LRMS (ESI) m/z: $[M + H]^+$ Calcd for $C_9H_{12}NO_3$ 182.08; Found 182.3. ^{1}H NMR (400 MHz, DMSO-d_6) δ 13.21 (s, 1H), 8.43 (s, 3H), 7.43 (d, *J* = 7.3 Hz, 1H), 7.34 (t, *J* = 7.8 Hz, 1H), 7.02 (m, 2H), 4.79 (d, *J* = 2.5 Hz, 2H), 4.01 (t, *J* = 5.6 Hz, 2H). ^{13}C NMR (101 MHz, DMSO) δ 170.5, 155.8, 130.3, 130.1, 122.3, 121.1, 112.3, 65.1, 37.8.

2-(3-(aminomethyl)phenoxy)acetic acid hydrochloride (19)

General procedure D was followed using Boc protected amine **XVII** (210 mg, 0.748 mmol) and 4 M HCl in dioxane (6 mL) to afford product **19** as white solid (158 mg, 97%). LRMS (ESI) m/z: $[M + H]^+$ Calcd for $C_9H_{12}NO_3$ 182.08; Found 182.2. ^1H NMR (400 MHz, DMSO-d_6) δ 8.54 (s, 3H), 7.31 (td, *J* = 7.9, 2.8 Hz, 1H), 7.10 (dd, *J* = 24.3, 4.9 Hz, 2H), 6.90 (dq, *J* = 8.1, 4.0, 3.1 Hz, 1H), 4.68 (d, *J* = 2.9 Hz, 2H), 3.96 (q, *J* = 6.1, 5.6 Hz, 2H). ^{13}C NMR (101 MHz, DMSO) δ 170.0, 157.8, 135.6, 129.7, 121.5, 115.3, 114.1, 64.4, 42.0.

Synthesis of building blocks towards macrocyclic analogues 32 and 33 and acyl sulfonamide building block.

Synthesis of XXI by Fmoc-protection of XX.

2-[2-(aminomethyl)phenyl]acetic acid **XX** (1.50 g, 9.98 mmol, 1.00 equiv.) and Fmoc-Cl (2.81 g, 10.9 mmol, 1.20 equiv.) were dissolved in 1,4-dioxane (20 mL) and 10 wt% sodium carbonate (18 mL) and the mixture was stirred at room temperature for 4 h. The reaction mixture was acidified with 1 M HCl followed by extraction with ethyl acetate (3 x 30 mL). The combined organic phases were washed with brine, dried over Na_2SO_4 and concentrated under reduced pressure. The oily crude was purified by flash column chromatography (gradient elution DCM:ethyl acetate 9:1 + 1% formic acid) to obtain the product **XXI** as a white fluffy solid (2.10 g, 60%). LRMS (ESI) m/z: $[M + H]^+$ Calcd for $C_{24}H_{22}NO_4$ 388.15; Found 388.1, $[2M + H]^+$ Calcd for $C_{48}H_{43}N_2O_8$ 775.30; Found 775.1. ^1H NMR (400 MHz, DMSO-d_6) δ 12.37 (s, 1H), 7.89 (d, *J* = 7.5 Hz, 2H), 7.77 (t, *J* = 6.0 Hz, 1H), 7.70 (d, *J* = 7.5 Hz, 2H), 7.42 (t, *J* = 7.4 Hz, 2H), 7.33 (td, *J* = 7.4, 1.2 Hz, 2H), 7.21 (m, 4H), 4.34 (d, *J* = 7.0 Hz, 2H), 4.22 (dd, *J* = 12.8, 6.3 Hz, 3H), 3.67 (s, 2H). ^{13}C NMR (101 MHz, DMSO) δ 172.6, 156.3, 143.9, 140.8, 138.0, 132.9, 130.5, 127.6, 127.5, 127.1, 126.9, 126.8, 125.2, 120.1, 65.4, 46.8, 41.4, 38.0.

Synthesis of amides and acyl sulfonamides XXII-XXV.

(9*H*-fluoren-9-yl)methyl (2-(2-(methylamino)-2-oxoethyl)benzyl)carbamate (XXII)

General procedure H was followed using **XXI** (240 mg, 0.619 mmol, 1.00 equiv.), DMAP (163 mg, 1.33 mmol, 2.15 equiv.), EDCl*HCl (255 mg, 1.33 mmol, 2.15 equiv.), methylamine (2 M in MeOH, 341.0 µL, 0.68 mmol,1.1 equiv.) and DCM (6.5 mL). The crude residue was purified by flash column chromatography (gradient elution isohexane:ethyl acetate 1:9) as white solid (67.0 mg, 27% yield). LRMS (ESI) m/z: $[M + H]^+$ Calcd for $C_{25}H_{25}N_2O_3$ 401.19; Found 401.3. ^1H NMR (500 MHz, Chloroform-*d*) δ 7.76 (dt, *J* = 7.5, 1.0 Hz, 2H), 7.60 (d, *J* = 7.5 Hz, 2H), 7.39 (tt, *J* = 7.5, 0.9 Hz, 2H), 7.29 (m, 5H), 7.25 (d, *J* = 6.0 Hz, 3H), 5.81 (s, 1H), 5.76 (s, 1H), 4.40 (dd, *J* = 9.1, 6.4 Hz, 4H), 4.22 (t, *J* = 7.0 Hz, 1H), 3.59 (s, 2H), 2.77 (d, *J* = 4.8 Hz, 3H). ^{13}C NMR (126 MHz, $CDCl_3$) δ 171.3, 144.0, 141.4, 137.1, 133.7, 131.1, 130.0, 128.5, 128.2, 127.8, 127.2, 125.3, 120.1, 66.9, 47.4, 43.1, 40.9, 26.6.

(9*H*-fluoren-9-yl)methyl (2-(2-(benzylamino)-2-oxoethyl)benzyl)carbamate (XXIII)

General procedure H was followed using **XXI** (120 mg, 0.310 mmol, 1.00 equiv.), DMAP (81.4 mg, 0.666 mmol, 2.15 equiv.), EDCl*HCl (128 mg, 0.666 mmol, 2.15 equiv.), benzylamine (37 µL, 0.34 mmol, 1.1 equiv.) and DCM (7.0 mL). Afterwards, a solution of isohexane:DCM (8:2) was added and the product **XXIII** precipitated out as a white dusty solid (91 mg, 62%). LRMS (ESI) m/z: $[M + H]^+$ Calcd for $C_{31}H_{29}N_2O_3$ 477.22; Found 477.3. ^1H NMR (500 MHz, Chloroform-*d*) δ 7.75 (d, *J* = 7.5 Hz, 2H), 7.57 (s, 2H), 7.39 (m, 3H), 7.27 (m, 12H), 4.41 (t, *J* = 6.1 Hz, 4H), 4.36 (d, *J* = 7.4 Hz, 2H), 4.19 (t, *J* = 7.1 Hz, 1H), 3.64 (s, 2H). ^{13}C NMR (126 MHz, $CDCl_3$) δ 170.8, 156.7, 144.0, 141.4, 138.1, 137.1, 133.6, 131.0, 130.2, 128.8, 128.5, 128.2, 127.8, 127.8, 127.7, 127.2, 125.3, 120.1, 66.9, 47.4, 43.9, 43.2, 41.0.

(9*H*-fluoren-9-yl)methyl (2-(2-(methylsulfonamido)-2-oxoethyl)benzyl)carbamate (XXIV)

General procedure H was followed using **XXI** (200 mg, 0.516 mmol, 1.00 equiv.), DMAP (202 mg, 1.65 mmol, 3.20 equiv.), EDCl*HCl (213 mg, 1.11 mmol, 2.15 equiv.), methanesulfonamide (147 mg, 1.55 mmol, 3.00 equiv.) and DCM (13 mL). The crude residue was purified by flash column chromatography (gradient elution isohexane:ethyl actetate 4:6 + 1% AcOH) as white solid (53.6 mg 23% yield). LRMS (ESI) m/z: $[M + H]^+$ Calcd for $C_{25}H_{25}N_2O_2S$ 465.15; Found 465.3. ^1H NMR (500 MHz, Chloroform-*d*) δ 9.56 (s, 1H), 7.71 (dd, *J* = 7.8, 3.7 Hz, 2H), 7.54 (m, 2H), 7.35 (m, 2H), 5.75 (s, 1H), 4.37 (t, *J* = 5.2 Hz, 2H), 4.29 (s, 2H), 4.17 (t, *J* = 6.0 Hz, 1H), 3.70 (s, 2H), 3.17 (d, *J* = 3.8 Hz, 3H). ^{13}C NMR (126 MHz, $CDCl_3$) δ 170.2, 156.9, 143.7, 141.2, 136.7, 131.4, 130.5, 130.0, 128.3, 128.2, 127.6, 127.0, 125.1, 119.9, 116.5, 67.0, 47.0, 42.5, 41.1, 39.9.

(9*H*-fluoren-9-yl)methyl (2-(2-oxo-2-(phenylsulfonamido)ethyl)benzyl)carbamate (XXV)

General procedure H was followed using **XXI** (50.0 mg, 0.129 mmol, 1.00 equiv.), DMAP (33.9 mg, 0.277 mmol, 2.15 equiv.), and EDCl*HCl (53.2 mg, 0.277 mmol, 2.15 equiv.), benzenesulfonamide (60.9 mg, 0.387 mmol, 3.00 equiv.) and DCM (3.23 mL). The crude residue was purified by flash column chromatography (gradient elution ethyl actetate:AcOH 1:0.1) as white solid (88.5 mg, 33%). LRMS (ESI) m/z: $[M + H]^+$ Calcd for $C_{30}H_{27}N_2O_5S$ 527.16; Found 527.3, $[2M + H]^+$ Calcd for $C_{60}H_{53}N_4O_{10}S_2$ 1053.32; Found 1053.8. ^1H NMR (500 MHz, Chloroform-*d*) δ 9.77 (s, 1H), 8.01 (d, *J* = 7.8 Hz, 2H), 7.76 (d, *J* = 7.5 Hz, 2H), 7.59 (m, 4H), 7.46 (t, *J* = 7.7 Hz, 2H), 7.39 (t, *J* = 7.5 Hz, 2H), 7.29 (t, *J* = 7.5 Hz, 2H), 7.22 (d, *J* = 43.8 Hz, 3H), 5.40 (t, *J* = 6.3 Hz, 1H), 4.50 (d, *J* = 6.6 Hz, 2H), 4.22 (t, *J* = 6.6 Hz, 3H), 3.61 (s, 2H).
^{13}C NMR (126 MHz, $CDCl_3$) δ 170.2, 156.9, 143.7, 141.2, 136.7, 131.4, 130.5, 130.0, 128.3, 128.2, 127.6, 127.0, 125.1, 119.9, 116.5, 67.0, 47.0, 42.5, 41.1, 39.9.

Supplementary Tables

Investigation of conditions for reduction of azides

In all explored conditions, we observed formation of the lactam **S2a** as either the sole product or the major component of a complex mixture. A similar phenomenon was also observed during *N*-Fmoc-protection of AMPA (**Table S2**). The use of the *tert*-butyl ester slowed the intramolecular cyclization to the δ-lactam **S2a** sufficiently to isolate the desired product **S3b**.

Table S1. Investigation of conditions to avoid formation of δ-lactam **S2** through intramolecular cyclization of δ-azido derivatives **S1a**-**c**.

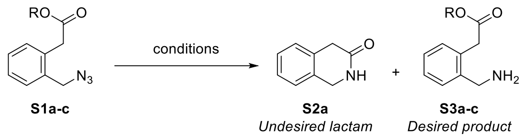

Entry	Target compound	R-group	Conditions	Comment
1	**S3a**	-ethyl	PPh_3 (1.1 equiv.), THF/H_2O	**S2a** (only product[a])
2	**S3a**	-ethyl	H_2, Pd-C (10mol%) in MeOH	**S2a** (only product[a])
3	**XX**	-H	H_2, Pd-C (10mol%) in MeOH	**S2a** (only product[a])
4	**S3b**	-*t*Bu	H_2, Pd-C (10mol%) in MeOH	**S2a** (major product[a]) + **S3b** (13%[b])

[a]Determined by LC-MS, [b]Isolated yield

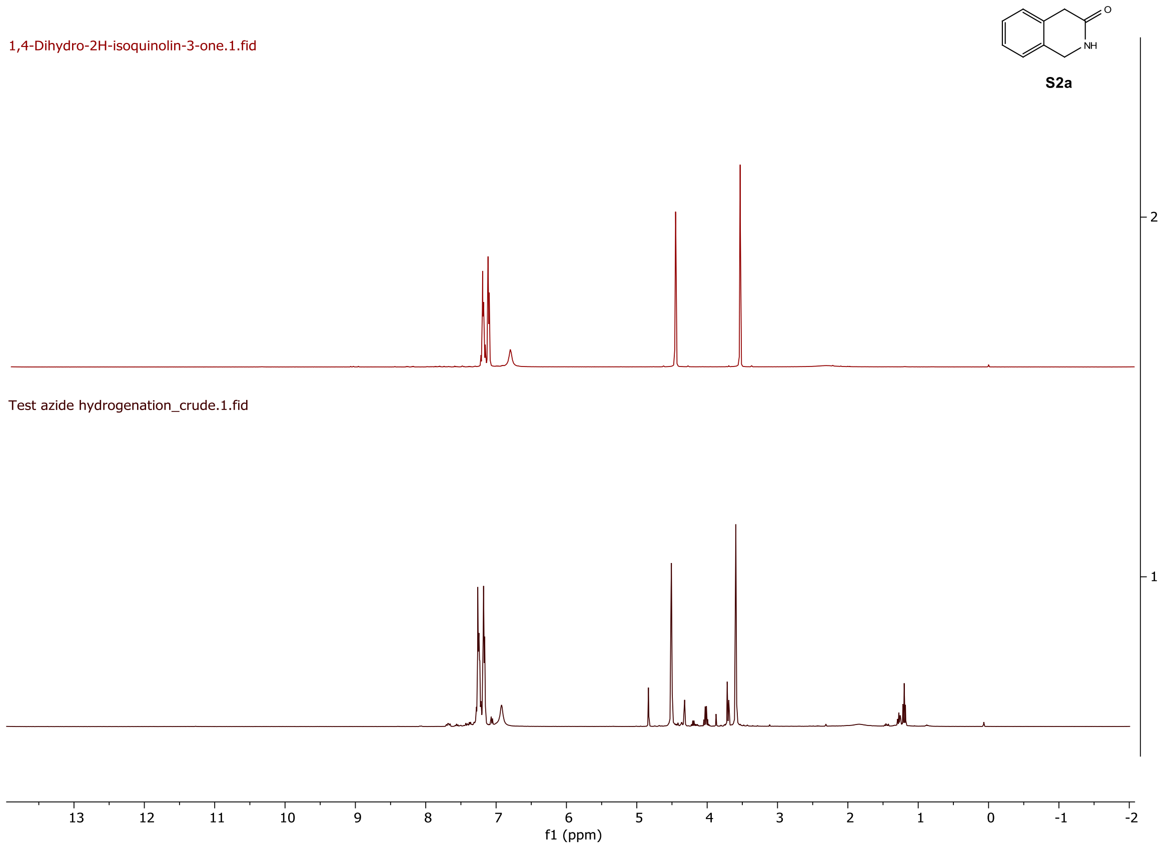

Figure S1. ^{1}H NMR spectra of reference compound 1,4-dihydro-2H-isoquinolin-3-one and isolated side product after attempted reduction of **S1a** to **S3a** (Table S1, Entry 2).

Optimization of conditions for synthesis of acyl sulfonamide-substituted benzylamine

Fmoc-protection of **XX** yielded 60% of **XXI**. Using T_3P for coupling of **XXI** with benzenesulfonamide in EtOAc resulted in exclusive formation of side product **S2b** (**Entry 1**, **Figure S2**). The use of Boc anhydride in DMF with pyridine as base did not improve the outcome (**Entry 2**, **Figure S3**). Employing EDC*HCl and DMAP in DCM did result in formation of **XXV**, although **S2b** was still the major product. The fact that the product concentration remained stable overtime (2h - overnight) indicates that the side product is formed directly from the activated ester that is formed by reaction between the carboxylic acid of **XXI** and EDC, rather than from the product.

Table S2. Optimization of reaction conditions for synthesis of acyl sulfonamide **XXV**.[a]

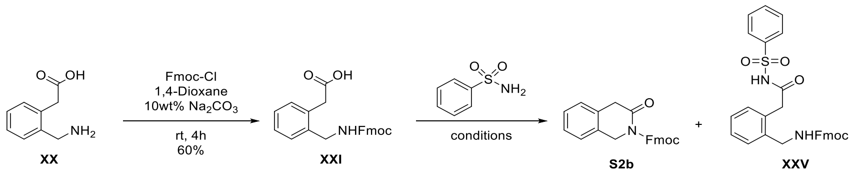

Entry	Conditions	Product
1	T_3P (2.00 equiv.), EtOAc, rt, 17h	**S2b** (major product[a])
2	Boc_2O (2.95 equiv.), pyridine (3.00 equiv.), DMF, rt, 17h	**S2b** (major product[a])
3	EDC*HCl (2.15 equiv.), DMAP (2.15 equiv.), DCM, rt, 4h	**S2b:XXV** (3:2[a])

[a]Determined by LC-MS

**Sample Name:**	BZ9415-rxm, r.t. O/N	**Injection Volume:**	0.9 uL
**Control Program:**	5-100% positive	**UserID**	Lorenzo
**Recording Time:**	3/14/2023 11:30		

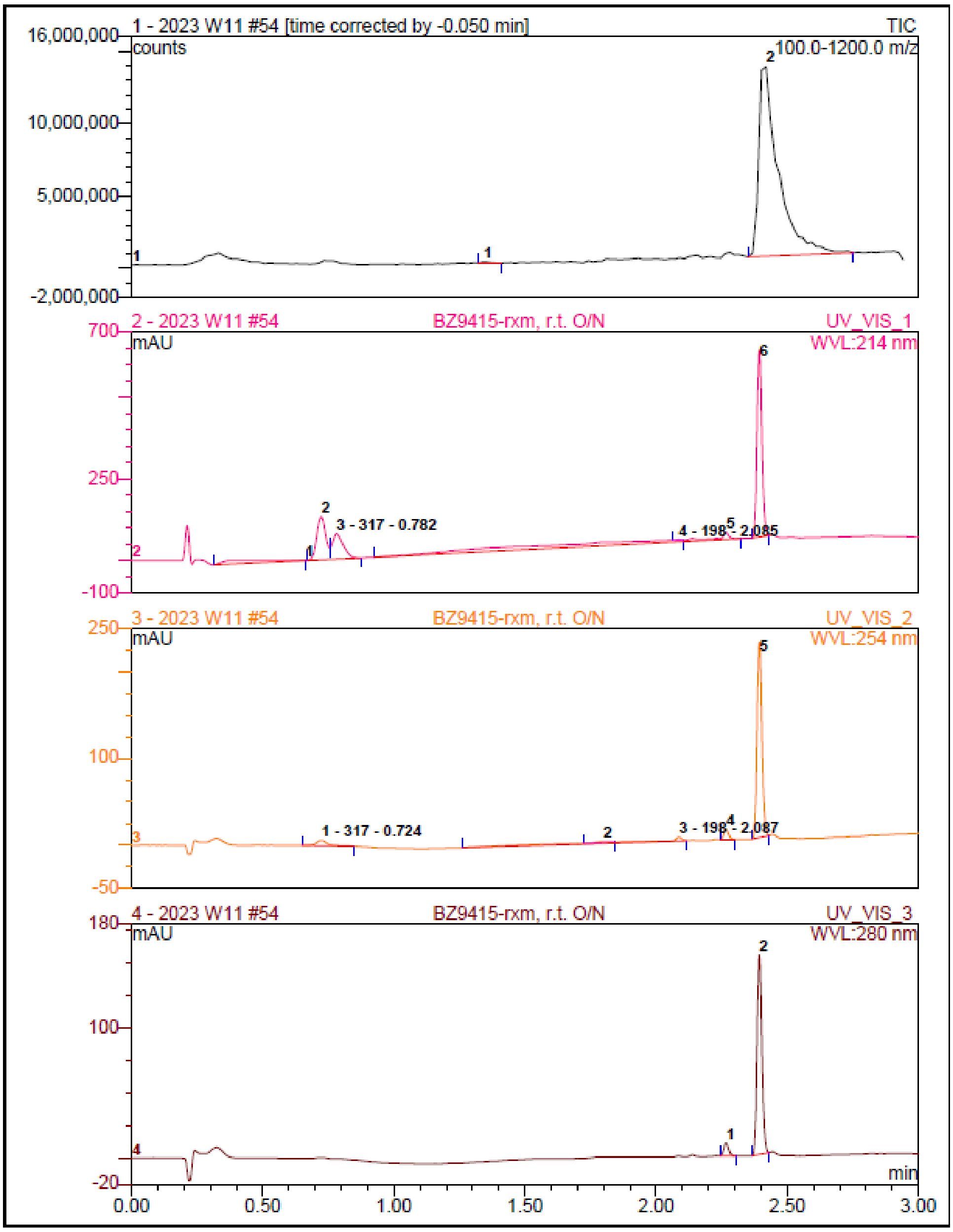

No.	Ret.Time min	Height mAU	Area mAU*min	Rel.Area %	Highest m/z responses Mass - 1 m/z	Mass -2 m/z	Mass - 3 m/z
1	0.72	6.036	0.306	5.02	466	998	457
2	1.80	0.840	0.055	0.90	660	561	669
3	2.09	4.501	0.640	10.51	605	408	497
4	2.27	12.528	0.263	4.32	406	784	789
5	2.39	224.677	4.829	79.25	370	392	761

Figure S2. LC-MS chromatogram for coupling of **XXI** with benzenesulfonamide in EtOAc using T_3P as coupling reagent (**Table S2**, **Entry 1**).

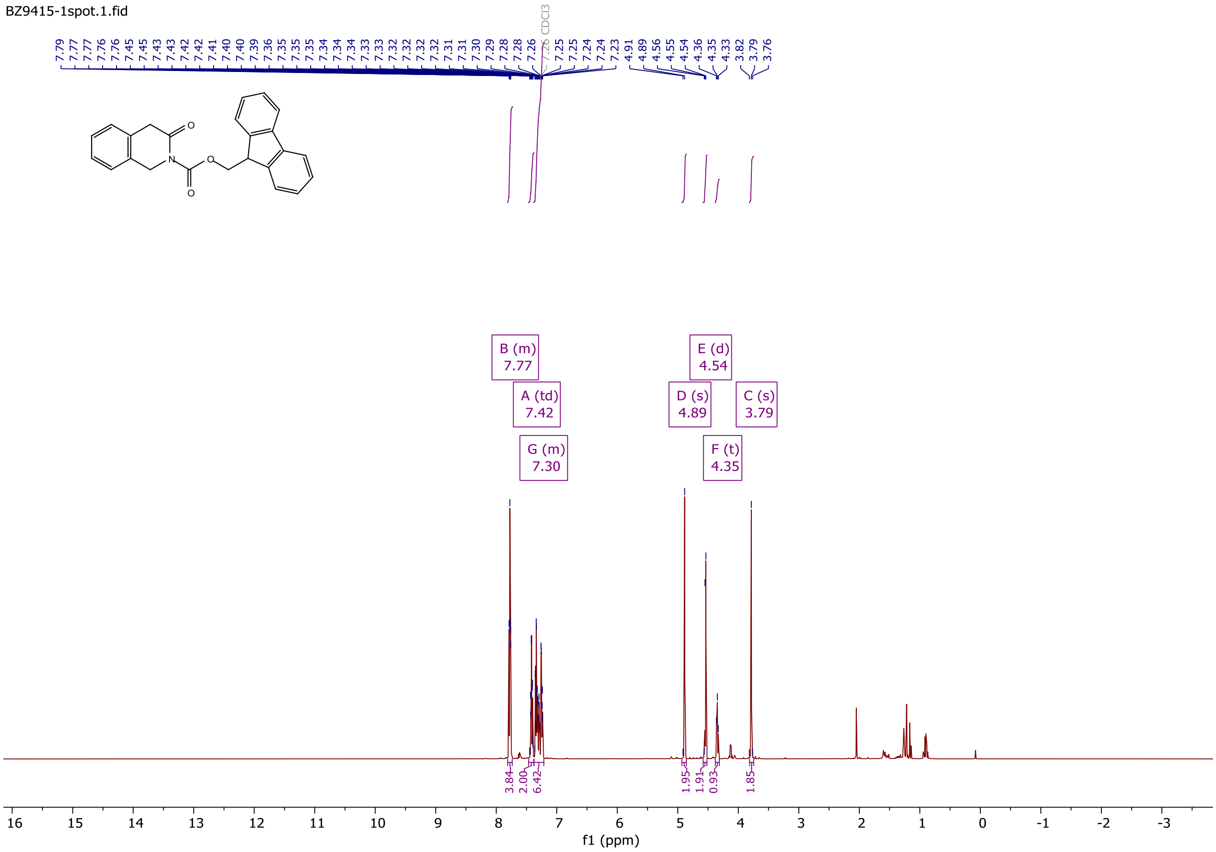

Figure S3. ^{1}H-NMR spectrum the isolated side product **S2b** formed by intramolecular cyclization (**Table S2, Entry 1**).

**Sample Name:**	BZ9418-rxm, 2h, r.t.	**Injection Volume:**	0.9 uL
**Control Program:**	5-100% positive	**UserID**	Lorenzo
**Recording Time:**	3/15/2023 16:33		

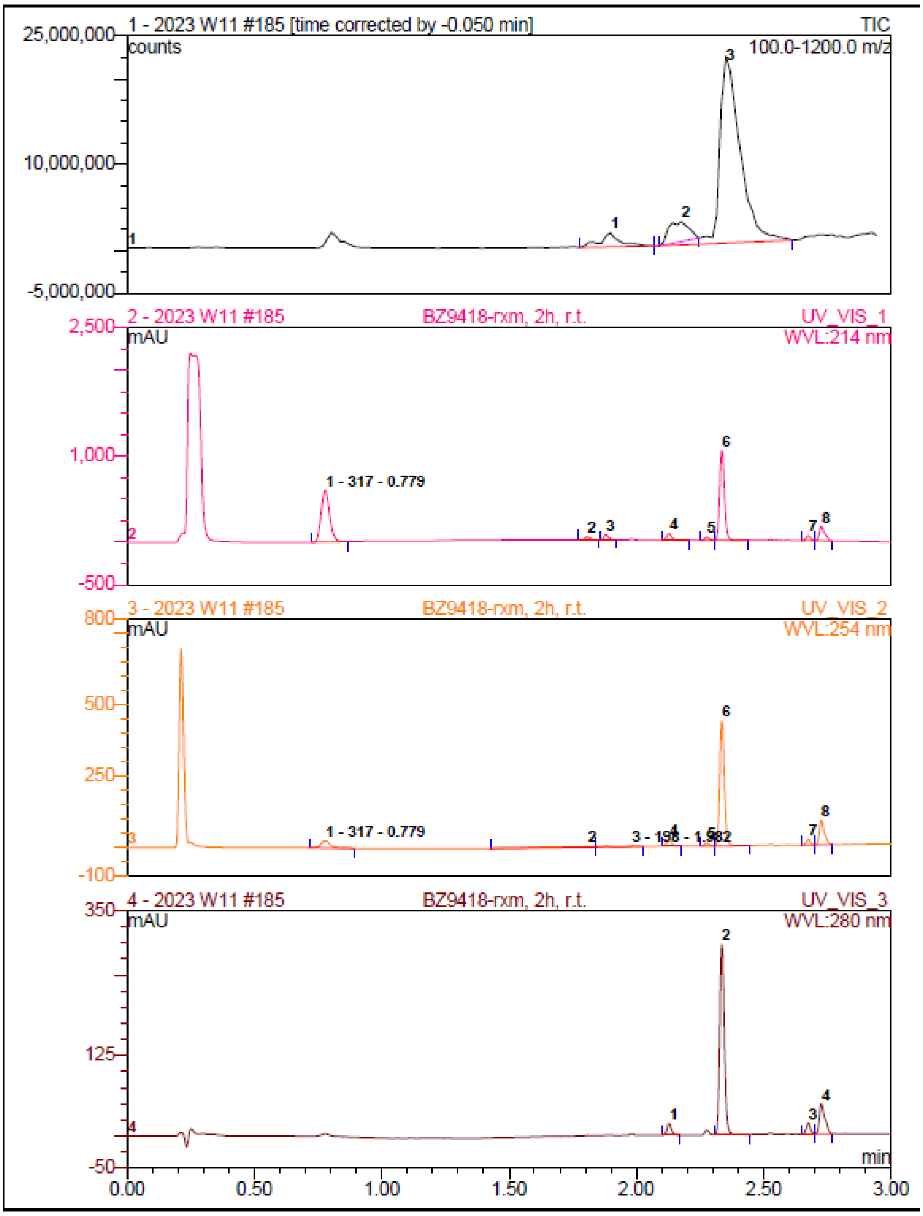

No.	Ret.Time min	Height mAU	Area mAU*min	Rel.Area %	Highest m/z responses Mass - 1 m/z	Mass -2 m/z	Mass - 3 m/z
1	0.78	26.058	1.042	7.60	451	409	335
2	1.81	2.402	0.229	1.67	321	537	335
3	1.98	3.382	0.206	1.50	409	387	322
4	2.13	26.156	0.529	3.86	415	416	437
5	2.28	11.125	0.220	1.60	527	528	305
6	2.34	438.267	9.107	66.40	370	233	179
7	2.68	20.963	0.405	2.95	571	729	330
8	2.73	87.113	1.977	14.41	728	729	628

Figure S4. LC-MS chromatogram for coupling of **XXI** with benzenesulfonamide in DMF using Boc anhydride as coupling reagent and with pyridine as base (**Table S2**, **Entry 2**). The chromatogram shows that the main species in the reaction mixture is **S2b** (RT 2.34 min, m/z 370).

**Sample Name:**	BZ9416-3setup before column	**Injection Volume:**	0.9 uL
**Control Program:**	5-100% positive	**UserID**	Lorenzo
**Recording Time:**	4/6/2023 10:12		

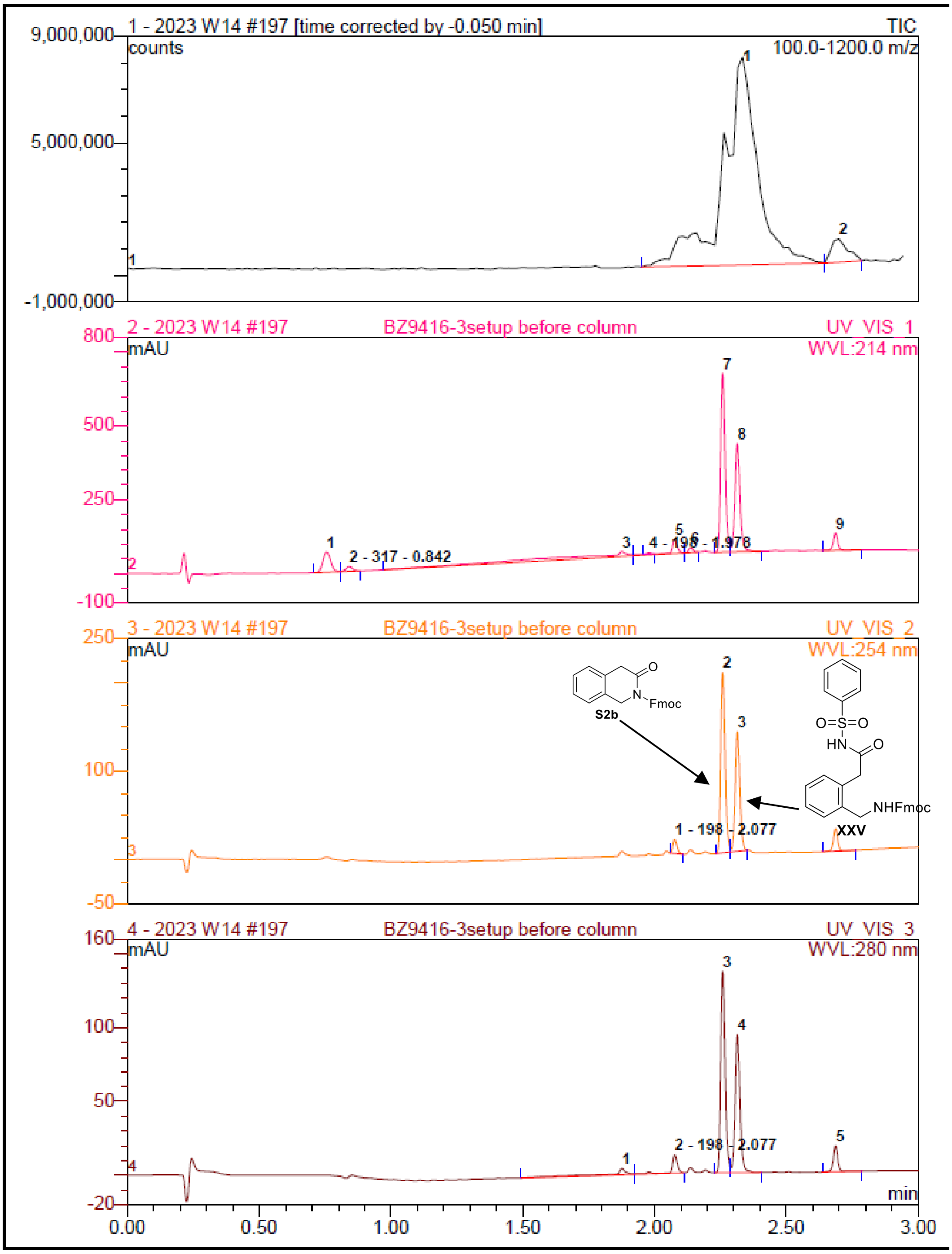

No.	Ret.Time min	Height mAU	Area mAU*min	Rel.Area %	Mass - 1 m/z	Mass -2 m/z	Mass - 3 m/z
					Highest m/z responses		
1	2.08	16.052	0.298	3.94	388	405	368
2	2.26	203.887	3.959	52.42	527	305	288
3	2.32	135.147	2.826	37.42	527	305	674
4	2.69	25.139	0.470	6.22	566	567	344

Figure S5. Using EDC*HCl for coupling of **XVII** with benzenesulfonamide (**Table S2, Entry 3**) resulted in formation of **S2b** and **XXV** in the ratio 3:2.

Characterization of macrocyclic tripeptide 4 (LC-MS and ^{1}H NMR)

Compound 4, LC-MS

Name: (4*R*,8*S*,11*S*)-11-((((9*H*-fluoren-9-yl)methoxy)carbonyl)amino)-8-(4-hydroxybenzyl)-6,10-dioxo-1,2-dithia-5,9-diazacy-clotridecane-4-carboxylic acid

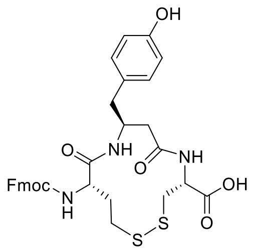

A)

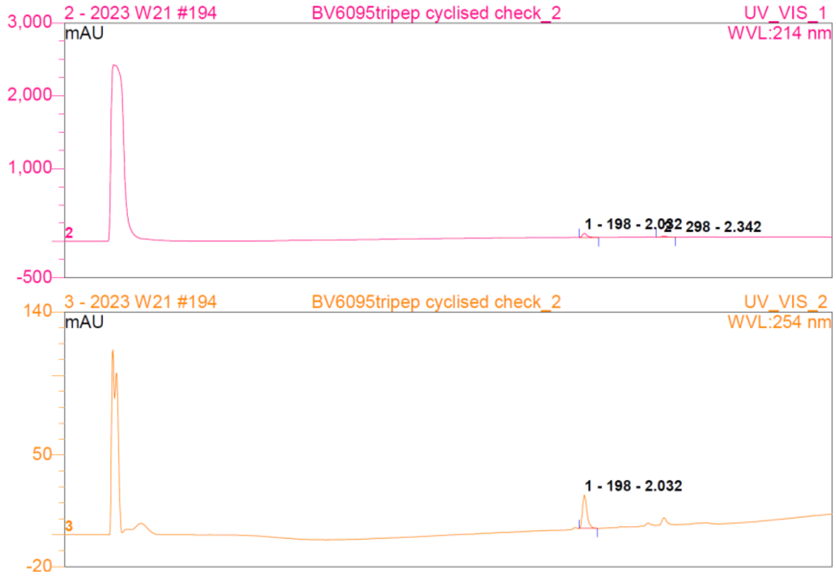

B)

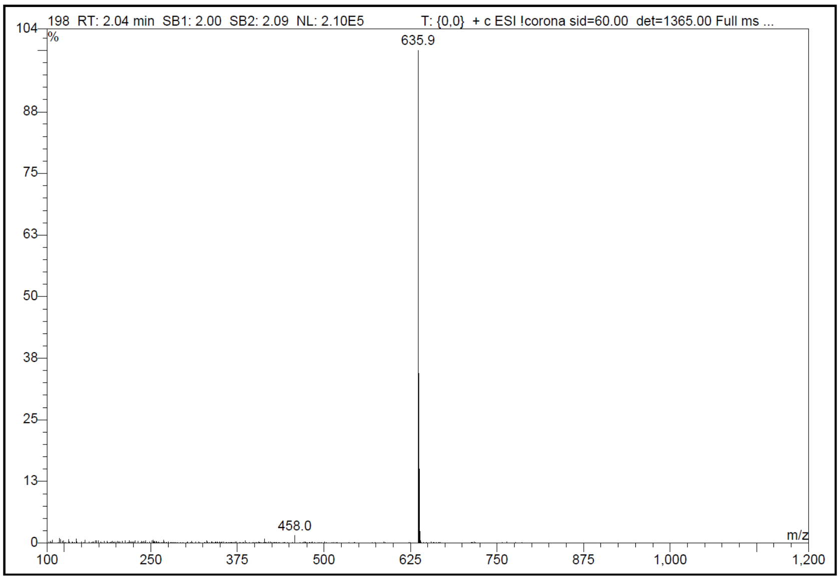

A) Analytical RP-HPLC of compound **4** on C18 column, acetonitrile gradient 5-100% of 0.05% formic acid in acetonitrile/water for 3 min. Top to bottom: UV detection at 214 and 254 nm. B) Low-resolution mass spectrum of **4**.

Compound 4, ^{1}H NMR (400 MHz, DMSO-d_6) spectrum

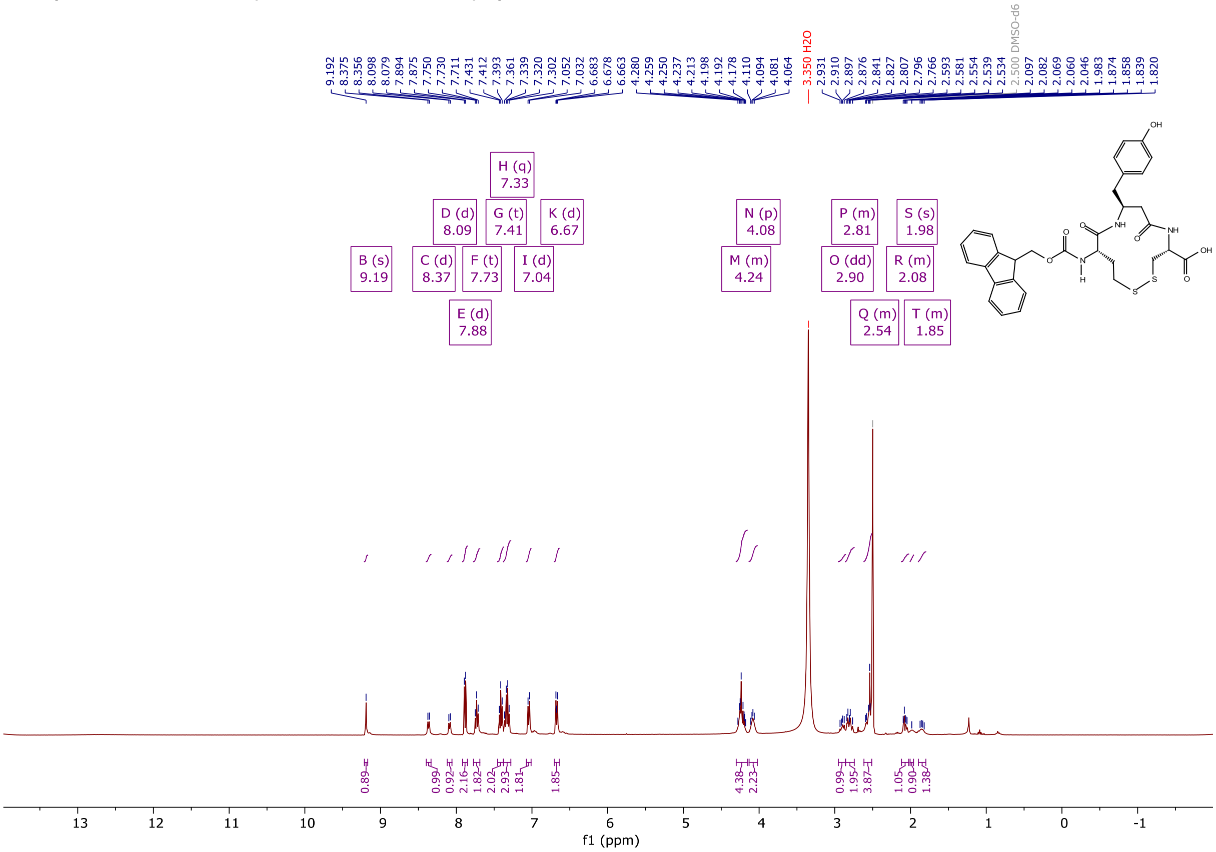

Characterization of Non-Natural Amino Acids 5, 9, 18-19, XXII-XXV and their building blocks II-IV, VI-VIII, XII-XVII and XXI (LC-MS, ^{1}H and ^{13}C NMR)

Compound S4, ^{1}H NMR (400 MHz, $CDCl_3$) spectrum

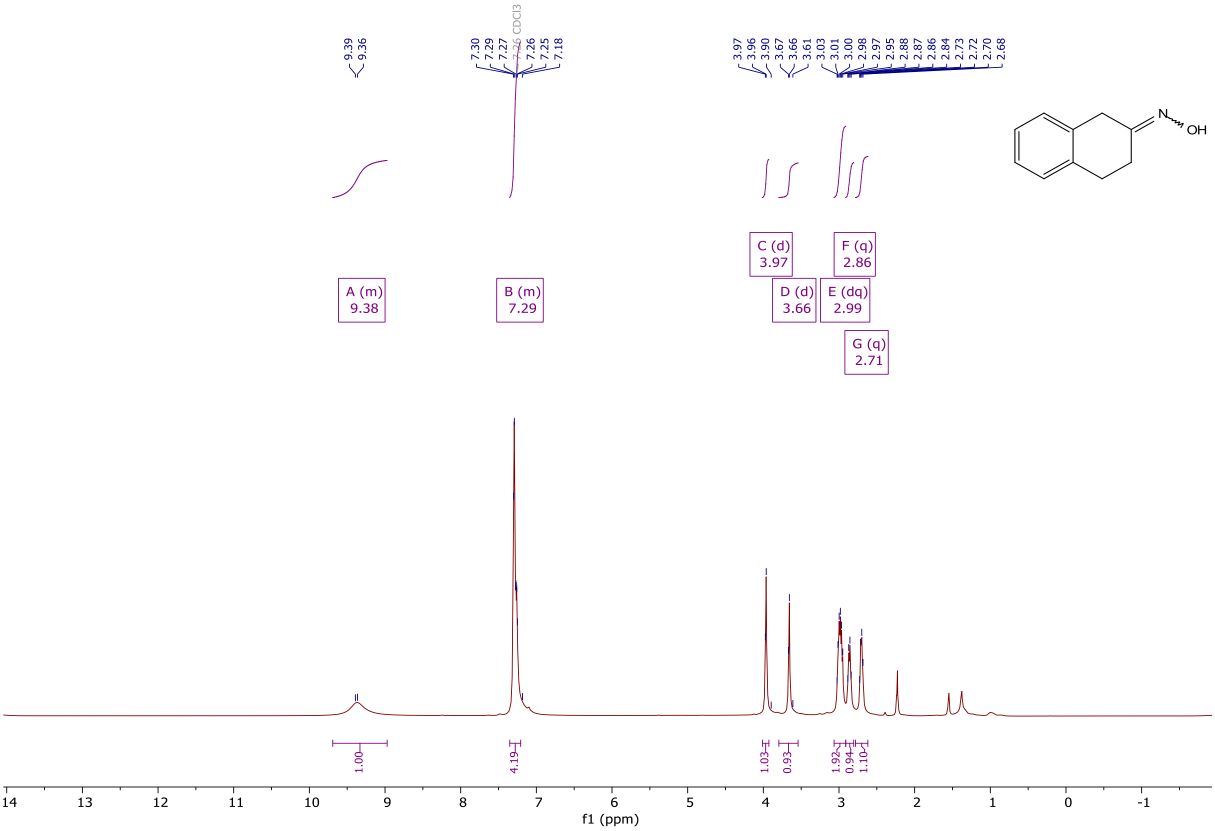

Compound S4, ^{13}C{1H} NMR (101 MHz, $CDCl_3$) spectrum

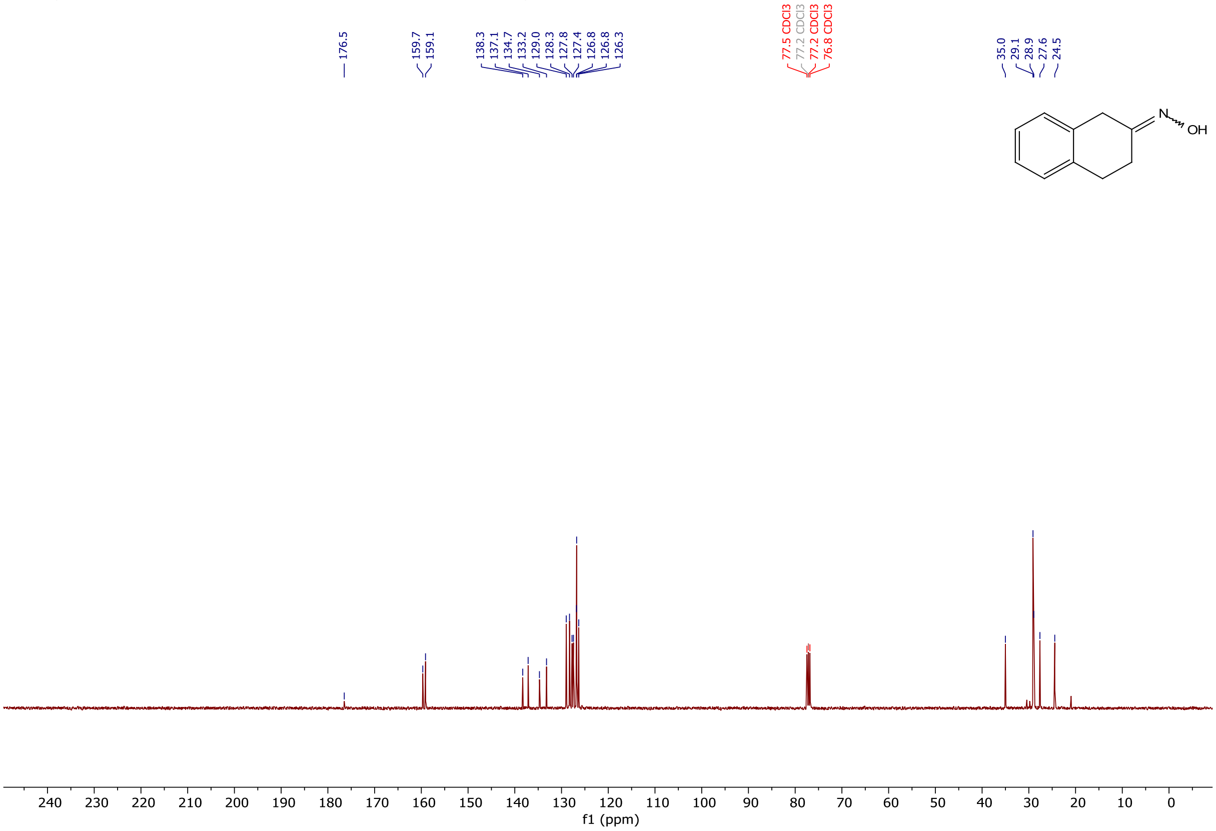

Compound II, LC-MS

A)

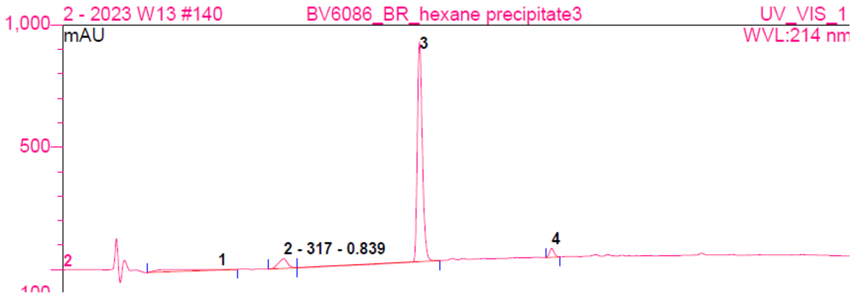

B)

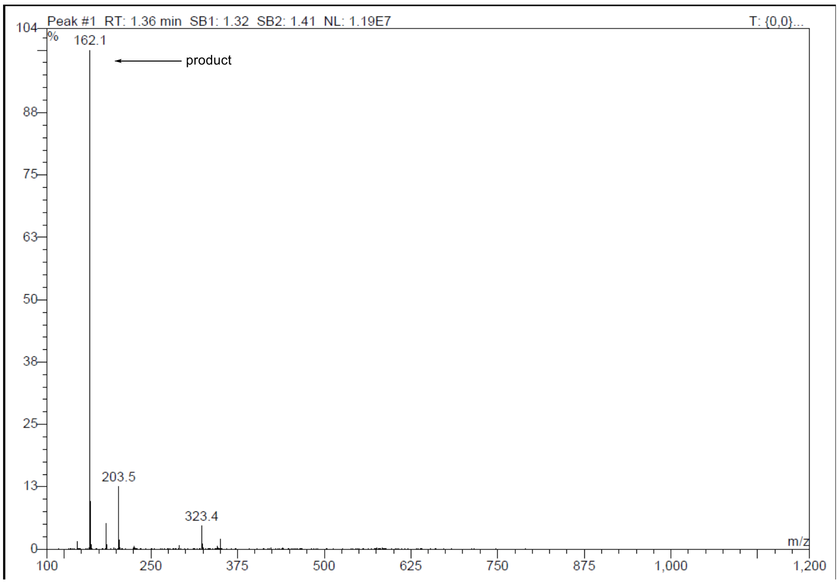

A) Analytical RP-HPLC of compound **II** on C18 column, acetonitrile gradient 5-100% of 0.05% formic acid in acetonitrile/water for 3 min. UV detection at 214 nm. B) Low-resolution mass spectrum of **II**.

Compound II, ^{1}H NMR (400 MHz, $CDCl_3$) spectrum

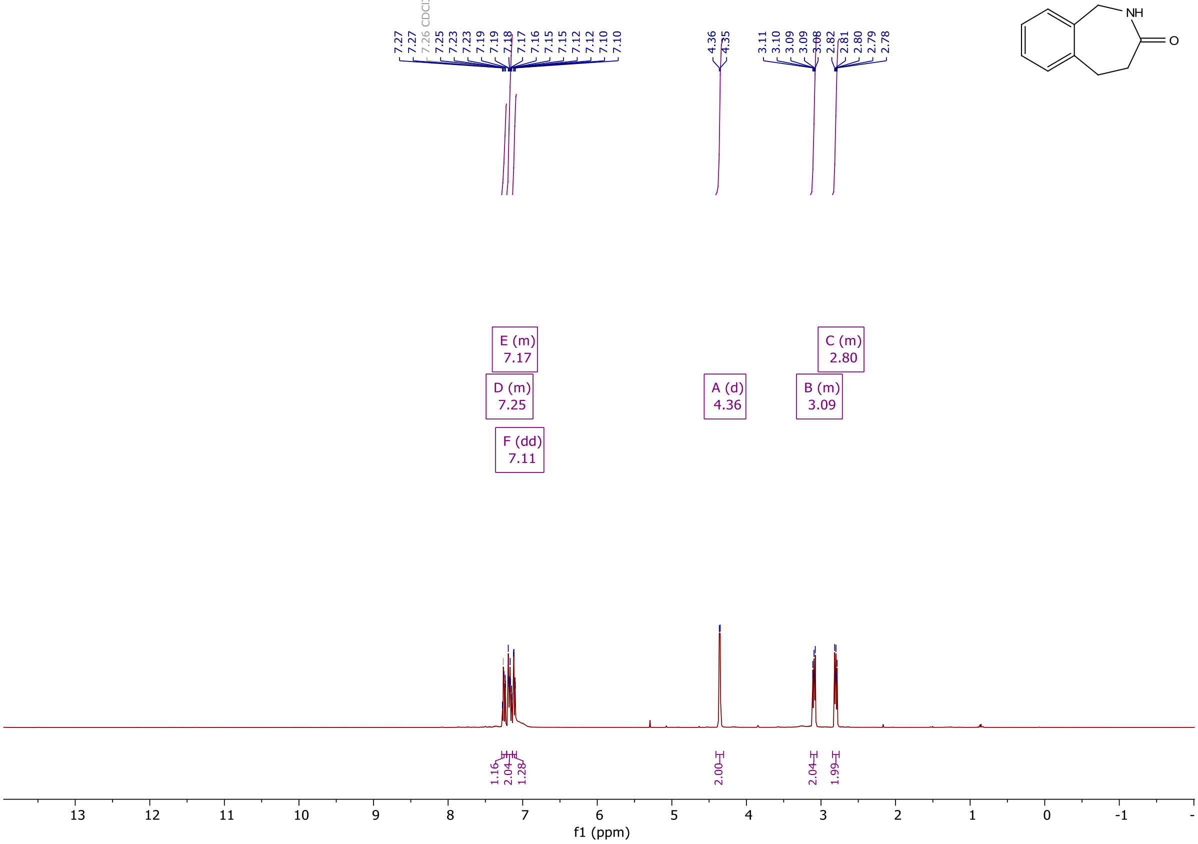

Compound II, ^{13}C{1H} NMR (101 MHz, $CDCl_3$) spectrum

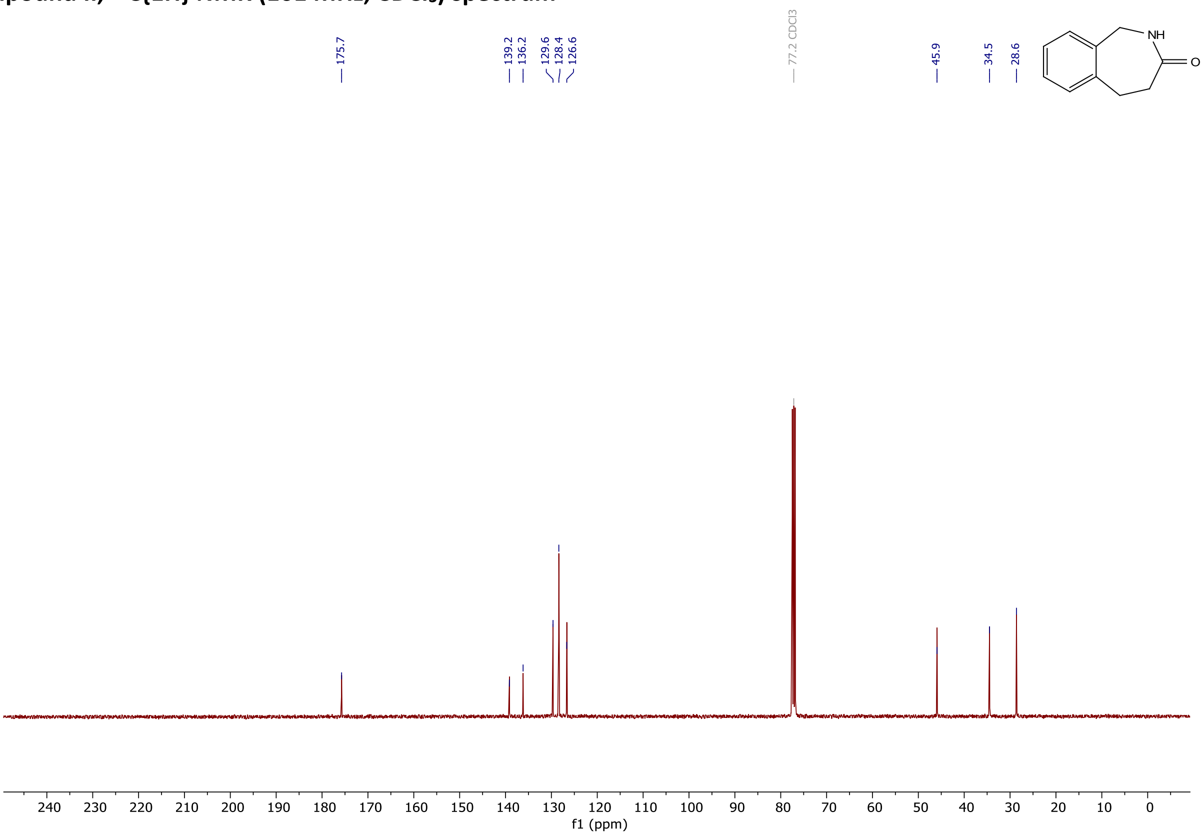

Compound III, ^{1}H NMR (400 MHz, $CDCl_3$) spectrum

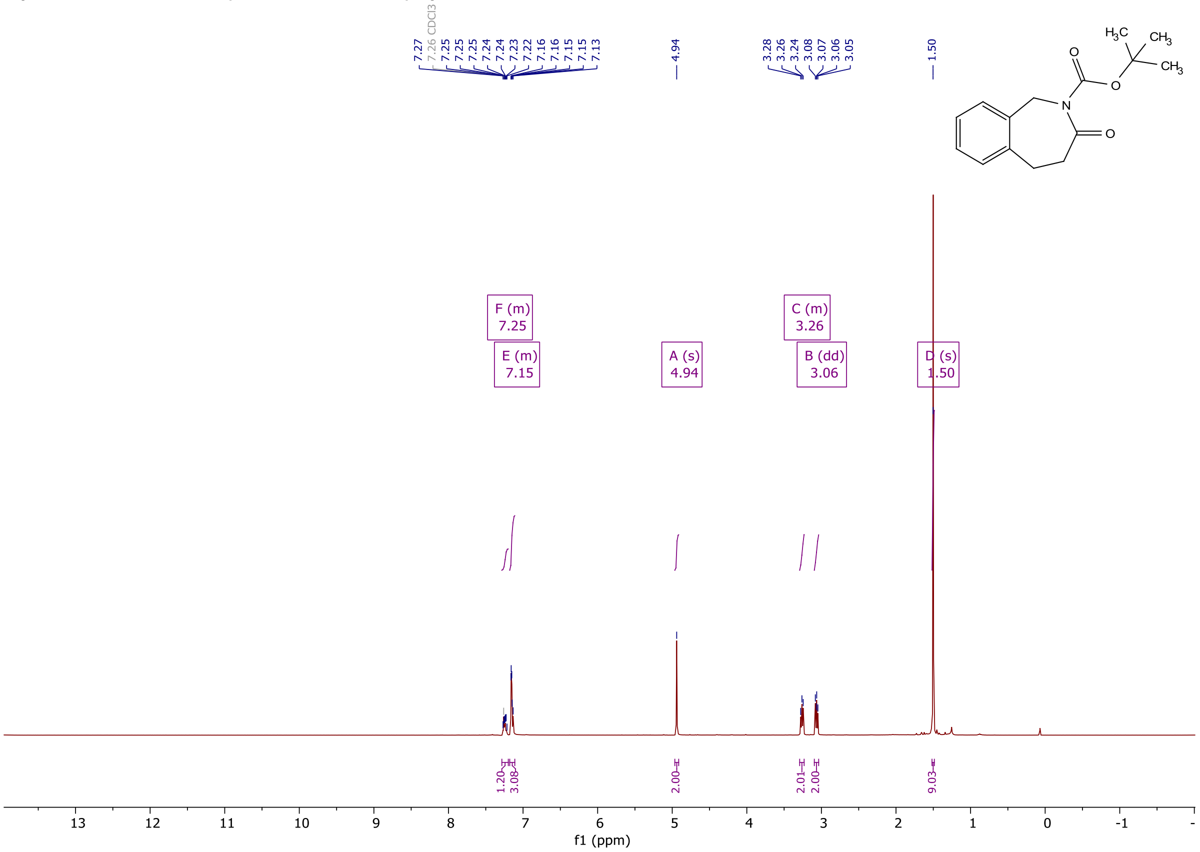

Compound III, $^{13}C\{1H\}$ NMR (101 MHz, $CDCl_3$) spectrum

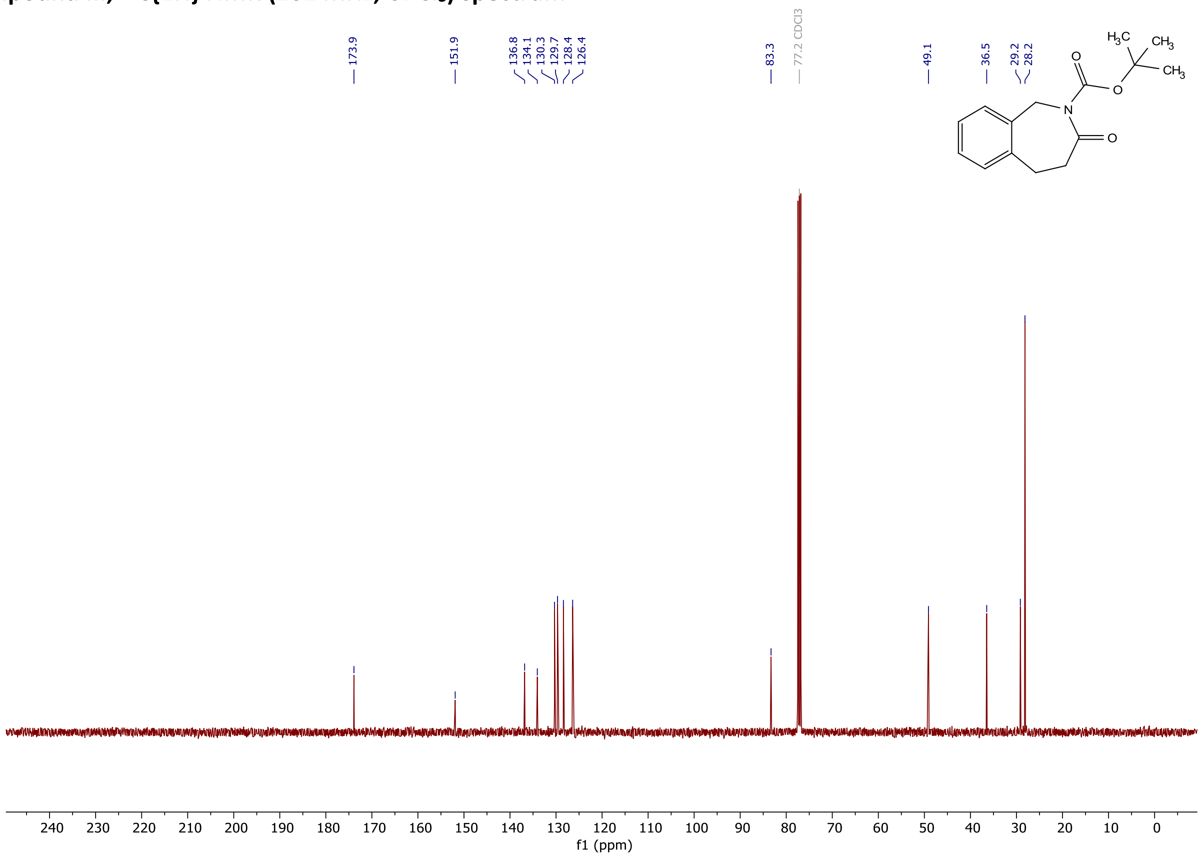

Compound IV, ^{1}H NMR (400 MHz, $CDCl_3$) spectrum

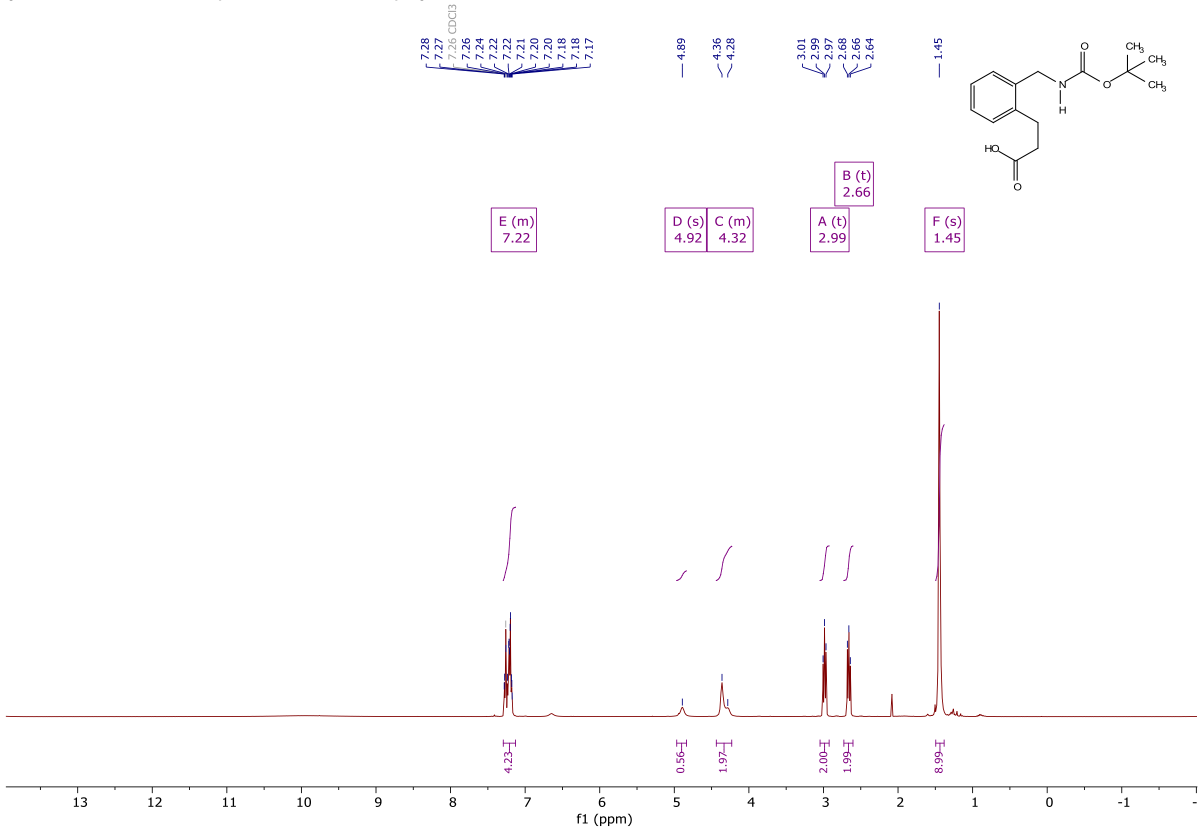

Compound IV, ^{13}C{1H} NMR (101 MHz, $CDCl_3$) spectrum

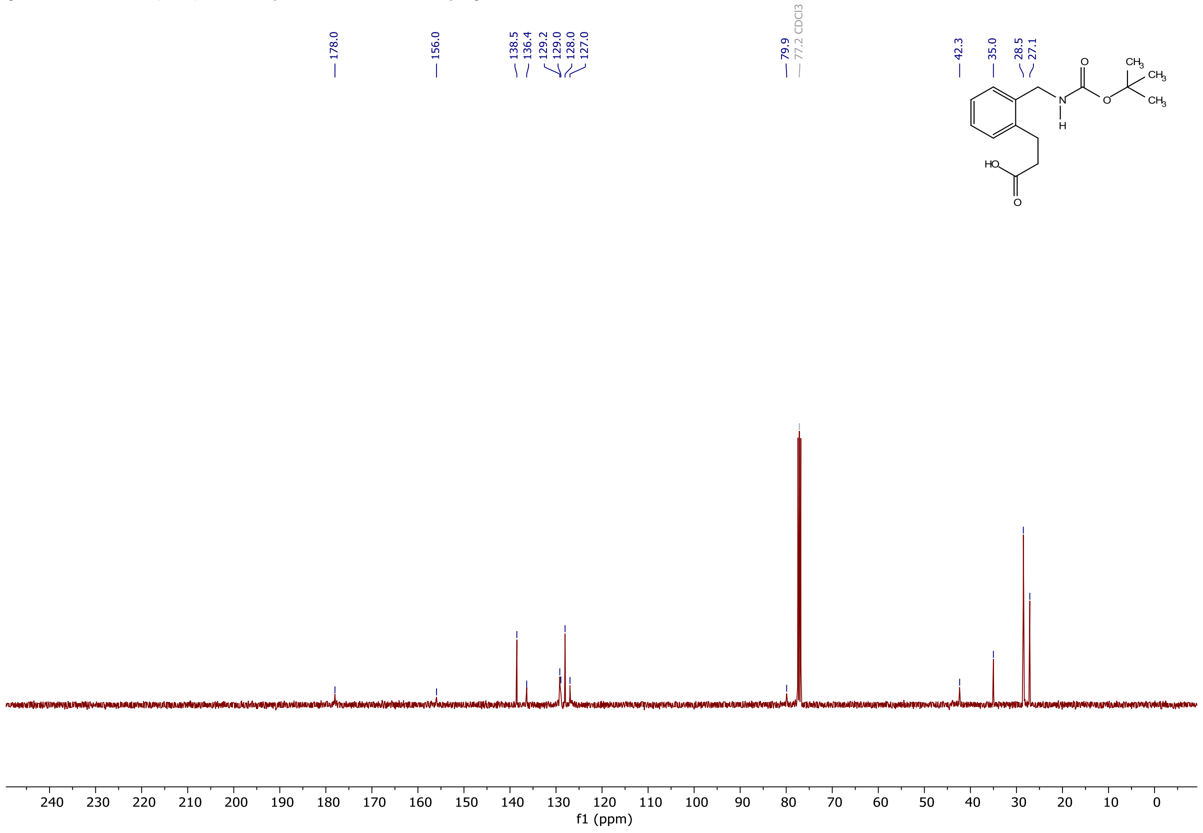

Compound 5, LC-MS

A)

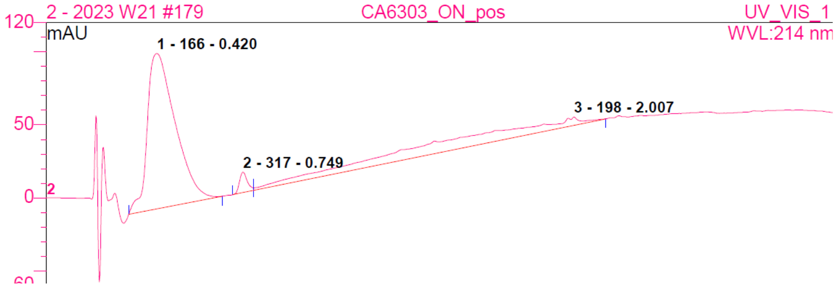

B)

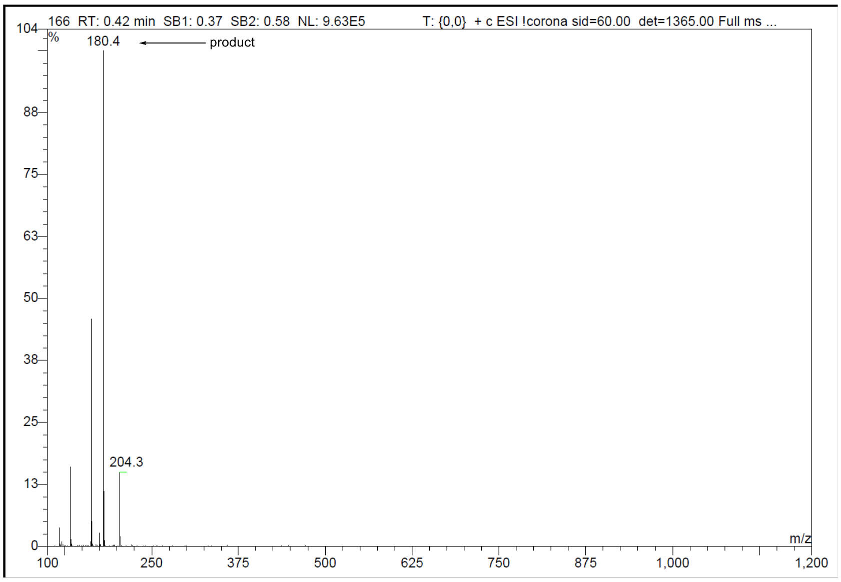

A) Analytical RP-HPLC of compound **5** on C18 column, acetonitrile gradient 5-100% of 0.05% formic acid in acetonitrile/water for 3 min. UV detection at 214 nm. B) Low-resolution mass spectrum of **5**.

Compound 5, ^{1}H NMR (400 MHz, MeOD) spectrum

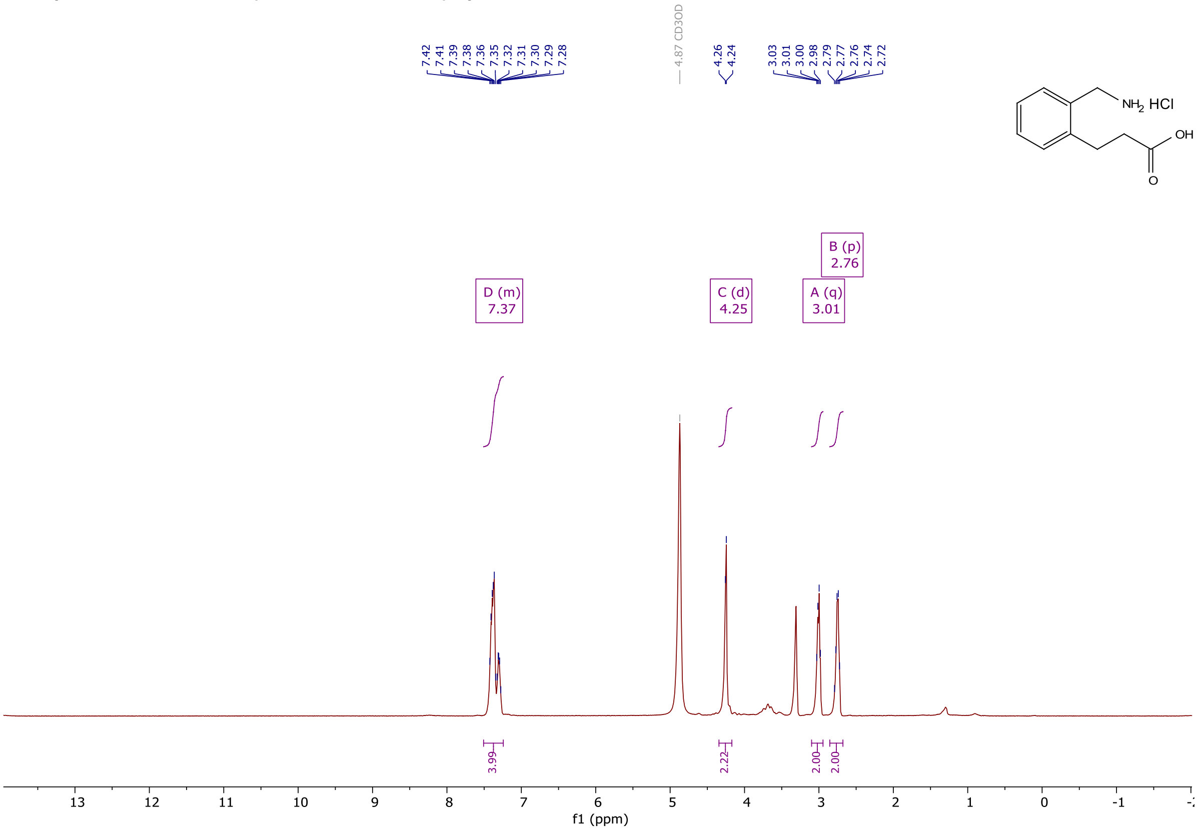

Compound 5, ^{13}C{1H} NMR (101 MHz, MeOD) spectrum

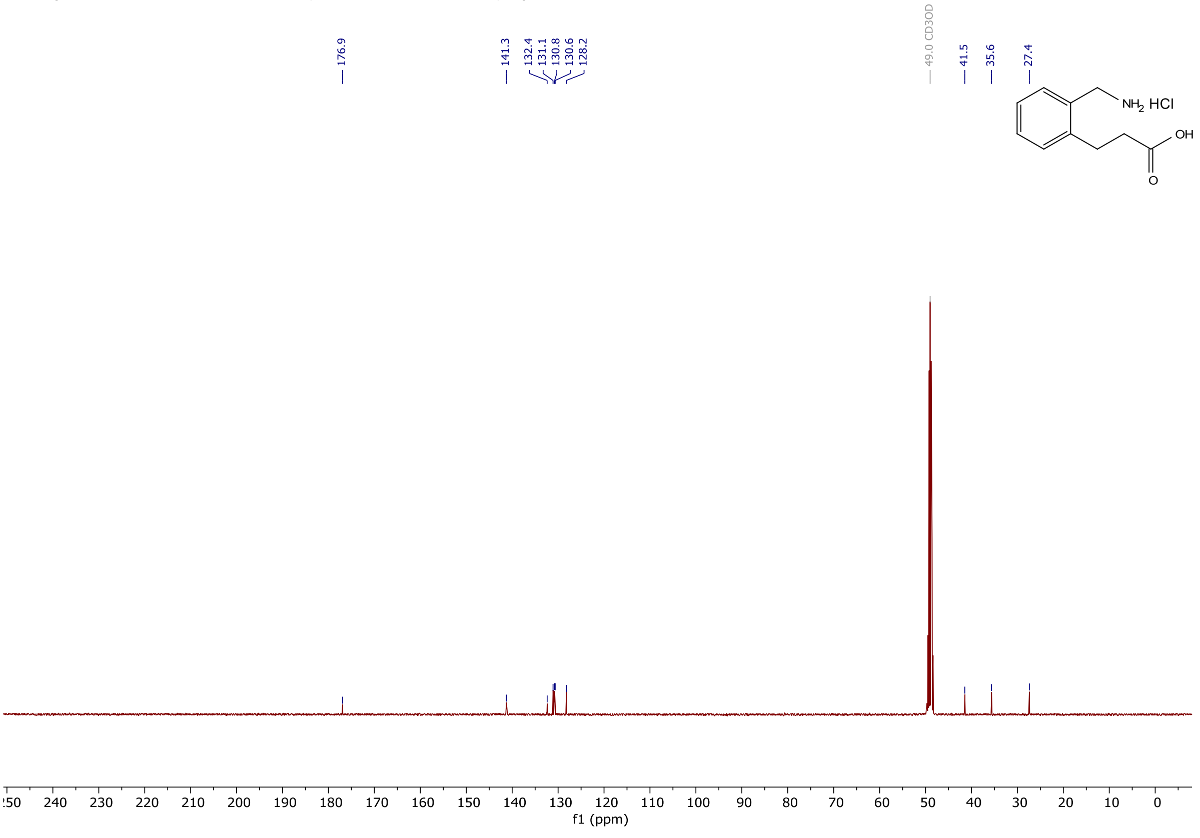

Compound VI, ^{1}H NMR (400 MHz, $CDCl_3$) spectrum

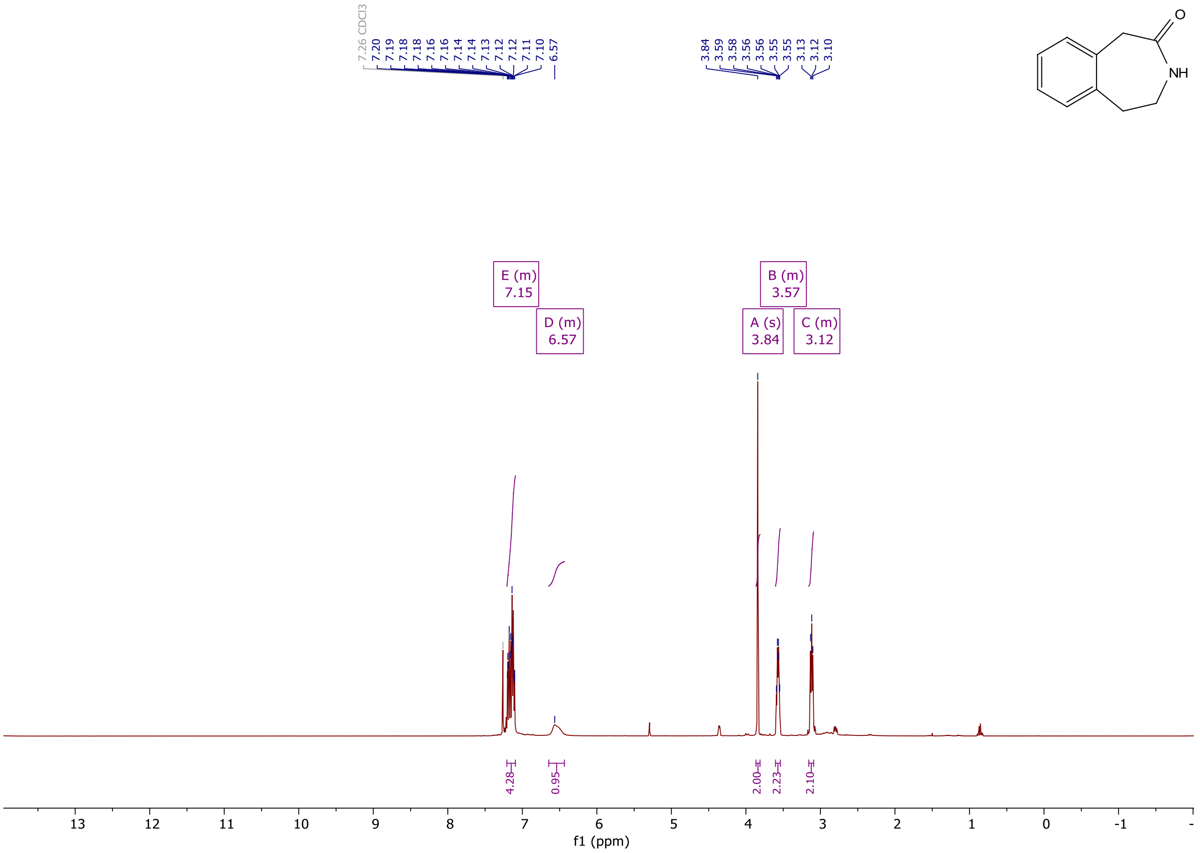

Compound VI, $^{13}C\{1H\}$ NMR (101 MHz, $CDCl_3$) spectrum

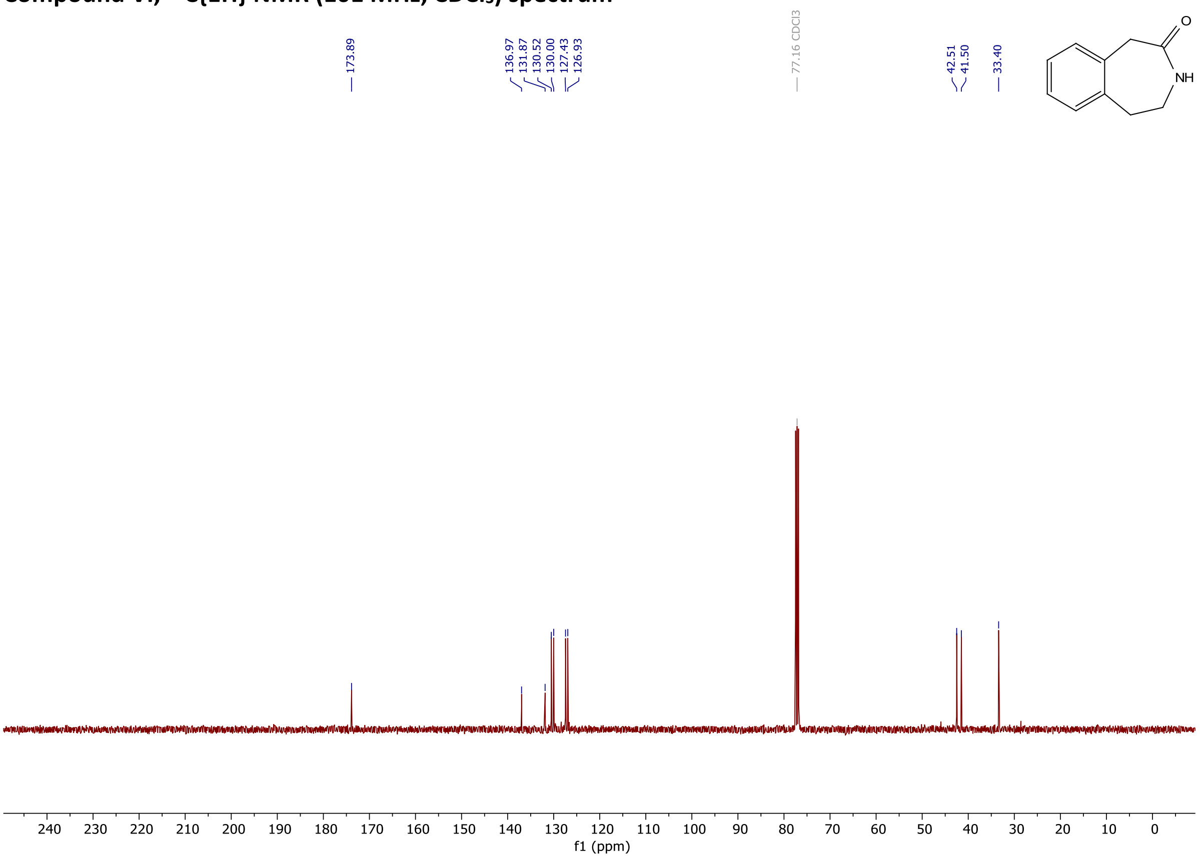

Compound VII, LC-MS

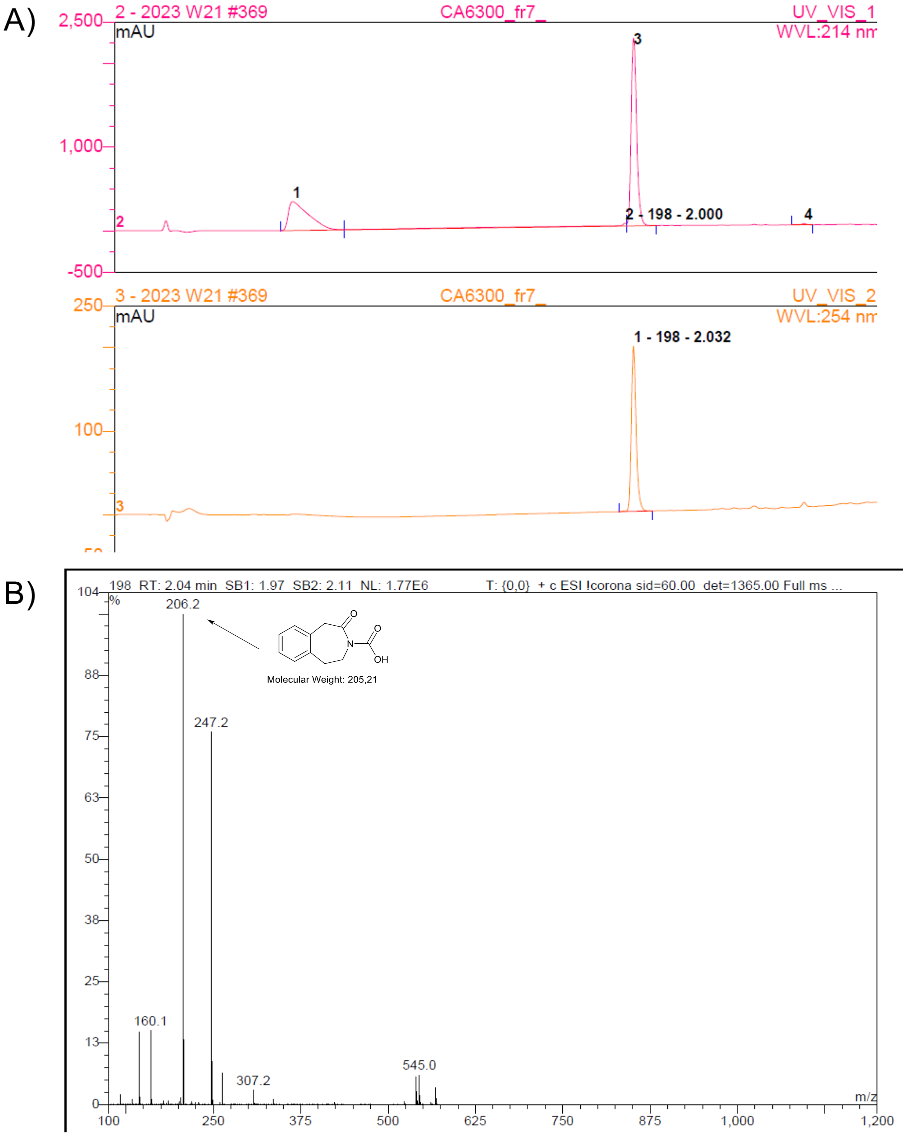

A) Analytical RP-HPLC of compound **VII** on C18 column, acetonitrile gradient 5-100% of 0.05% formic acid in acetonitrile/water for 3 min. Top to bottom: UV detection at 214 and 254 nm. B) Low-resolution mass spectrum of **VII**, fragment with *t*Bu loss detected.

Compound VII, ^{1}H NMR (400 MHz, $CDCl_3$) spectrum

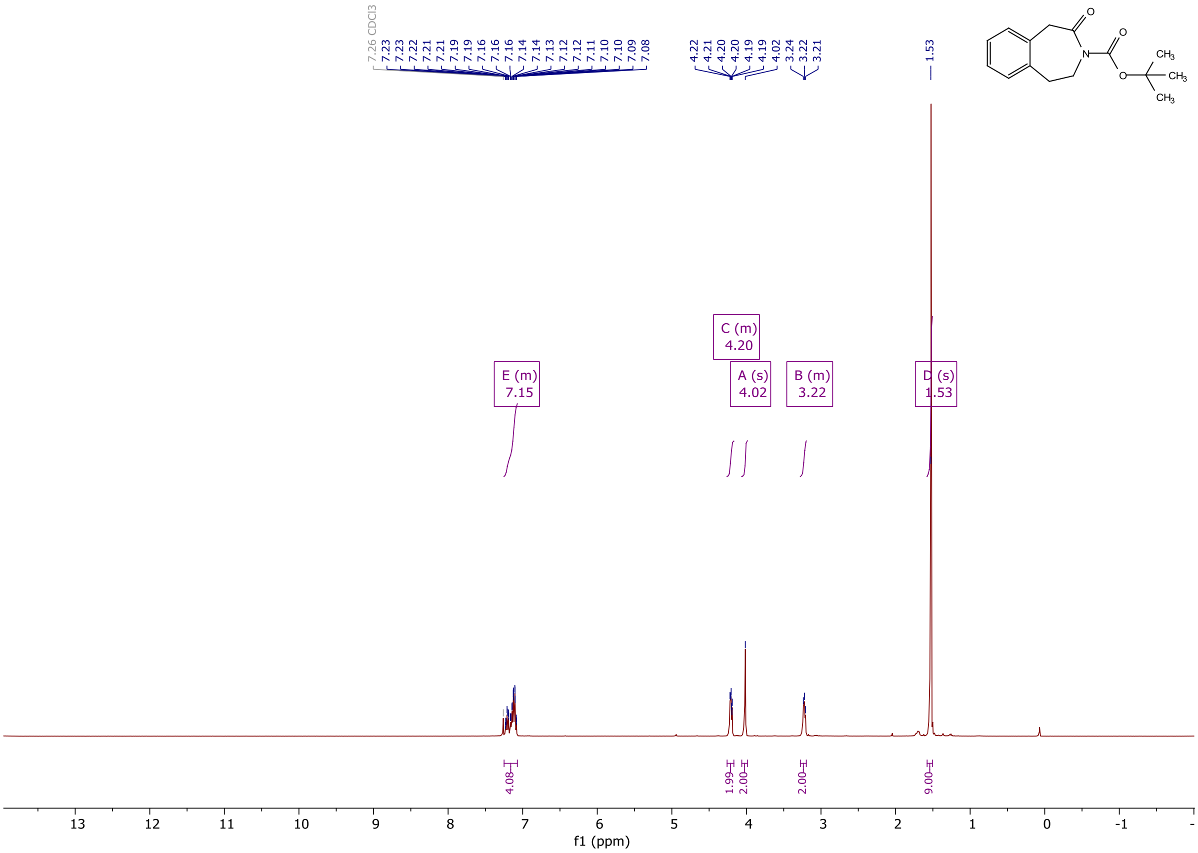

Compound VII, ^{13}C{1H} NMR (101 MHz, $CDCl_3$) spectrum

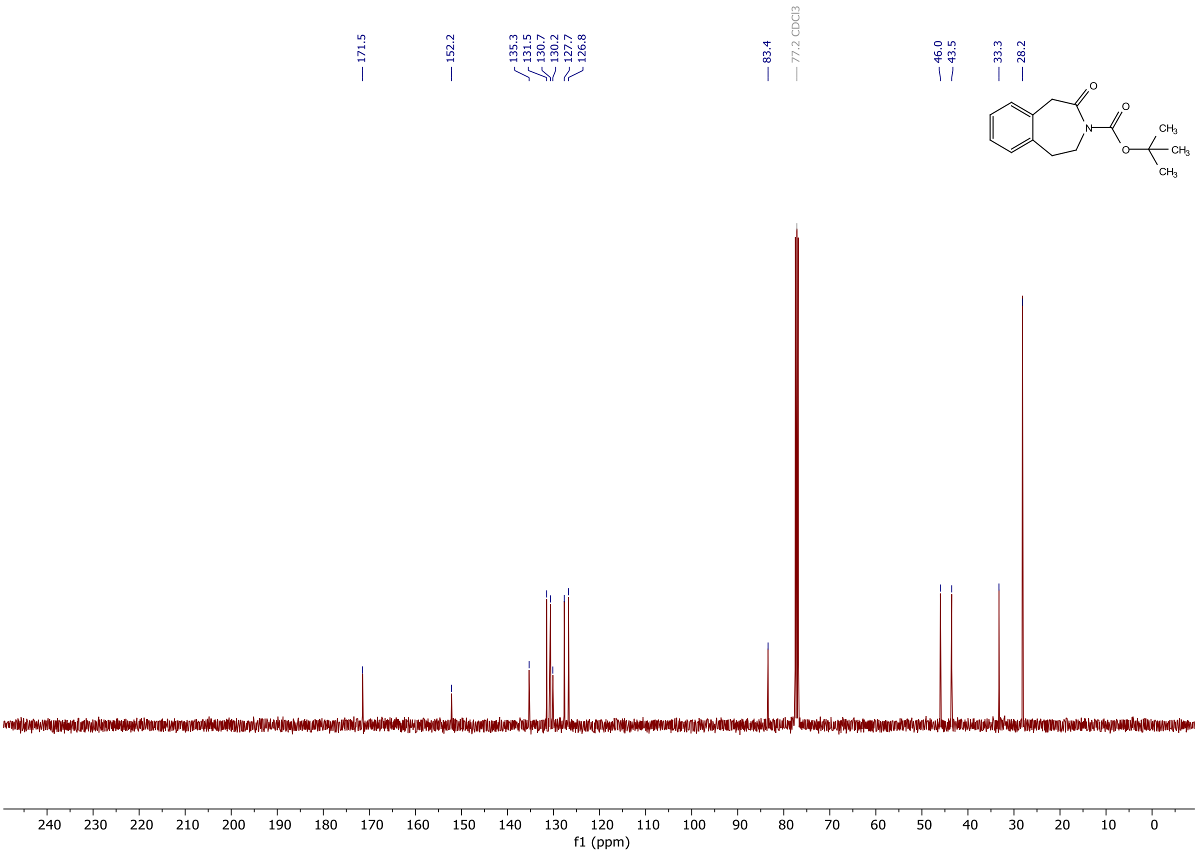

Compound VIII, LC-MS

A)

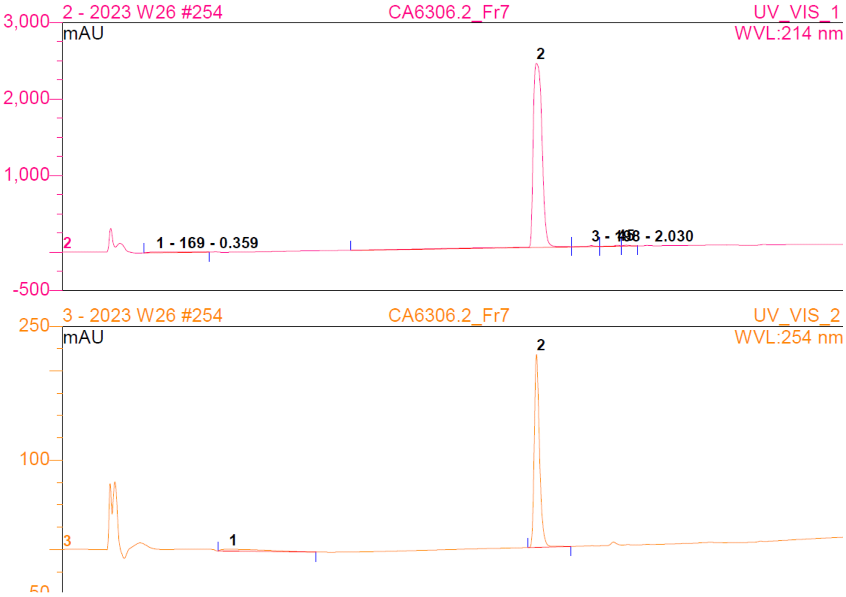

B)

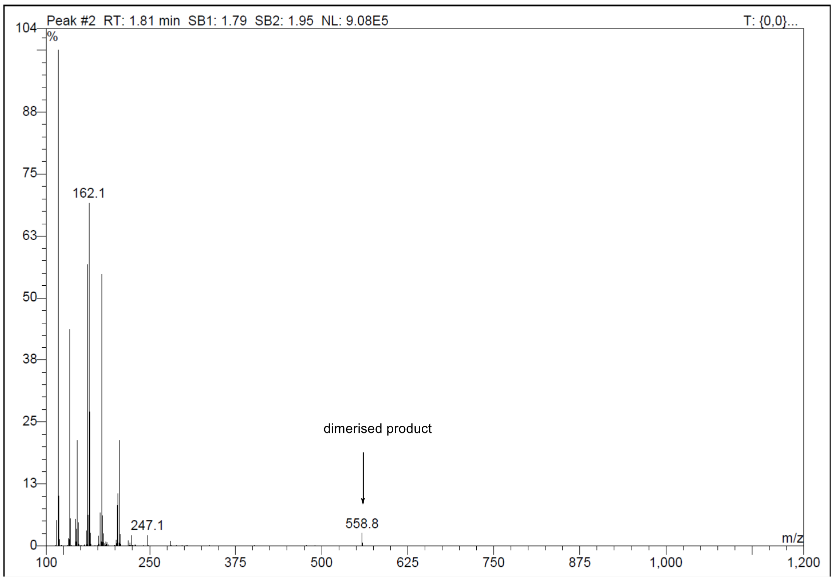

A) Analytical RP-HPLC of compound **VIII** on C18 column, acetonitrile gradient 5-100% of 0.05% formic acid in acetonitrile/water for 3 min. Top to bottom: UV detection at 214 and 254 nm. B) Low-resolution mass spectrum of **VIII**, dimerized product detected.

Compound VIII, ^{1}H NMR (400 MHz, $CDCl_3$) spectrum

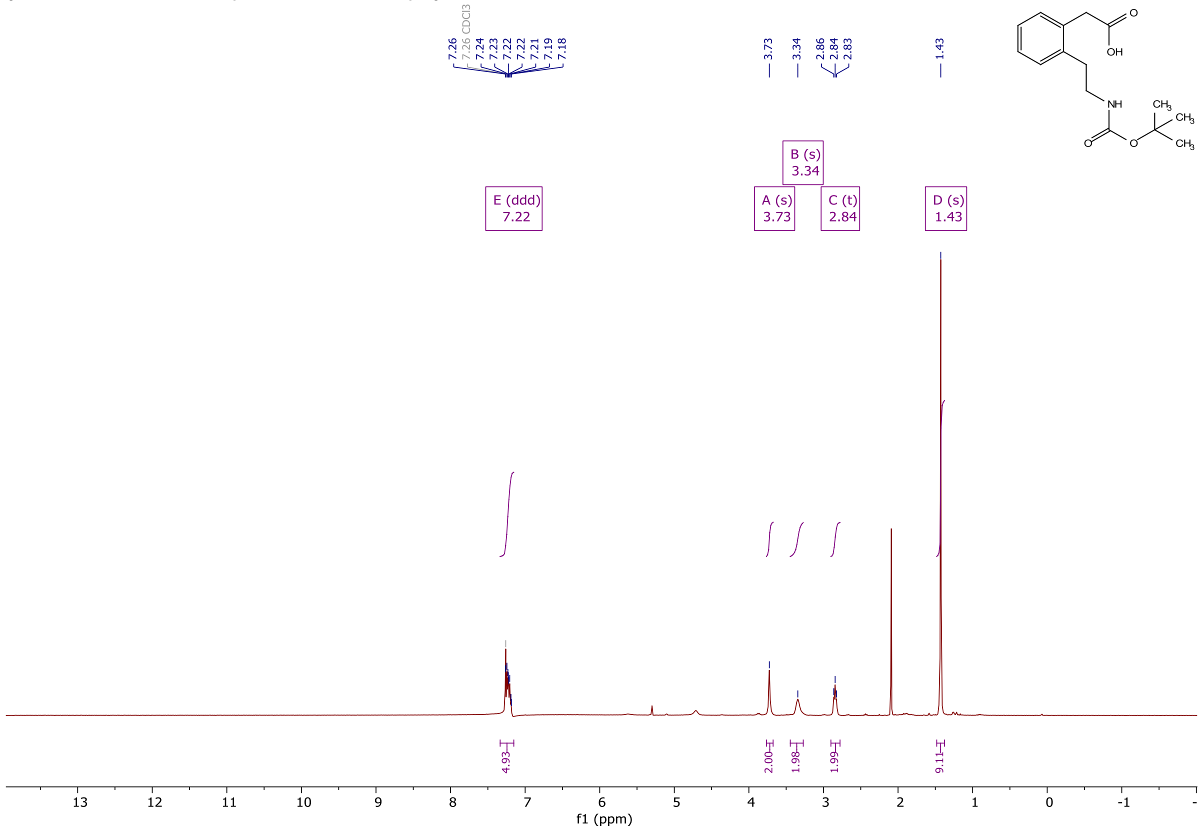

Compound VIII, $^{13}C\{1H\}$ NMR (126 MHz, $CDCl_3$) spectrum

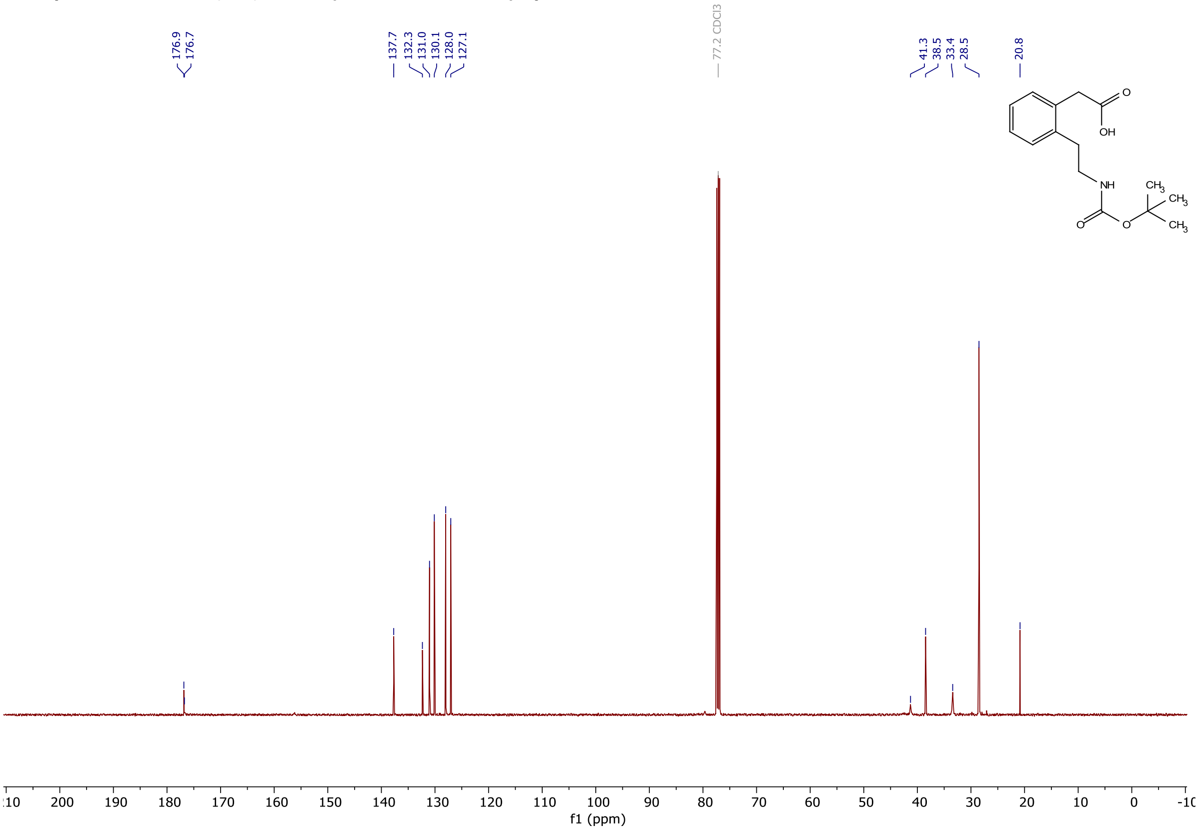

Compound 9, LC-MS

A)

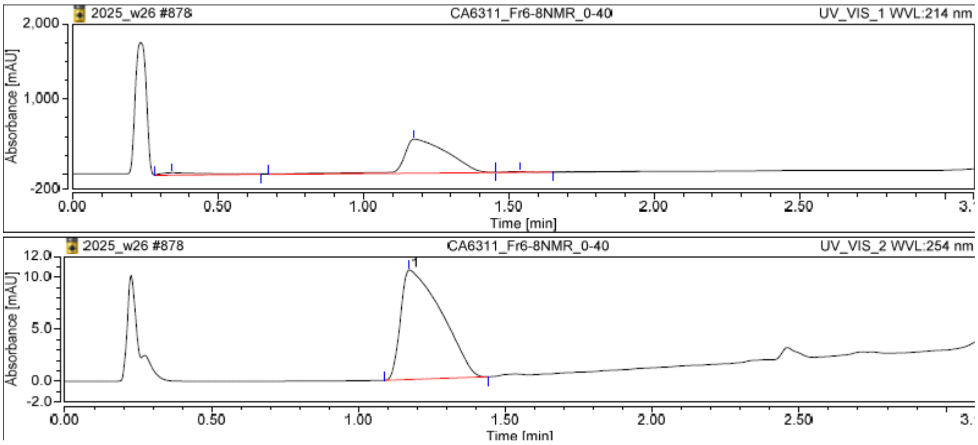

B)

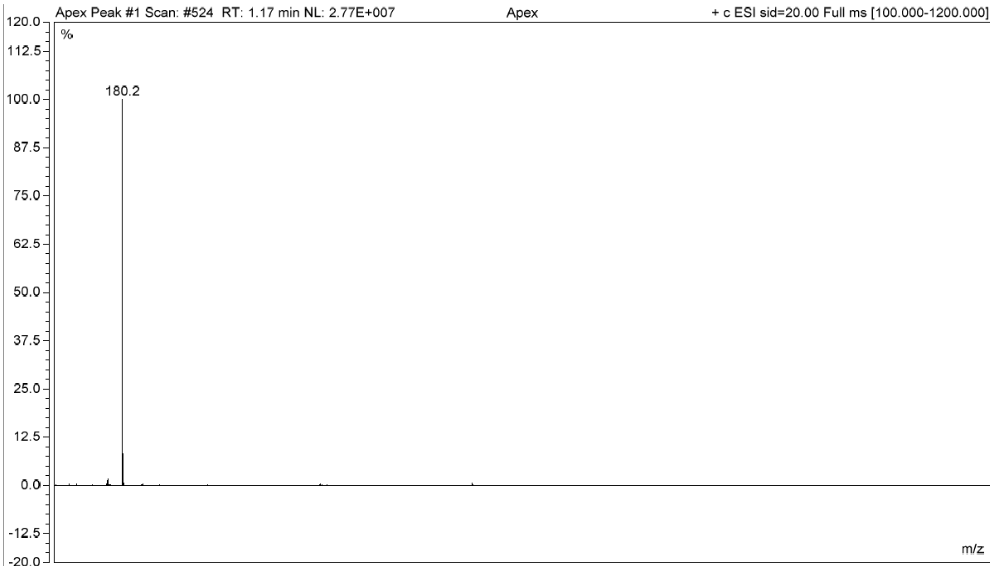

A) Analytical RP-HPLC of compound **9** on C18 column, acetonitrile gradient 0-40% of 0.05% formic acid in acetonitrile/water for 3 min. UV detection at 214 nm. B) Low-resolution mass spectrum of **9**.

Compound 9, ^{1}H NMR (400 MHz, DMSO-d_6) spectrum

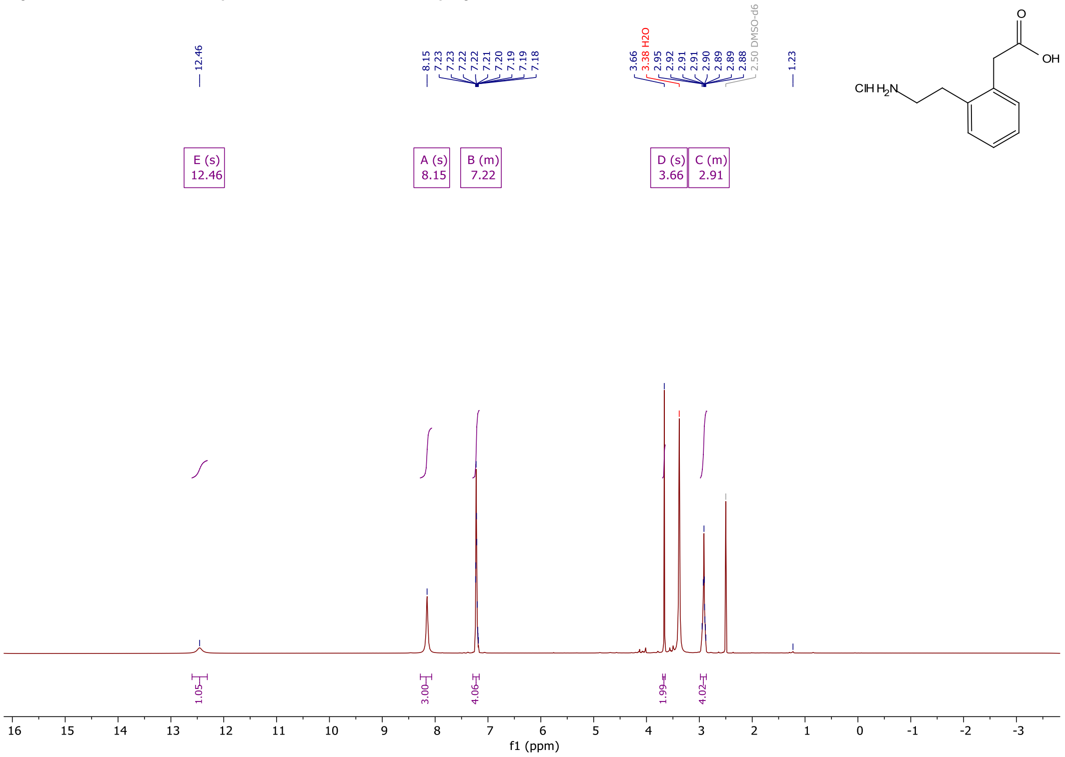

Compound 9, ^{13}C{1H} NMR (126 MHz, DMSO-d_6) spectrum

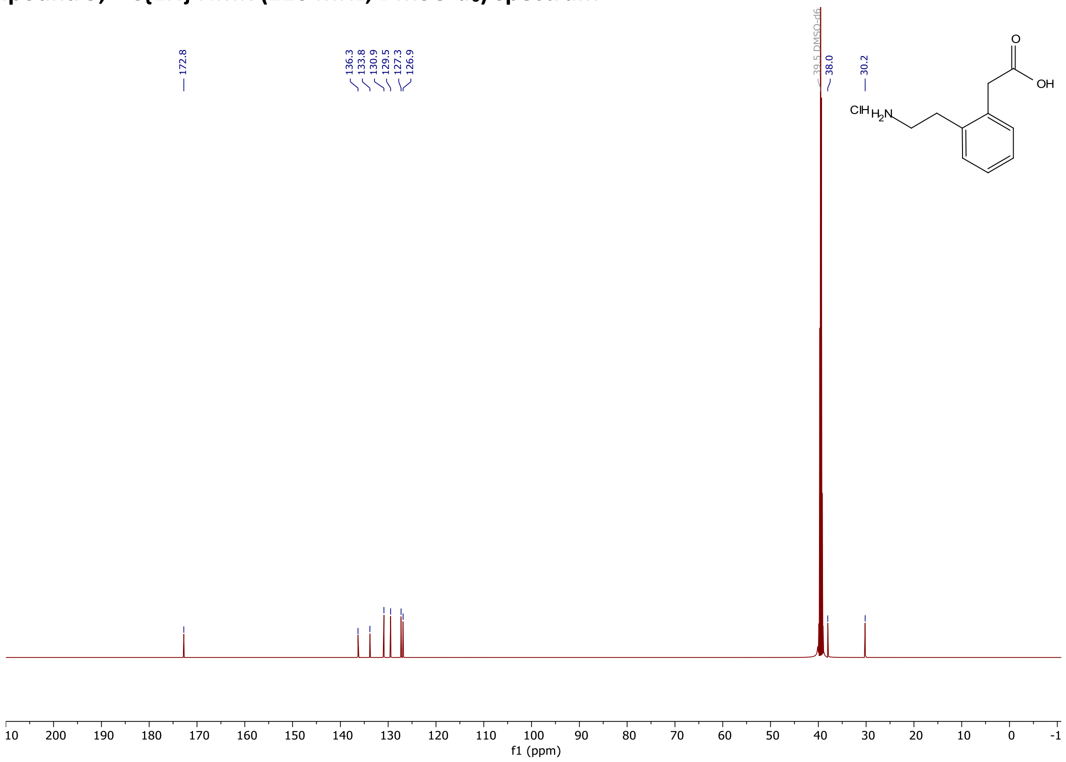

Compound XII, ^{1}H NMR (400 MHz, CDCl$_3$) spectrum

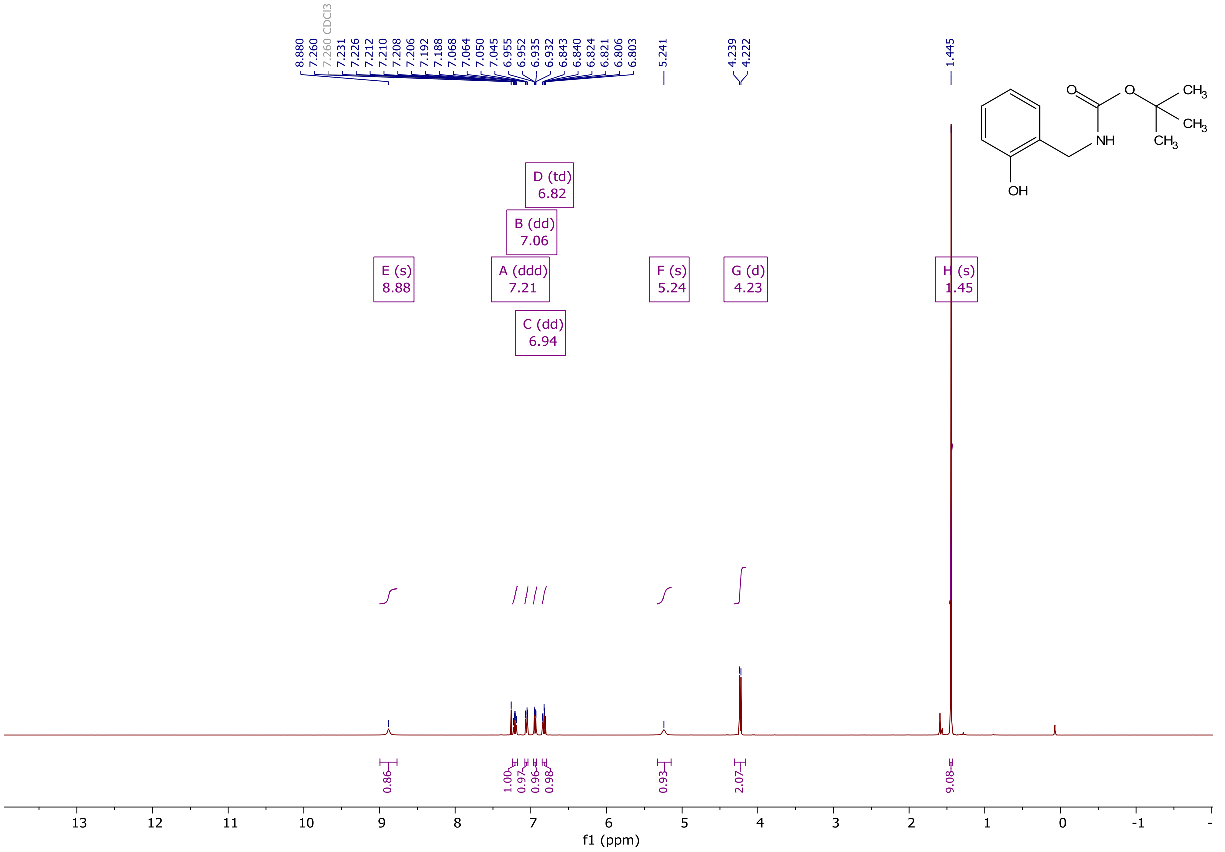

Compound XII, ^{13}C{1H} NMR (126 MHz, CDCl$_3$) spectrum

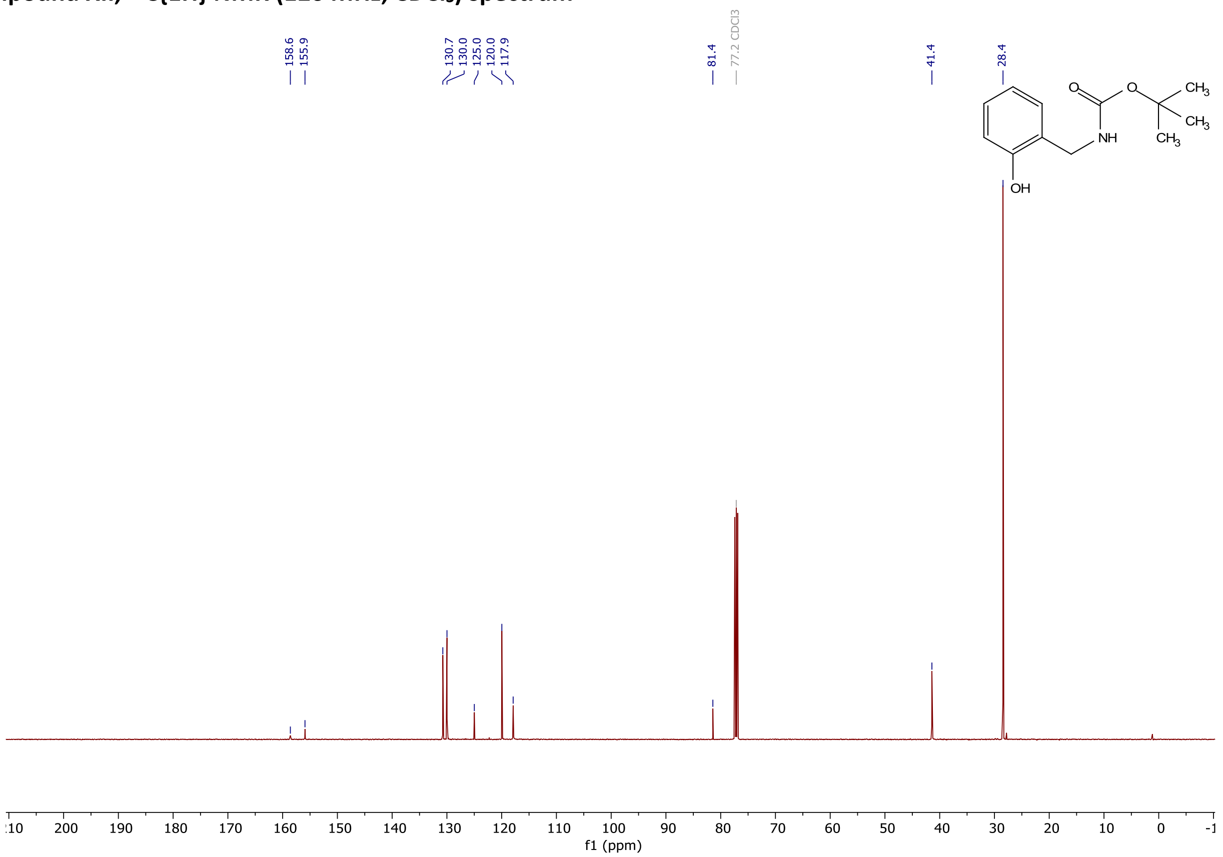

Compound XIII, ^{1}H NMR (400 MHz, $CDCl_3$) spectrum

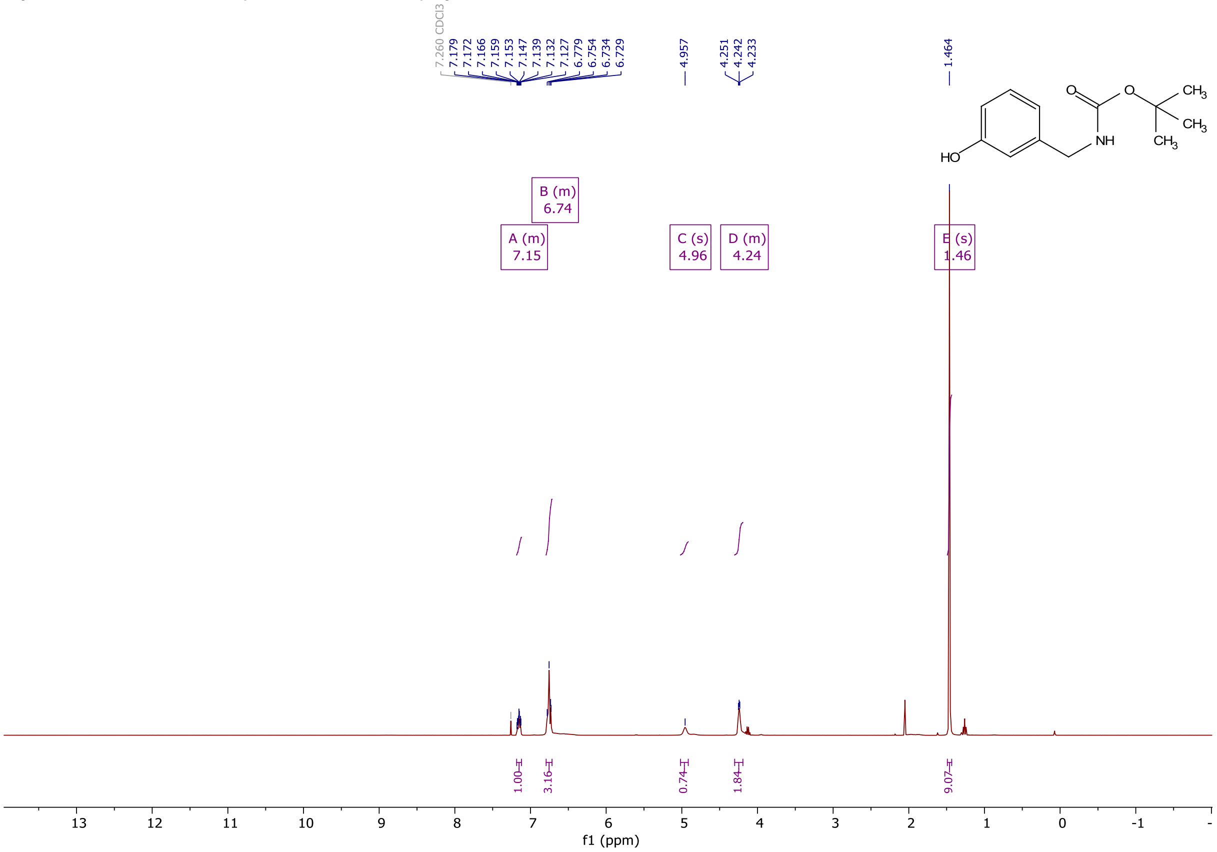

Compound XIII, $^{13}C\{1H\}$ NMR (126 MHz, $CDCl_3$) spectrum

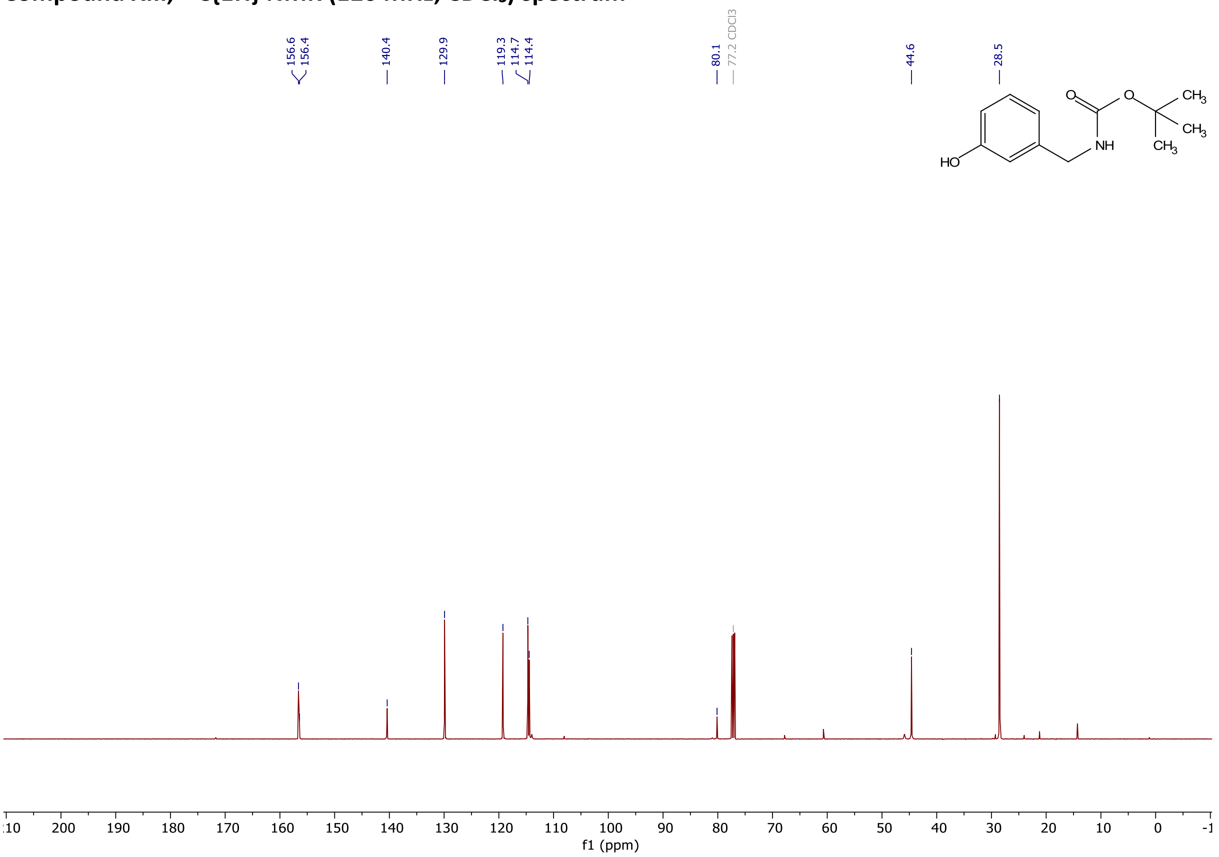

Compound XIV, LC-MS

A)

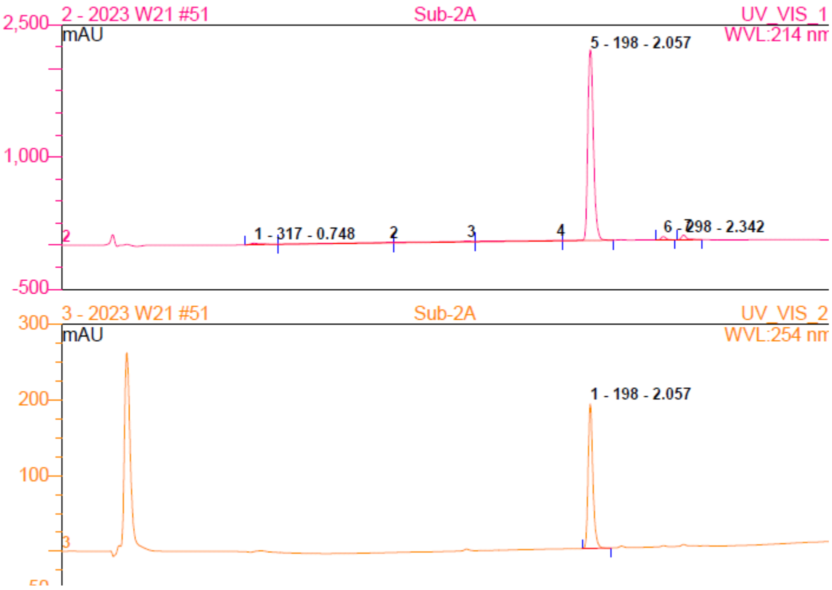

B)

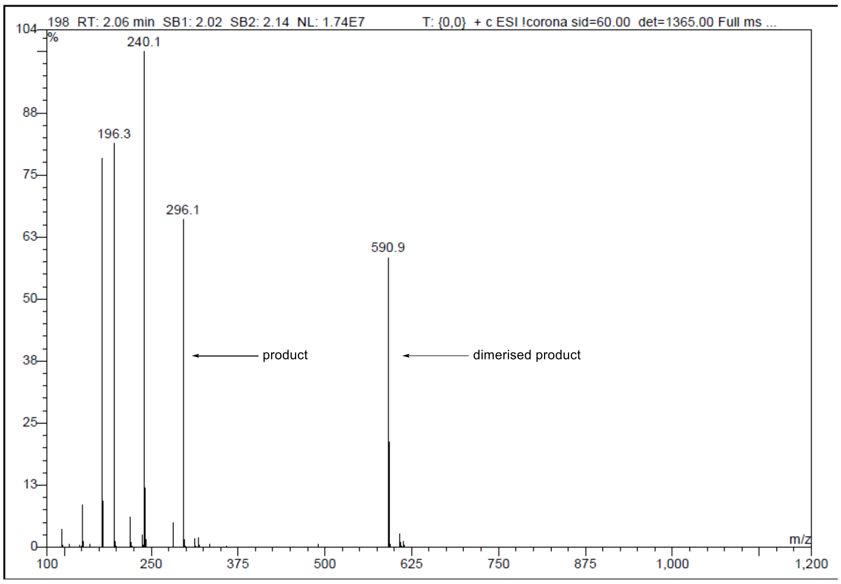

A) Analytical RP-HPLC of compound **XIV** on C18 column, acetonitrile gradient 5-100% of 0.05% formic acid in acetonitrile/water for 3 min. Top to bottom: UV detection at 214 and 254 nm. B) Low-resolution mass spectrum of **XIV**.

Compound XIV, ^{1}H NMR (400 MHz, $CDCl_3$) spectrum

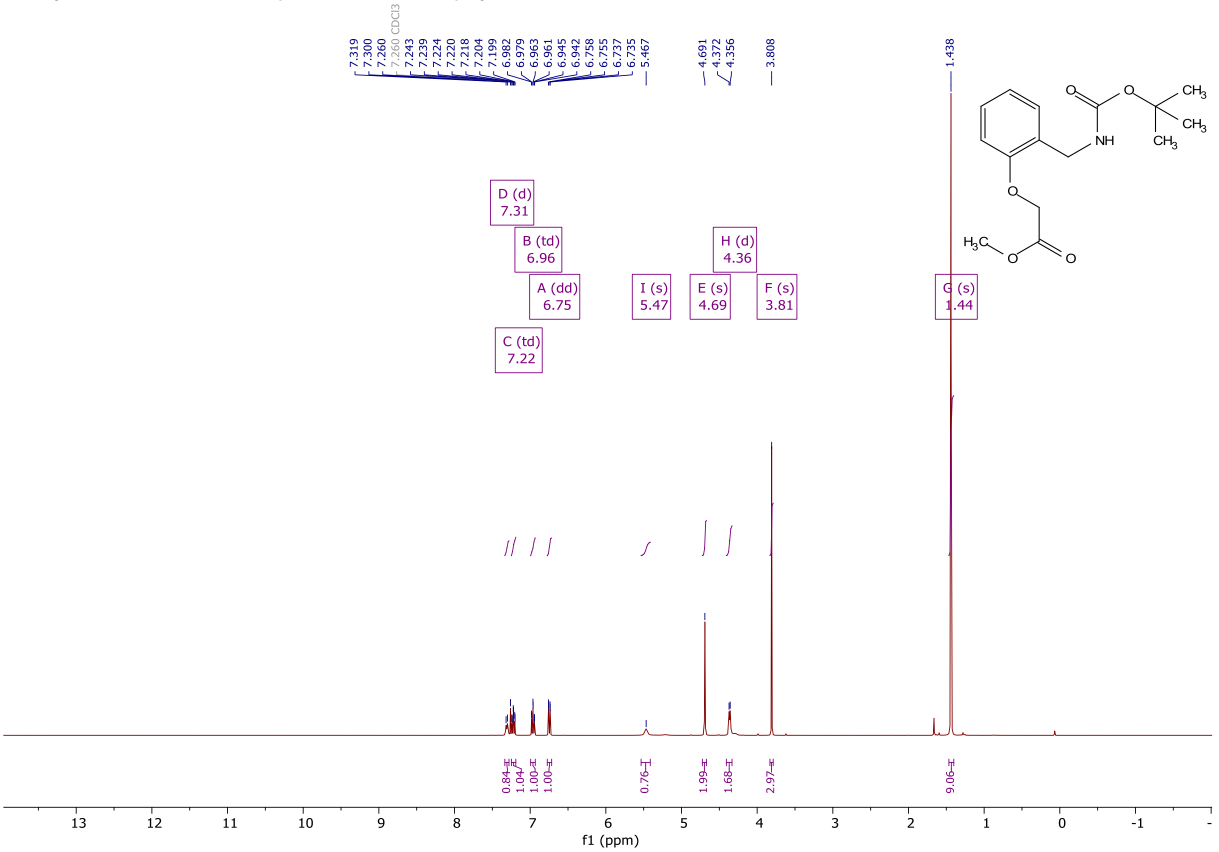

Compound XIV, $^{13}C\{1H\}$ NMR (126 MHz, $CDCl_3$) spectrum

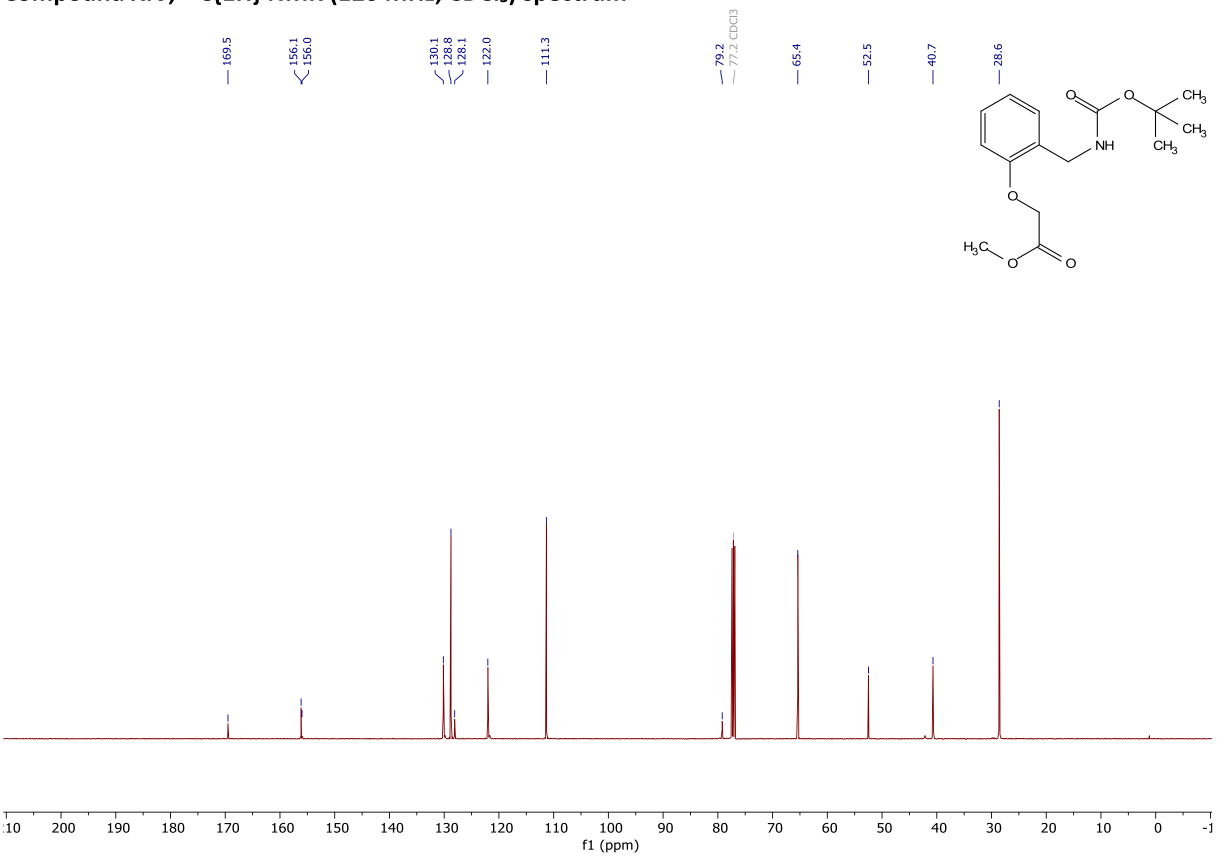

Compound XV, LC-MS

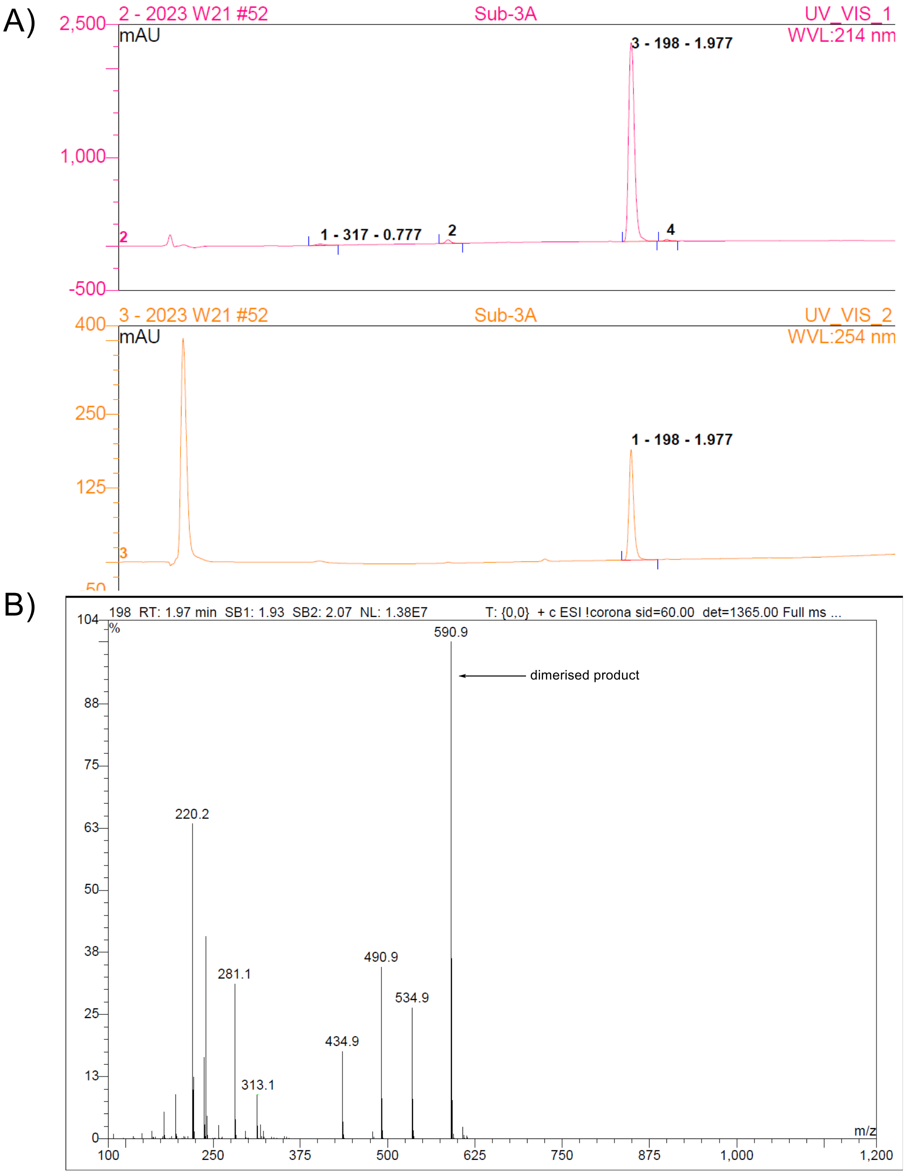

A) Analytical RP-HPLC of compound **XV** on C18 column, acetonitrile gradient 5-100% of 0.05% formic acid in acetonitrile/water for 3 min. Top to bottom: UV detection at 214 and 254 nm. B) Low-resolution mass spectrum of **XV**, dimerized product detected.

Compound XV, ^{1}H NMR (400 MHz, CDCl$_3$) spectrum

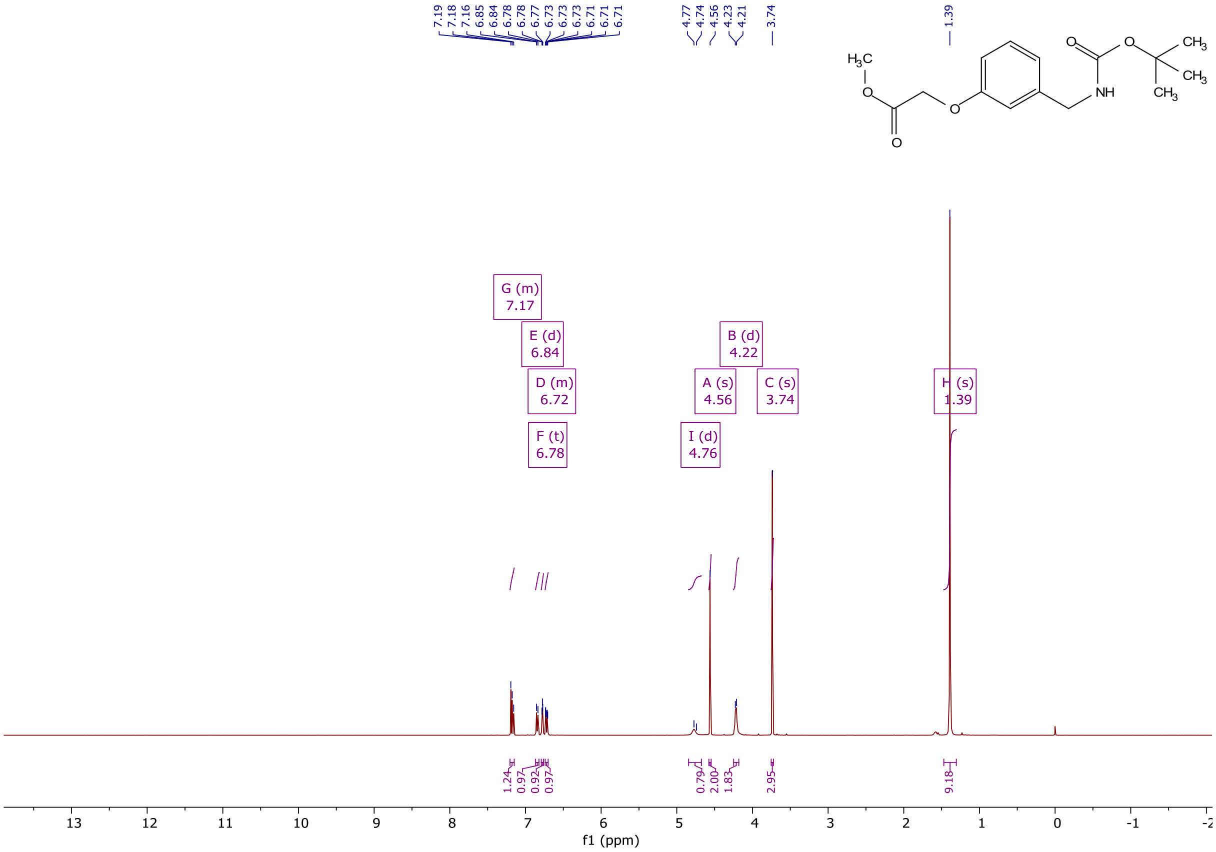

Compound XV, ^{13}C{1H} NMR (126 MHz, CDCl$_3$) spectrum

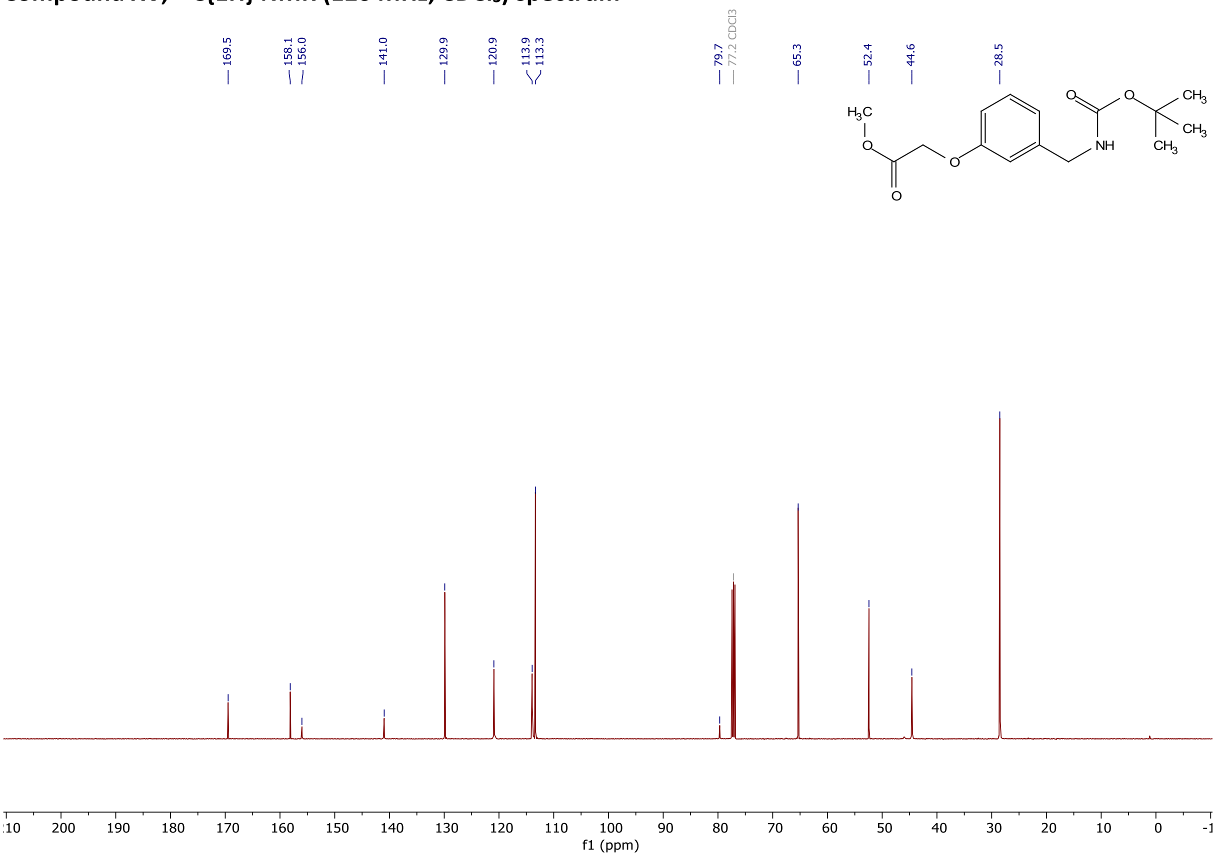

Compound XVI, LC-MS

A)

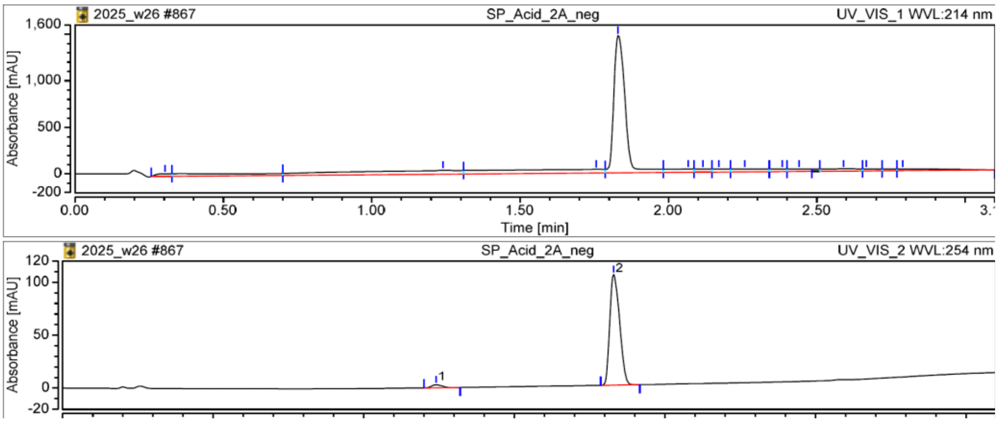

B)

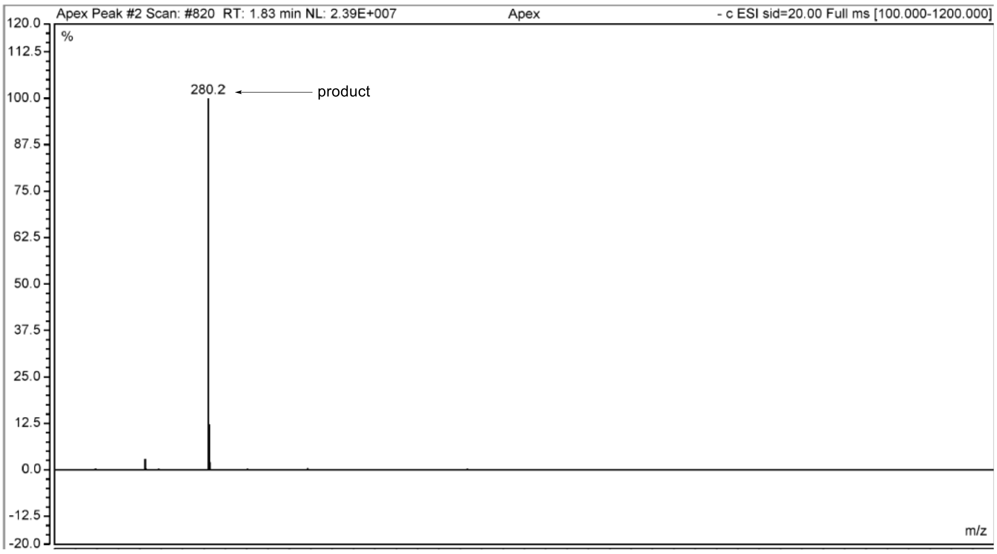

A) Analytical RP-HPLC of compound **XVI** on C18 column, acetonitrile gradient 5-100% of 0.05% formic acid in acetonitrile/water for 3 min. Top to bottom: UV detection at 214 and 254 nm. B) Low-resolution mass spectrum of **XVI**.

Compound XVI, ^{1}H NMR (400 MHz, $CDCl_3$) spectrum

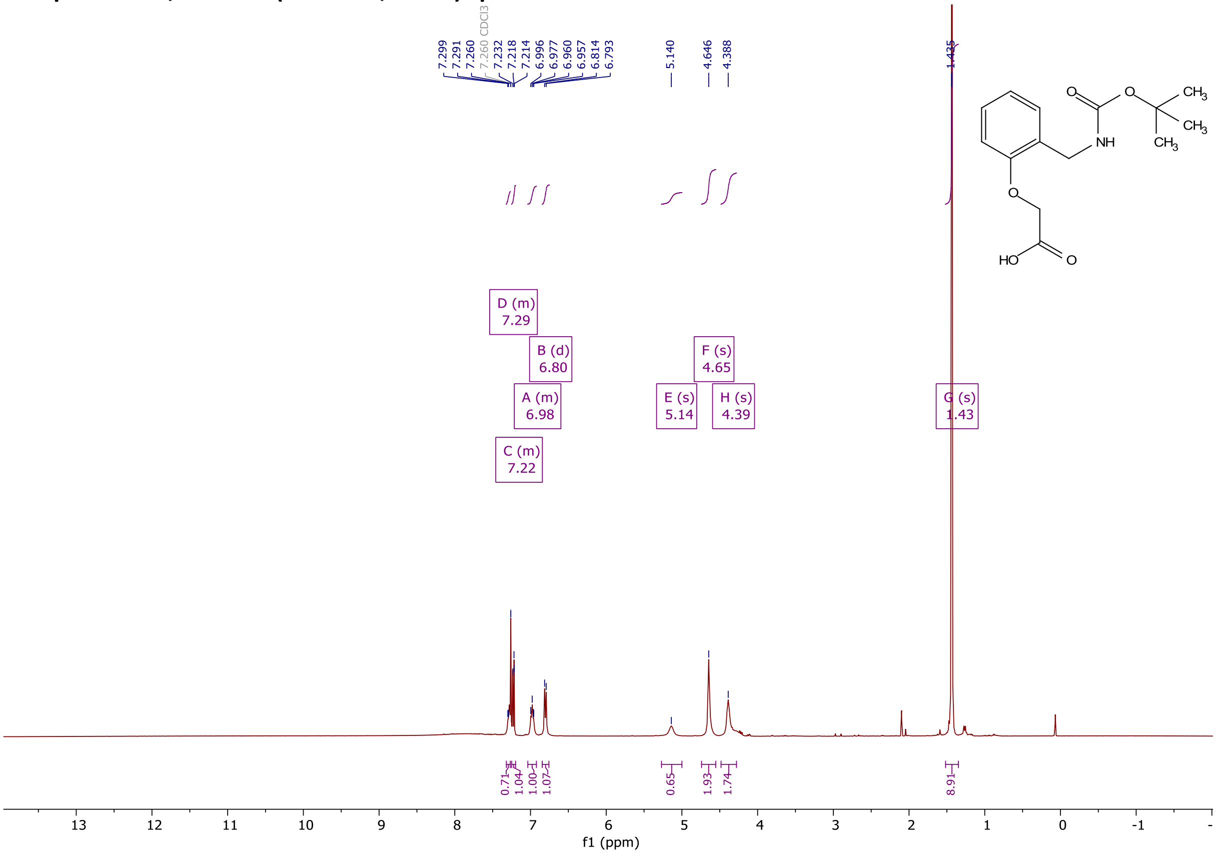

Compound XVI, $^{13}C\{1H\}$ NMR (126 MHz, $CDCl_3$) spectrum

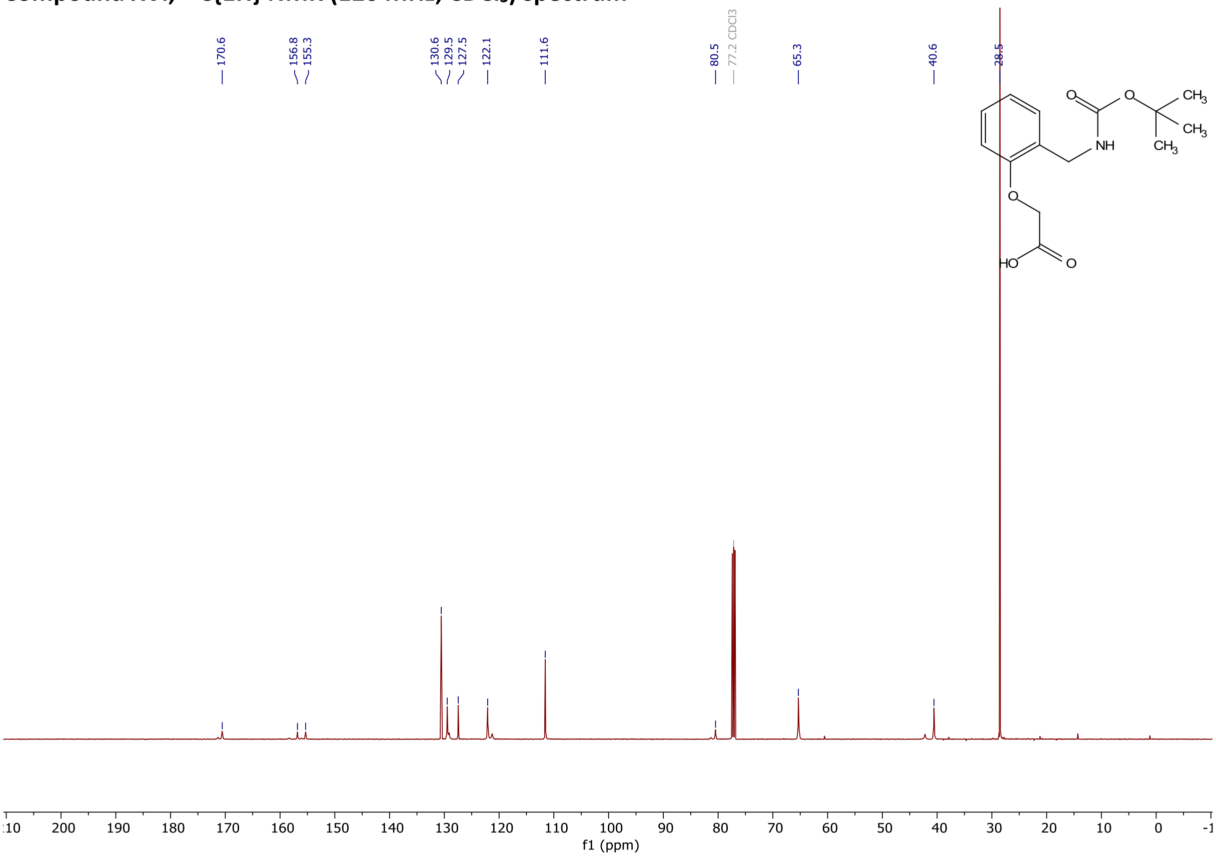

Compound XVII, LC-MS

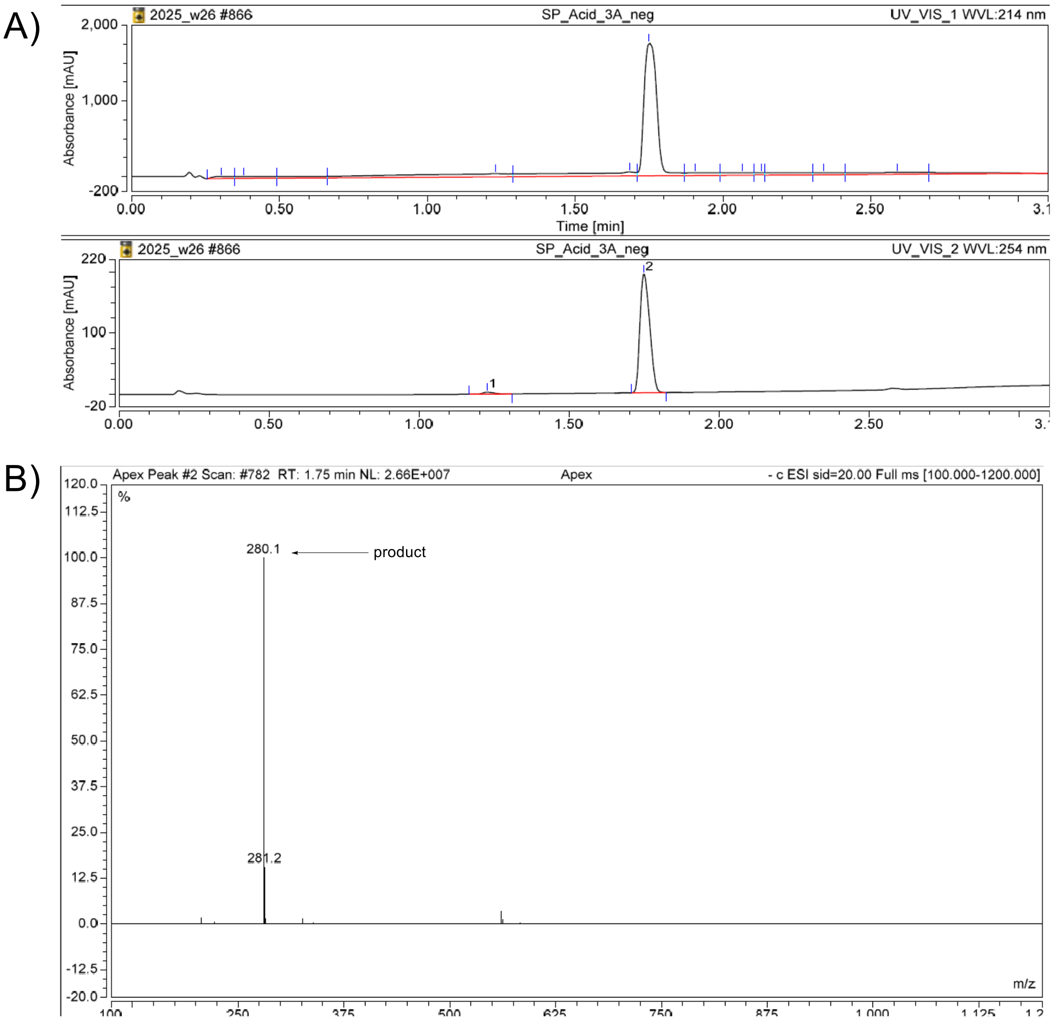

A) Analytical RP-HPLC of compound **XVII** on C18 column, acetonitrile gradient 5-100% of 0.05% formic acid in acetonitrile/water for 3 min. Top to bottom: UV detection at 214 and 254 nm. B) Low-resolution mass spectrum of **XVII**.

Compound XVII, ^{1}H NMR (400 MHz, CDCl$_3$) spectrum

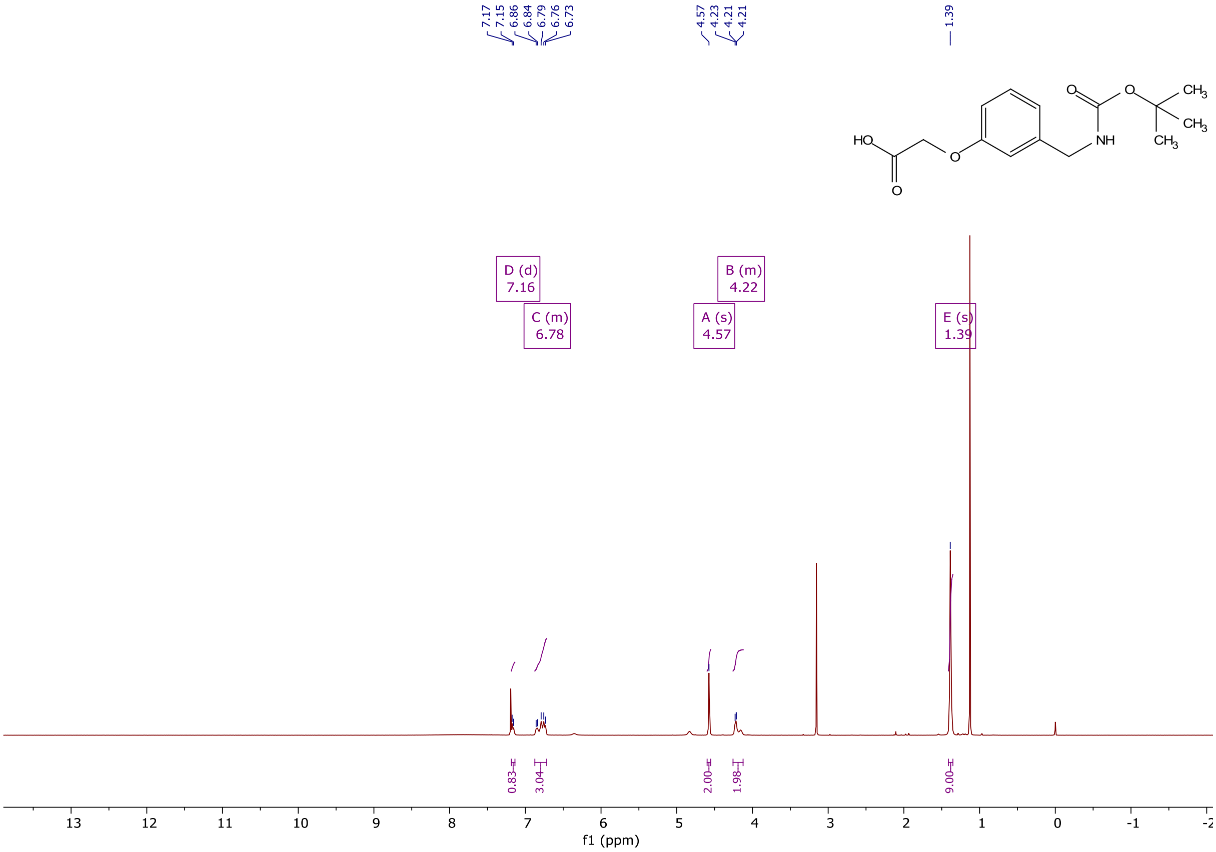

Compound XVII, ^{13}C{1H} NMR (101 MHz, CDCl$_3$) spectrum

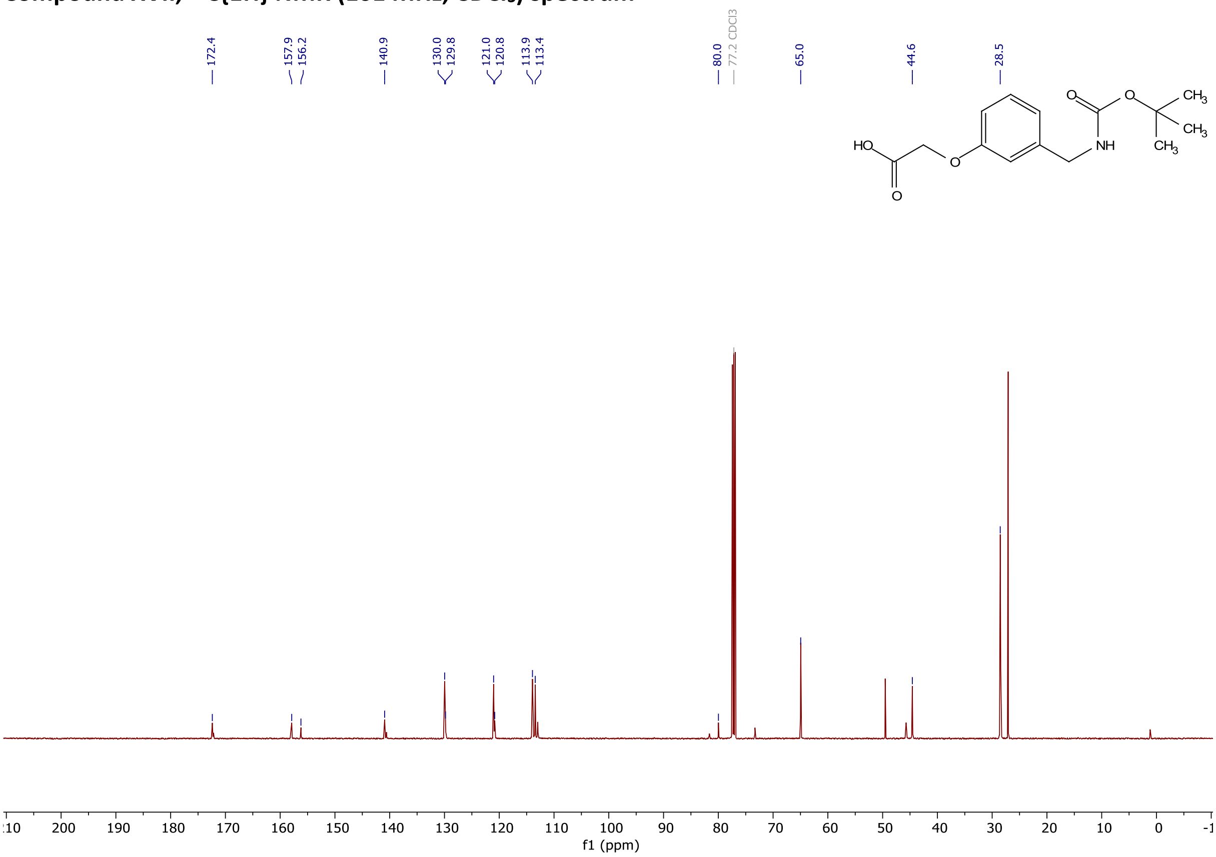

Compound 18, LC-MS

A)

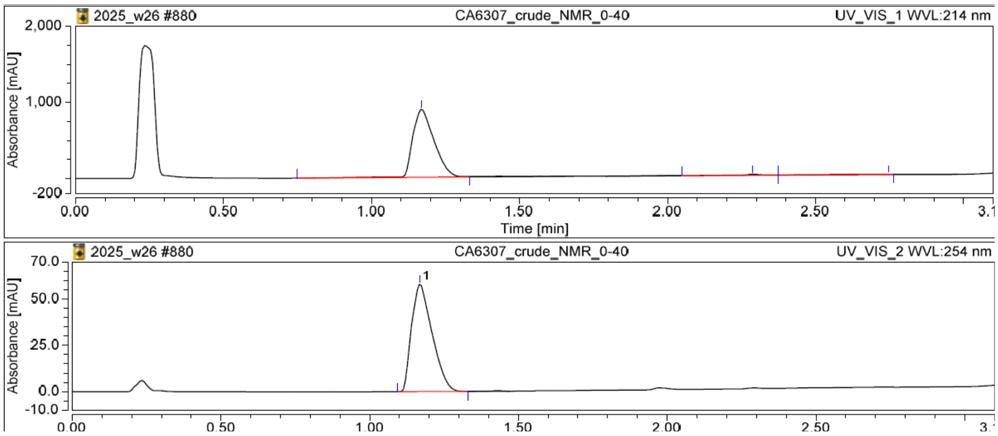

B)

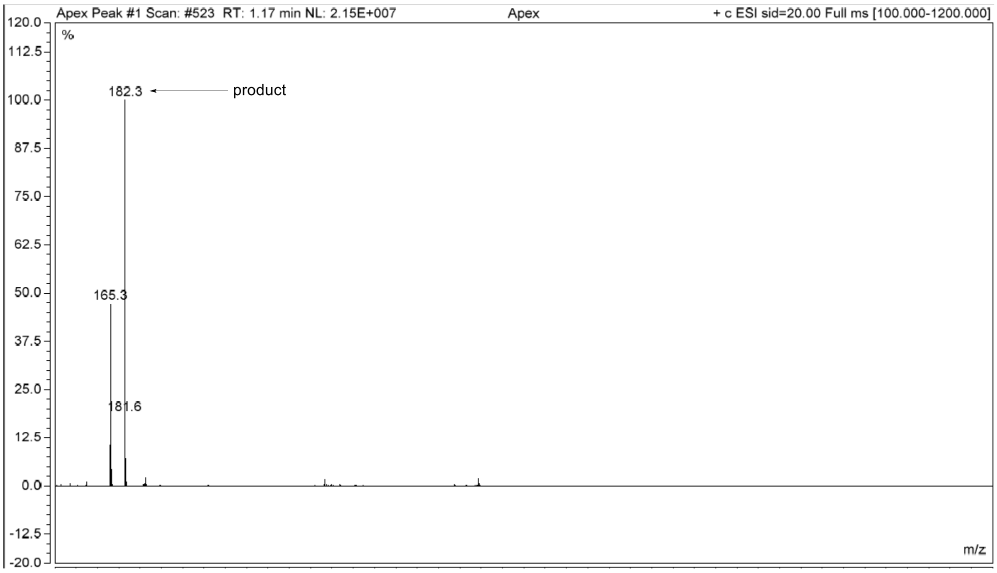

A) Analytical RP-HPLC of compound **18** on C18 column, acetonitrile gradient 0-40% of 0.05% formic acid in acetonitrile/water for 3 min. Top to bottom: UV detection at 214 and 254 nm. B) Low-resolution mass spectrum of **18**.

Compound 18, ^{1}H NMR (400 MHz, DMSO-d_6) spectrum

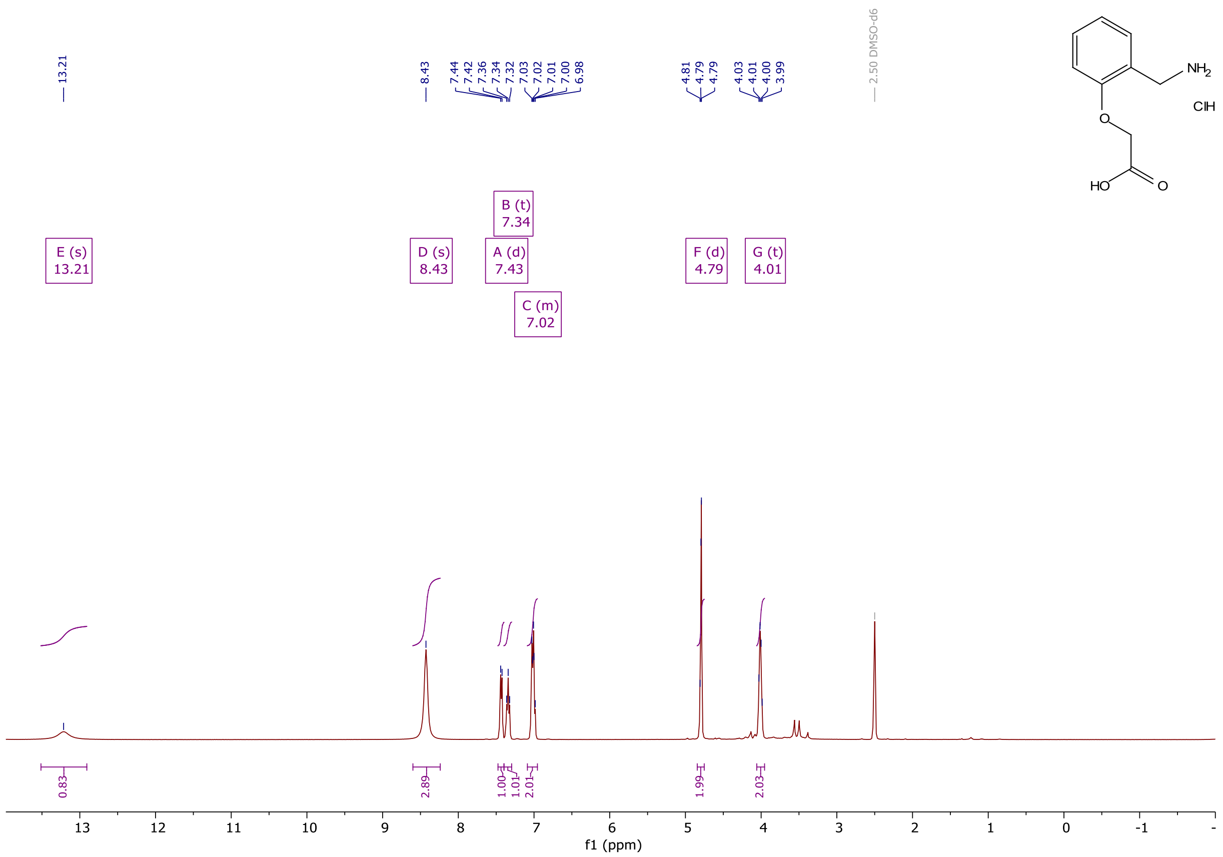

Compound 18, ^{13}C{1H} NMR (101 MHz, DMSO-d_6) spectrum

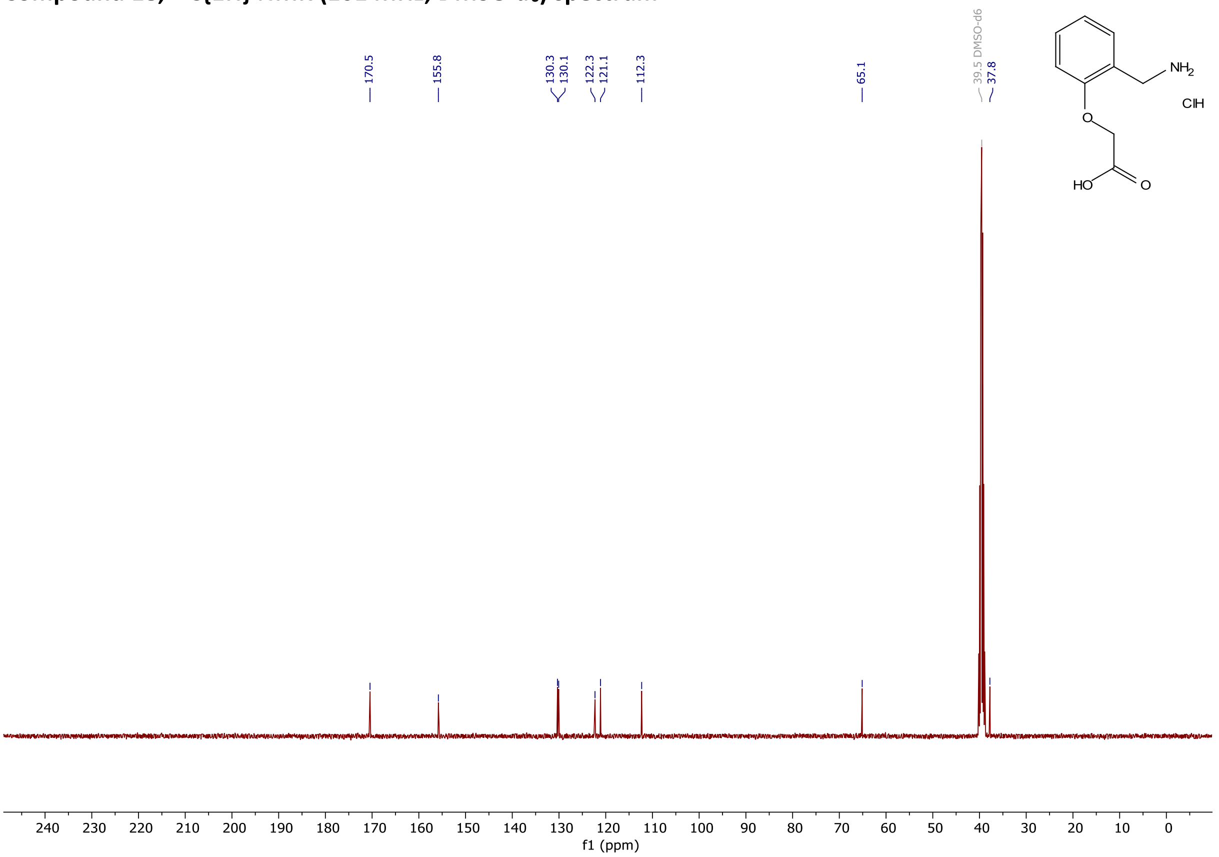

Compound 19, LC-MS

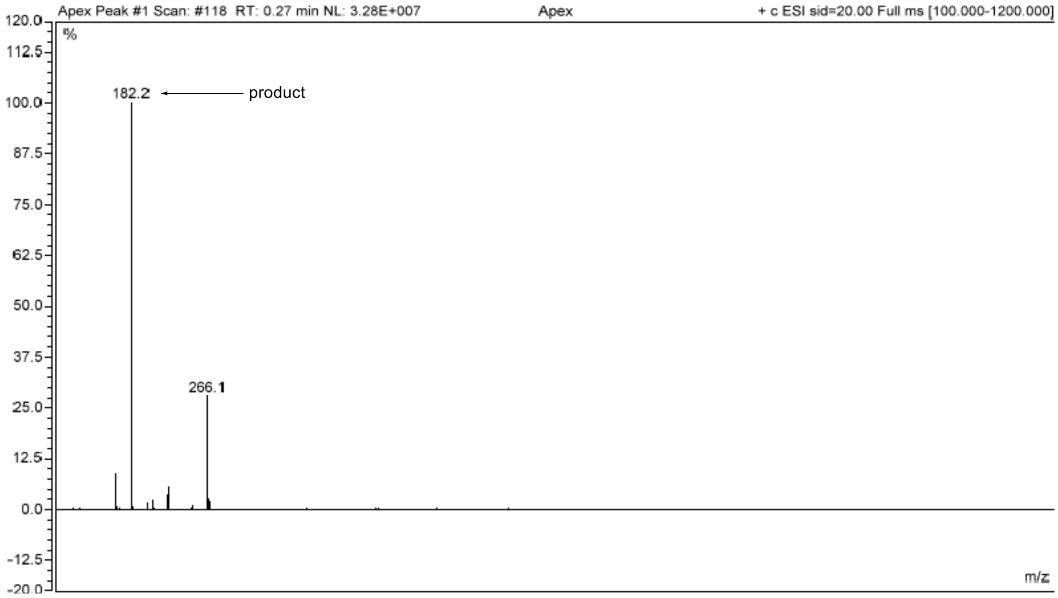

Low-resolution mass spectrum of **19**.

Compound 19, ^{1}H NMR (400 MHz, DMSO-d_6) spectrum

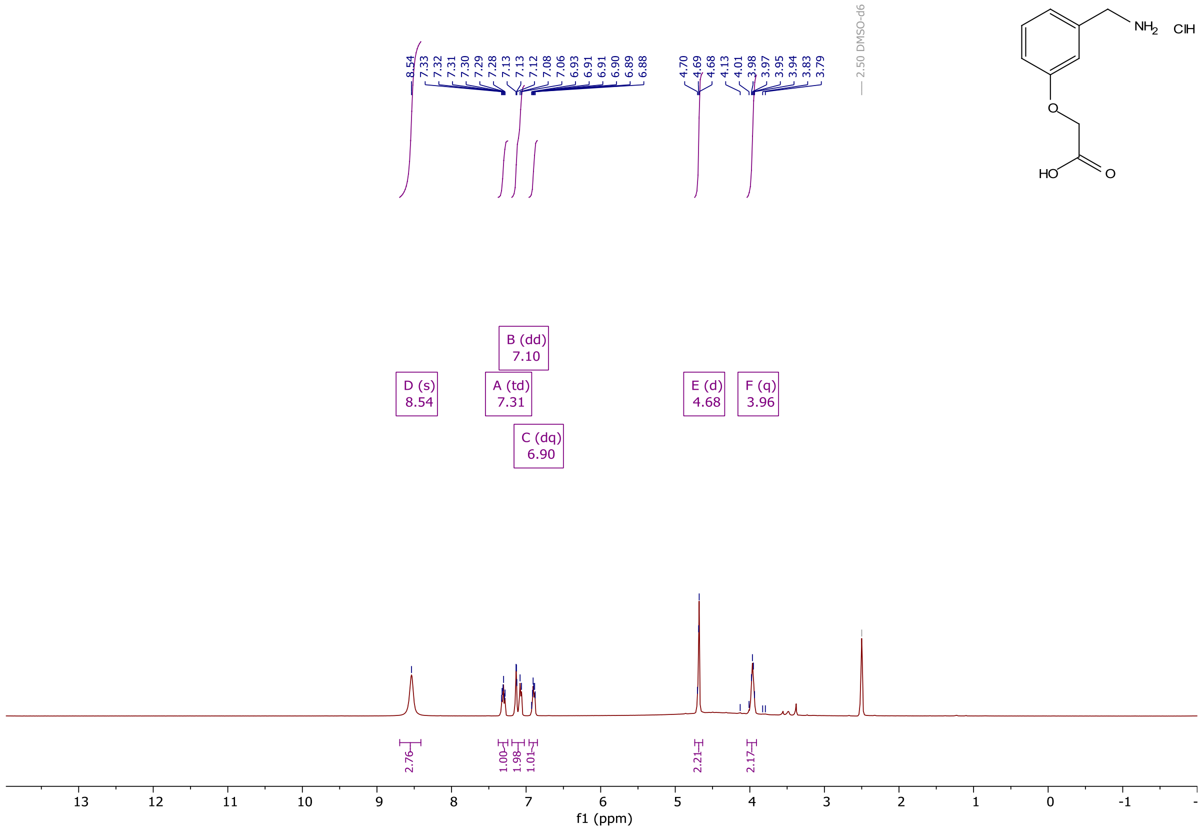

Compound 19, $^{13}C\{1H\}$ NMR (101 MHz, DMSO-d_6) spectrum

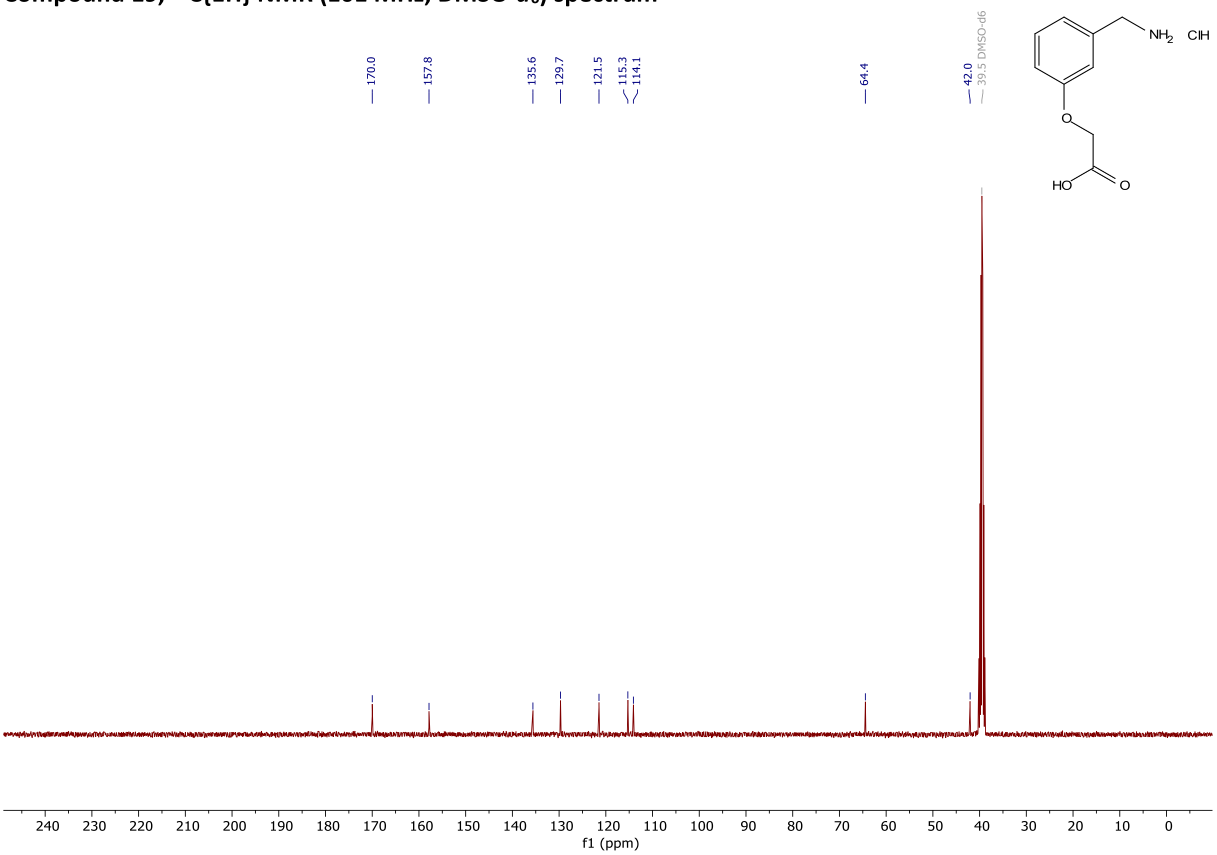

Compound XXI, LC-MS

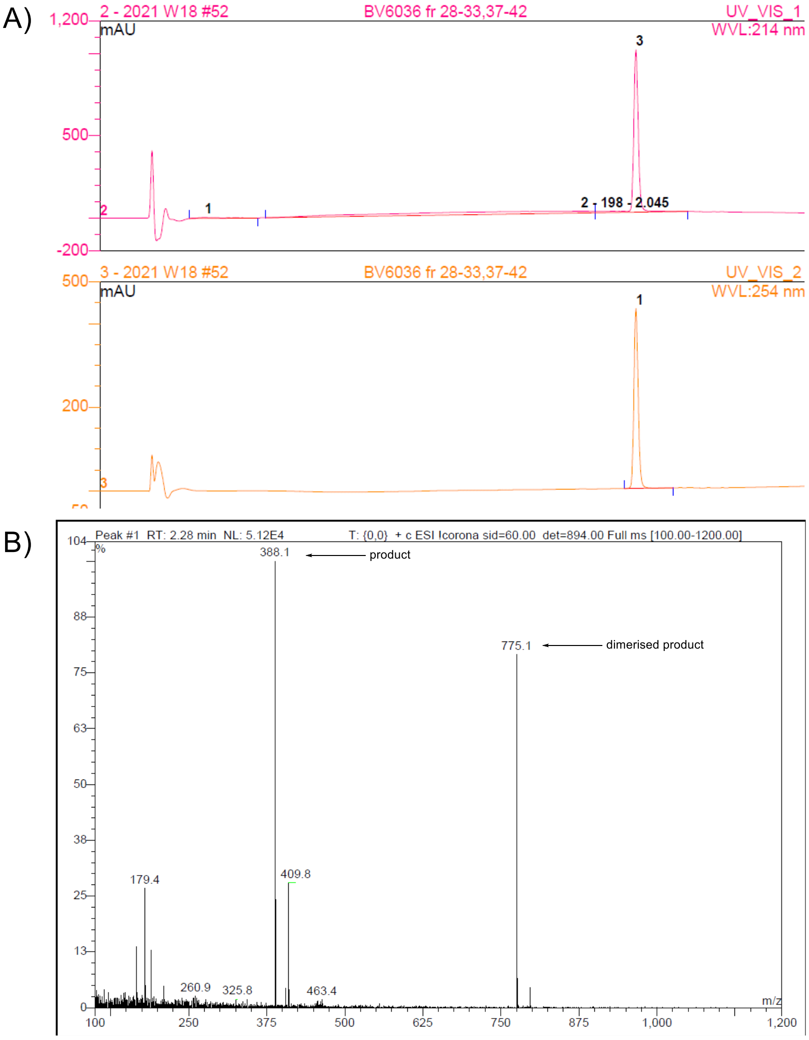

A) Analytical RP-HPLC of compound **XXI** on C18 column, acetonitrile gradient 5-100% of 0.05% formic acid in acetonitrile/water for 3 min. Top to bottom: UV detection at 214 and 254 nm. B) Low-resolution mass spectrum of **XXI**.

Compound XXI, ^{1}H NMR (400 MHz, DMSO-d_6) spectrum

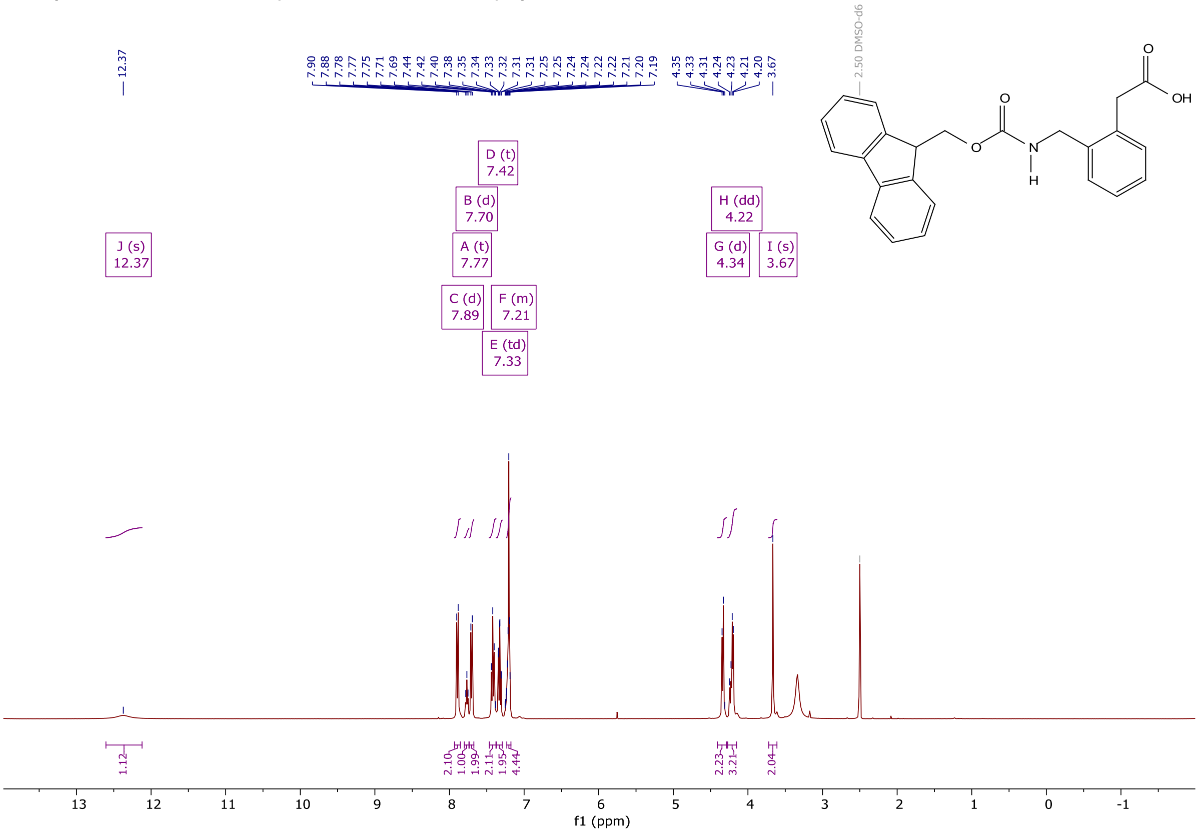

Compound XXI, ^{13}C{1H} NMR (101 MHz, DMSO-d_6) spectrum

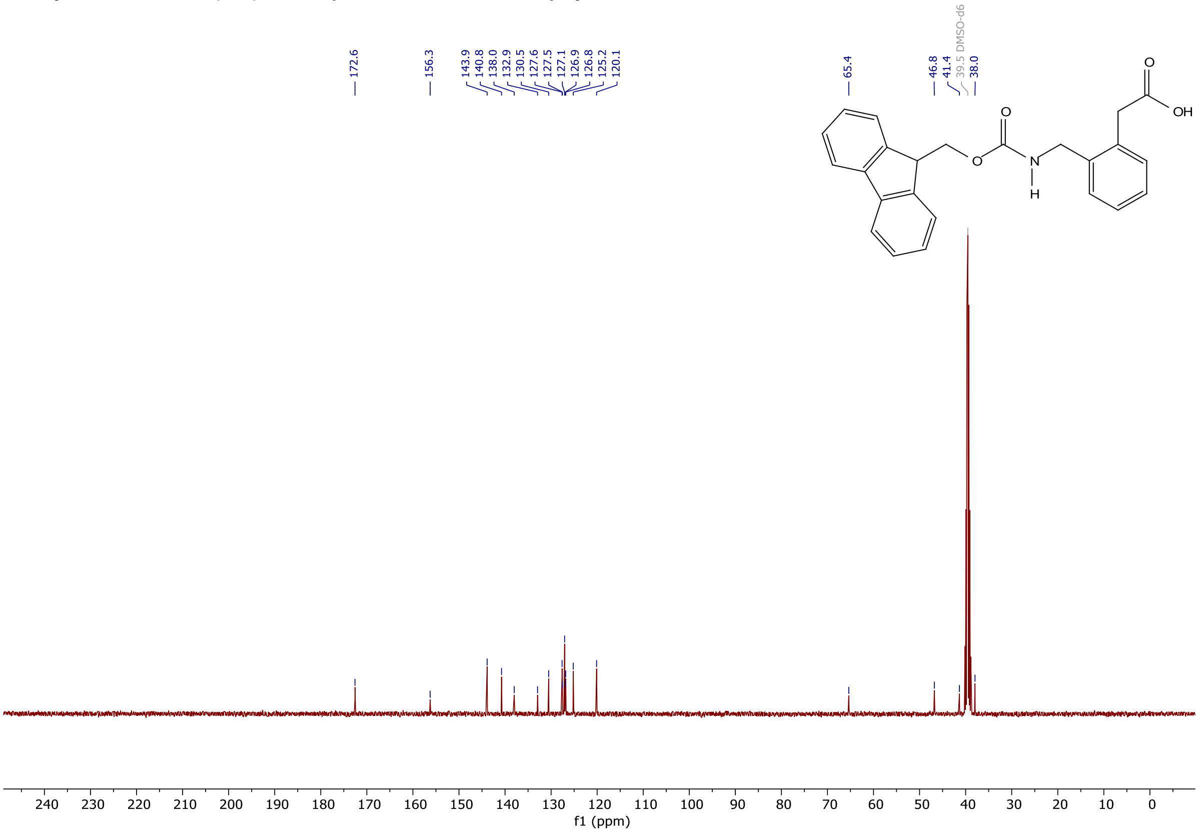

Compound XXII, LC-MS

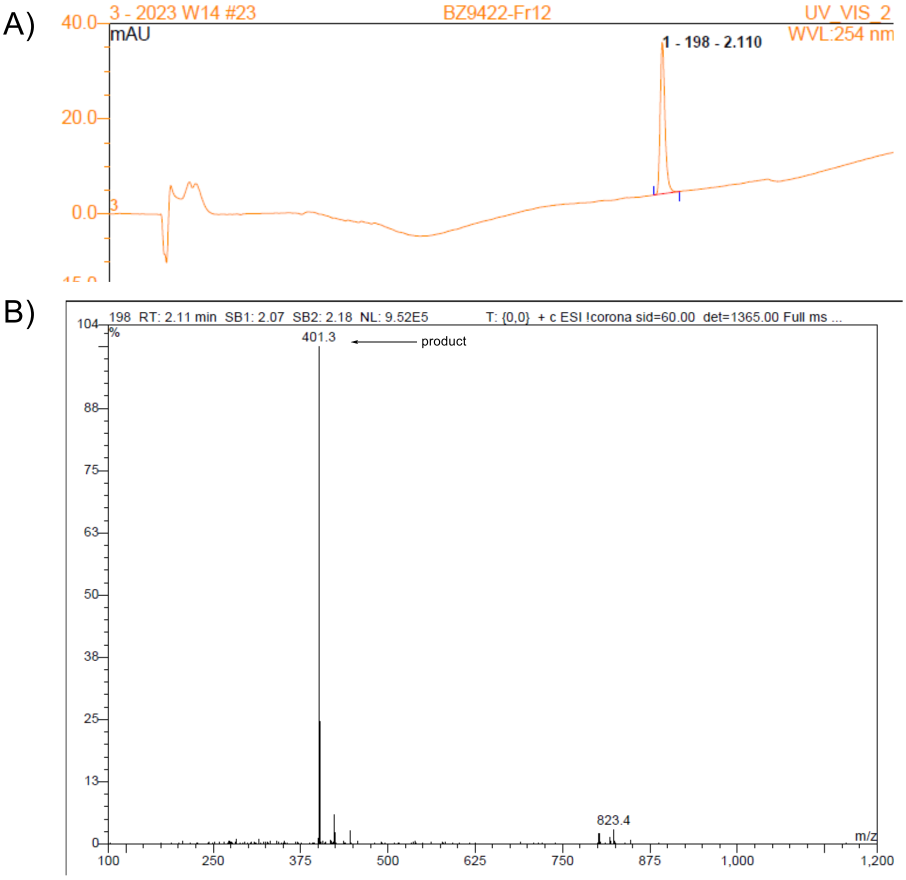

A) Analytical RP-HPLC of compound **XXII** on C18 column, acetonitrile gradient 5-100% of 0.05% formic acid in acetonitrile/water for 3 min. UV detection at 254 nm. B) Low-resolution mass spectrum of **XXII**

Compound XXII, ^{1}H NMR (500 MHz, $CDCl_3$) spectrum

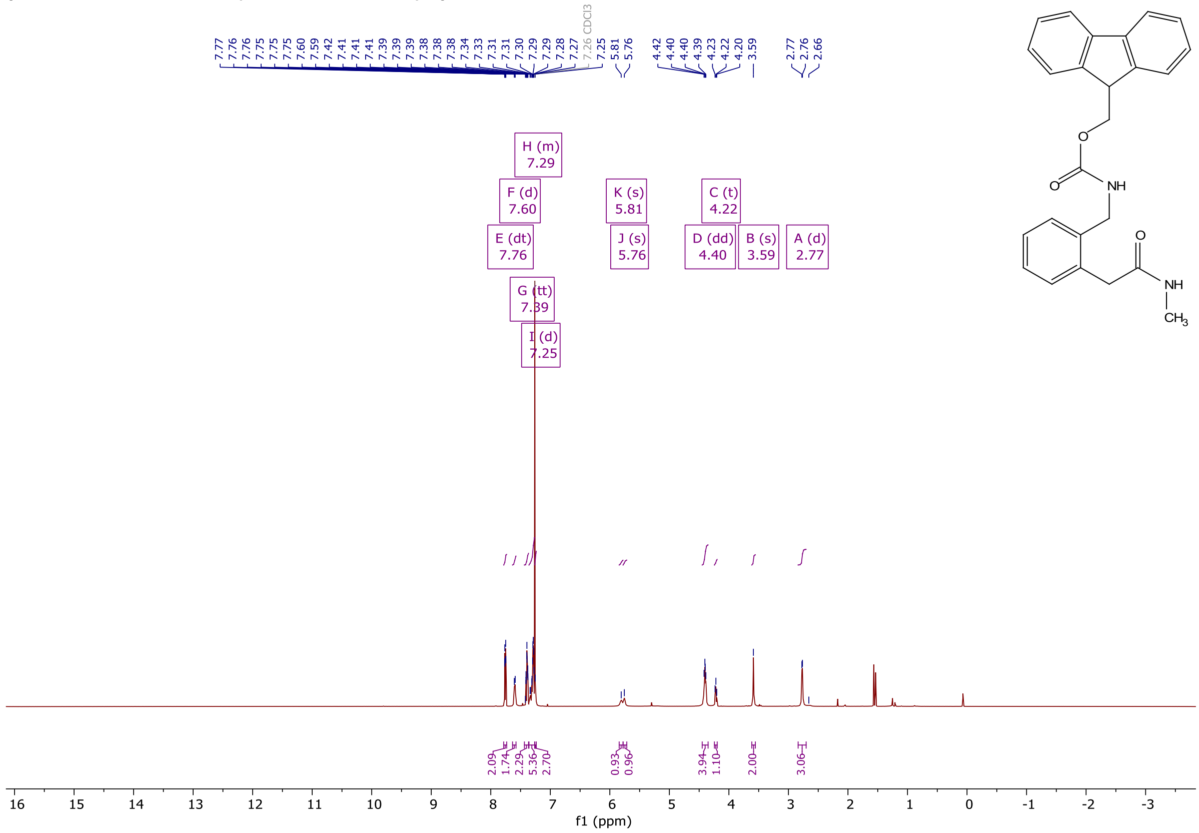

Compound XXII, ^{13}C{1H} NMR (126 MHz, $CDCl_3$) spectrum

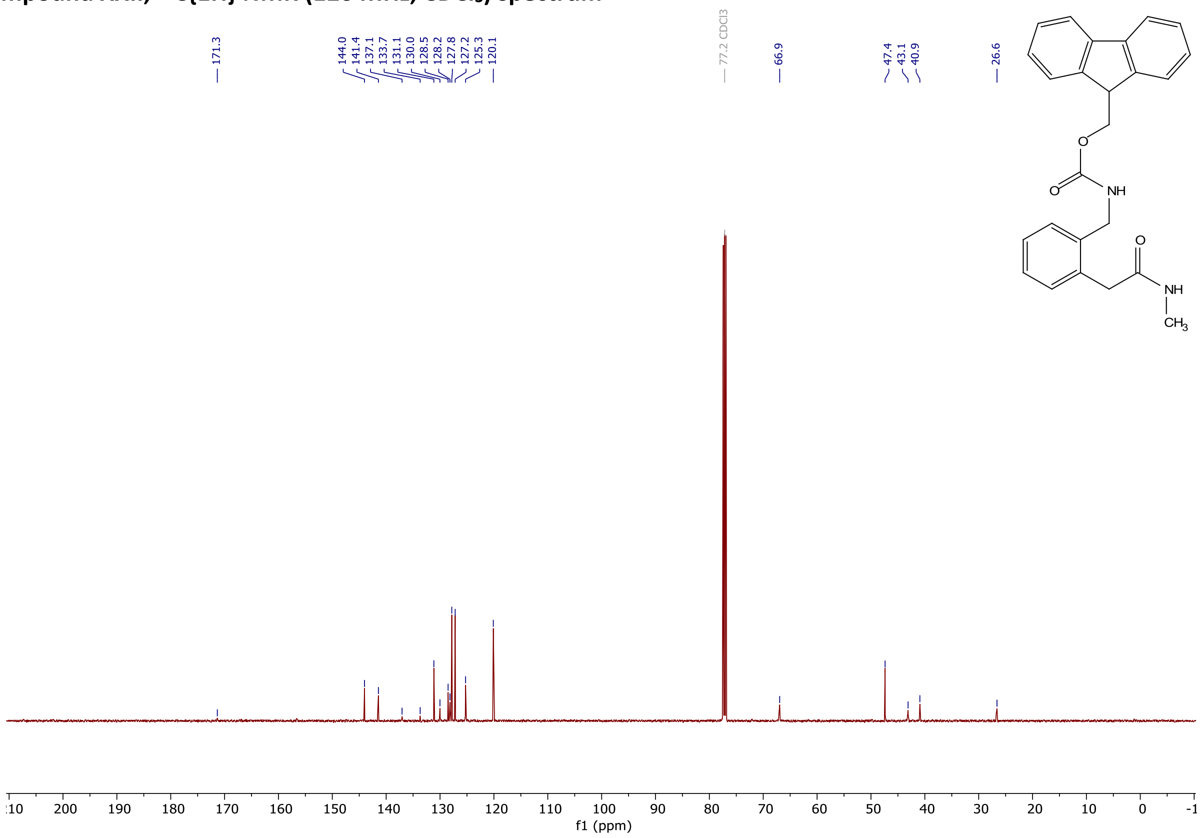

Compound XXIII, LC-MS

A)

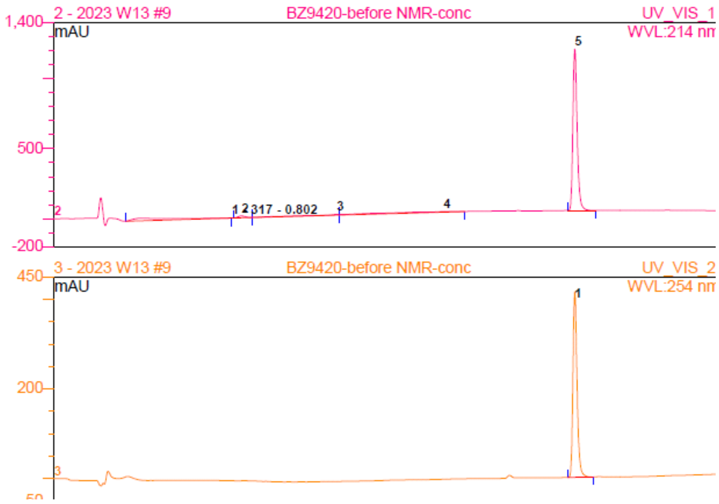

B)

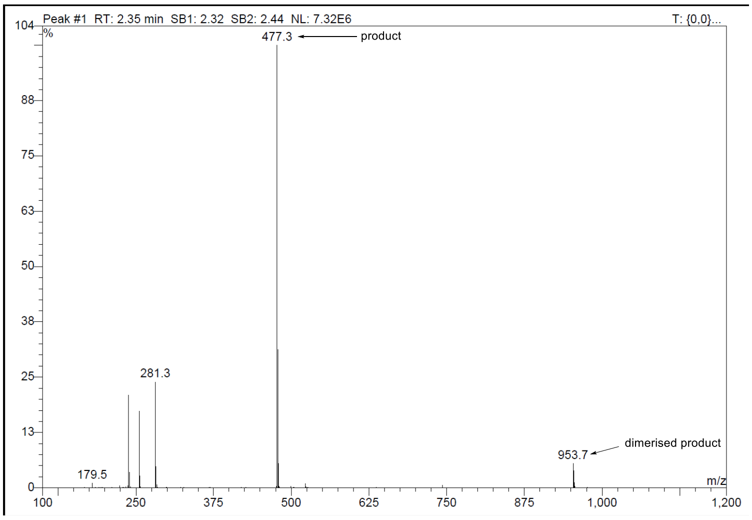

A) Analytical RP-HPLC of compound **XXIII** on C18 column, acetonitrile gradient 5-100% of 0.05% formic acid in acetonitrile/water for 3 min. Top to bottom: UV detection at 214 and 254 nm. B) Low-resolution mass spectrum of **XXIII**.

Compound XXIII, ^{1}H NMR (500 MHz, $CDCl_3$) spectrum

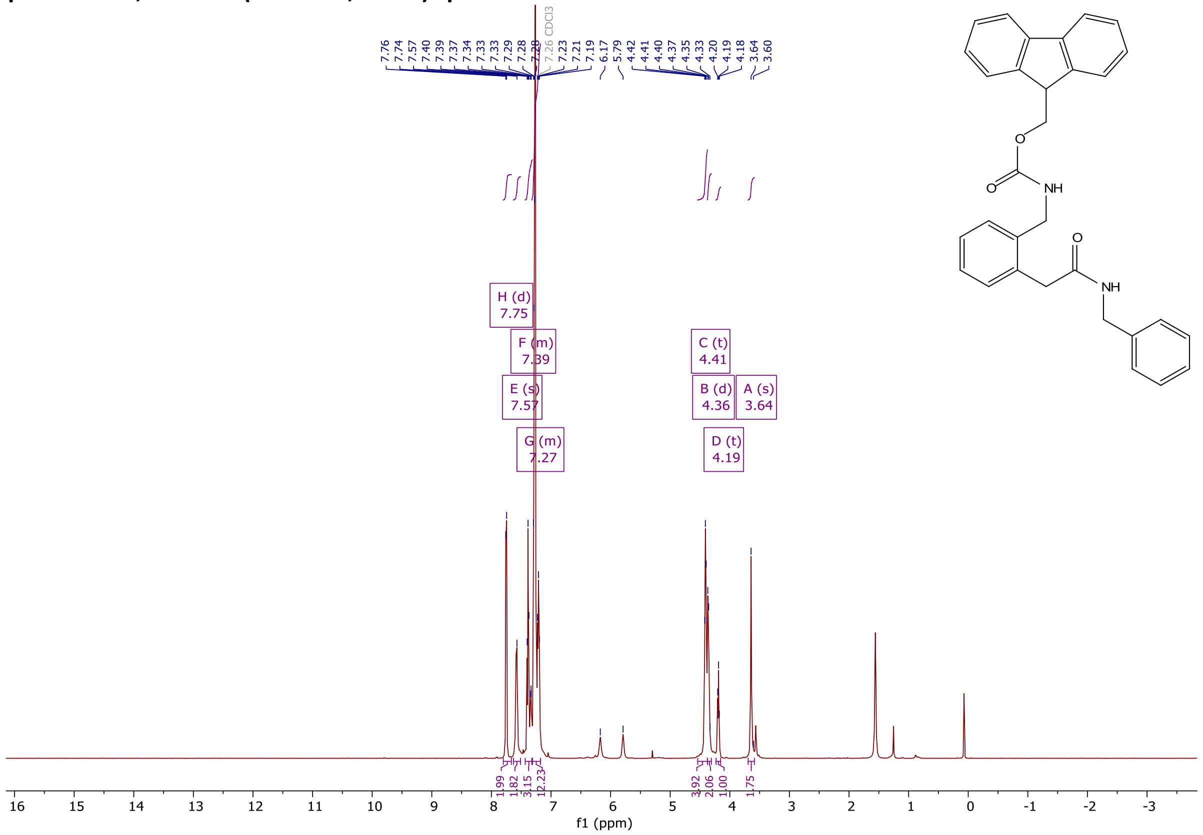

Compound XXIII, ^{13}C{1H} NMR (126 MHz, $CDCl_3$) spectrum

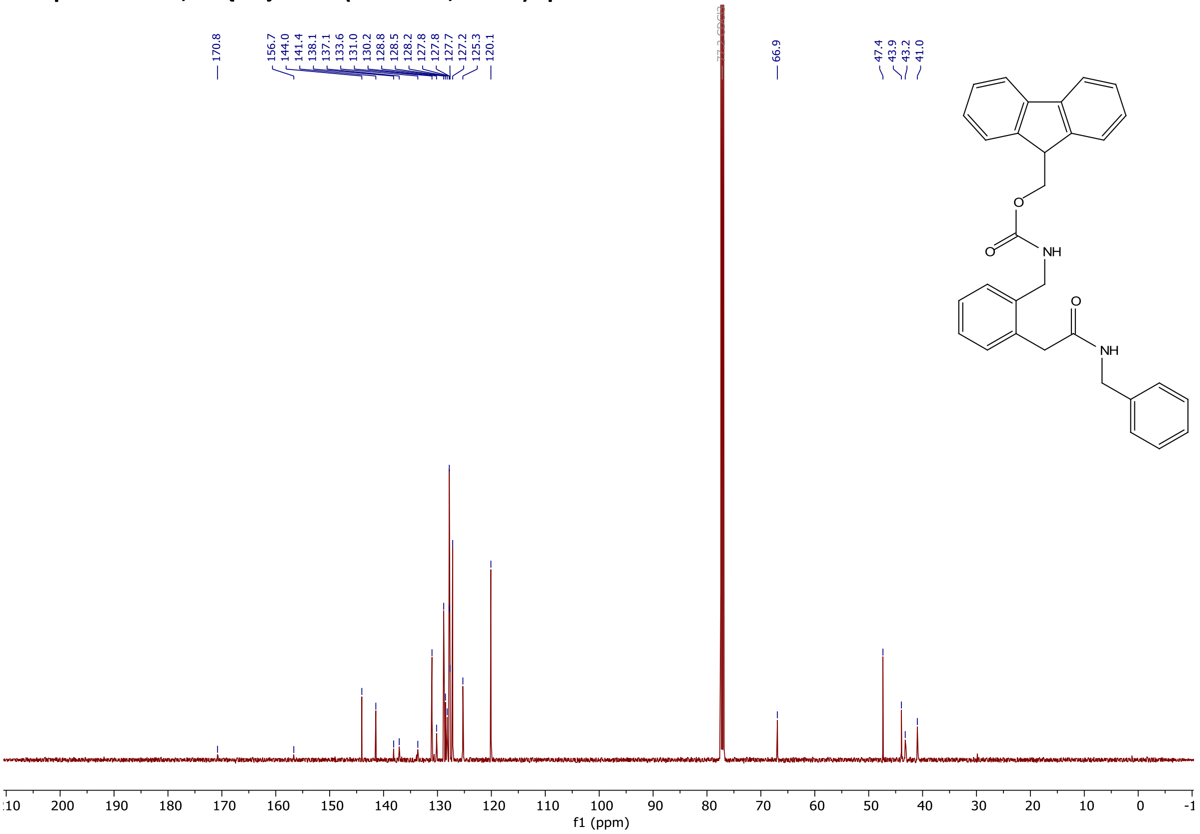

Compound XXIV, LC-MS

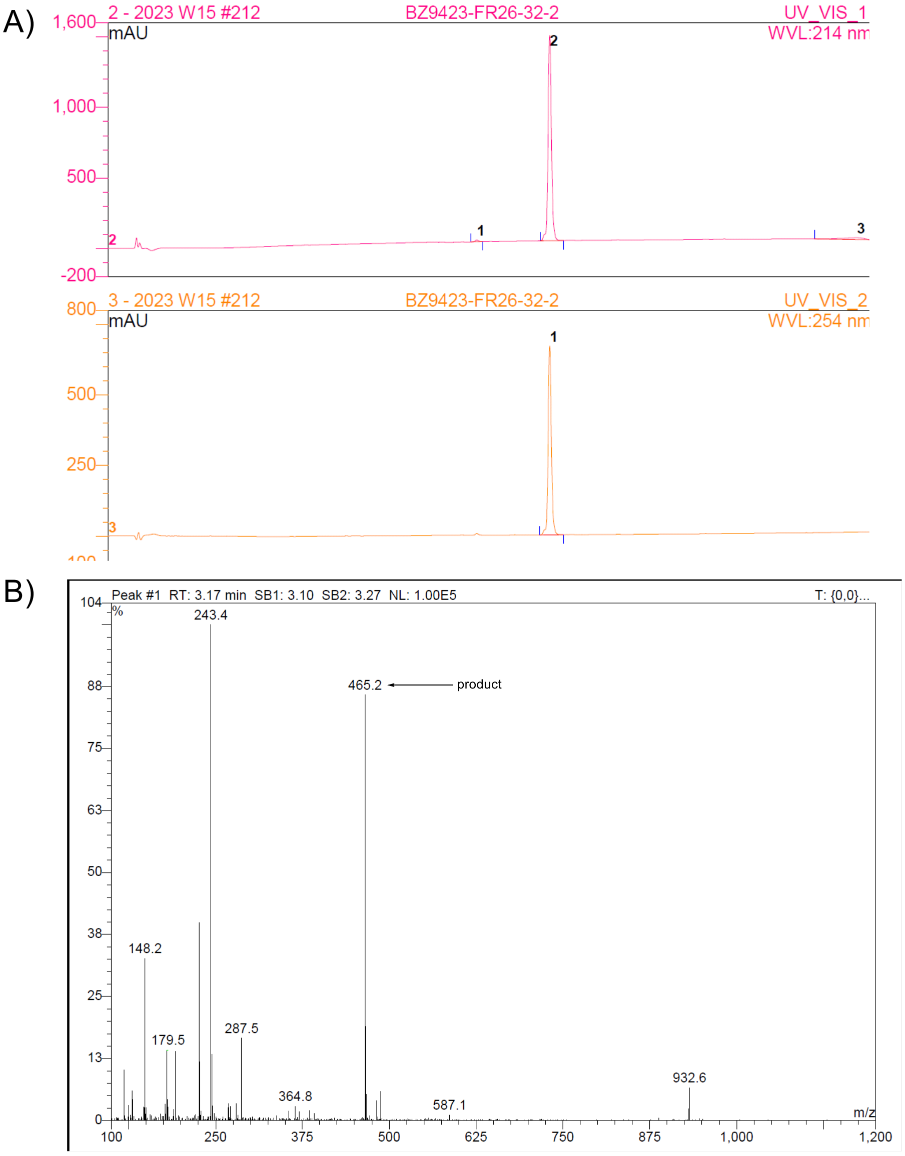

A) Analytical RP-HPLC of compound **XXIV** on C18 column, acetonitrile gradient 5-100% of 0.05% formic acid in acetonitrile/water for 5 min. Top to bottom: UV detection at 214 and 254 nm. B) Low-resolution mass spectrum of **XXIV.**

Compound XXIV, ^{1}H NMR (500 MHz, $CDCl_3$) spectrum

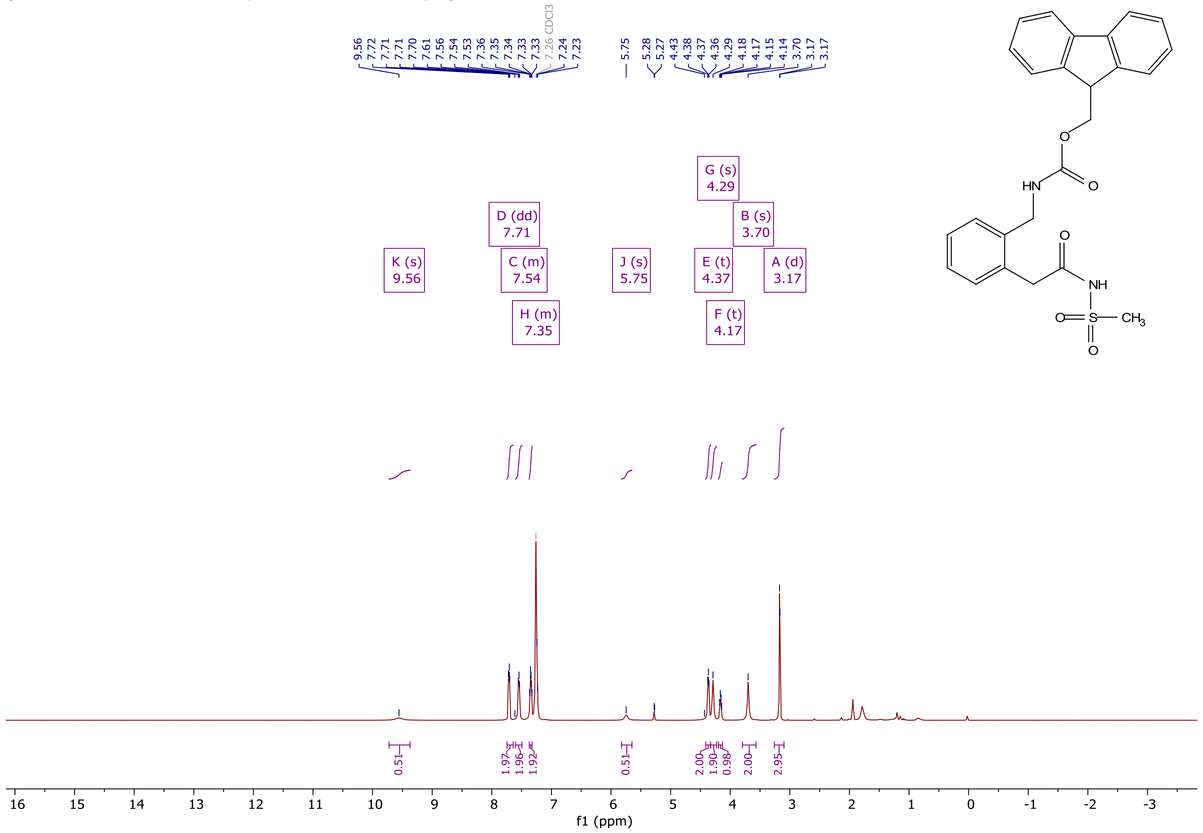

Compound XXIV, ^{13}C{1H} NMR (126 MHz, $CDCl_3$) spectrum

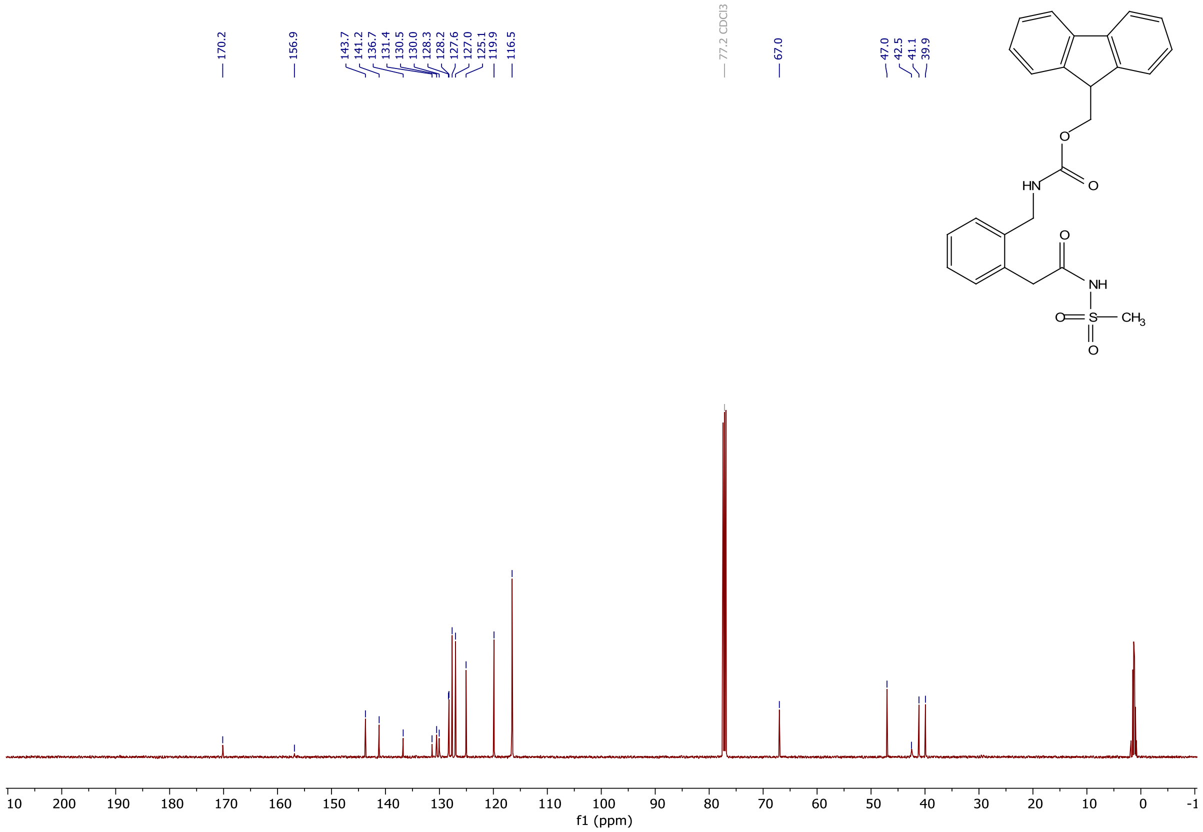

Compound XXV, LC-MS

A)

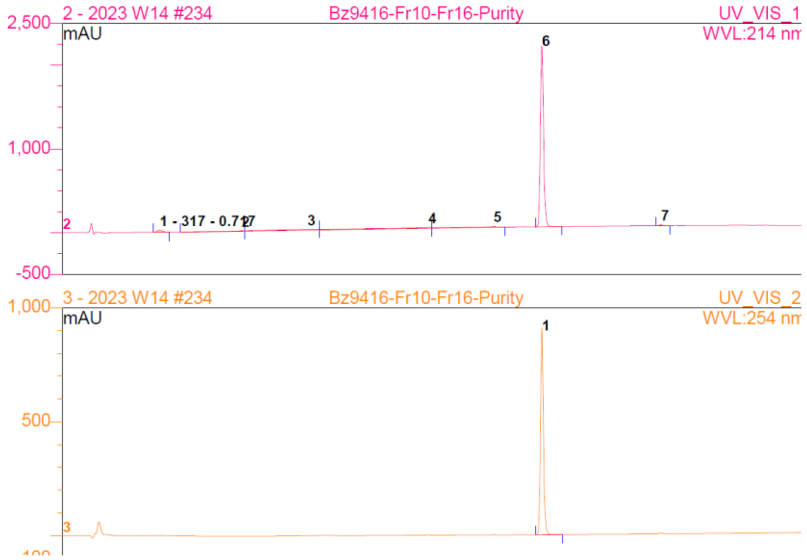

B)

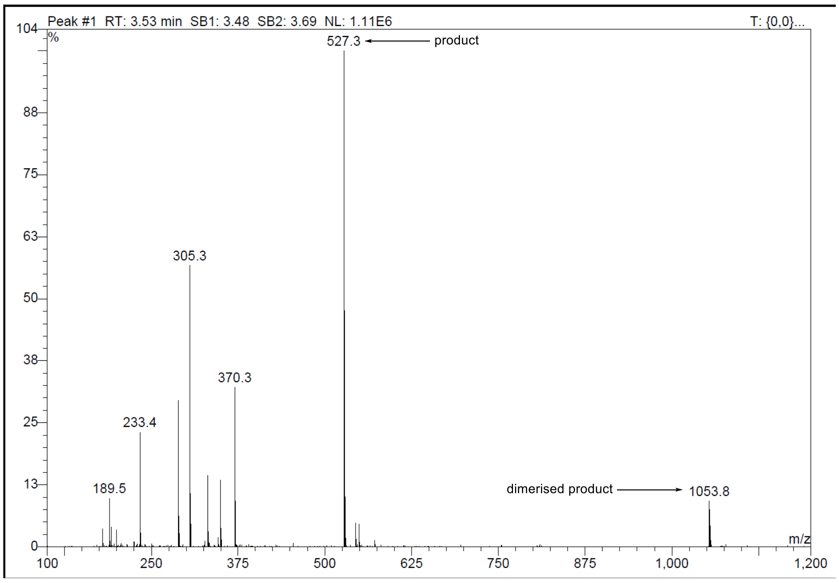

A) Analytical RP-HPLC of compound **XXV** on C18 column, acetonitrile gradient 5-100% of 0.05% formic acid in acetonitrile/water for 5 min. Top to bottom: UV detection at 214 and 254 nm. B) Low-resolution mass spectrum of **XXV**

Compound XXV, ^{1}H NMR (500 MHz, $CDCl_3$) spectrum

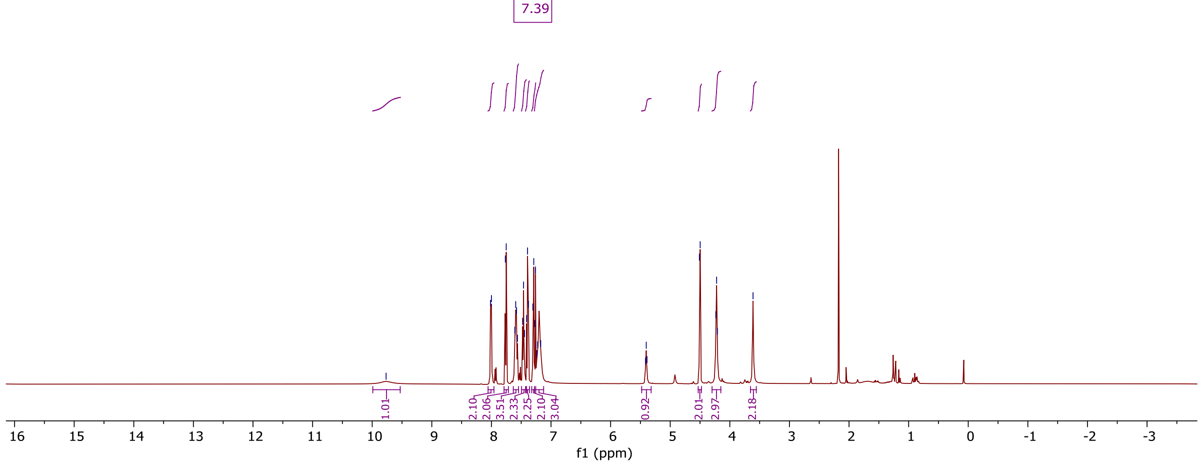

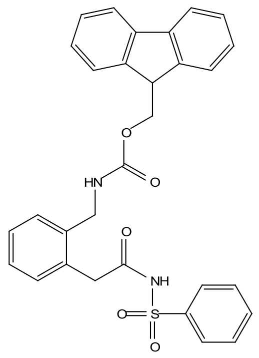

Compound XXV, ^{13}C{1H} NMR (126 MHz, $CDCl_3$) spectrum

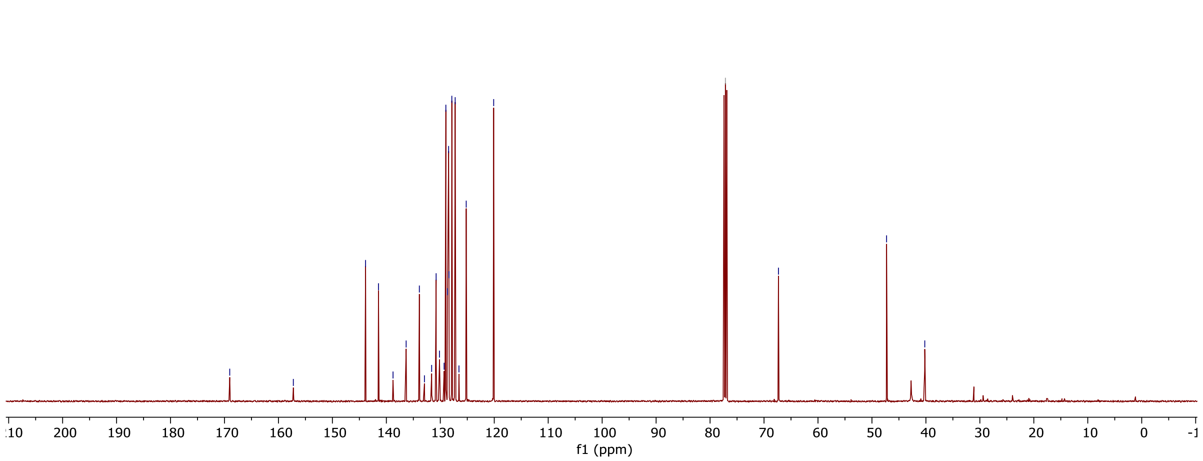

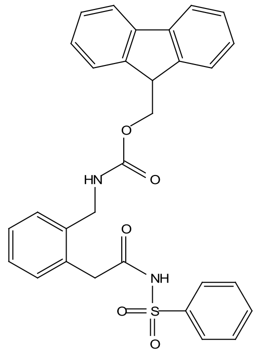

Characterization of Peptidomimetics 28-39 (LC-MS and ^{1}H NMR)

Compound 28, LC-MS

Name: 3-(2-(((4*R*,8*S*,11*S*)-11-amino-8-(4-hydroxybenzyl)-6,10-dioxo-1,2-dithia-5,9-diazacyclotridecane-4-carboxamido)methyl)phenyl)propanoic acid (TFA salt)

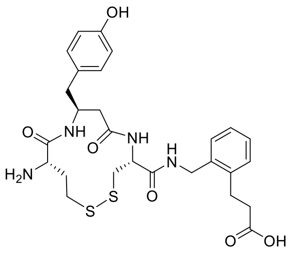

A)

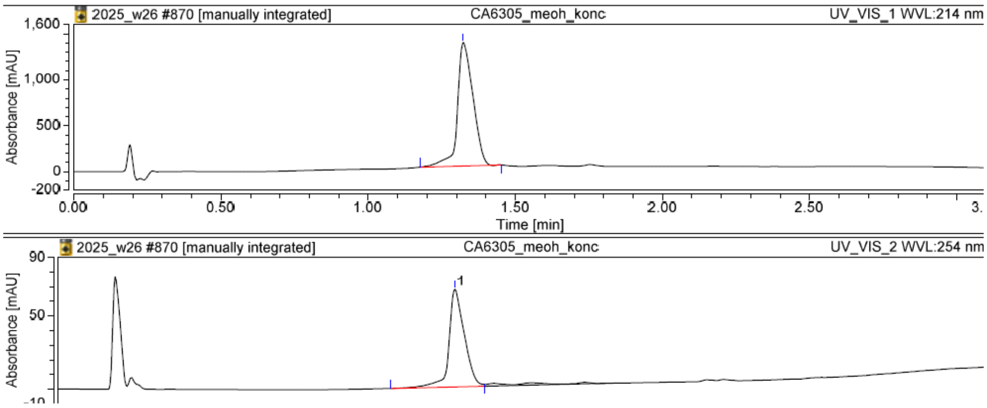

B)

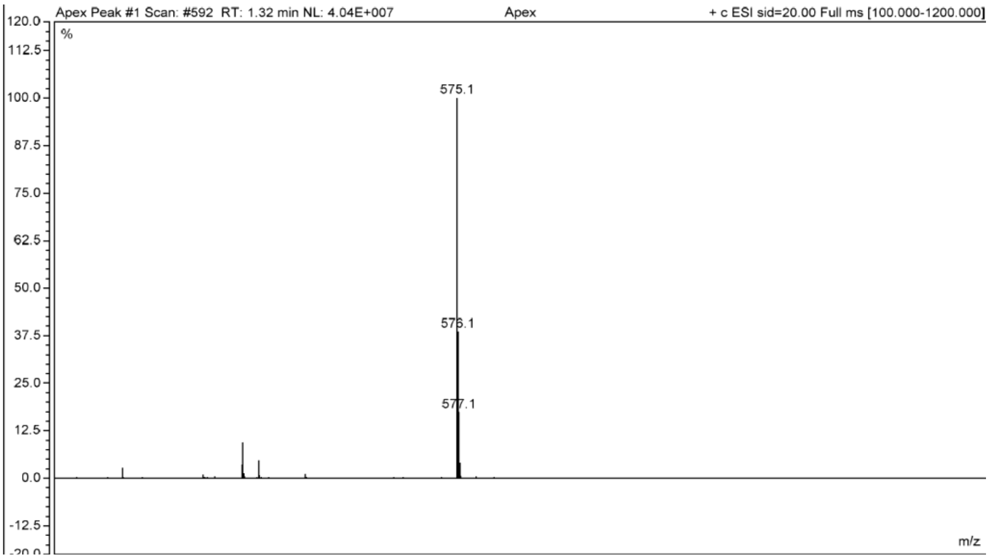

A) Analytical RP-HPLC of compound **28** on C18 column, acetonitrile gradient 5-100% of 0.05% formic acid in acetonitrile/water for 3 min. Top to bottom: UV detection at 214 and 254 nm. B) Low-resolution mass spectrum of **28**.

Compound 28, HRMS (ESI/TOF)

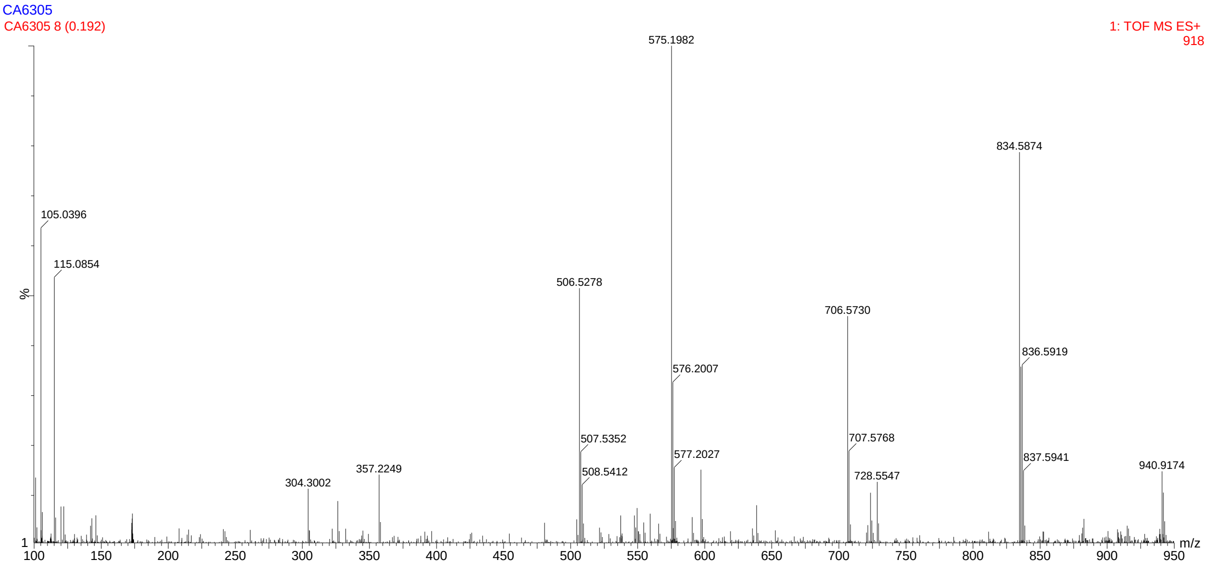

HRMS spectrum for **28**. m/z: $[M + H]^+$ Calcd for $C_{27}H_{35}N_4O_6S_2$ 575.1998; Found 575.1982.

Compound 28, ^{1}H NMR (400 MHz, DMSO-d_6) spectrum

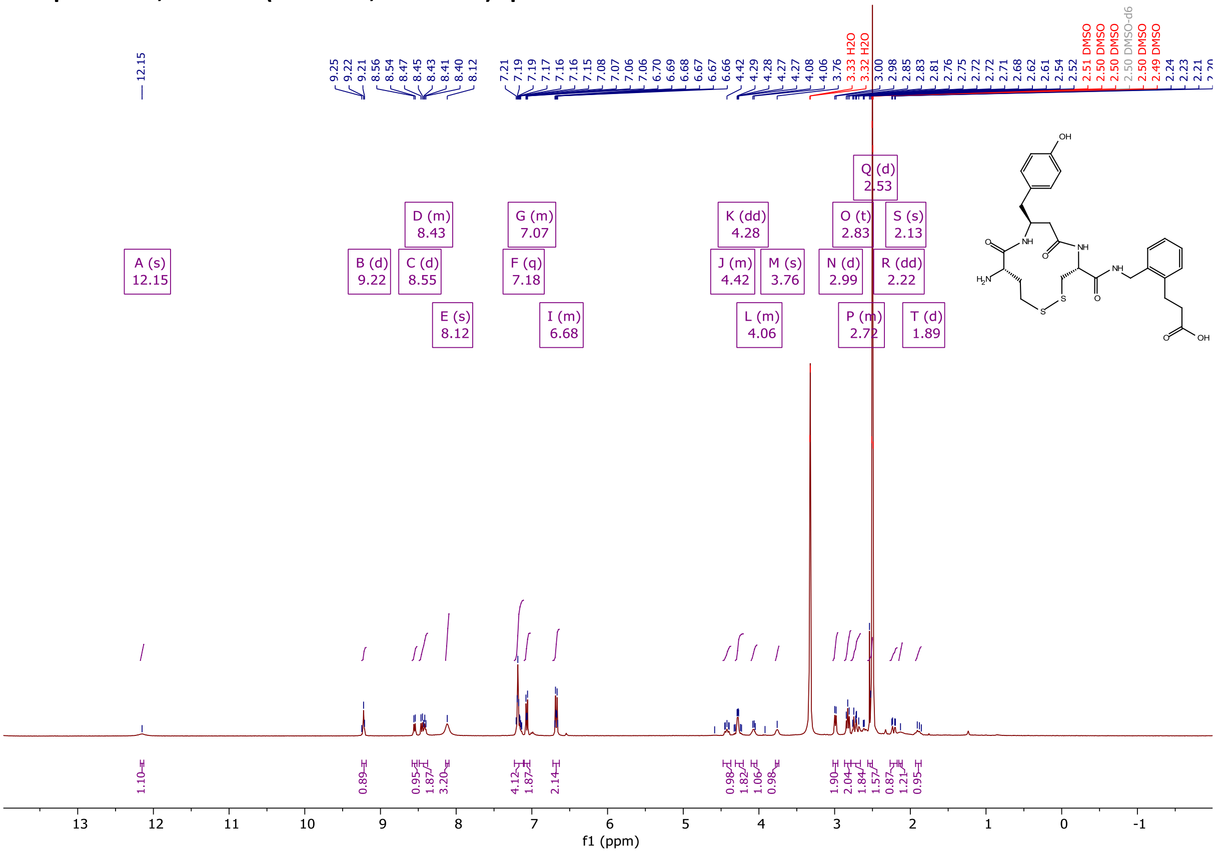

Compound 28, ^{19}F NMR (376 MHz, DMSO-d_6) spectrum

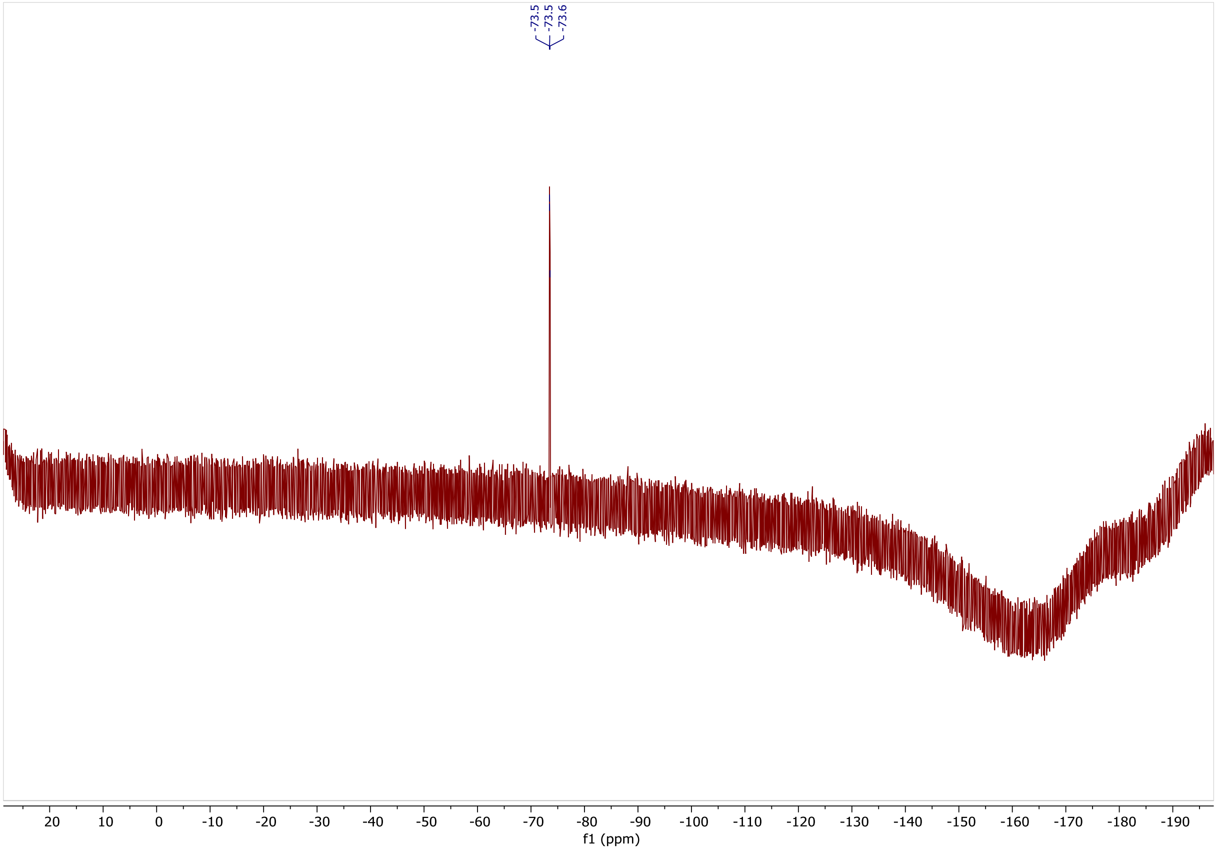

Compound 29, LC-MS

Name: 2-(2-(2-((4*R*,8*S*,11*S*)-11-amino-8-(4-hydroxybenzyl)-6,10-dioxo-1,2-dithia-5,9-diazacyclotridecane-4-carboxamido)ethyl)phenyl)acetic acid (TFA salt)

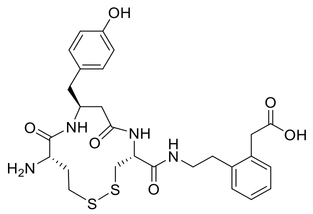

A)

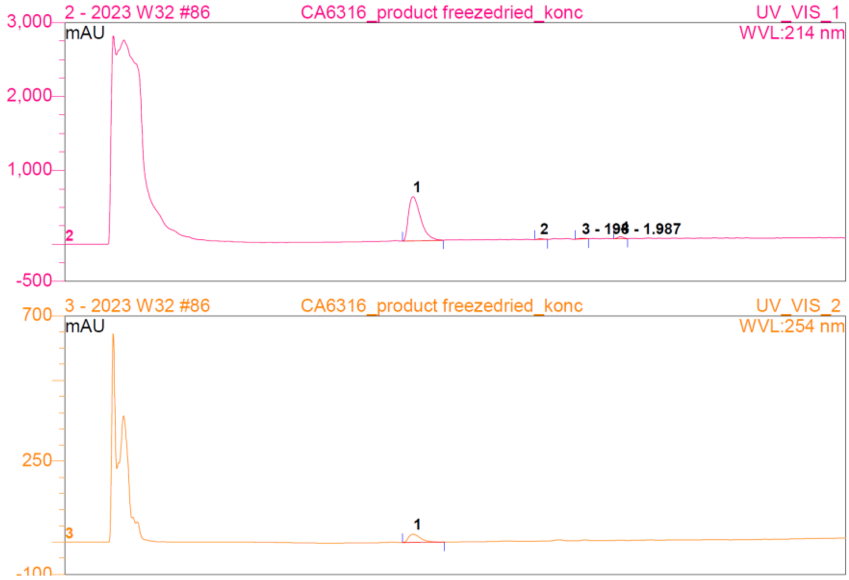

B)

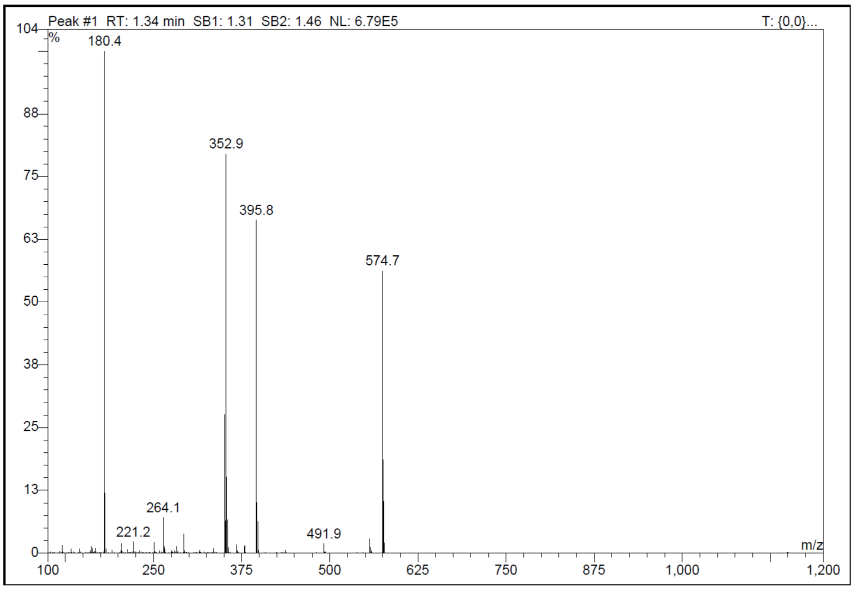

A) Analytical RP-HPLC of compound **29** on C18 column, acetonitrile gradient 5-100% of 0.05% formic acid in acetonitrile/water for 3 min. Top to bottom: UV detection at 214 and 254 nm. B) Low-resolution mass spectrum of **29**.

Compound 29, HRMS (ESI/TOF)

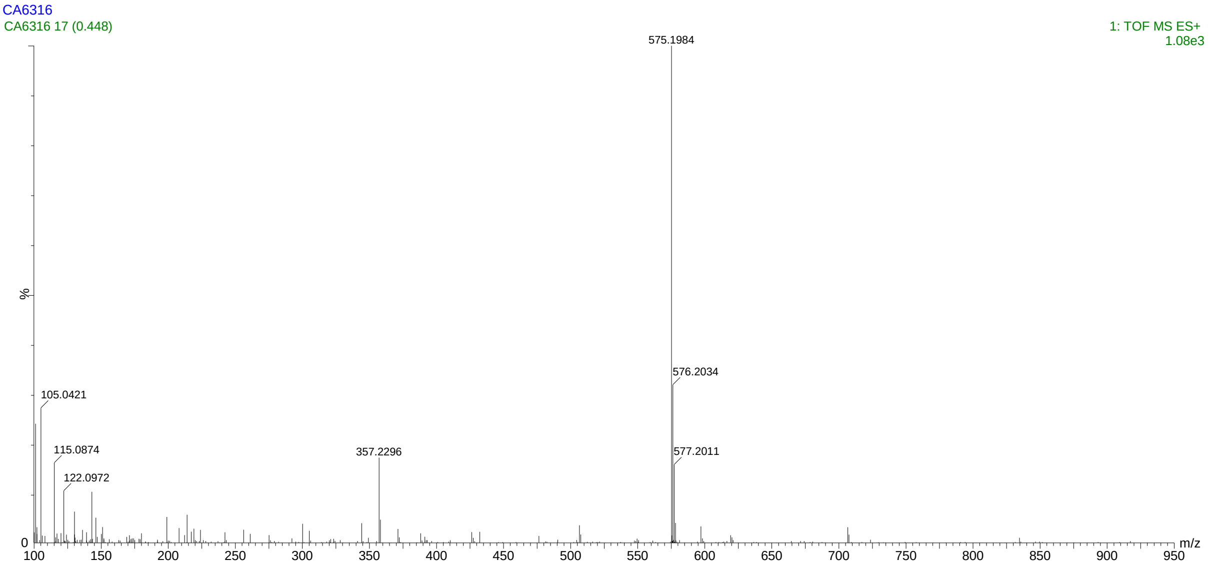

HRMS spectrum for **29**. m/z: $[M + H]^+$ Calcd for $C_{27}H_{35}N_4O_6S_2$ 575.1998; Found 575.1984

Compound 30, LC-MS

Name:2-(2-(((4*R*,8*S*,11*S*)-11-amino-8-(4-hydroxybenzyl)-6,10-dioxo-1,2-dithia-5,9-diazacyclotridecane-4-carboxamido)methyl)phenoxy)acetic acid (TFA salt)

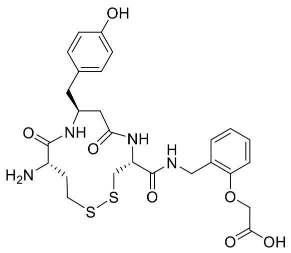

A)

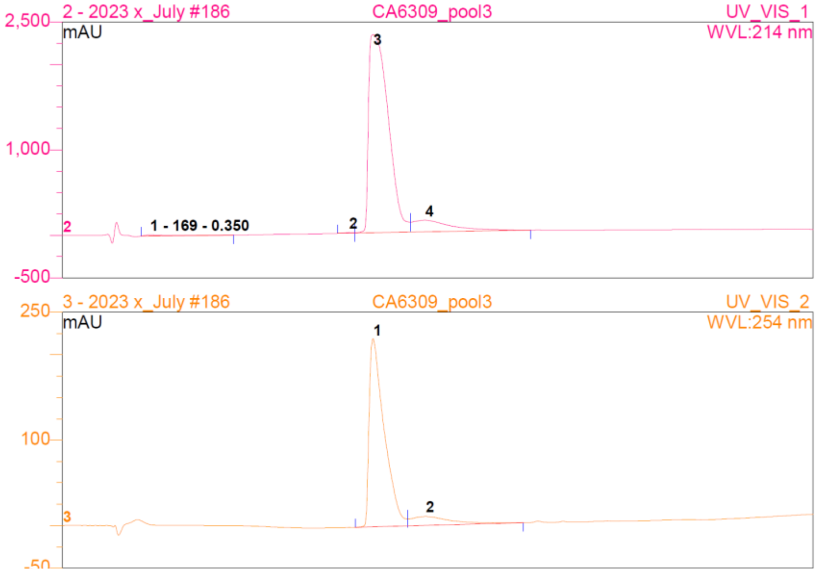

B)

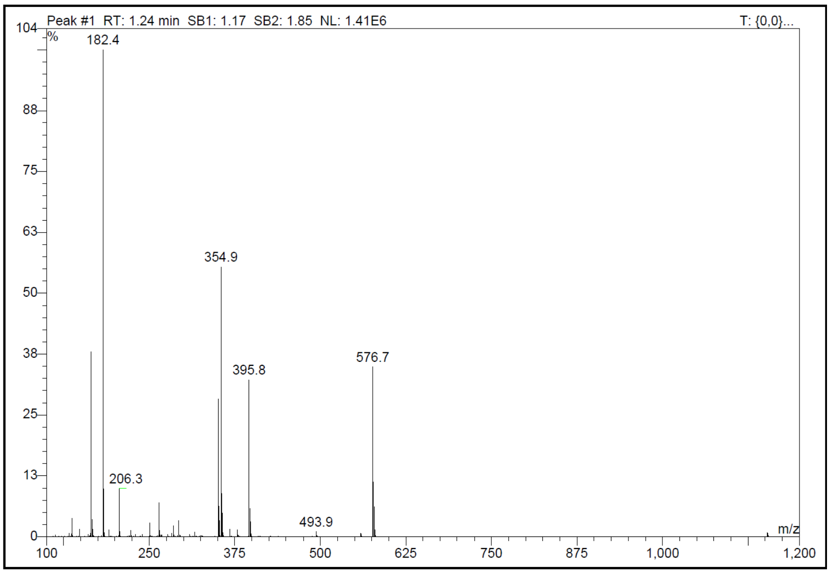

A) Analytical RP-HPLC of compound **30** on C18 column, acetonitrile gradient 5-100% of 0.05% formic acid in acetonitrile/water for 3 min. Top to bottom: UV detection at 214 and 254 nm. B) Low-resolution mass spectrum of **30**.

Compound 30, HRMS (ESI/TOF)

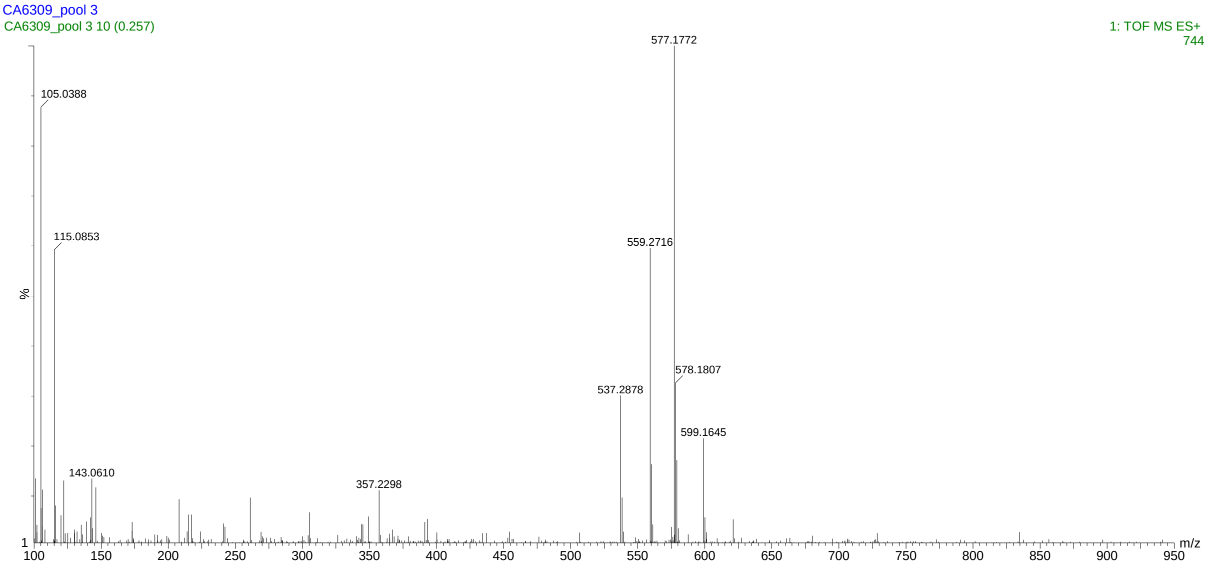

HRMS spectrum for **30**. m/z: $[M + H]^+$ Calcd for $C_{26}H_{33}N_4O_7S_2$ 571.1791; Found 577.1782

Compound 30, ^{1}H NMR (400 MHz, DMSO-d_6) spectrum

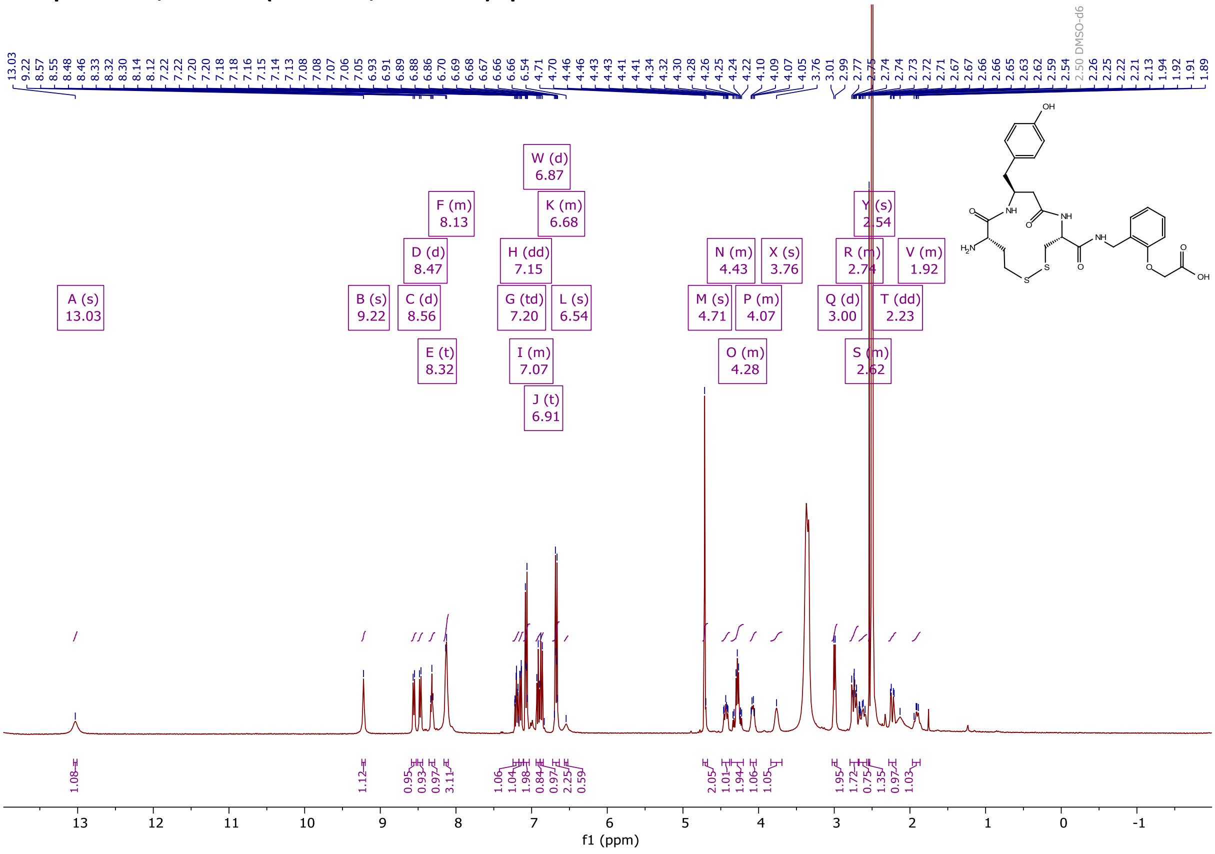

Compound 31, LC-MS

Name: 2-(3-(((4*R*,8*S*,11*S*)-11-amino-8-(4-hydroxybenzyl)-6,10-dioxo-1,2-dithia-5,9-diazacyclotridecane-4-carboxamido)methyl)phenoxy)acetic acid (TFA salt)

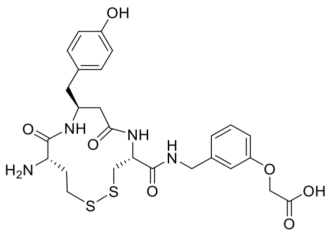

A)

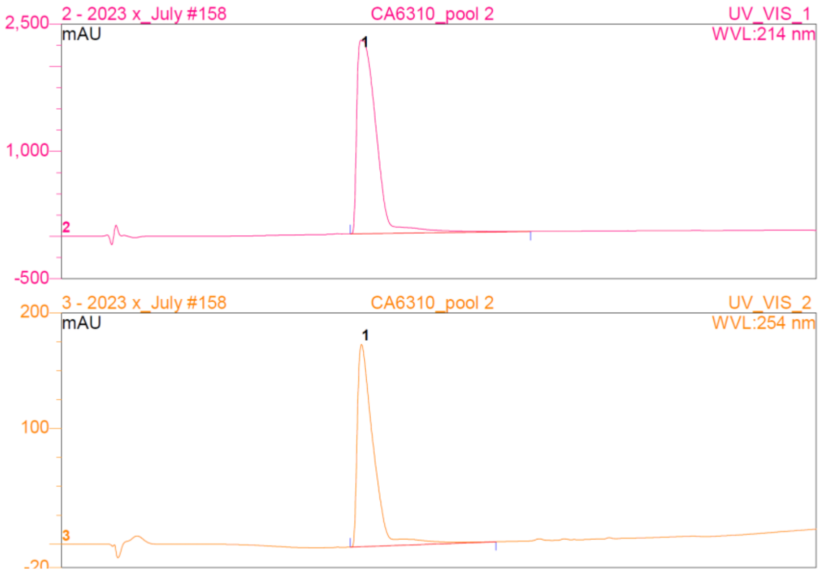

B)

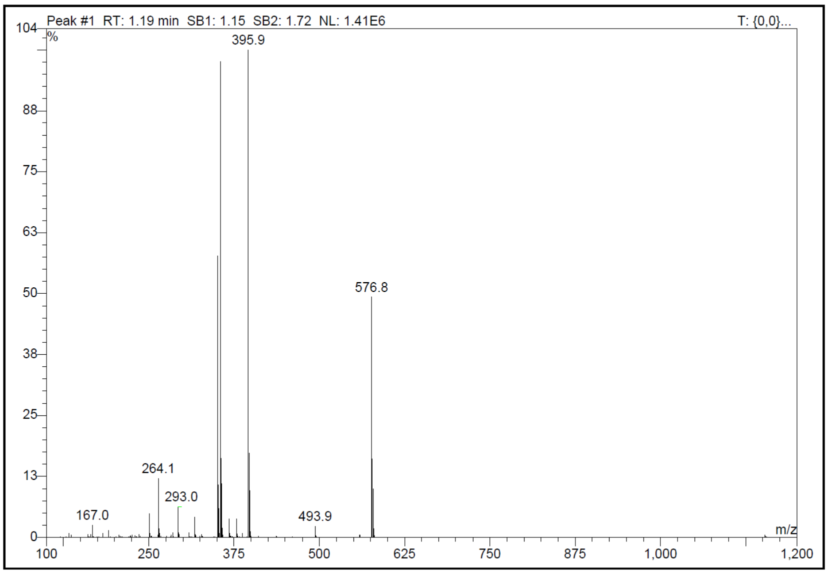

A) Analytical RP-HPLC of compound **31** on C18 column, acetonitrile gradient 5-100% of 0.05% formic acid in acetonitrile/water for 3 min. Top to bottom: UV detection at 214 and 254 nm. B) Low-resolution mass spectrum of **31**.

Compound 31, HRMS (ESI/TOF)

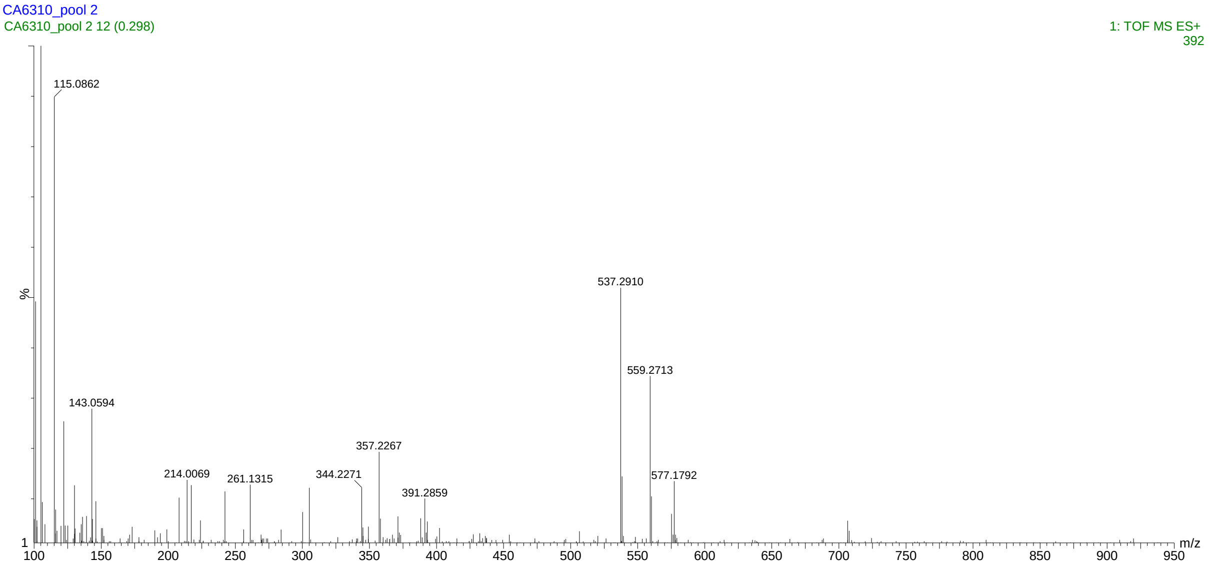

HRMS spectrum for **31**. m/z: [M + H]+ Calcd for $C_{26}H_{33}N_4O_7S_2$ 571.1791; Found 577.1792

Compound 31, ^{1}H NMR (400 MHz, DMSO-d_6) spectrum

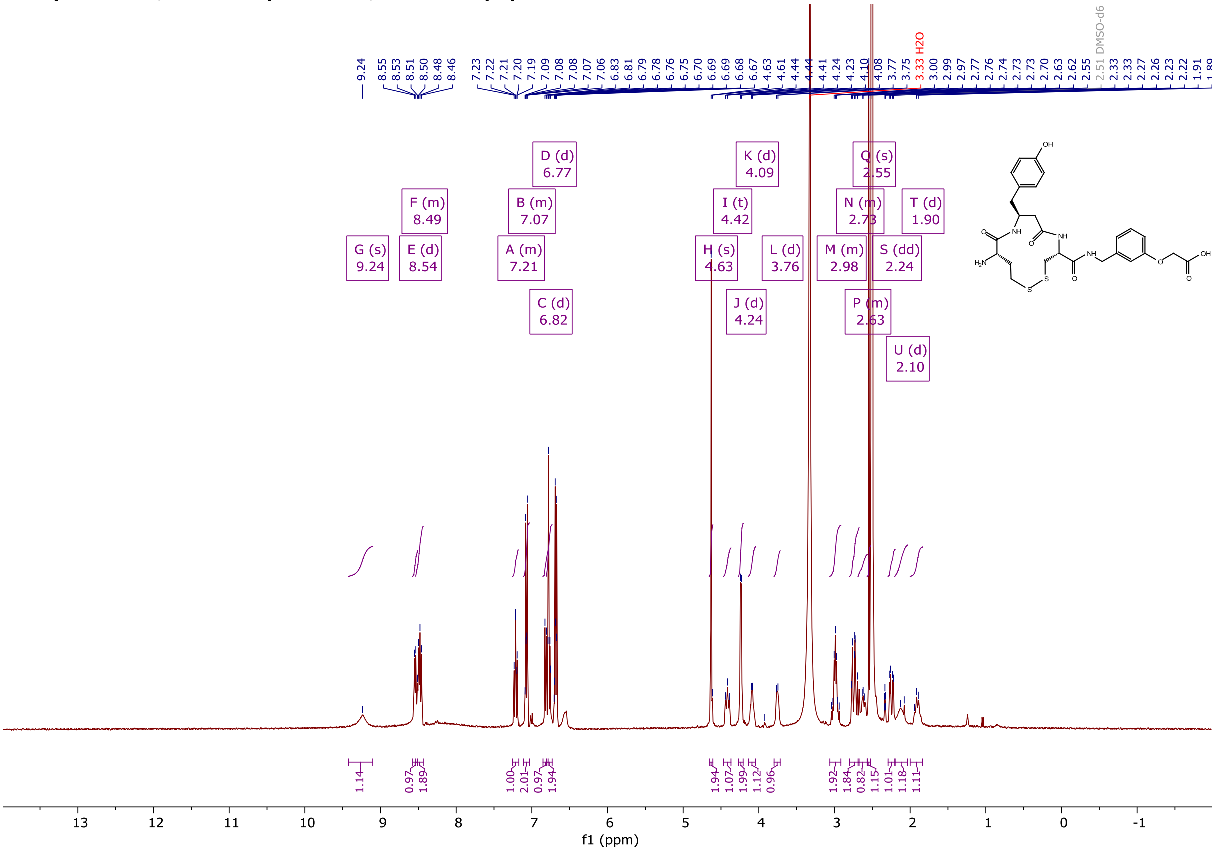

Compound 32, LC-MS

Name: (4*R*,8*S*,11*S*)-11-amino-8-(4-hydroxybenzyl)-*N*-(2-(2-(methylamino)-2-oxoethyl)benzyl)-6,10-dioxo-1,2-dithia-5,9-diazacyclotridecane-4-carboxamide (TFA salt)

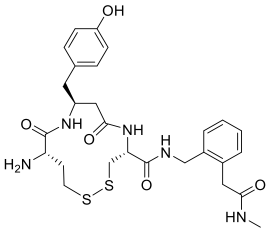

A)

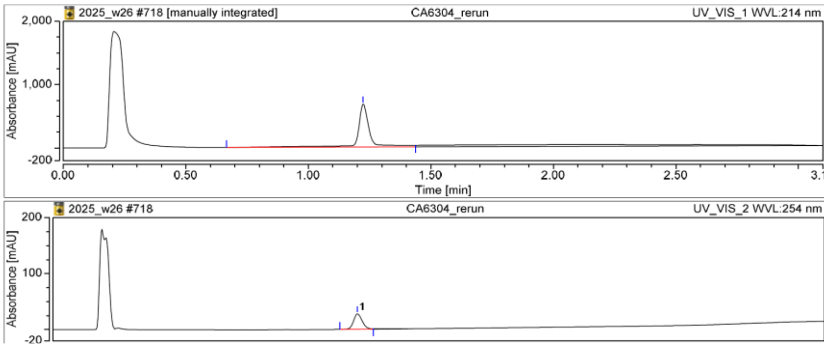

B)

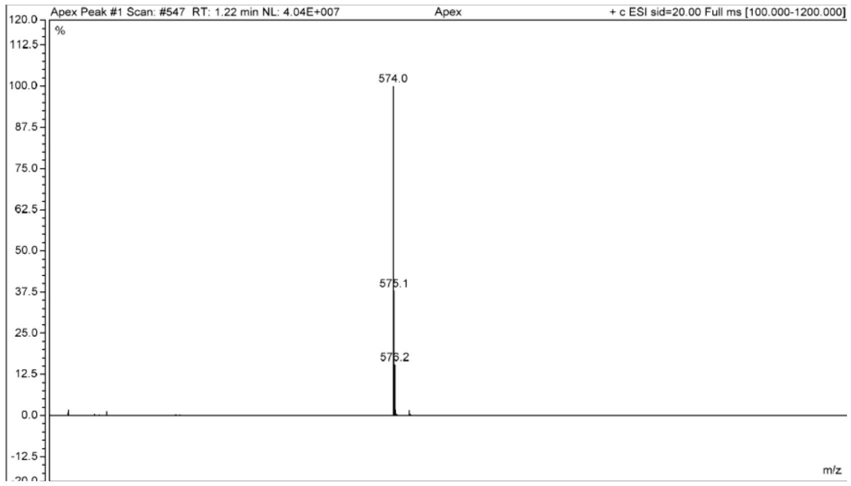

A) Analytical RP-HPLC of compound **32** on C18 column, acetonitrile gradient 5-100% of 0.05% formic acid in acetonitrile/water for 3 min. Top to bottom: UV detection at 214 and 254 nm. B) Low-resolution mass spectrum of **32**.

Compound 32, HRMS (ESI/TOF)

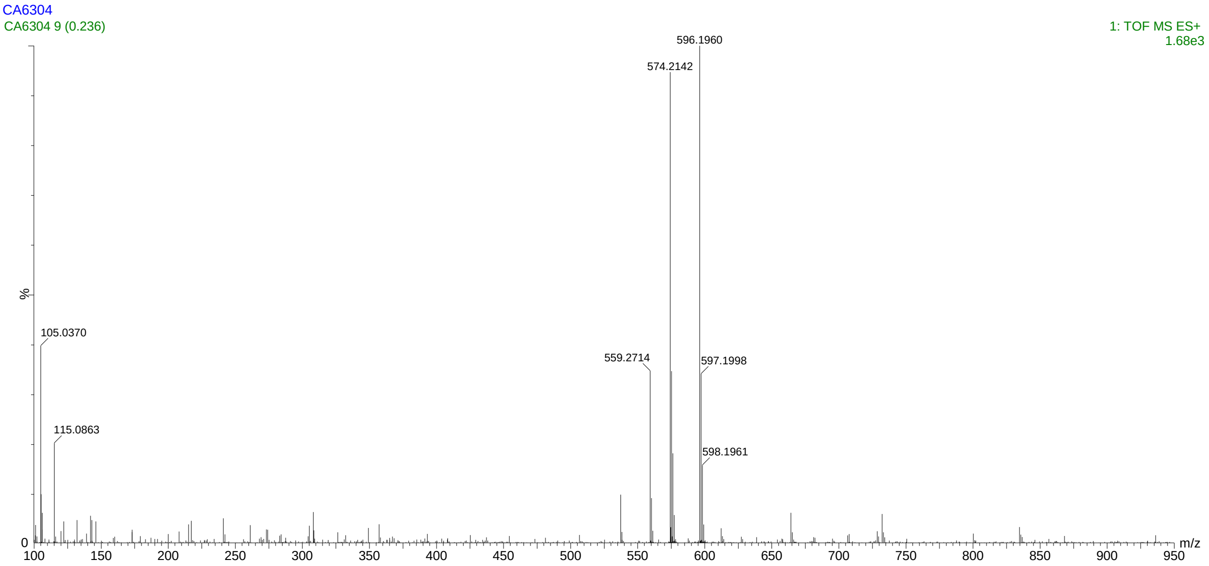

HRMS spectrum for **32**. m/z: $[M + H]^+$ Calcd for $C_{27}H_{36}N_5O_5S_2$ 574.2158; Found 574.2142.

Compound 32, ^{1}H NMR (400 MHz, DMSO-d_6) spectrum

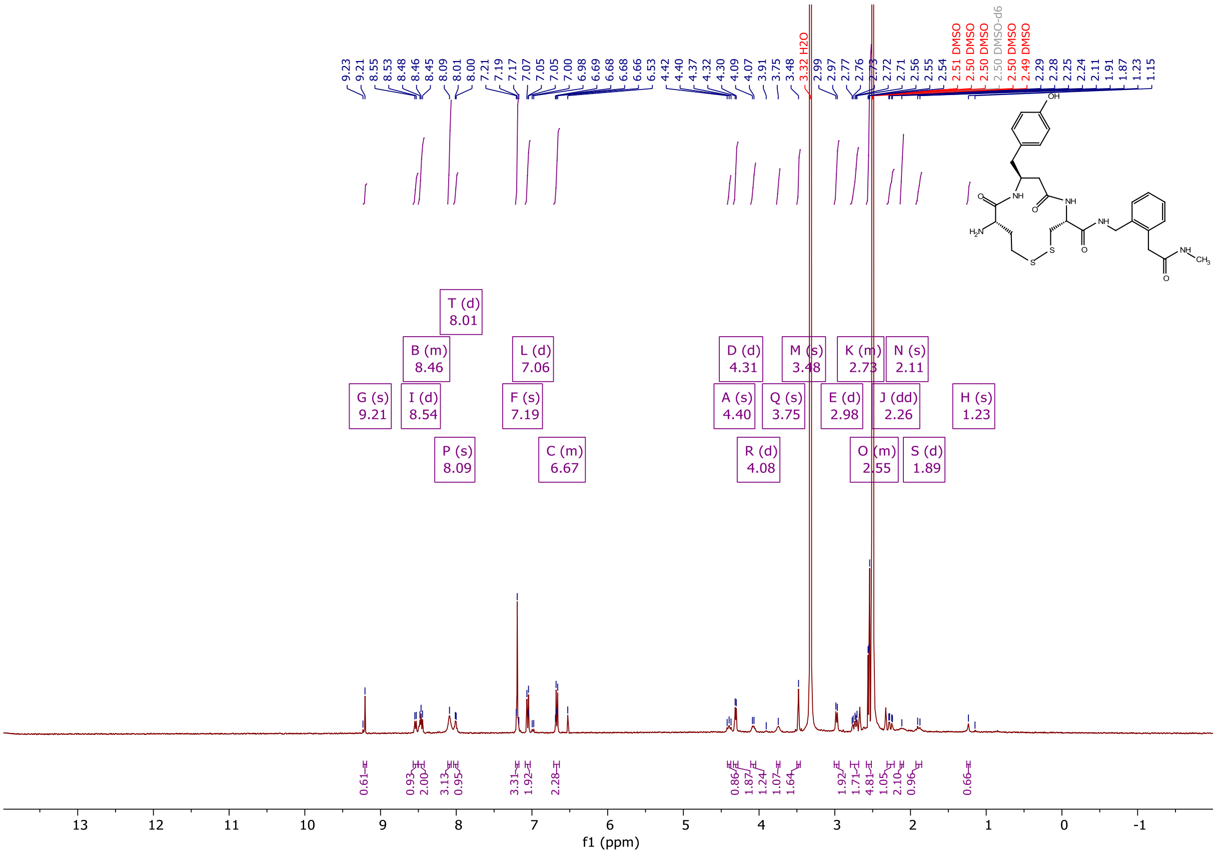

Compound 33, LC-MS

Name: (4*R*,8*S*,11*S*)-11-amino-*N*-(2-(2-(benzylamino)-2-oxoethyl)benzyl)-8-(4-hydroxybenzyl)-6,10-dioxo-1,2-dithia-5,9-diazacyclotridecane-4-carboxamide (TFA salt)

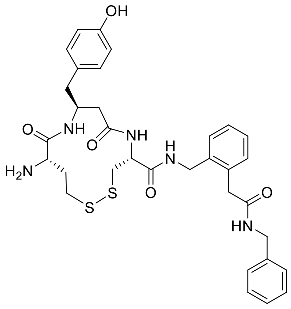

A)

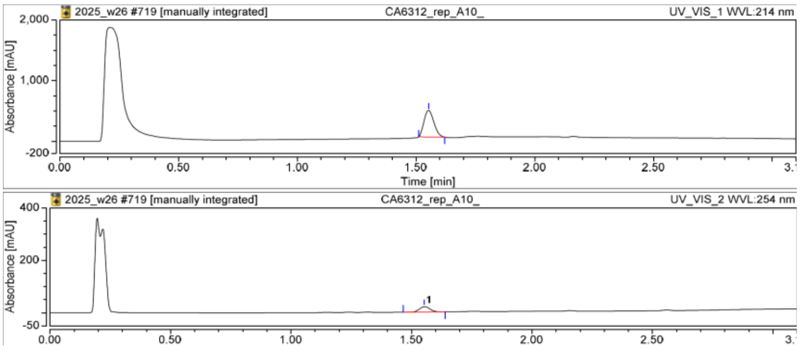

B)

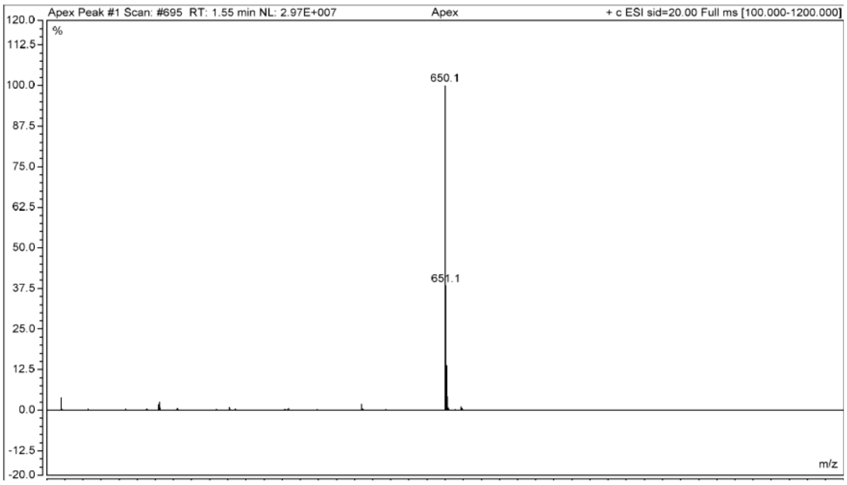

A) Analytical RP-HPLC of compound **33** on C18 column, acetonitrile gradient 5-100% of 0.05% formic acid in acetonitrile/water for 3 min. Top to bottom: UV detection at 214 and 254 nm. B) Low-resolution mass spectrum of **33**.

Compound 33, HRMS (ESI/TOF)

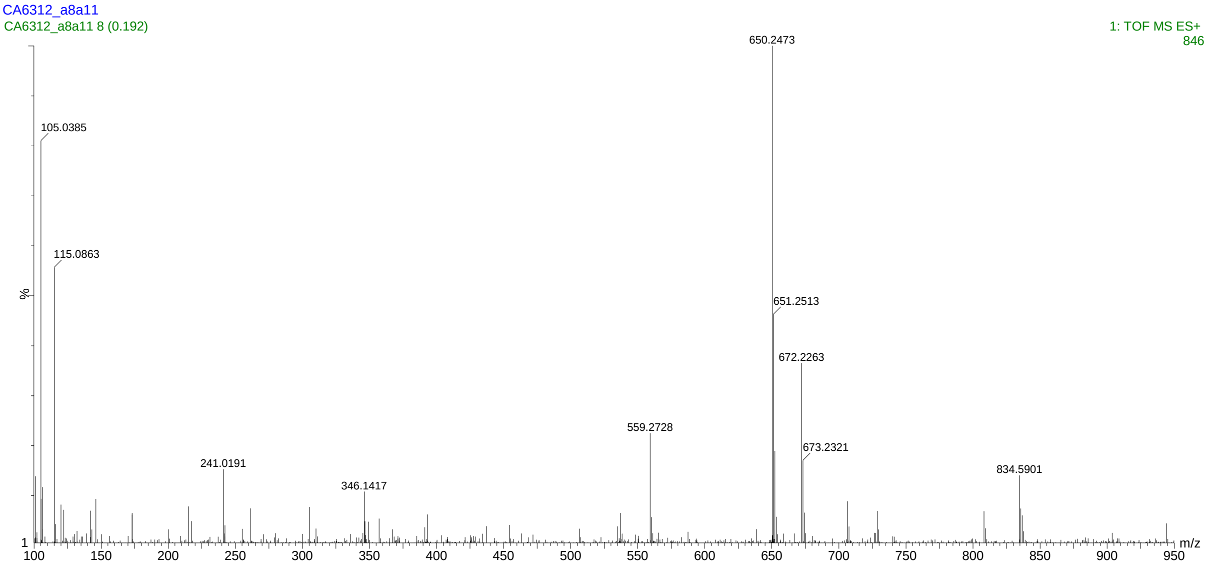

HRMS spectrum for **33**. m/z: $[M + H]^+$ Calcd for $C_{33}H_{40}N_5O_5S_2$ 650.2471; Found 650.2473.

Compound 34, LC-MS

Name: (4*R*,8*S*,11*S*)-11-amino-8-(4-hydroxybenzyl)-*N*-(2-(hydroxymethyl)benzyl)-6,10-dioxo-1,2-dithia-5,9-diazacyclotride-cane-4-carboxamide (TFA salt)

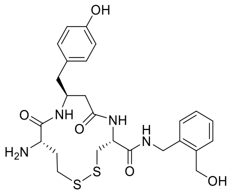

A)

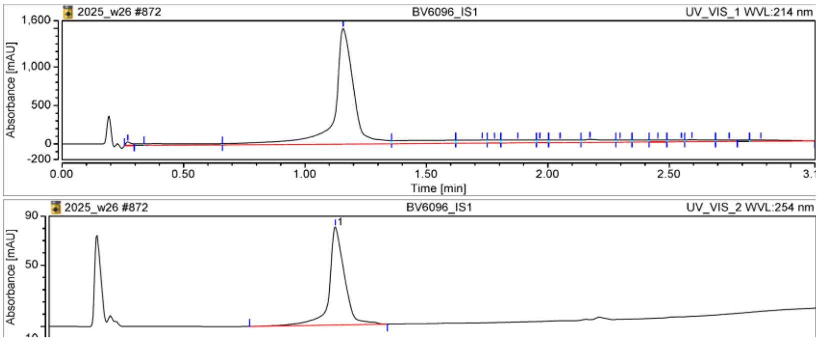

B)

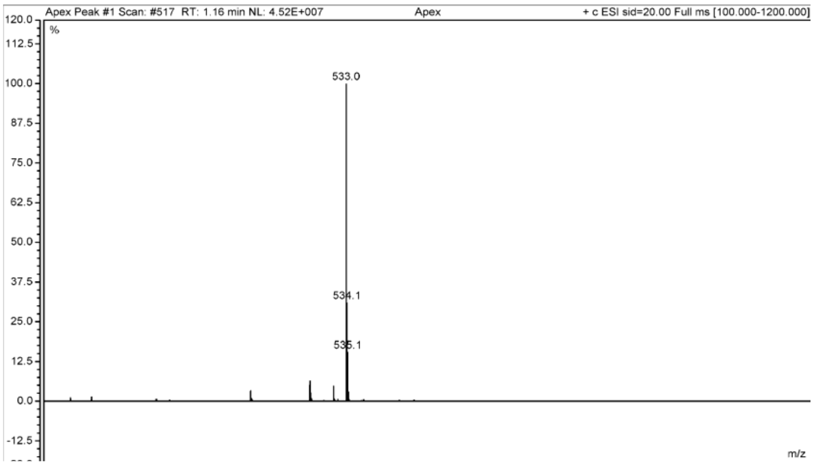

A) Analytical RP-HPLC of compound **34** on C18 column, acetonitrile gradient 5-100% of 0.05% formic acid in acetonitrile/water for 3 min. Top to bottom: UV detection at 214 and 254 nm. B) Low-resolution mass spectrum of **34**.

Compound 34, HRMS (ESI/TOF)

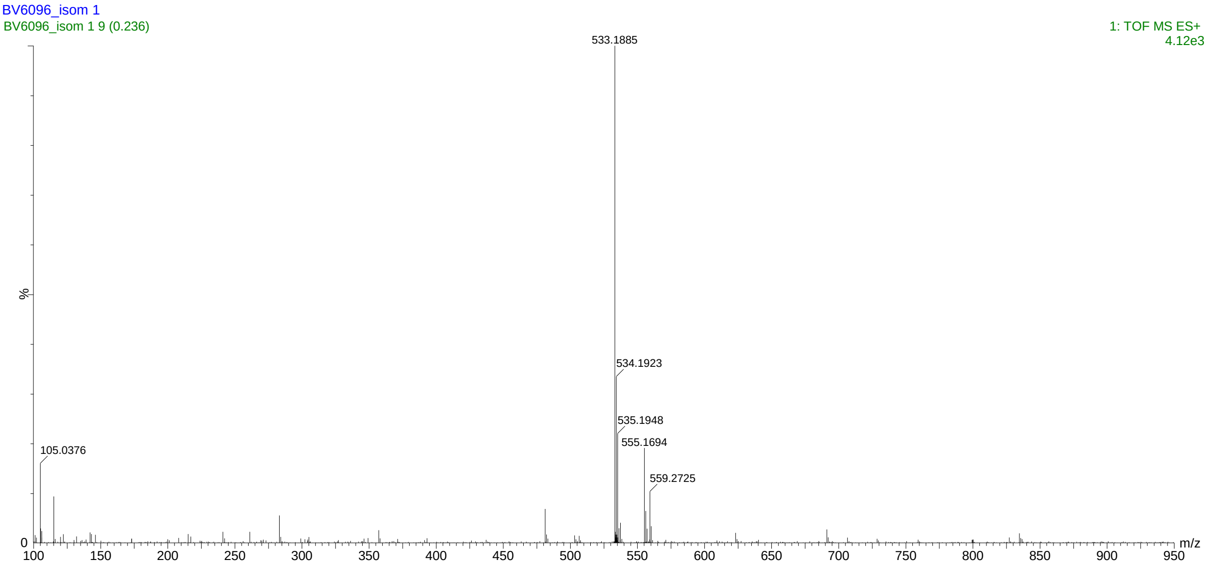

HRMS spectrum for **34**. m/z: $[M + H]^+$ Calcd for $C_{25}H_{33}N_4O_5S_2$ 533.1892; Found 533.1885.

Compound 34, ^{1}H NMR (400 MHz, DMSO-d_6) spectrum

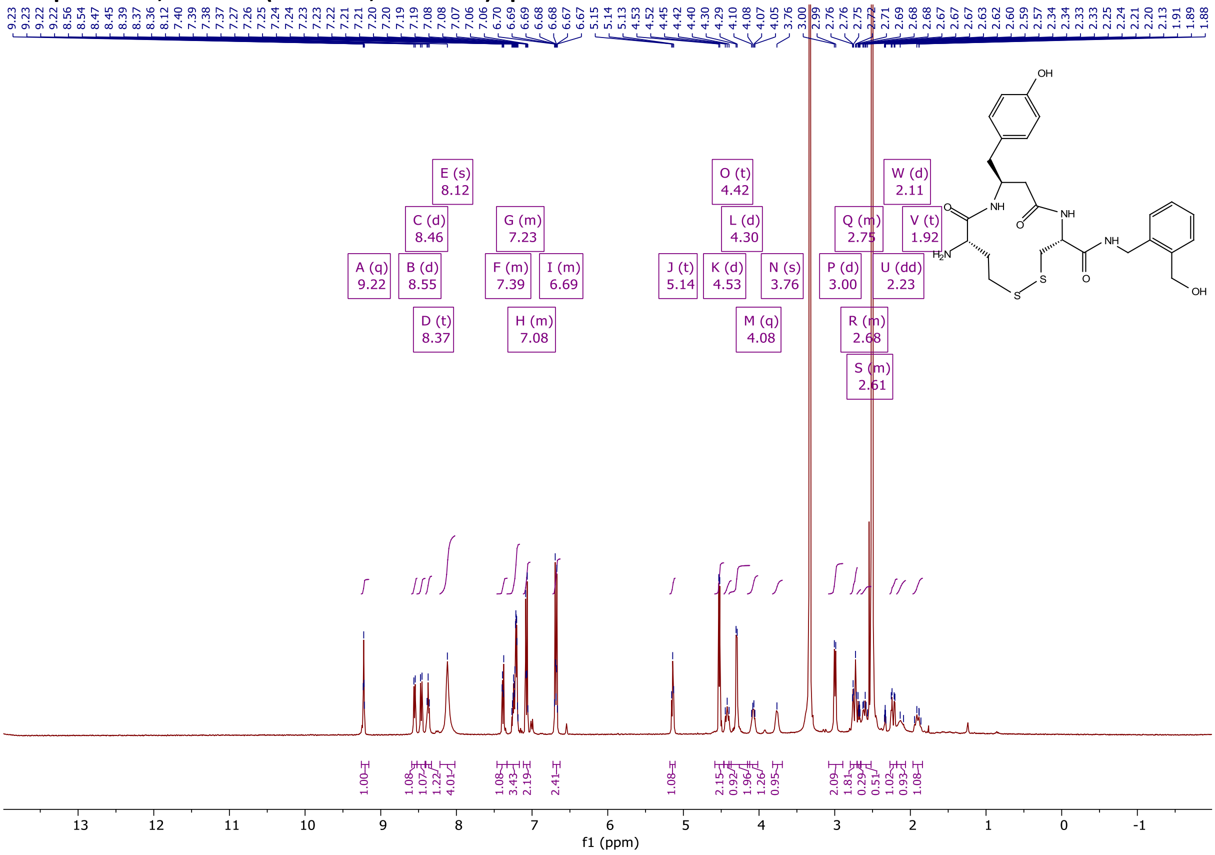

Compound 35, LC-MS

Name: 3-((4*R*,8*S*,11*S*)-11-amino-8-(4-hydroxybenzyl)-6,10-dioxo-1,2-dithia-5,9-diazacyclotridecane-4-carboxamido)cyclo-hexane-1-carboxylic acid (TFA salt)

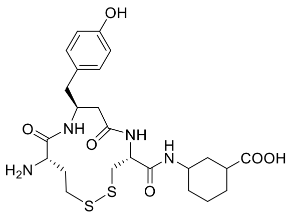

A)

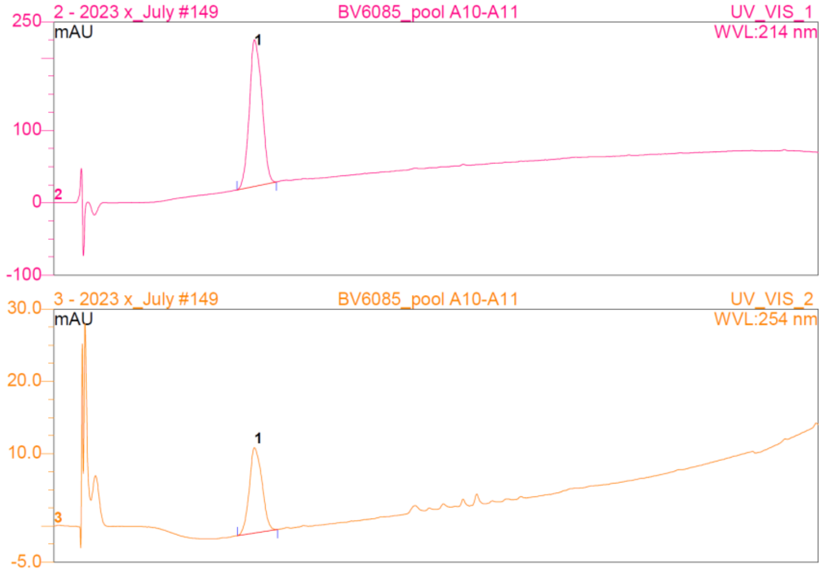

B)

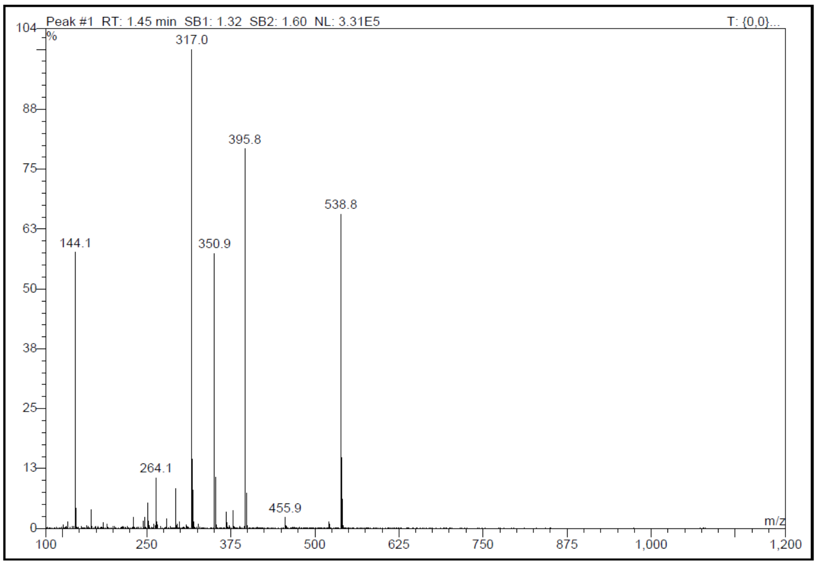

A) Analytical RP-HPLC of compound **35** on C18 column, acetonitrile gradient 5-100% of 0.05% formic acid in acetonitrile/water for 3 min. Top to bottom: UV detection at 214 and 254 nm. B) Low-resolution mass spectrum of **35**.

Compound 35, HRMS (ESI/TOF)

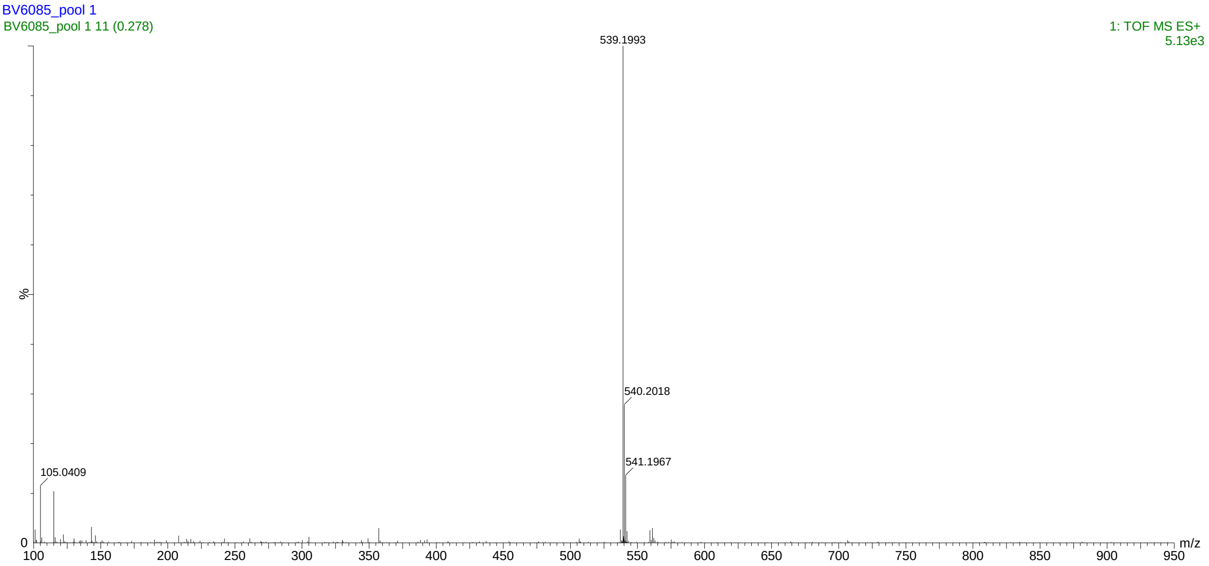

HRMS spectrum for **35**. m/z: $[M + H]^+$ Calcd for $C_{24}H_{35}N_4O_6S_2$ 539.1998; Found 539.1993.

Compound 35, ^1H NMR (400 MHz, DMSO-d_6) spectrum

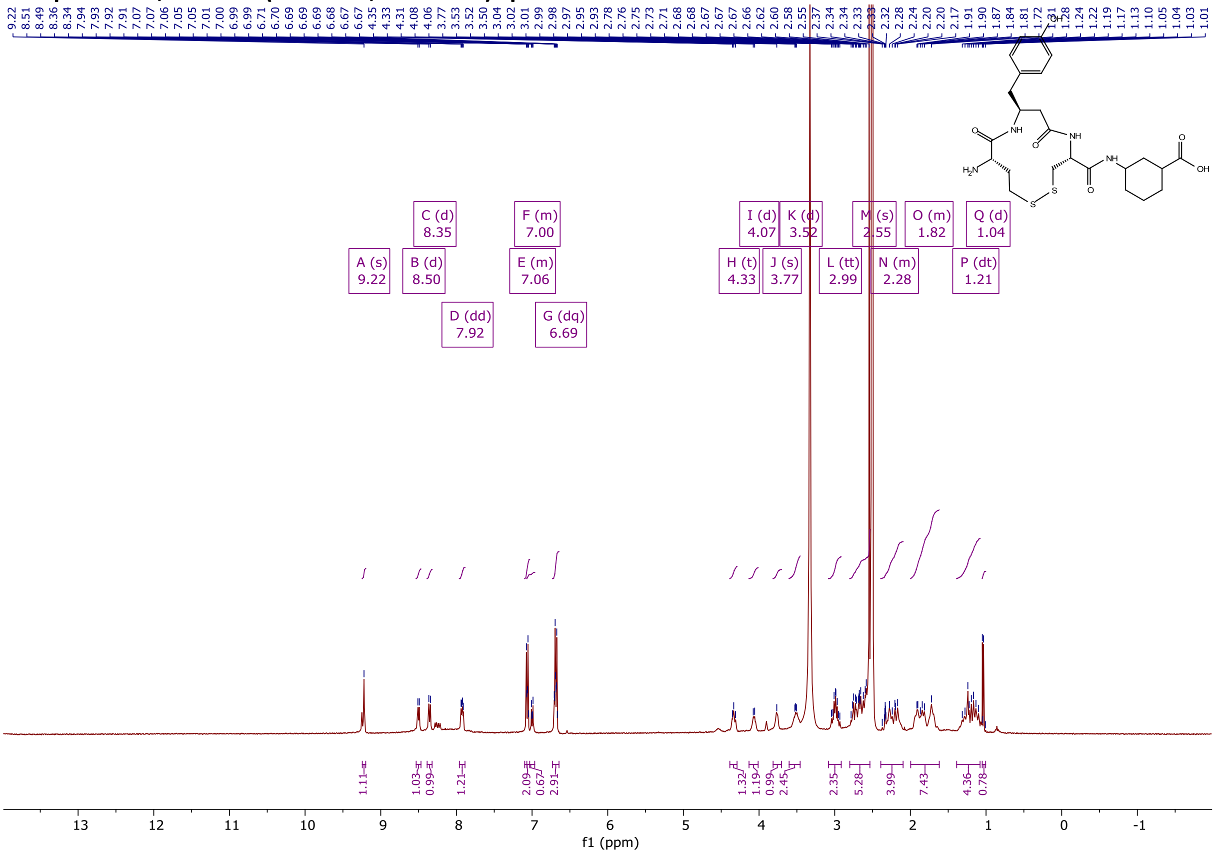

Compound 36, LC-MS

Name: 5-((4*R*,8*S*,11*S*)-11-amino-8-(4-hydroxybenzyl)-6,10-dioxo-1,2-dithia-5,9-diazacyclotridecane-4-carboxamido)pentanoic acid (TFA salt)

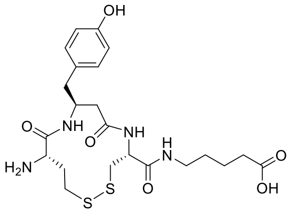

A)

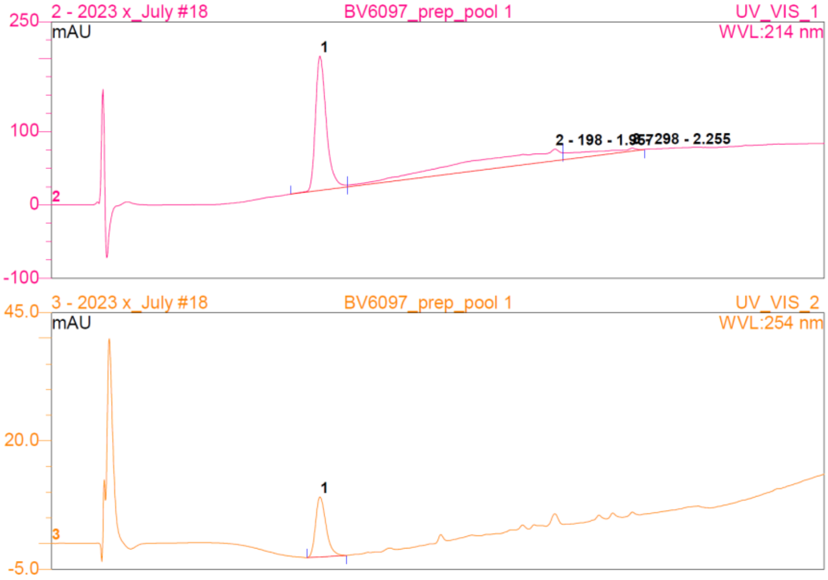

B)

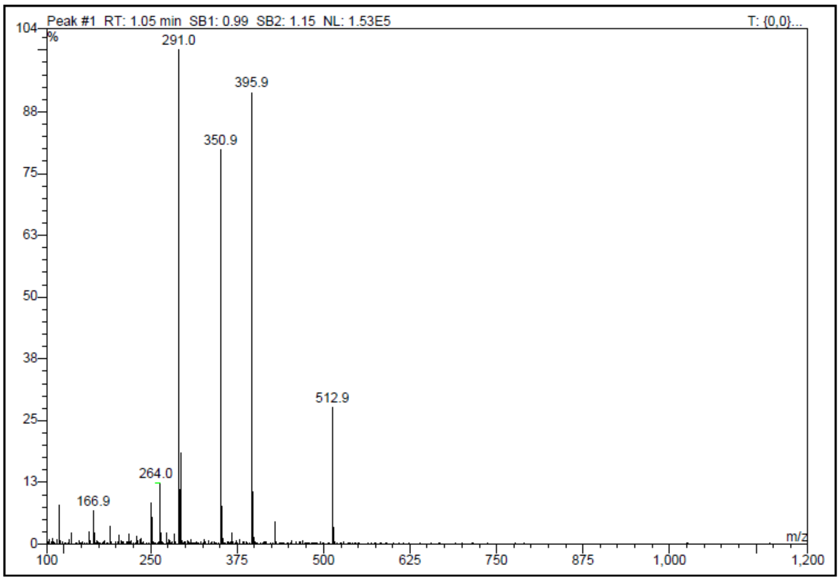

A) Analytical RP-HPLC of compound **36** on C18 column, acetonitrile gradient 5-100% of 0.05% formic acid in acetonitrile/water for 3 min. Top to bottom: UV detection at 214 and 254 nm. B) Low-resolution mass spectrum of **36**.

Compound 36, HRMS (ESI/TOF)

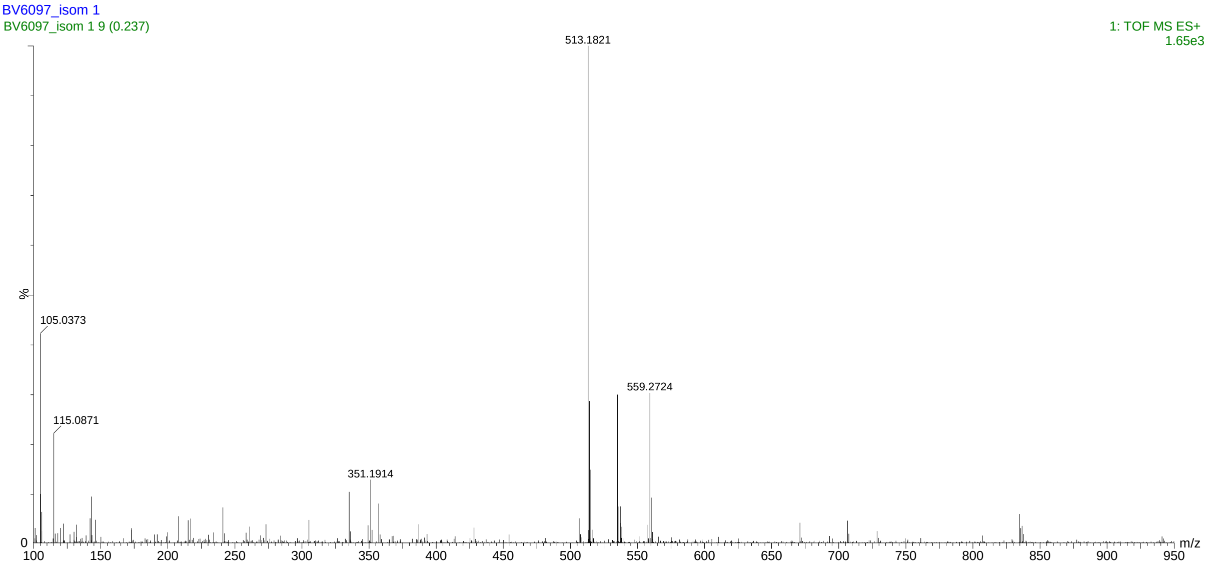

HRMS spectrum for **36**. m/z: $[M + H]^+$ Calcd for $C_{22}H_{33}N_4O_6S_2$ 513.1842; Found 513.1821.

Compound 36, ^1H NMR (400 MHz, DMSO-d_6) spectrum

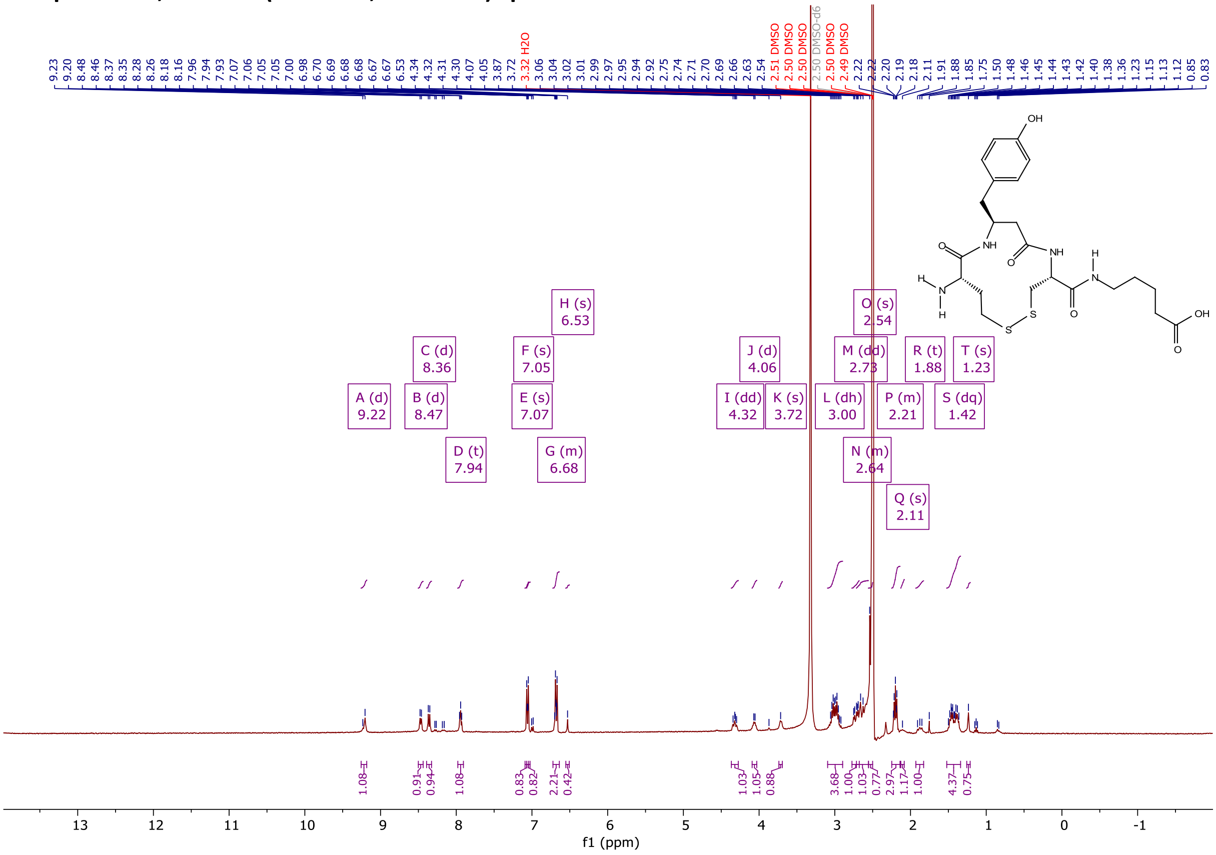

Compound 37, LC-MS

Name: 2-(((4*R*,8*S*,11*S*)-11-amino-8-(4-hydroxybenzyl)-6,10-dioxo-1,2-dithia-5,9-diazacyclotridecane-4-carboxamido)methyl)benzoic acid (TFA salt)

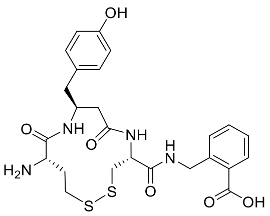

A)

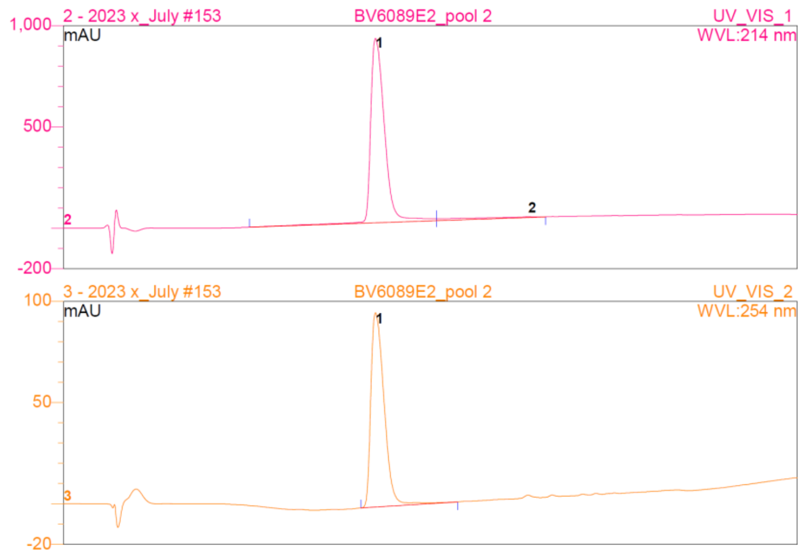

B)

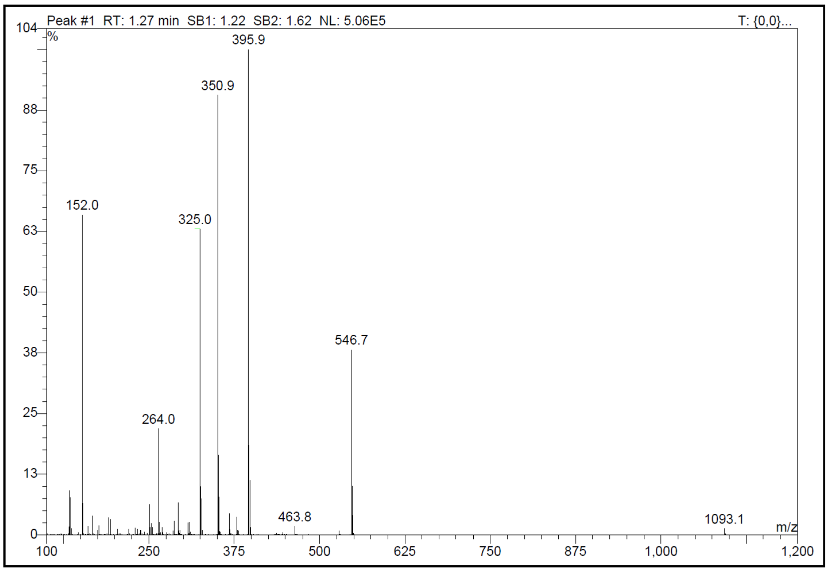

A) Analytical RP-HPLC of compound **37** on C18 column, acetonitrile gradient 5-100% of 0.05% formic acid in acetonitrile/water for 3 min. Top to bottom: UV detection at 214 and 254 nm. B) Low-resolution mass spectrum of **37**.

Compound 37, HRMS (ESI/TOF)

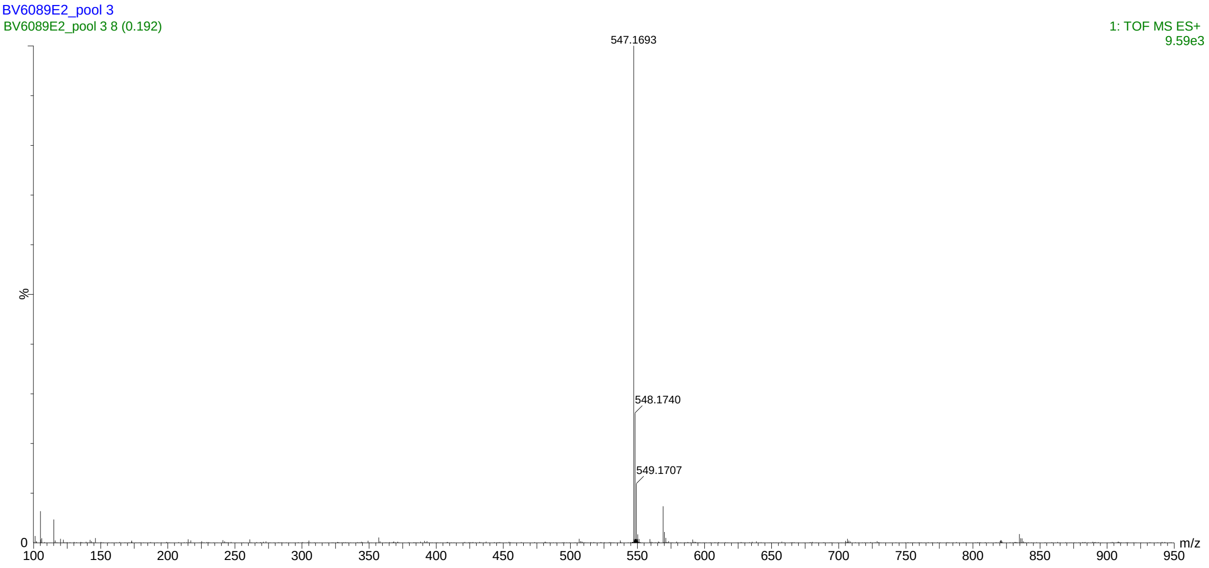

HRMS spectrum for **37**. m/z: $[M + H]^+$ Calcd for $C_{25}H_{31}N_4O_6S_2$ 547.1685; Found 547.1693.

Compound 37, ^{1}H NMR (400 MHz, DMSO-d_6) spectrum

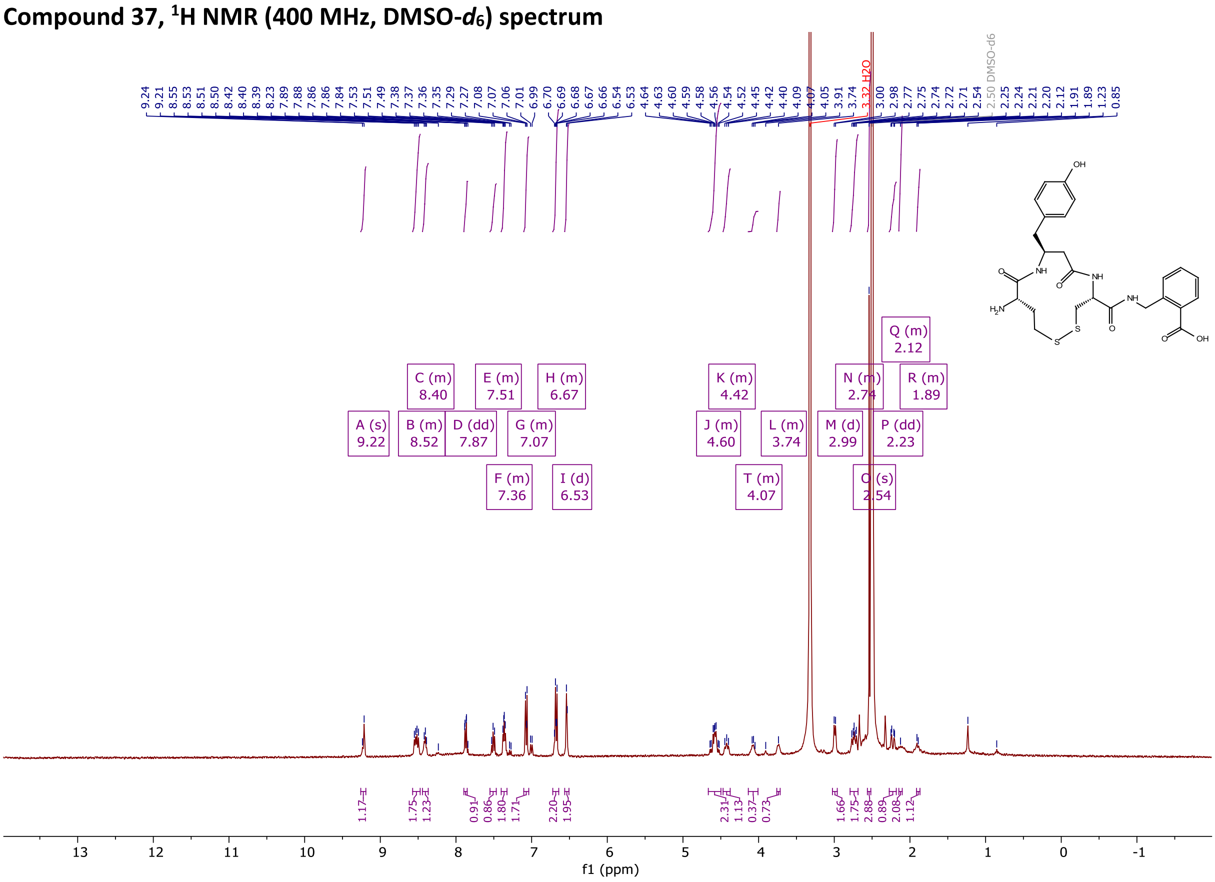

Compound 38, LC-MS

Name: 3-(((4*R*,8*S*,11*S*)-11-amino-8-(4-hydroxybenzyl)-6,10-dioxo-1,2-dithia-5,9-diazacyclotridecane-4-carboxamido)me-thyl)benzoic acid (TFA salt)

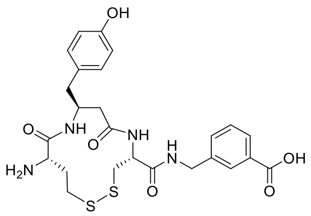

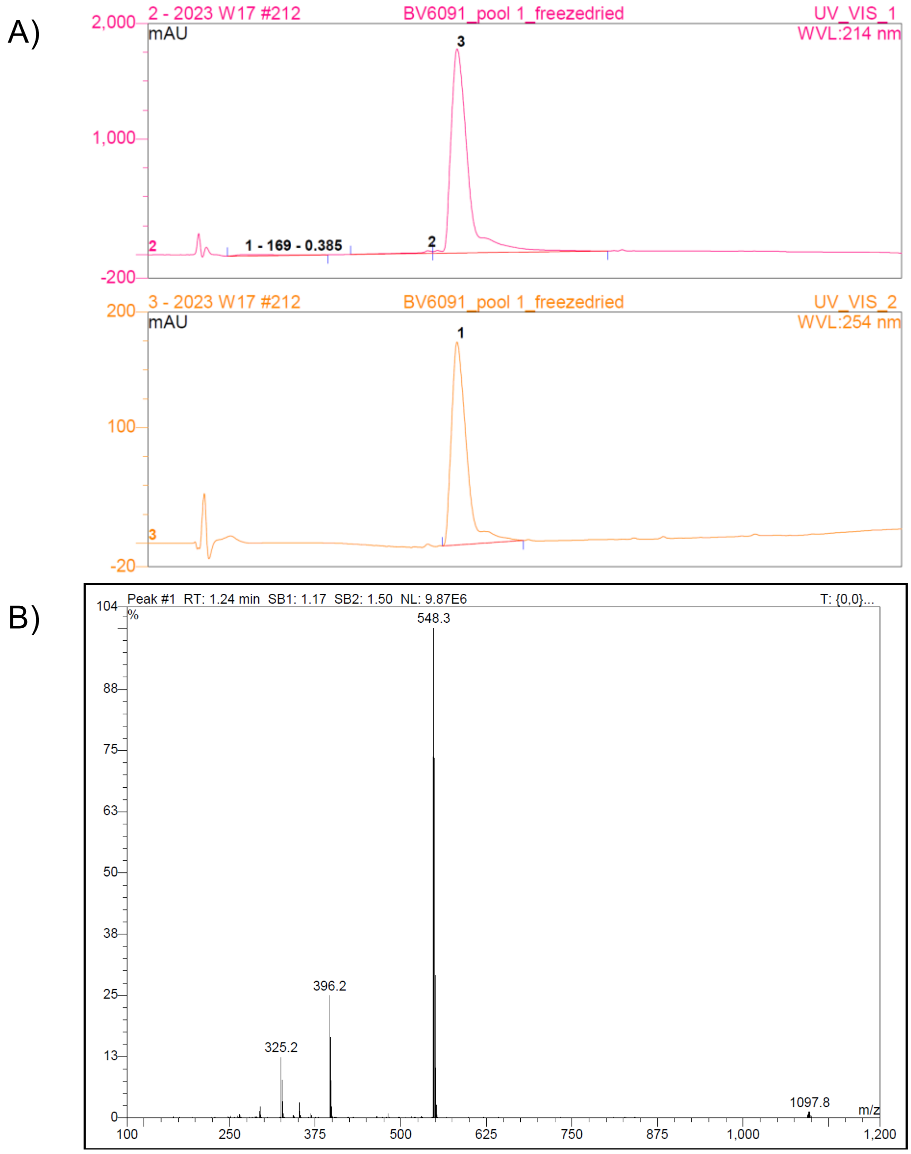

A) Analytical RP-HPLC of compound **38** on C18 column, acetonitrile gradient 5-100% of 0.05% formic acid in acetoni-trile/water for 3 min. Top to bottom: UV detection at 214 and 254 nm. B) Low-resolution mass spectrum of **38**.

Compound 38, HRMS (ESI/TOF)

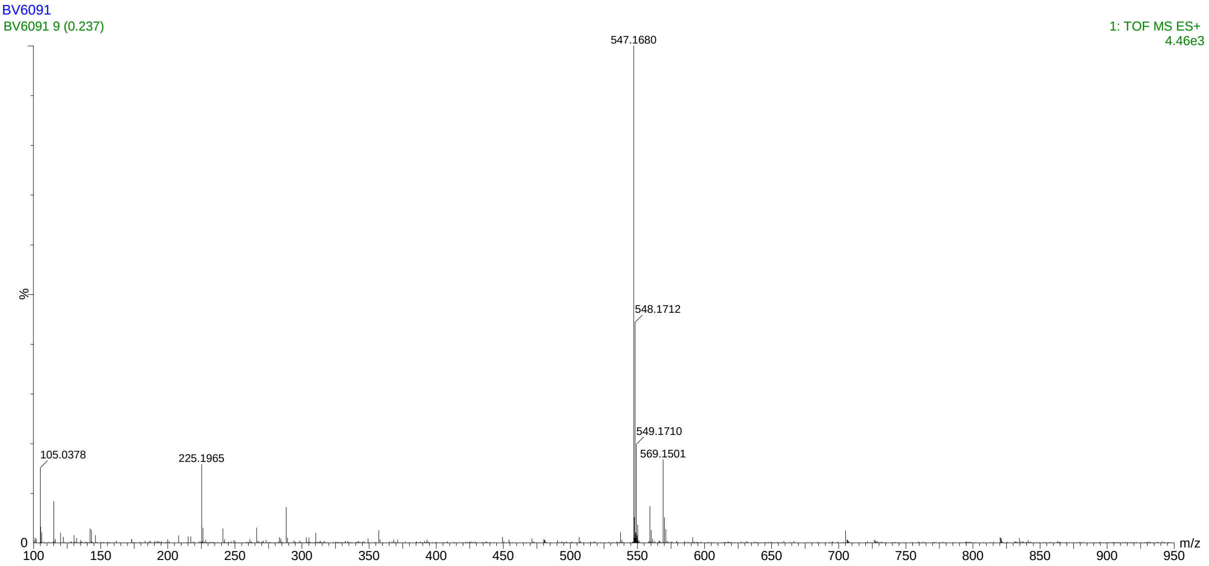

HRMS spectrum for **38**. m/z: $[M + H]^+$ Calcd for $C_{25}H_{31}N_4O_6S_2$ 547.1685; Found 547.1680

Compound 38, ^1H NMR (400 MHz, DMSO-d_6) spectrum

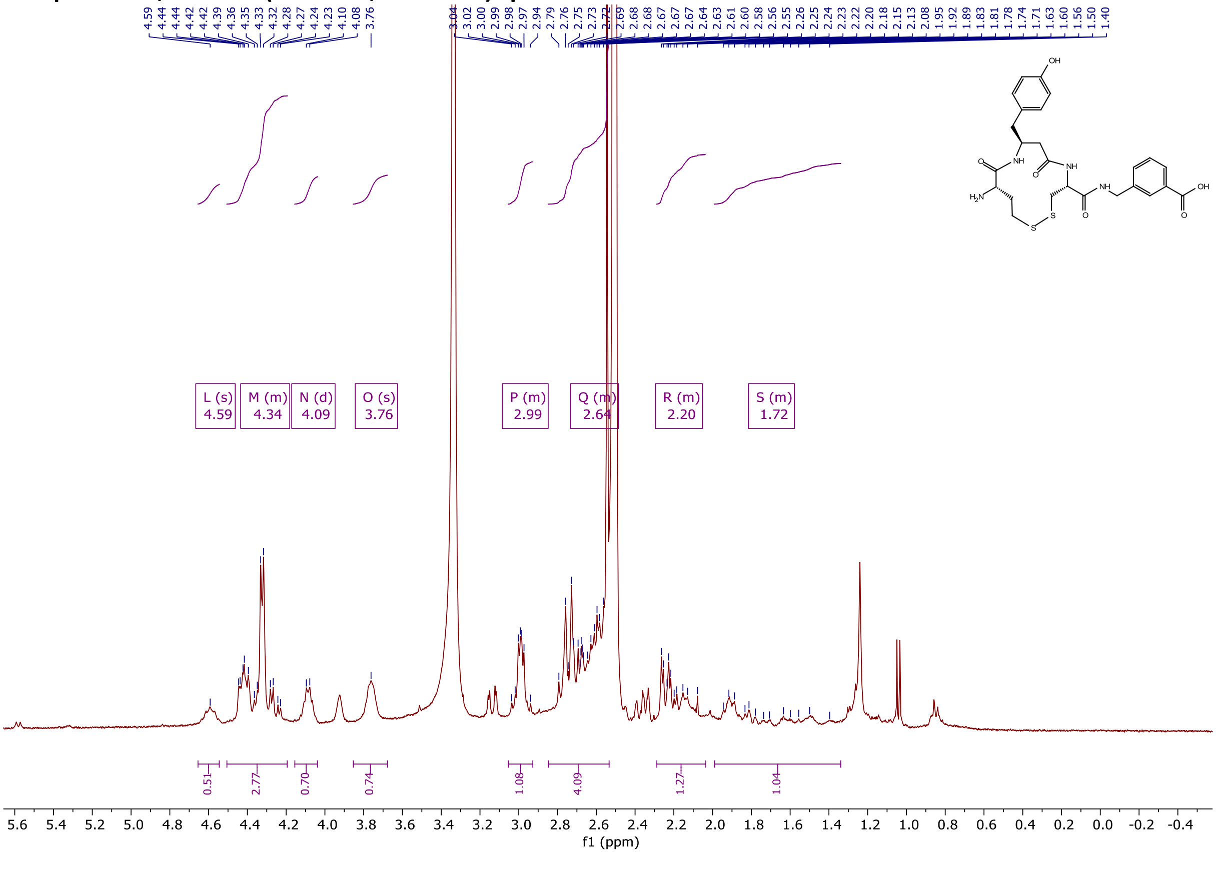

Compound 39, LC-MS

Name: 4-(((4*R*,8*S*,11*S*)-11-amino-8-(4-hydroxybenzyl)-6,10-dioxo-1,2-dithia-5,9-diazacyclotridecane-4-carboxamido)methyl)benzoic acid (TFA salt)

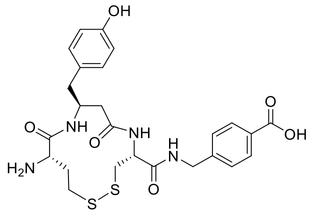

A)

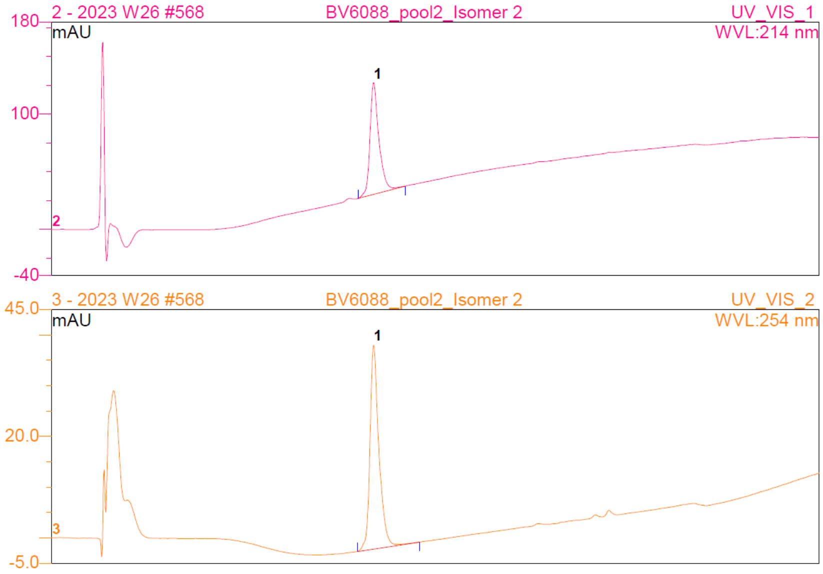

B)

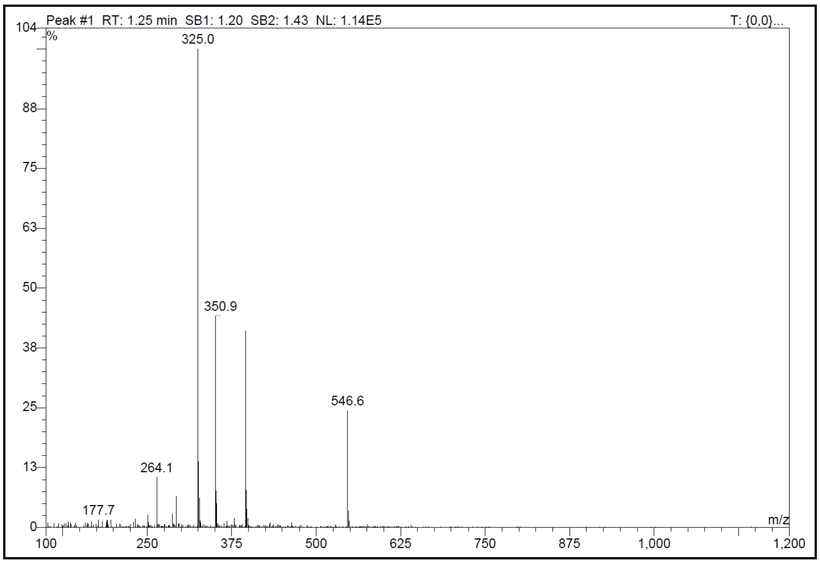

A) Analytical RP-HPLC of compound **39** on C18 column, acetonitrile gradient 5-100% of 0.05% formic acid in acetonitrile/water for 3 min. Top to bottom: UV detection at 214 and 254 nm. B) Low-resolution mass spectrum of **39**.

Compound 39, HRMS (ESI/TOF)

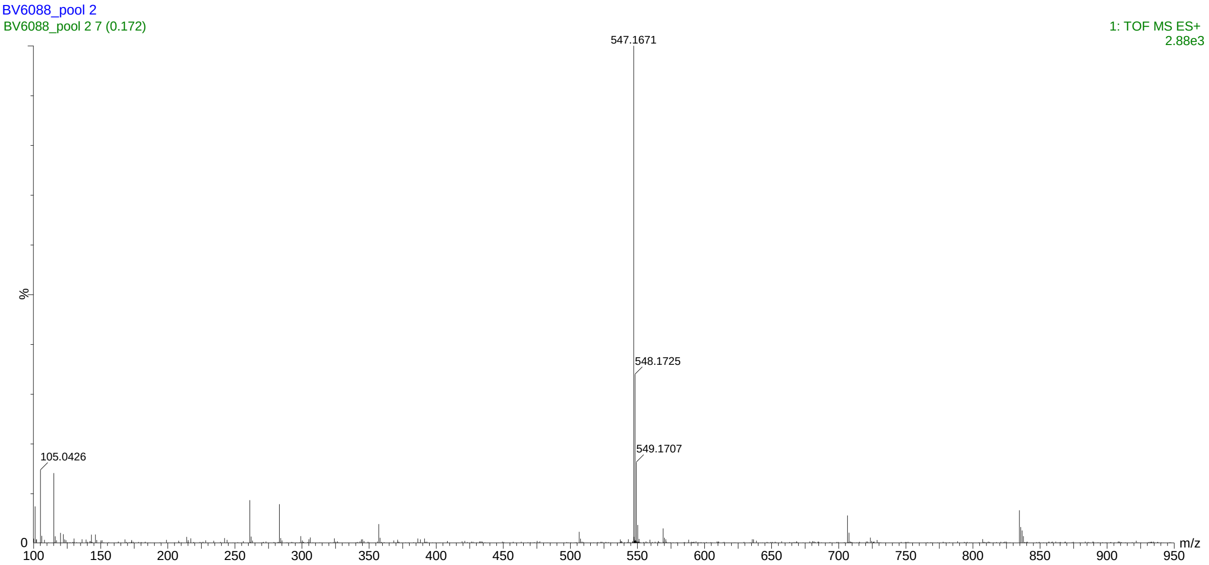

HRMS spectrum for **39**. m/z: $[M + H]^+$ Calcd for $C_{25}H_{31}N_4O_6S_2$ 547.1685; Found 547.1671

Compound 39, ^1H NMR (400 MHz, DMSO-d_6) spectrum

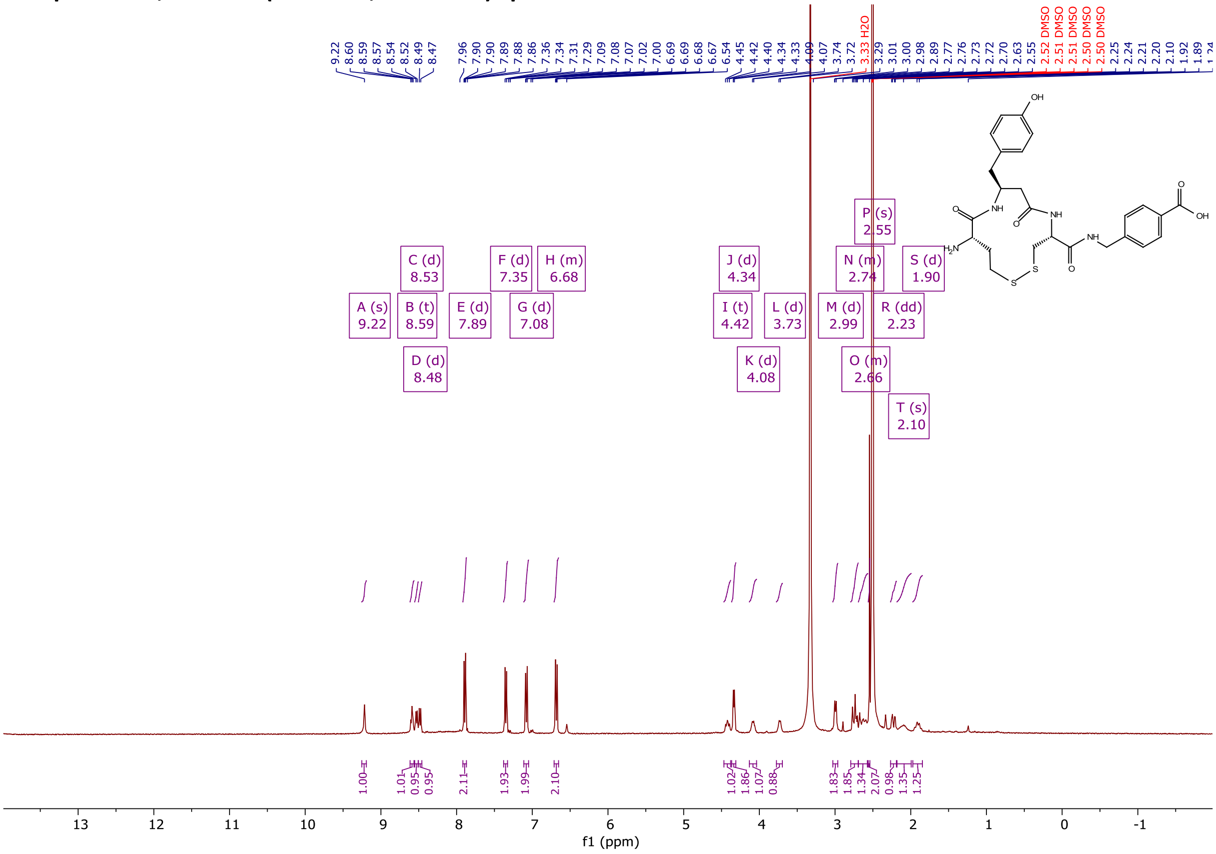

Dose-response curves for Peptidomimetics 28-39

The compounds were tested in a single experimental run at 11 concentration points and the IC_{50} curves were generated using XLfit version 5.4.0.8. The assay robustness has been previously reported[a] using the reference compound HA08, IC_{50} = 16 nM ± 2 nM (n = 3), determined in three separate testing rounds.

a) Beveridge, J.; Söderström, M.; Prieto-Díaz, R.; Gutierrez-de-Teran, H.; Odell, L.R.; Hallberg, M., Larhed, M.; Gising, J. Benzylhydroxamic acids as inhibitors of insulin regulated aminopeptidase (IRAP), *Eur J. Med. Chem. Reports*, **2024**, *12*, 100215. https://doi.org/10.1016/j.ejmcr.2024.100215

Compound 28

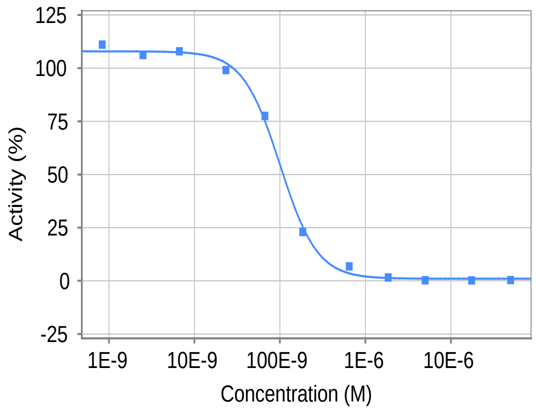

Compound 29

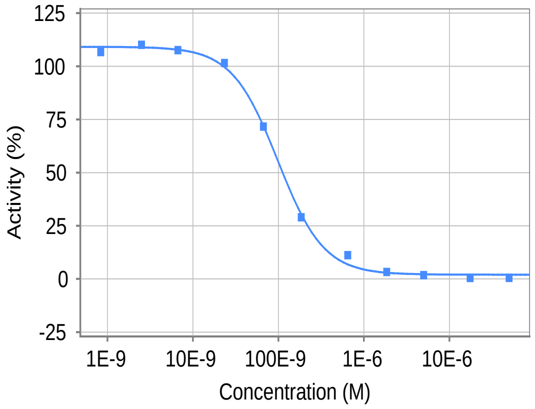

Compound 30

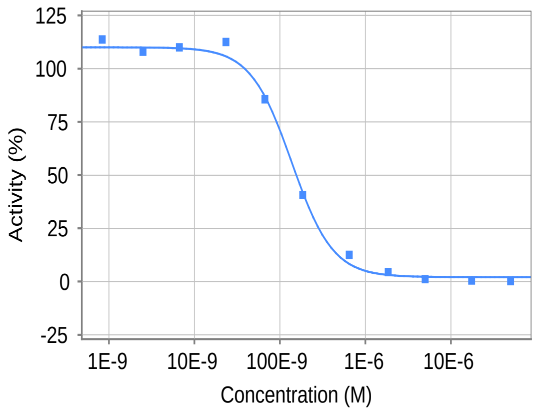

Compound 31

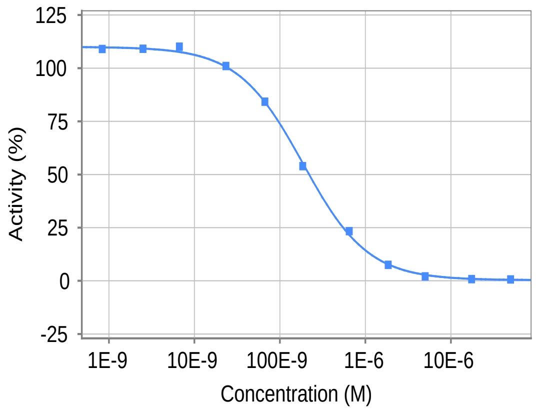

Compound 32

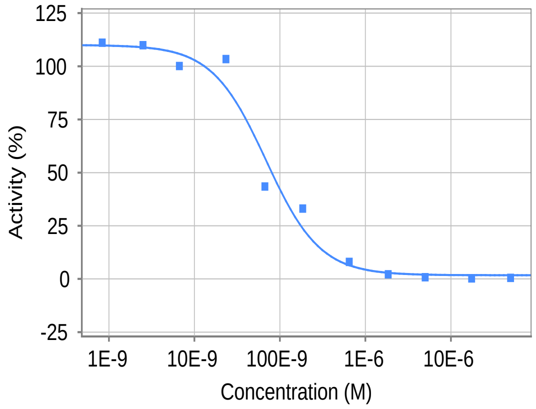

Compound 33

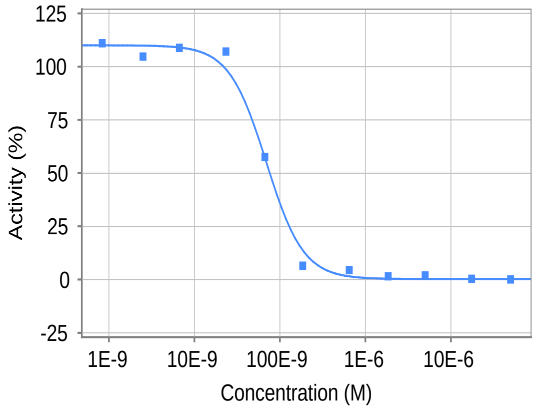

Compound 34

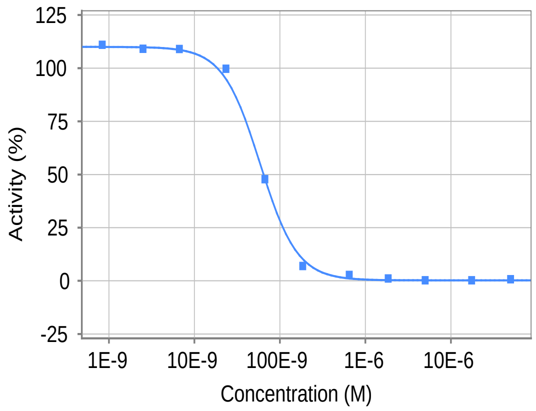

Compound 35

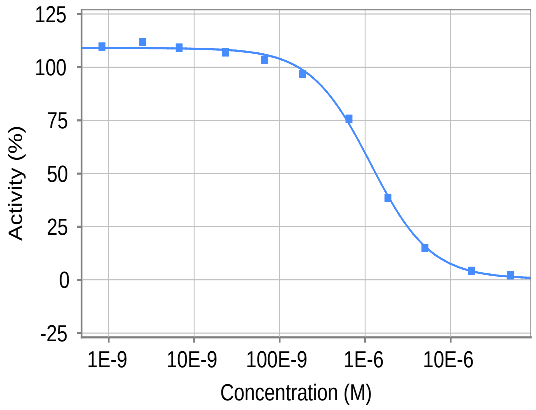

Compound 36

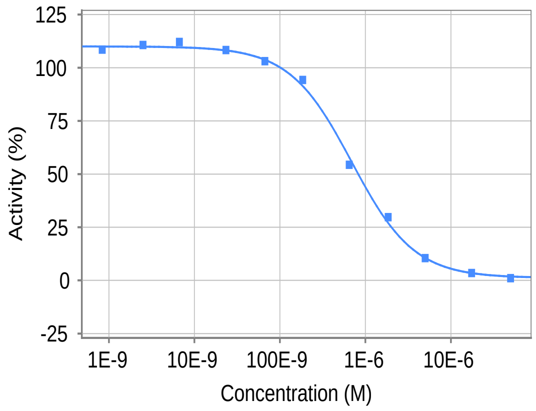

Compound 37

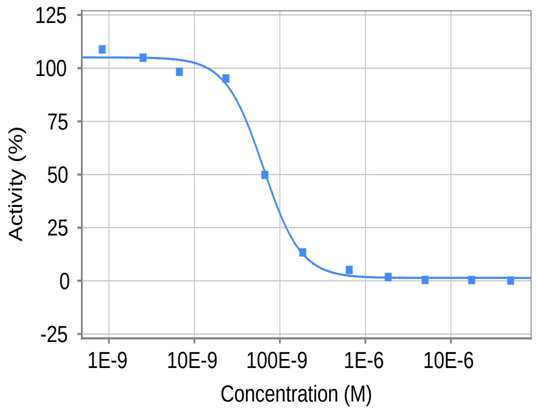

Compound 38

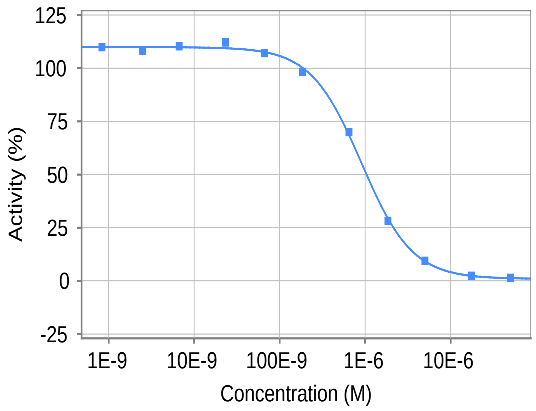

Compound 39

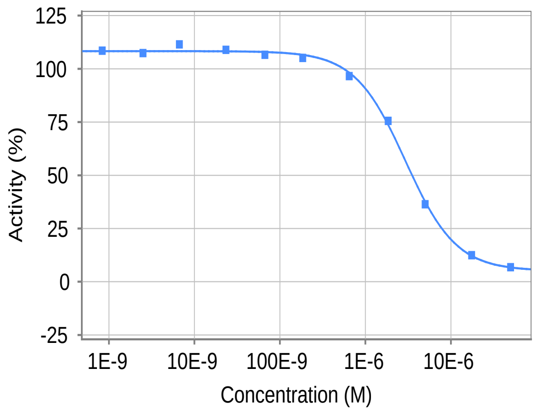

MD simulation and PLS modelling details

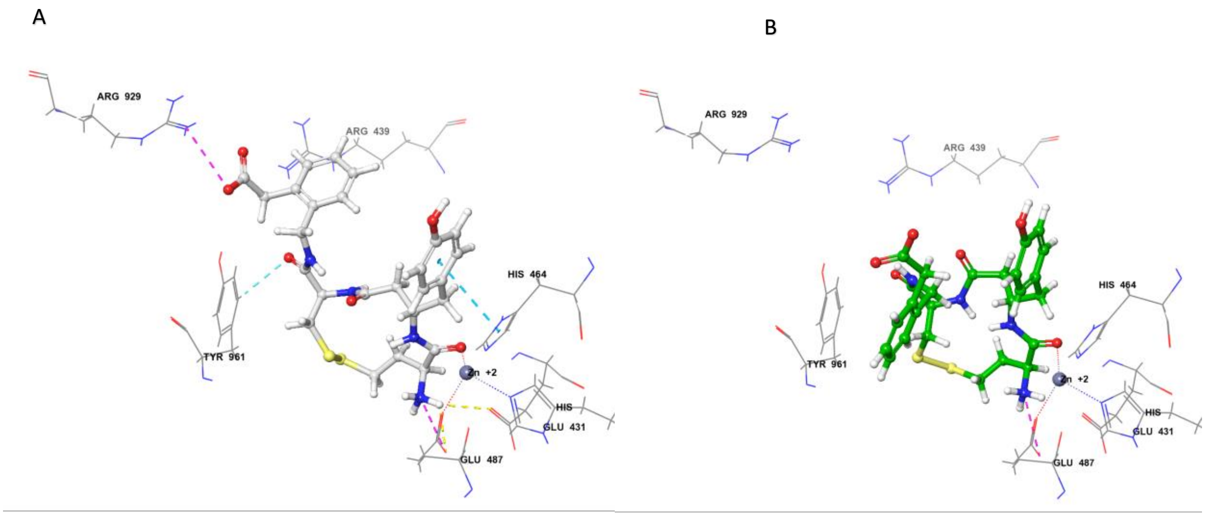

Figure S6. Crystal structure binding pose of **HA08** from pdbid:6YDX obtained after protein preparation is shown in A. Electrostatic interactions to ARG929 and proximity to ARG439 are visible, as well as the interactions between the N-terminal and the protein which are found throughout the simulations for all ligands. Hydrogen bonds are shown with a yellow dashed line, ionic interactions with a purple dashed line, and pi-pi interactions with a light blue dashed line, coordination to the zinc is shown as a dotted line. Shown in B is an alternate crystal structure binding pose of **HA08** from pdbid:6YDX can be seen featuring pi-pi stacking between the TYR961 and the C-terminal phenyl ring.

The molecular dynamics simulation was configured in a cubic water box using the TIP3P water model with a 10 Å buffer from the protein in all directions. The system was neutralized with sodium ions. Sodium and chloride ions were then added to a concentration of 0.15 M, with an exclusion zone of 15 Å around the ligand. The system was set up in an NPT ensemble using the MTK method with a temperature of 310 K and pressure of 1.01325 bar. The simulation was run for 500 ns with a timestep of 0.001 ps for bonded interactions, 0.001 ps for near non-bonded interactions, and 0.003 ps for far non-bonded interactions. Temperature regulation was maintained using a Nosé-Hoover thermostat with a relaxation time of 1.0 ps, while pressure was controlled with a Langevin barostat with a relaxation time of 2.0 ps. Electrostatic interactions were calculated using the u-series method with a cutoff radius of 10.0 Å. Trajectory data was recorded every 100 ps, and energy information was collected every 1.2 ps. Periodic boundary conditions and checkpointing (240.06 ps intervals) were implemented using Desmond's Multigrator framework.

Table S3. Simulation quality properties for **HA08**.

**HA08 Run 1 Properties**			
**Property**	**Average**	**Std. Dev.**	**Slope (ps^{-1})**
Total energy (kcal/mol)	-264273.532	275.367	0
Potential energy (kcal/mol)	-329725.279	228.443	0
Temperature (K)	309.693	0.739	0
Pressure (bar)	1.522	72.908	0
Volume (Å^3)	1034503.1	1210.421	-0.001
**HA08 Run 2 Properties**			
**Property**	**Average**	**Std. Dev.**	**Slope (ps^{-1})**
Total energy (kcal/mol)	-293184.129	159.618	0

Potential energy (kcal/mol)	-365266.109	140.634	0
Temperature (K)	309.693	0.339	0
Pressure (bar)	1.196	30.27	0
Volume (Å^3)	144864.477	673.416	-0.001
**HA08 Run 3 Properties**			
**Property**	**Average**	**Std. Dev.**	**Slope (ps^{-1})**
Total energy (kcal/mol)	-293176.757	157.192	0
Potential energy (kcal/mol)	-365243.416	137.557	0
Temperature (K)	309.691	0.339	0
Pressure (bar)	0.954	30.352	0
Volume (Å^3)	144406.398	672.54	-0.001

Table S4. Simulation quality properties for ligand **37**.

**Ligand 37 Run 1 Properties**			
**Property**	**Average**	**Std. Dev.**	**Slope (ps^{-1})**
Total energy (kcal/mol)	-260006.812	276.594	0
Potential energy (kcal/mol)	-324475.4	226.97	0
Temperature (K)	309.69	0.754	0
Pressure (bar)	0.956	73.491	0
Volume (Å^3)	1018101.53	1198.909	-0.001
**Ligand 37 Run 2 Properties**			
**Property**	**Average**	**Std. Dev.**	**Slope (ps^{-1})**
Total energy (kcal/mol)	-846836.98	248.375	0
Potential energy (kcal/mol)	-1042662.5	212.573	0
Temperature (K)	309.695	0.235	0
Pressure (bar)	1.004	15.344	0
Volume (Å^3)	3200641.91	1053.045	-0.001
**Ligand 37 Run 3 Properties**			
**Property**	**Average**	**Std. Dev.**	**Slope (ps^{-1})**
Total energy (kcal/mol)	-1037617	269.384	0
Potential energy (kcal/mol)	-1277157	230.924	0
Temperature (K)	309.695	0.22	0
Pressure (bar)	0.91	13.632	0
Volume (Å^3)	3926828.67	1143.997	-0.001

Table S5. Simulation quality properties for ligand **38**.

**Ligand 38 Run 1 Properties**			
**Property**	**Average**	**Std. Dev.**	**Slope (ps^{-1})**
Total energy (kcal/mol)	-984517.18	260.23	0
Potential energy (kcal/mol)	-1211882.4	228.53	0
Temperature (K)	309.694	0.223	0
Pressure (bar)	0.868	13.983	0
Volume (Å^3)	3724876.65	1095.8	0
**Ligand 38 Run 2 Properties**			
**Property**	**Average**	**Std. Dev.**	**Slope (ps^{-1})**
Total energy (kcal/mol)	-259997.51	277.395	0
Potential energy (kcal/mol)	-324473.178	228.275	0
Temperature (K)	309.697	0.754	0
Pressure (bar)	0.808	73.165	0
Volume (Å^3)	1018509.95	1207.793	0
**Ligand 38 Run 3 Properties**			
**Property**	**Average**	**Std. Dev.**	**Slope (ps^{-1})**
Total energy (kcal/mol)	-903114.85	251.393	0
Potential energy (kcal/mol)	-1111823.6	214.037	0
Temperature (K)	309.695	0.229	0
Pressure (bar)	1.146	14.721	0
Volume (Å^3)	3414897.98	1068.439	-0.001

Table S6. Simulation quality properties for ligand **39**.

**Ligand 39 Run 1 Properties**			
**Property**	**Average**	**Std. Dev.**	**Slope (ps^{-1})**
Total energy (kcal/mol)	-907498.78	254.717	0
Potential energy (kcal/mol)	-117176.333	217.232	0
Temperature (K)	309.697	0.229	0
Pressure (bar)	0.998	14.668	0
Volume (Å^3)	4430637.251	1073.589	-0.001
**Ligand 39 Run 2 Properties**			
**Property**	**Average**	**Std. Dev.**	**Slope (ps^{-1})**
Total energy (kcal/mol)	-1018996.89	273.68	0
Potential energy (kcal/mol)	-1254253.61	235.413	0
Temperature (K)	309.695	0.221	0
Pressure (bar)	1.041	13.736	0
Volume (Å^3)	3855707.244	1136.091	-0.001
**Ligand 39 Run 3 Properties**			
**Property**	**Average**	**Std. Dev.**	**Slope (ps^{-1})**
Total energy (kcal/mol)	-260033.25	279.377	0
Potential energy (kcal/mol)	-324507.15	230.351	0
Temperature (K)	309.689	0.755	0
Pressure (bar)	1.175	73.269	0
Volume (Å^3)	1018114.008	1212.733	0.001

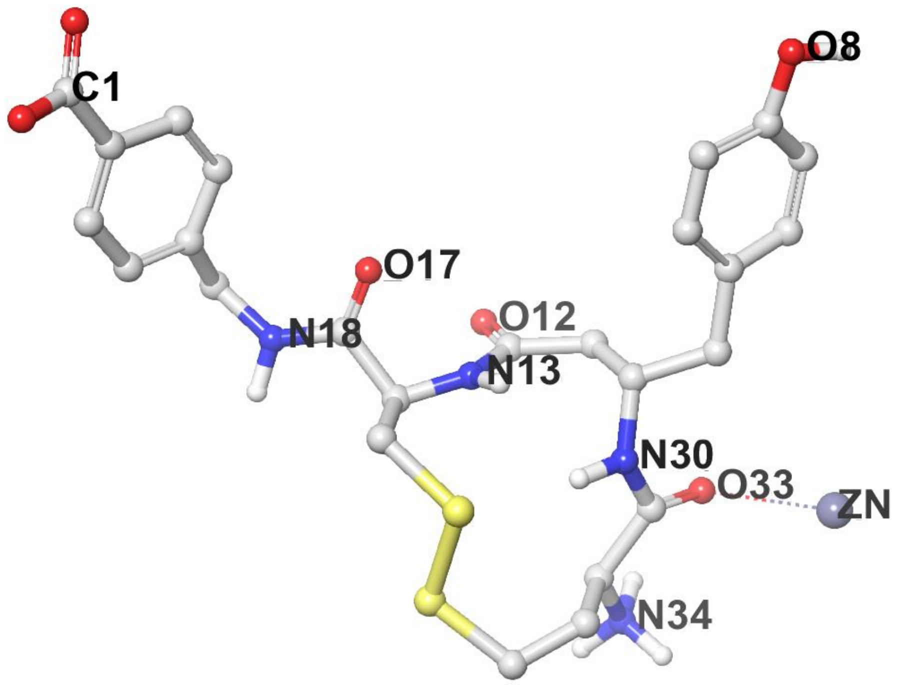

Figure S7. Ligand example with atom names labelled. For **HA08** C1 is named C27 in the trajectories in **Figure S9-S12**.

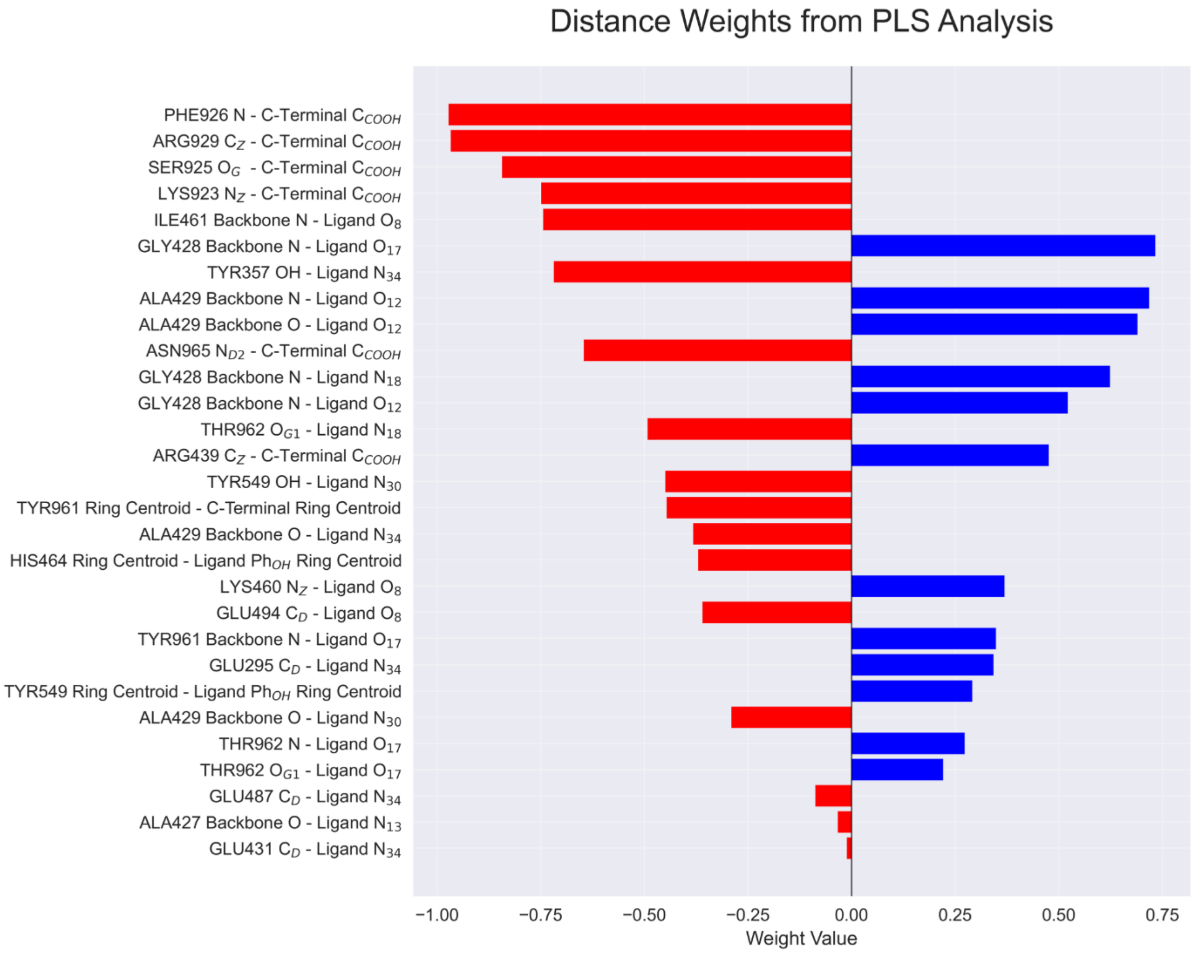

Figure S8. Distance weights from Partial Least Squares (PLS) analysis. The bar graph illustrates the relative importance (weights) of specific distances between ligand atoms and protein residues as determined by PLS regression modeling.

Table S7. Coefficients of the PLS model.

Feature	Coefficient
GLY428 N - Ligand O_{17}	0.0683214355336663
ALA429 N - Ligand O_{12}	0.0665468746704422
ALA429 O - Ligand O_{12}	0.06336508033948718
ARG439 C_Z - Ligand C-Terminal C_{COOH}	0.05749357170804193
GLY428 N - Ligand N_{18}	0.05713978275863275
GLY428 N - Ligand O_{12}	0.04489917370114061
TYR961 N - Ligand O_{17}	0.04446754026048563
GLU295 C_D - Ligand N_{34}	0.042857757327274724
THR962 N - Ligand O_{17}	0.03758538176932008
THR962 O_{G1} - Ligand O_{17}	0.03195632254017837
LYS460 N_Z - Ligand O_8	0.02834774473629549
TYR549 Ring Centroid - Ligand Ph-OH Ring Centroid	0.02057330642917553
GLU431 C_D - Ligand N-Terminal Amine	0.008628919962454329
ALA427 O - Ligand N_{13}	0.005542848709271484
GLU487 C_D - Ligand N_{34}	-0.002716373172414233
ALA429 Backbone O - Ligand N_{30}	-0.025156858054975136
TYR961 Ring Centroid - Ligand C-Terminal Ring Centroid	-0.03665383127722303
ALA429 Backbone O - Ligand N34	-0.03717319818481611
TYR549 OH - Ligand N_{30}	-0.040075397106171015
GLU494 C_D - Ligand O_8	-0.04628702439026984
HIS464 Ring Centroid - Ligand Ph-OH Ring Centroid	-0.046458694407943174
THR962 O_{G1} - Ligand N_{18}	-0.04784926299999238
ASN965 N_{D2} - Ligand C-Terminal C_{COOH}	-0.0640783569921399
ILE461 Backbone N - Ligand O_8	-0.07603665318667994
TYR357 OH - Ligand N_{34}	-0.0772168721170838
LYS923 N_Z - Ligand C-Terminal C_{COOH}	-0.08089460080752713
SER925 O_G - Ligand C-Terminal C_{COOH}	-0.08262254909682357
ARG929 C_Z - Ligand C-Terminal C_{COOH}	-0.10171471926840502
PHE926 N - Ligand C-Terminal C_{COOH}	-0.10184444503748707

Table S8. Distance cut-off for generating occupancy time for protein-ligand interaction.

To Residue:	Distance Cut-off (Å)
Default	3.5 Å
Glu	3.75 Å
Arg	6.0 Å
Lys929	5.0 Å
Lys517	5.0 Å
Asn	5.0 Å
Phe	5.0 Å
Ser	4.5 Å
Ring to Ring	6.0 Å

ARG929 C_Z - C-Terminal C_{COOH}
Distance Over Time

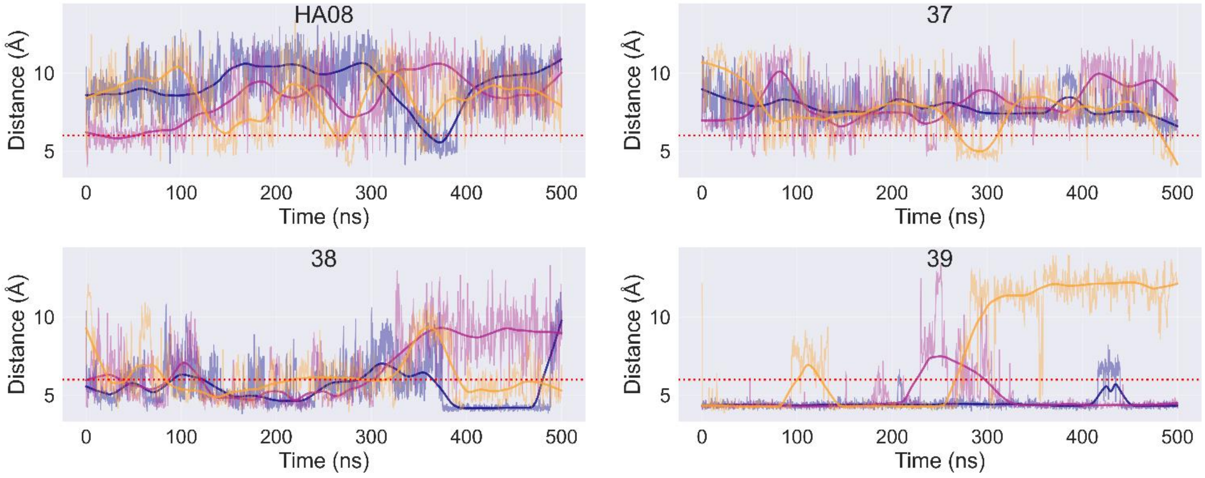

ARG439 C_Z - C-Terminal C_{COOH}
Distance Over Time

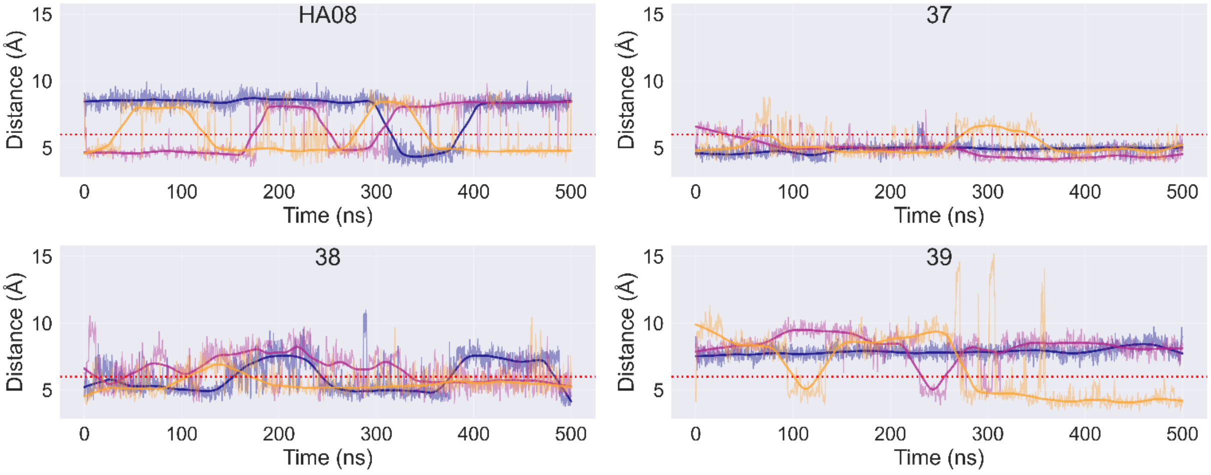

TYR961 Ring Centroid - C-Terminal Ring Centroid
Distance Over Time

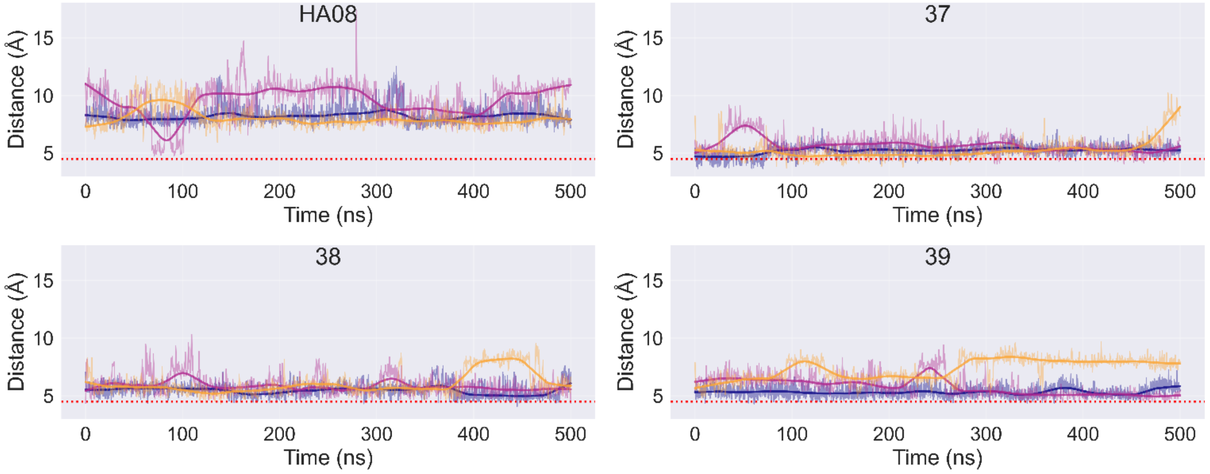

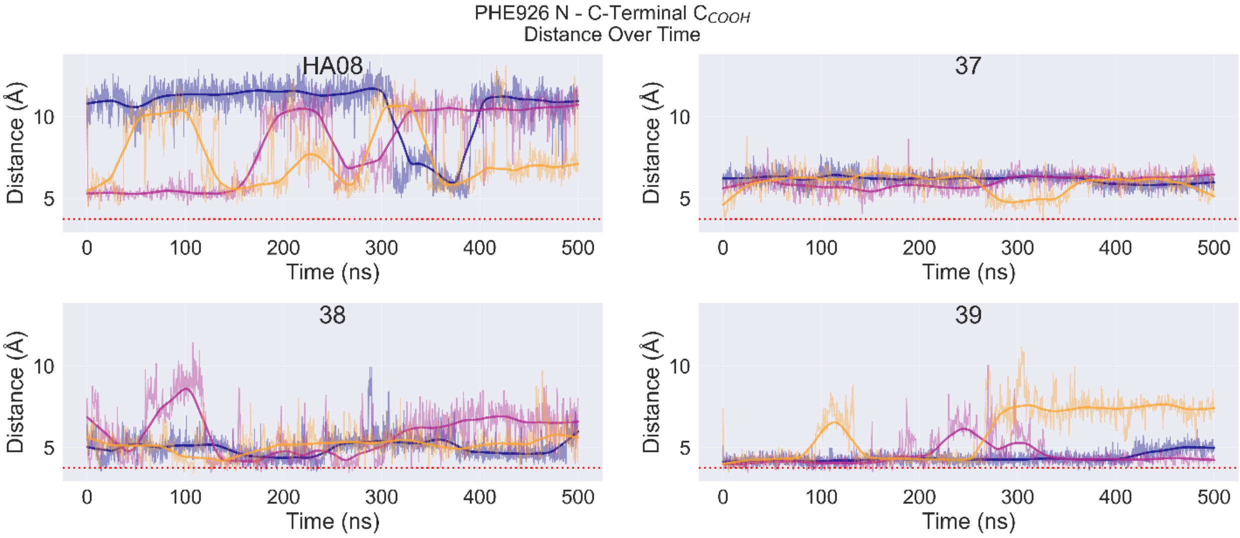
PHE926 N - C-Terminal C_{COOH}
Distance Over Time
HA08
37
38
39
Distance (Å)
Time (ns)
0
100
200
300
400
500
5
10

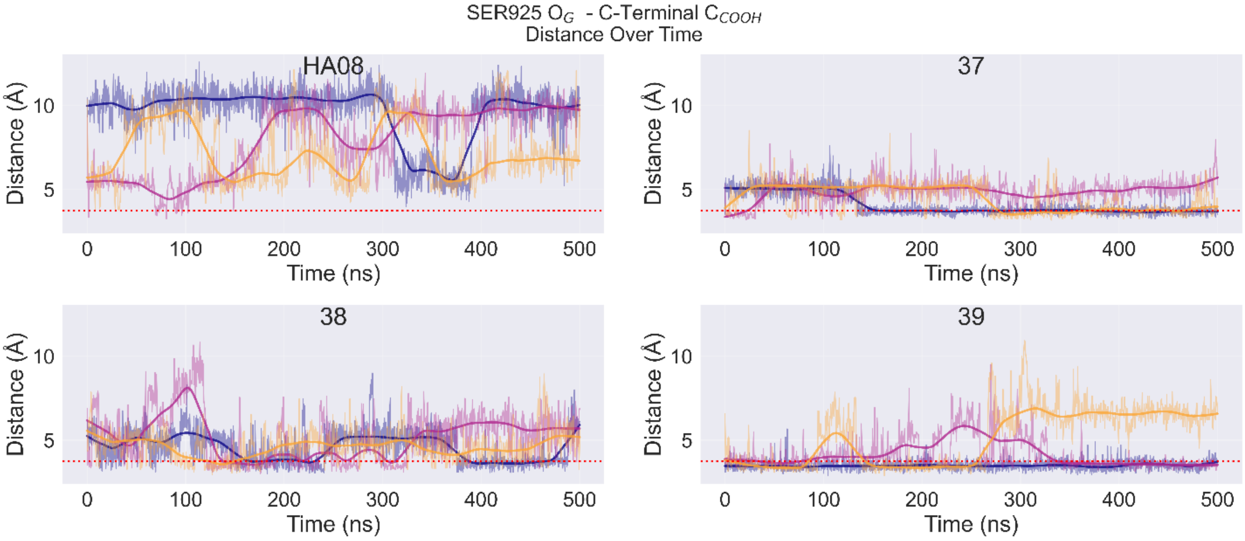
SER925 O_G - C-Terminal C_{COOH}
Distance Over Time
HA08
37
38
39
Distance (Å)
Time (ns)
0
100
200
300
400
500
5
10

LYS923 N_Z - C-Terminal C_{COOH}
Distance Over Time

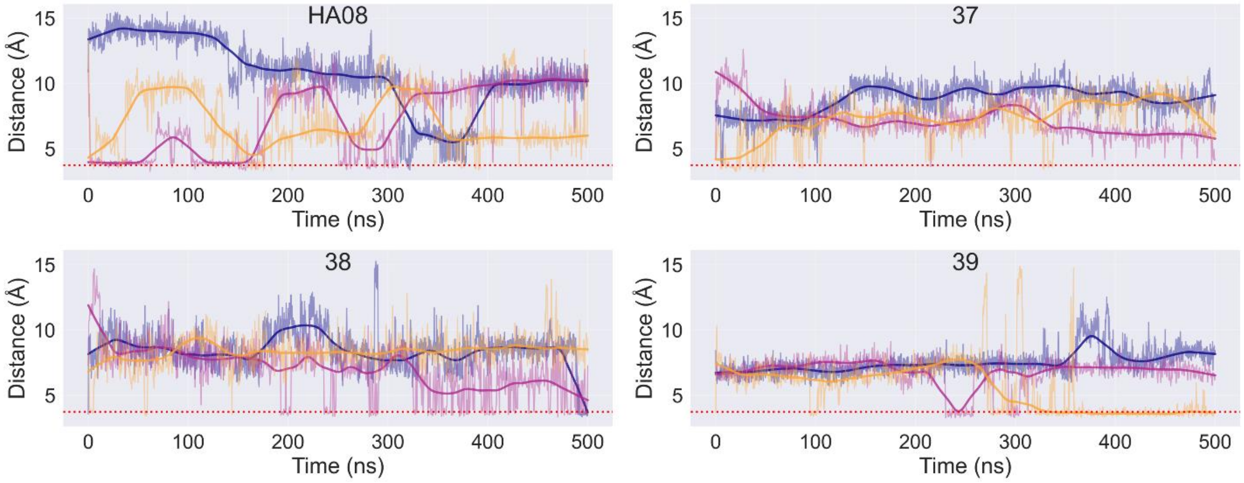

TYR357 OH - Ligand N_{34}
Distance Over Time

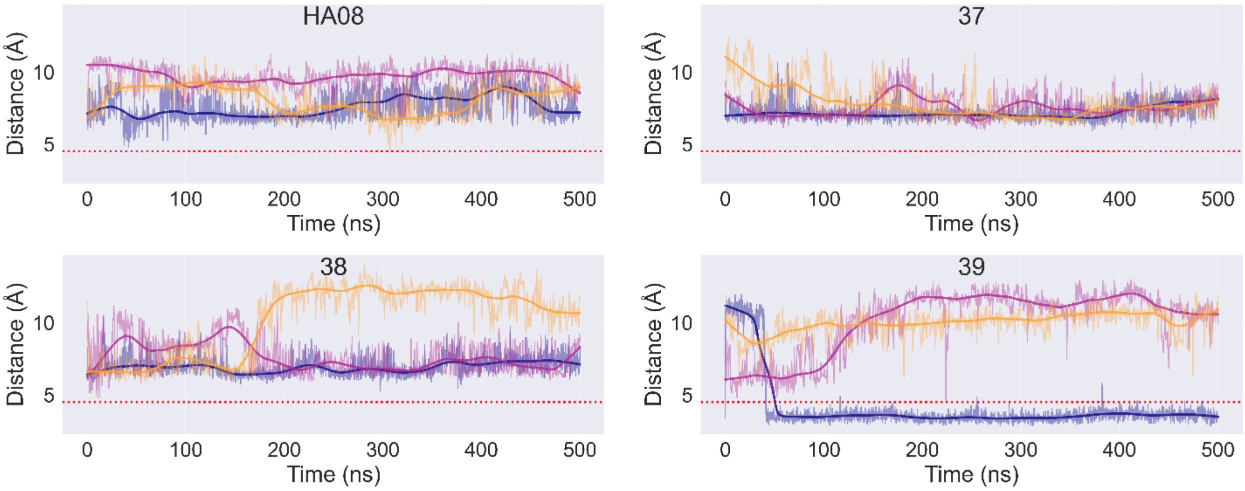

ILE461 Backbone N - Ligand O_8
Distance Over Time

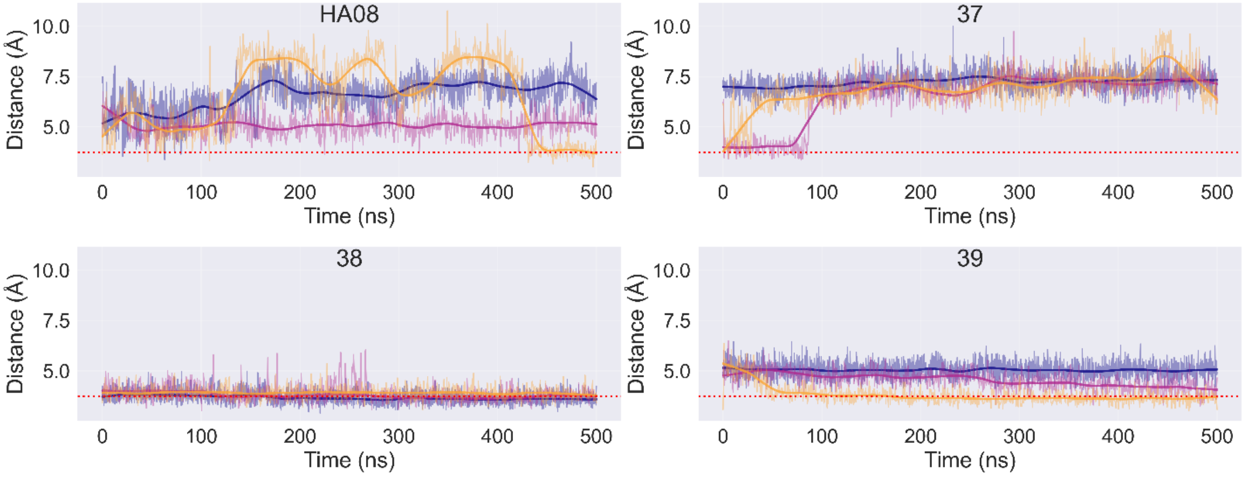

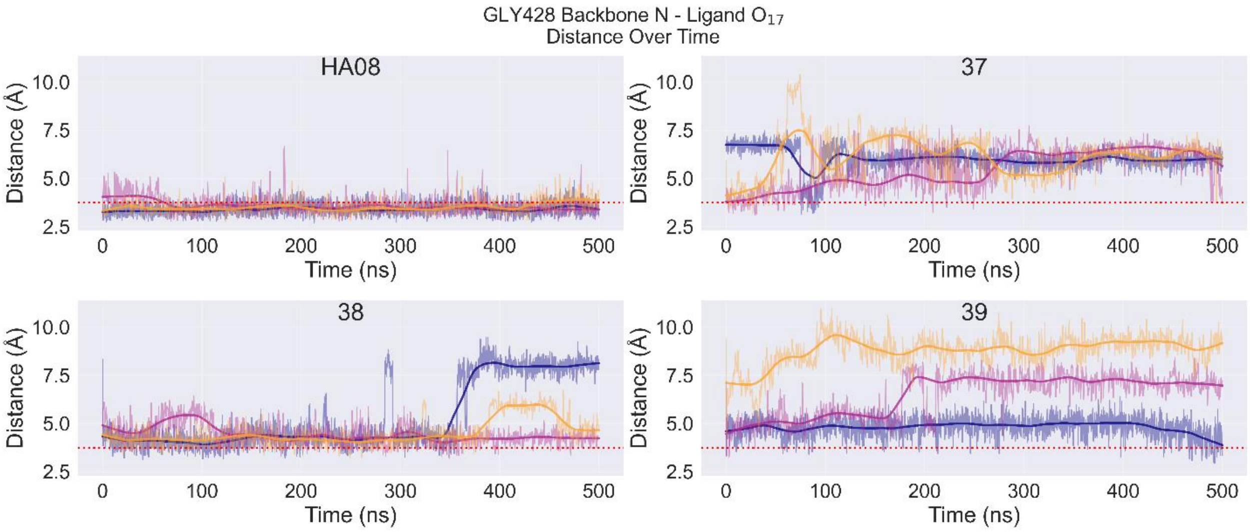
GLY428 Backbone N - Ligand O_{17}
Distance Over Time
HA08
37
38
39
Distance (Å)
Time (ns)

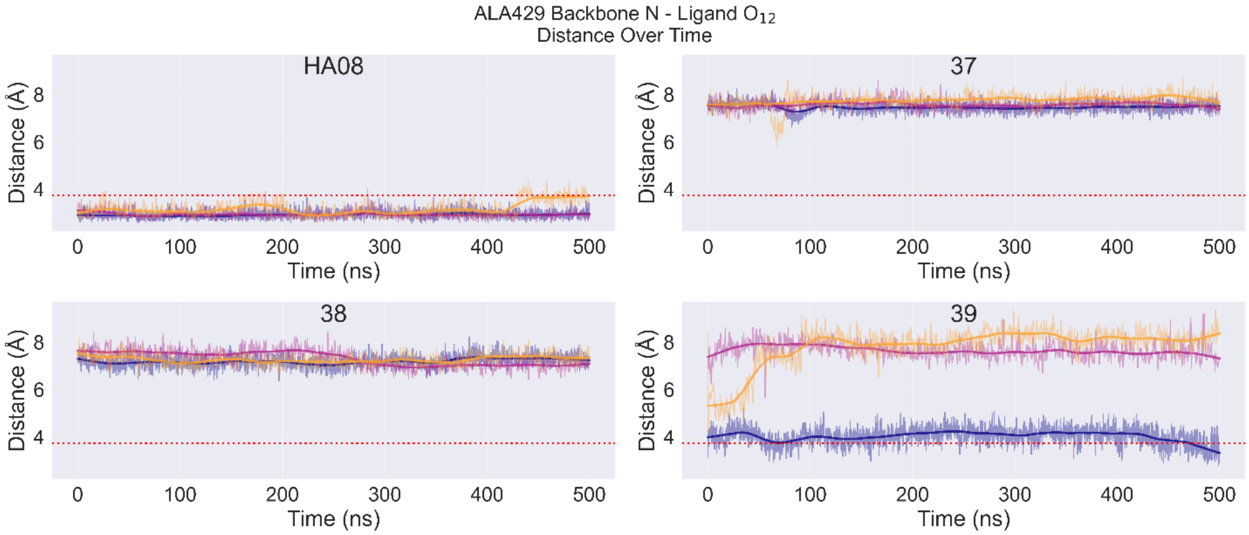
ALA429 Backbone N - Ligand O_{12}
Distance Over Time
HA08
37
38
39
Distance (Å)
Time (ns)

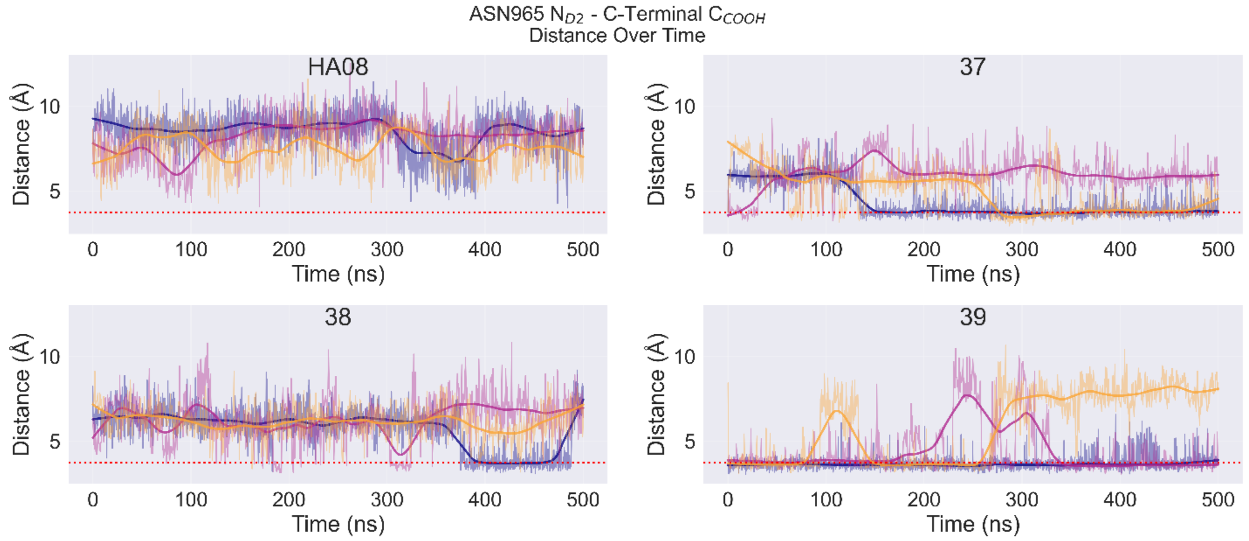
ASN965 N_{D2} - C-Terminal C_{COOH}
Distance Over Time
HA08
37
38
39
Distance (Å)
Time (ns)

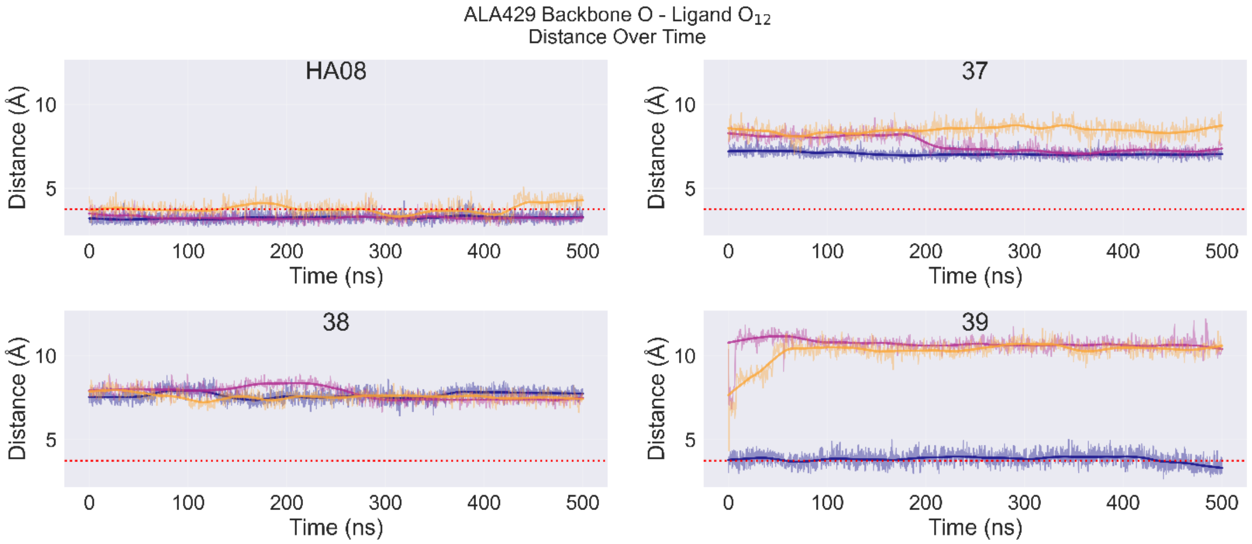

Figure S9. Time evolution plots for distances with PLS coefficient absolute value > 0.06. Color legend: Run 1 = blue, Run 2 = magenta, Run 3 = yellow.

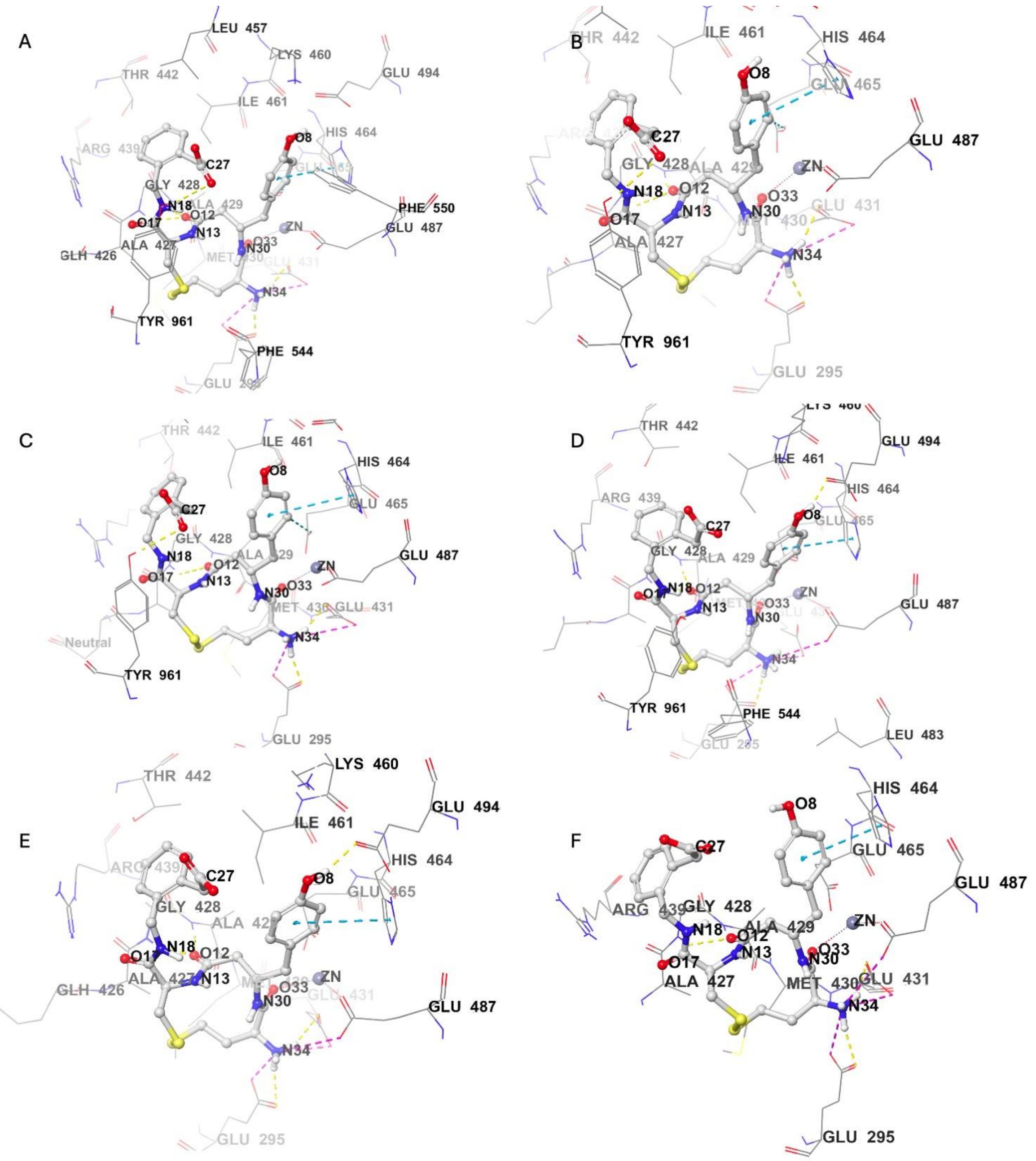

Figure S10. Selected frames at 100 ns intervals from frame 0, for ligand **HA08** run 1.

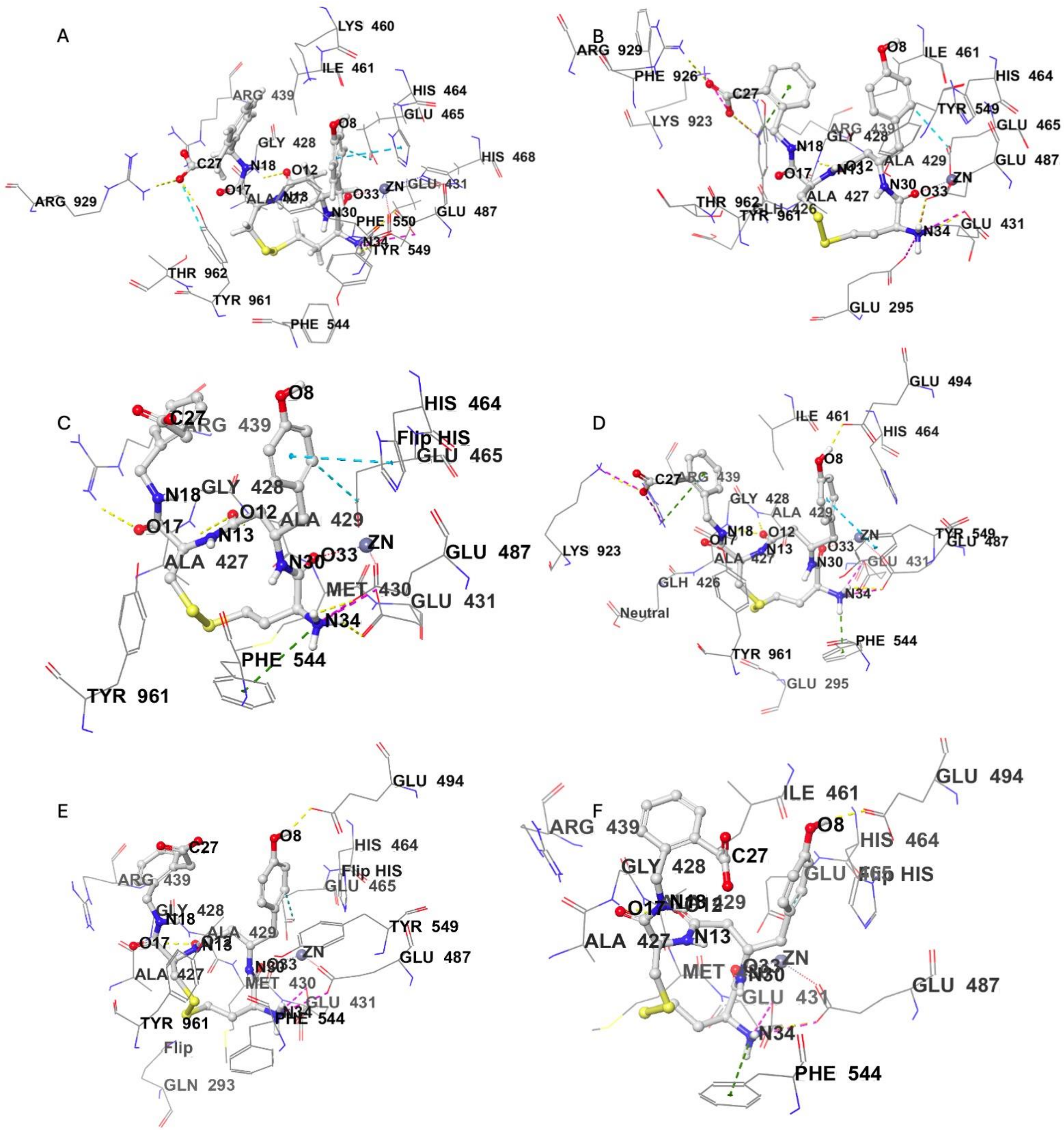

Figure S11. Selected frames at 100 ns intervals from frame 0, for ligand **HA08** run 2.

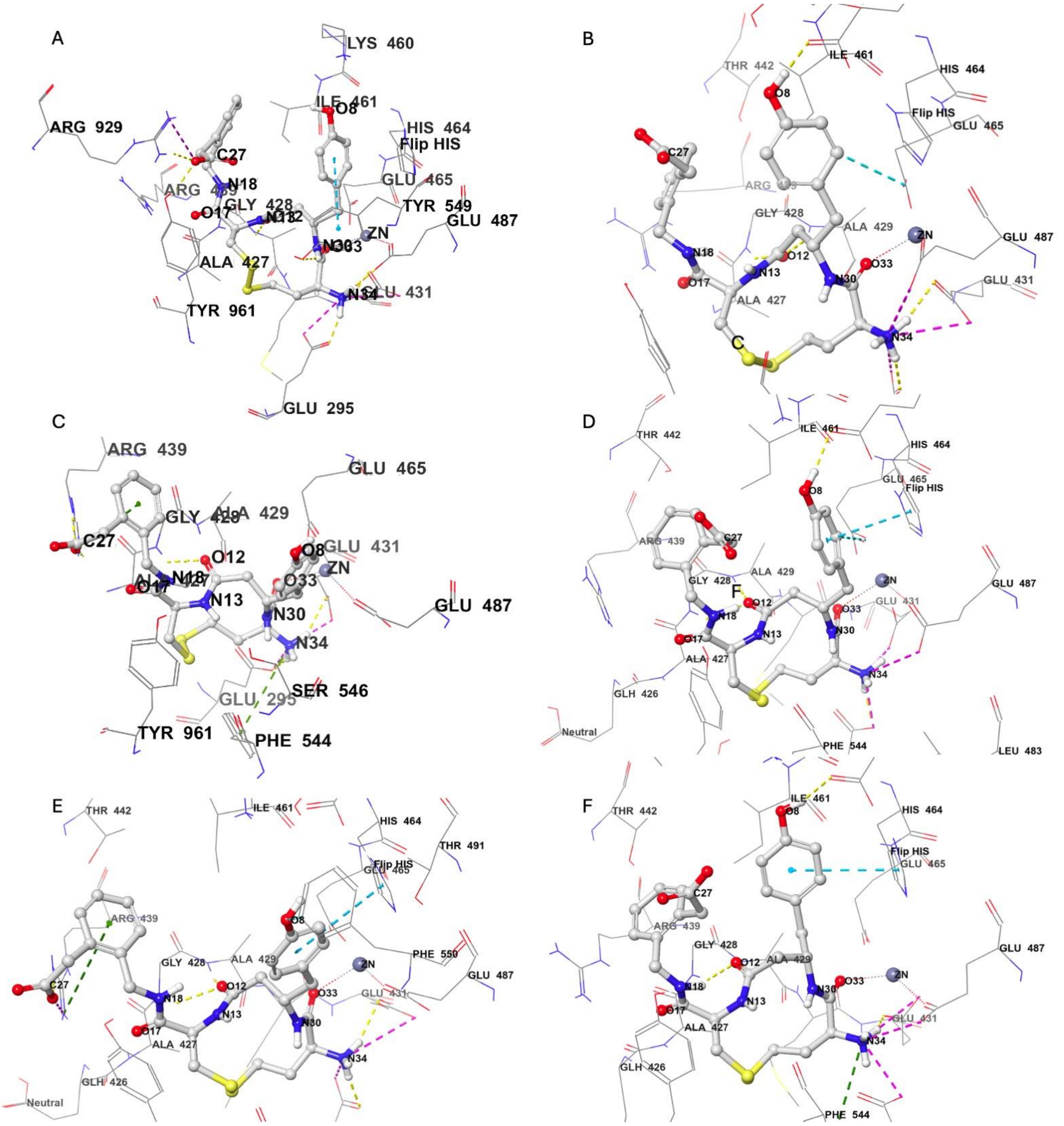

Figure S12. Selected frames at 100 ns intervals from frame 0, for ligand **HA08** run 3.

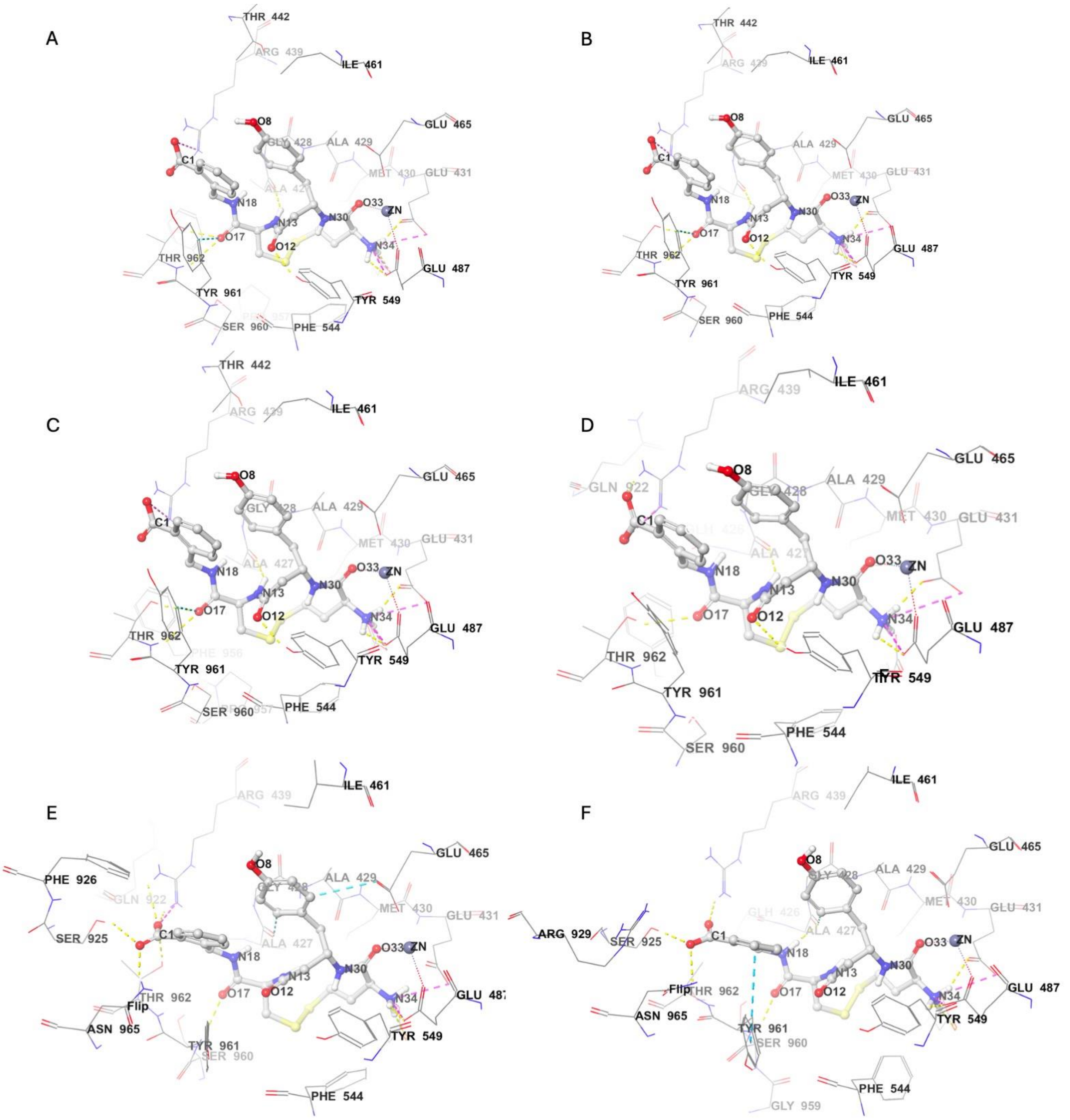

Figure S13. Selected frames at 100 ns intervals from frame 0, for ligand **37** run 1.

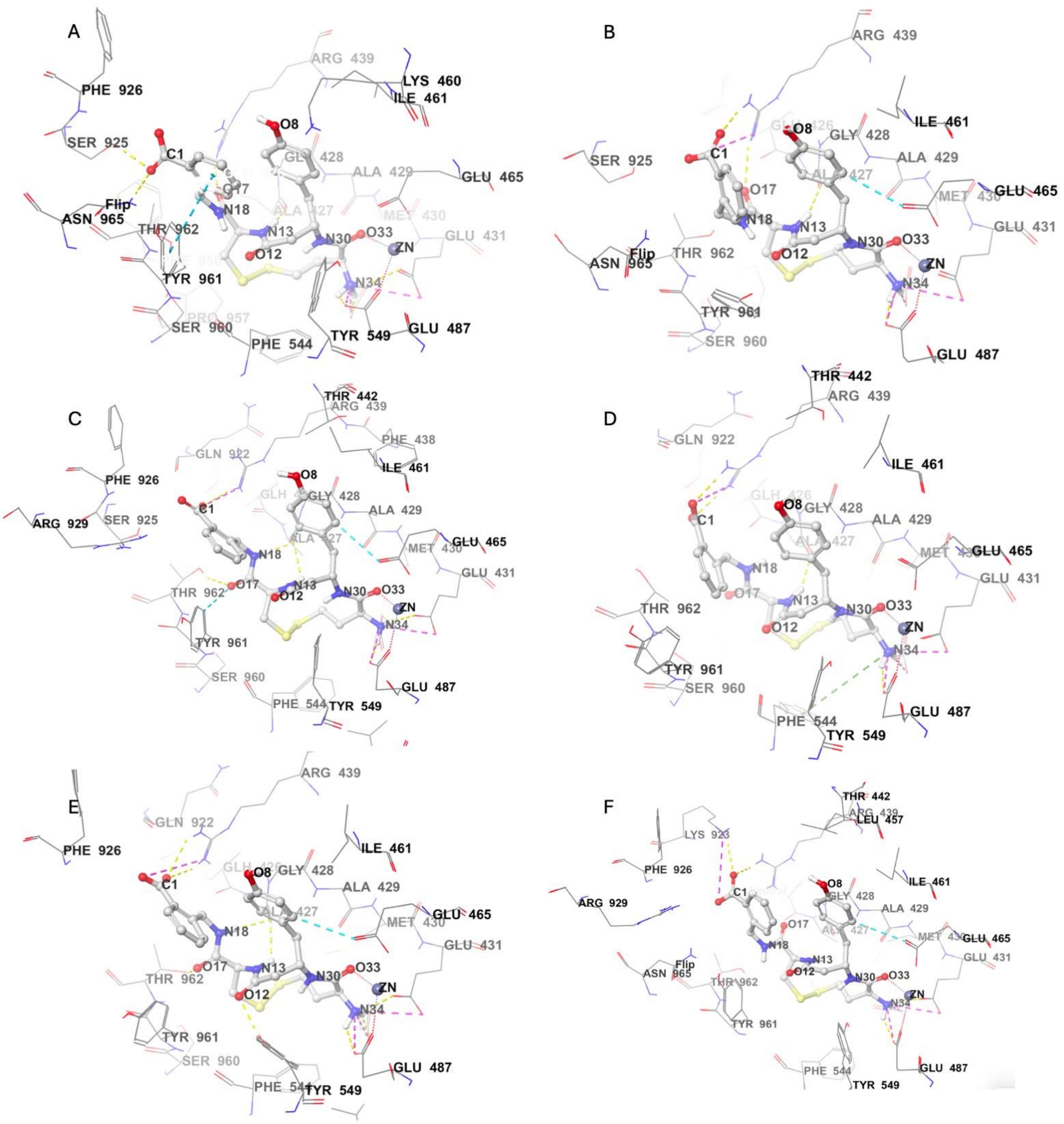

Figure S14. Selected frames at 100 ns intervals from frame 0, for ligand **37** run 2.

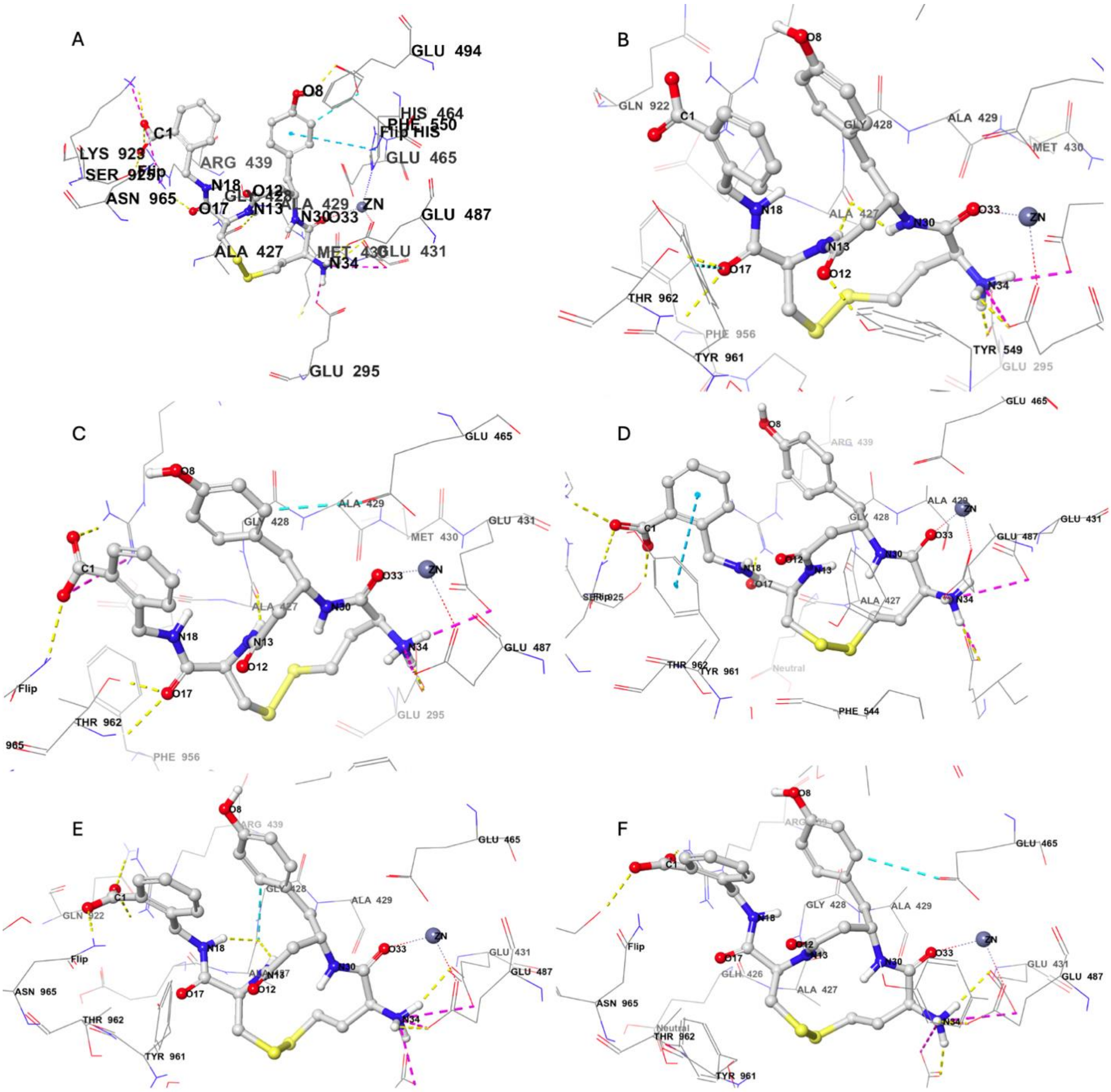

Figure S15. Selected frames at 100 ns intervals from frame 0, for ligand **37** run 3.

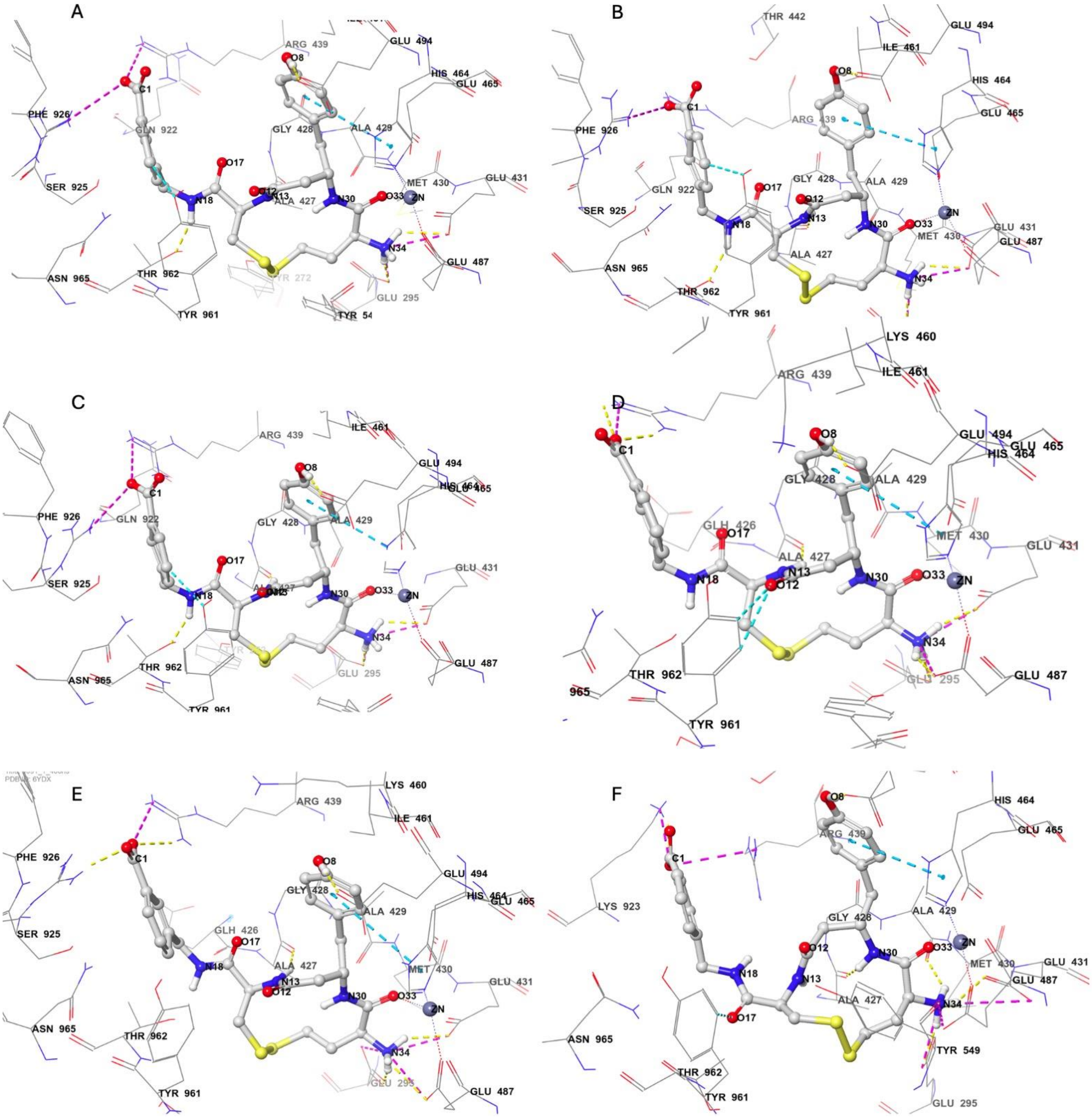

Figure S16. Selected frames at 100 ns intervals from frame 0, for ligand **38** run 1.

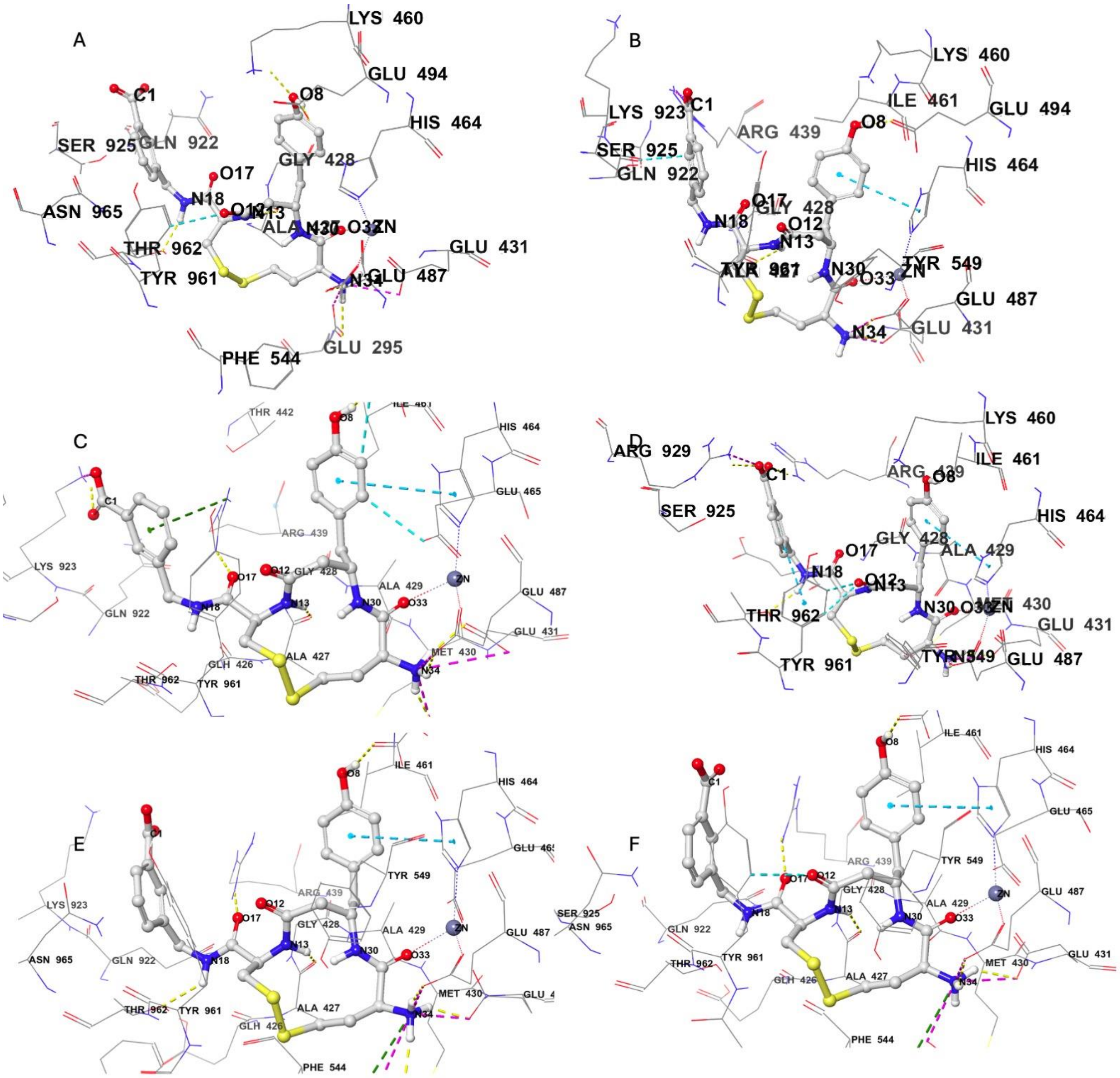

Figure S17. Selected frames at 100 ns intervals from frame 0, for ligand **38** run 2.

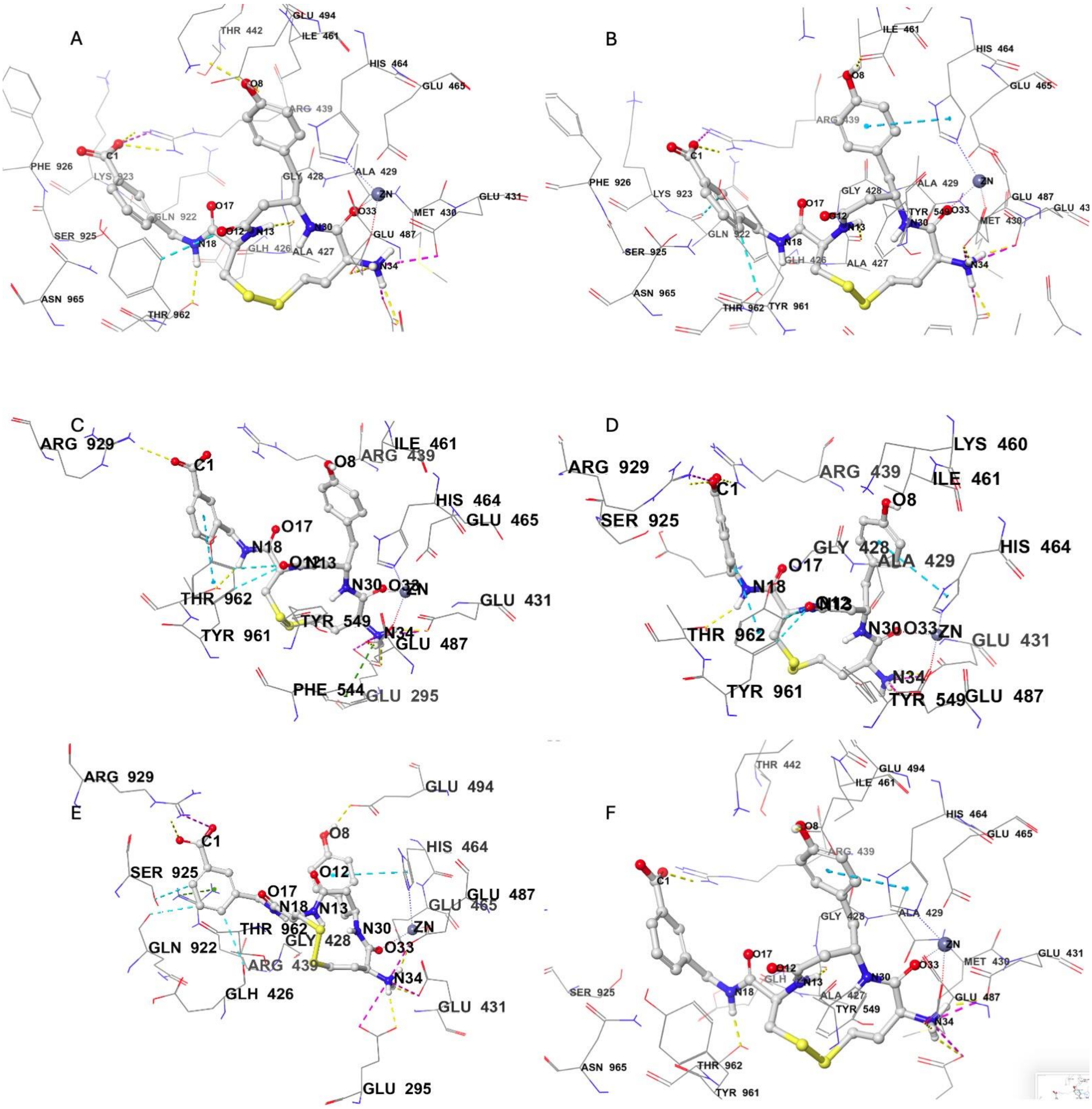

Figure S18. Selected frames at 100 ns intervals from frame 0, for ligand **38** run 3.

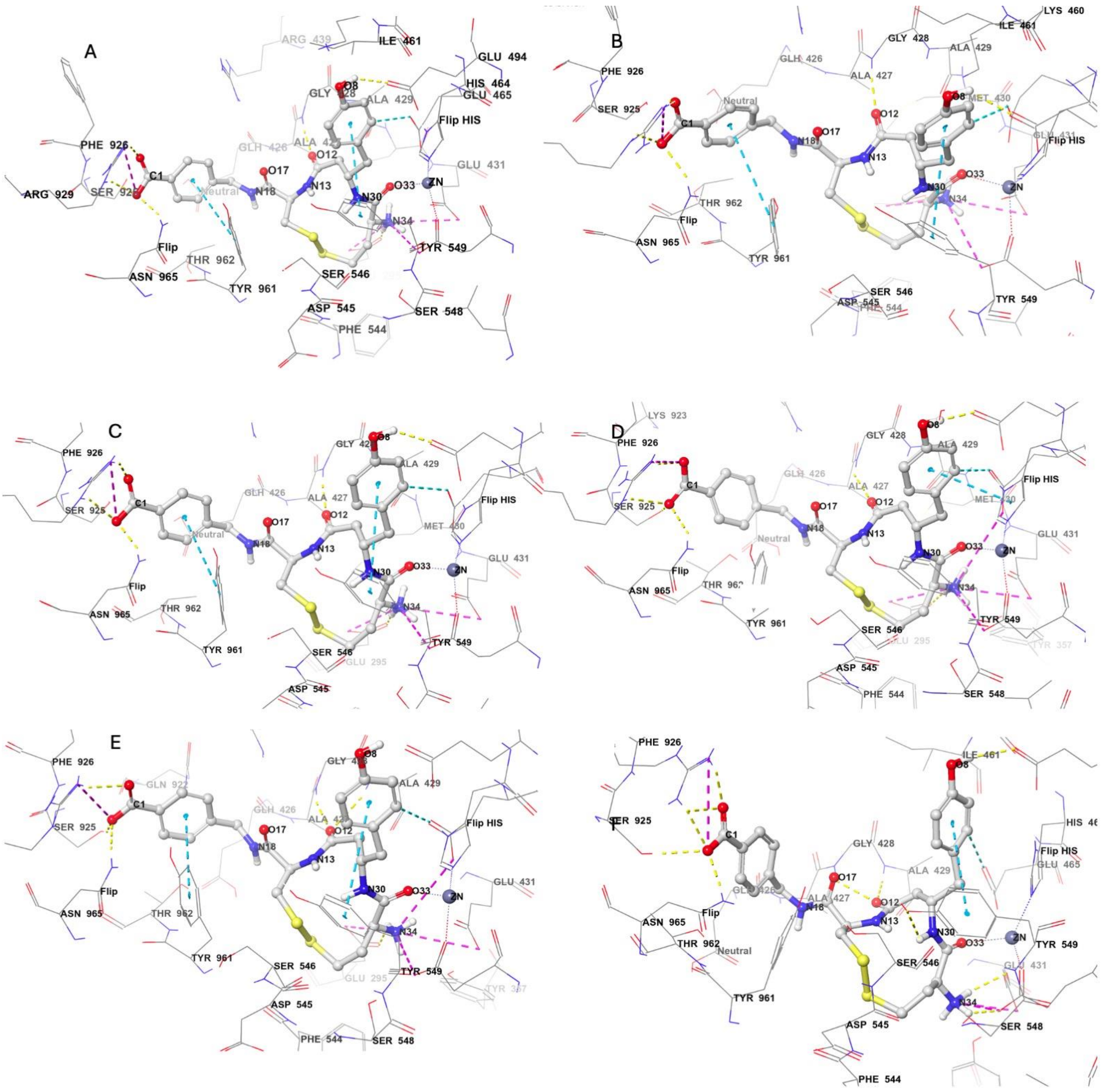

Figure S19. Selected frames at 100 ns intervals from frame 0, for ligand **39** run 1.

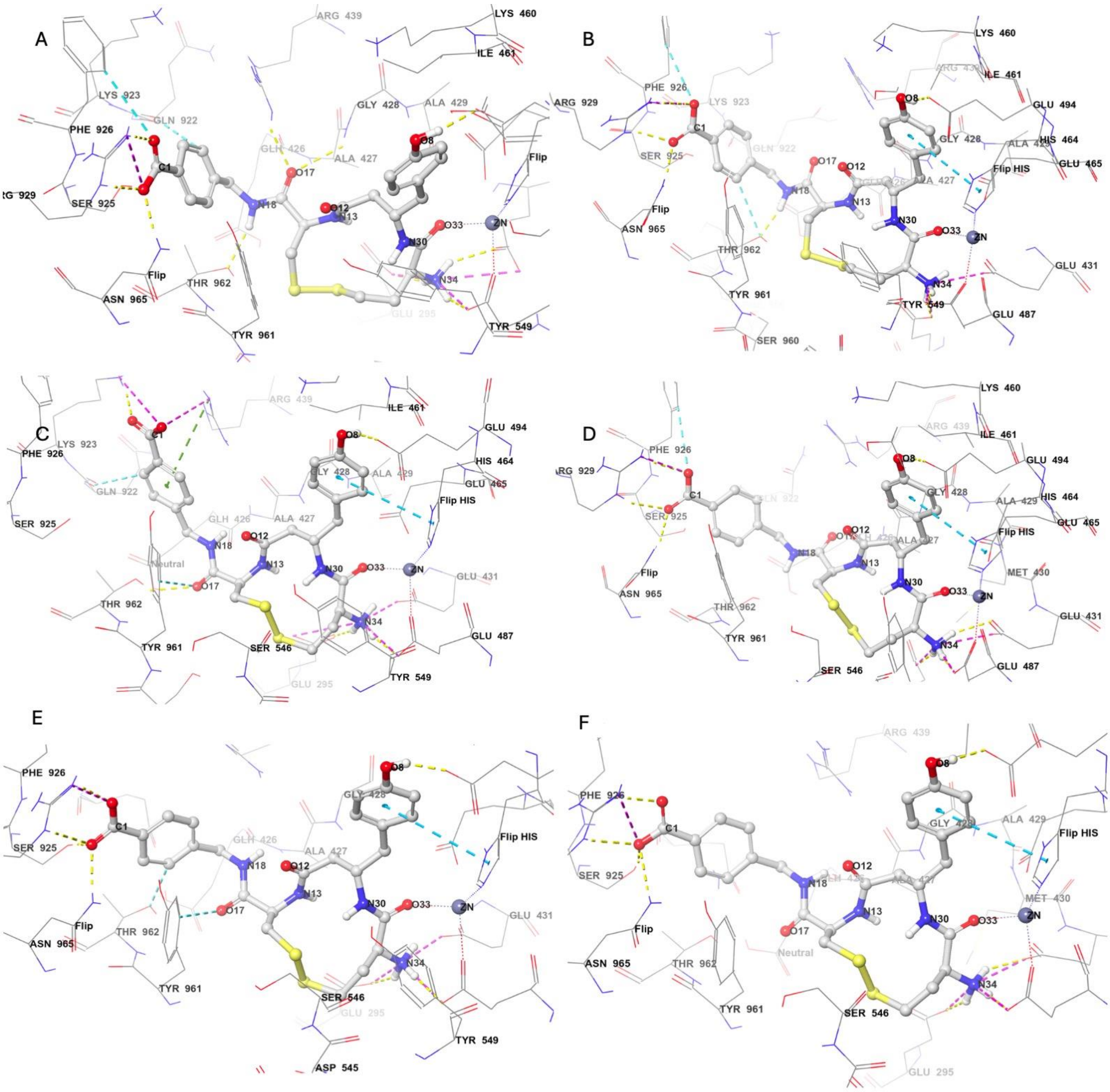

Figure S20. Selected frames at 100 ns intervals from frame 0, for ligand **39** run 2.

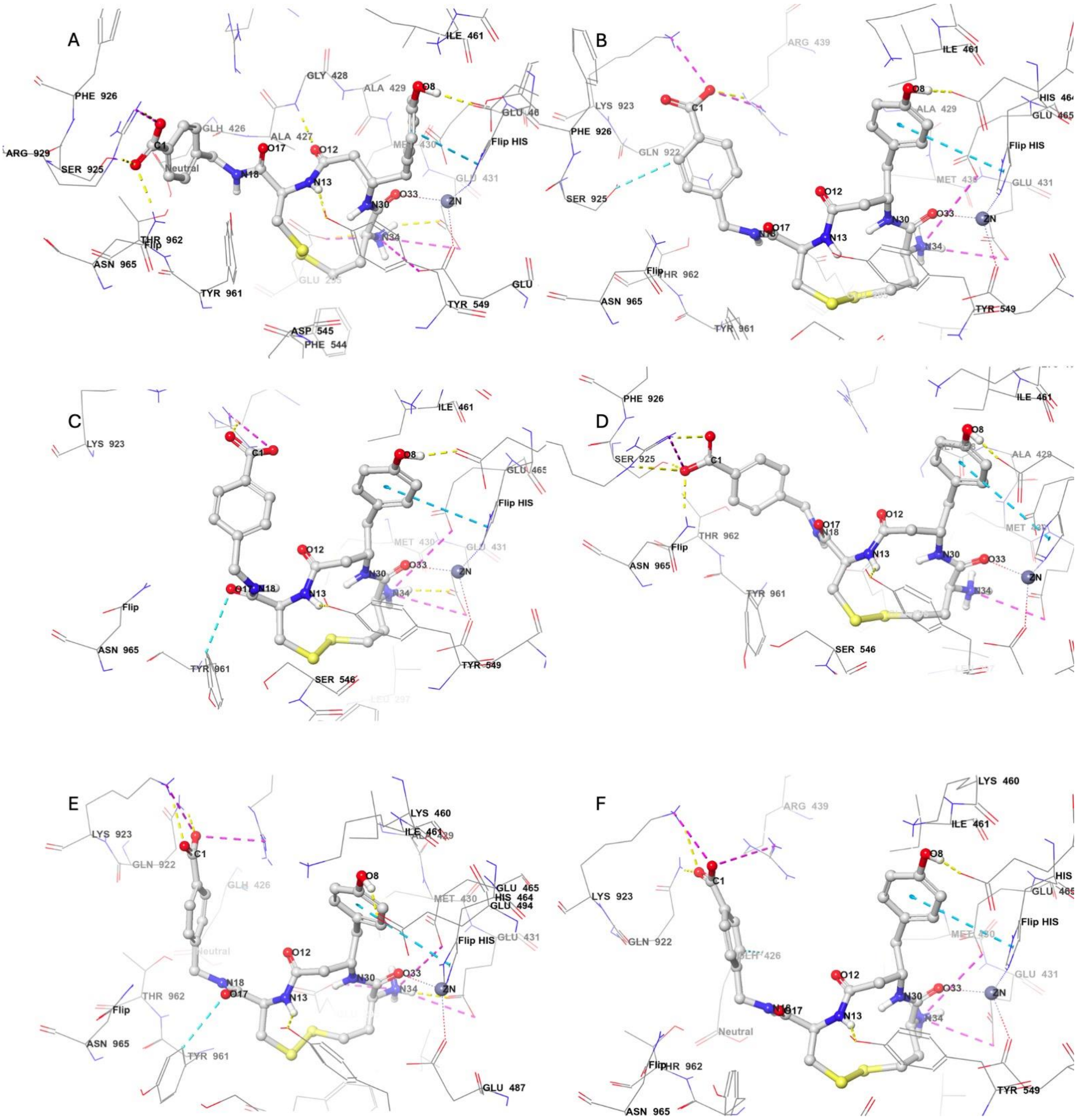

Figure S21. Selected frames at 100 ns intervals from frame 0, for ligand **39** run 3.

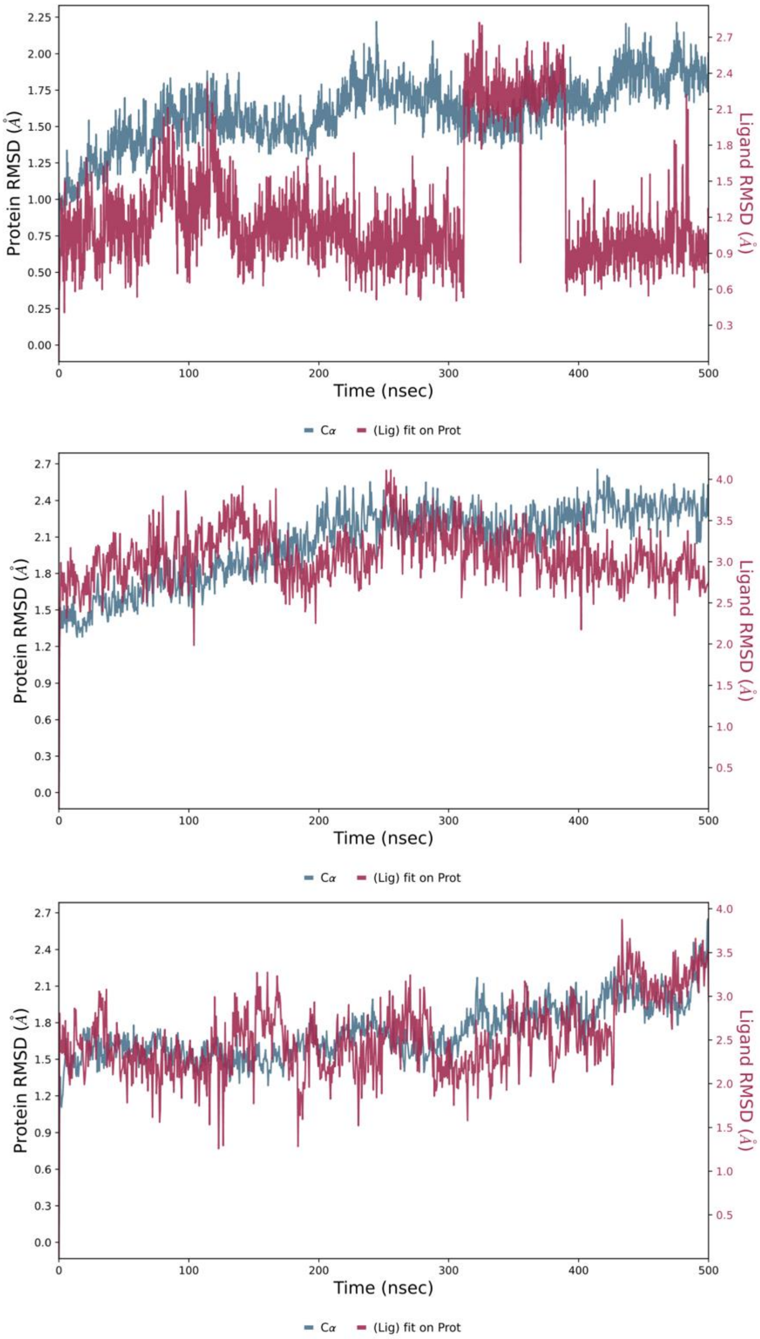

Figure S22. RMSD plots for protein and ligand for MD simulations of **HA08** run 1 (top), run 2 (middle) and run 3 (bottom).

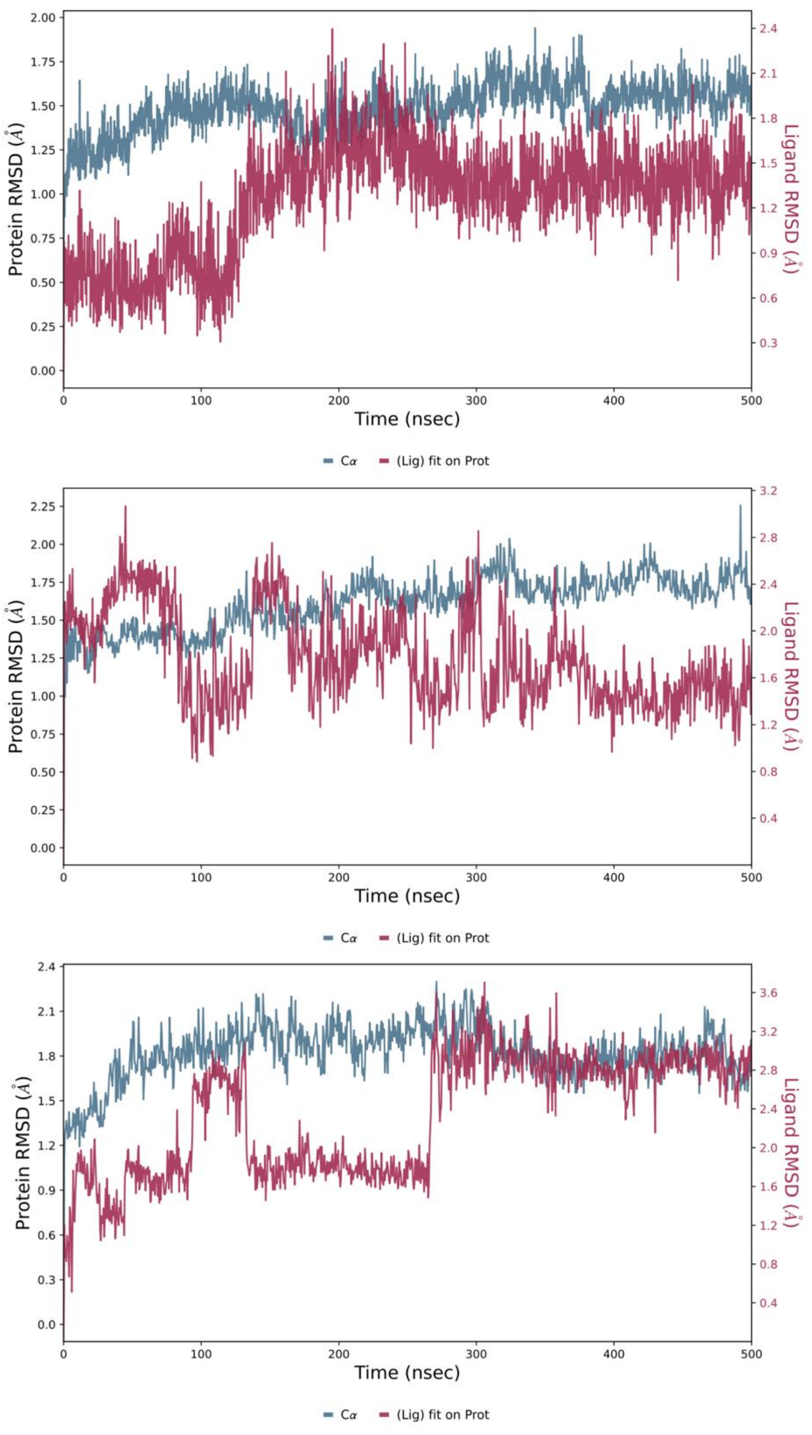

Figure S23. RMSD plots for protein and ligand for MD simulations of ligand **37** run 1 (top), run 2 (middle) and run 3 (bottom).

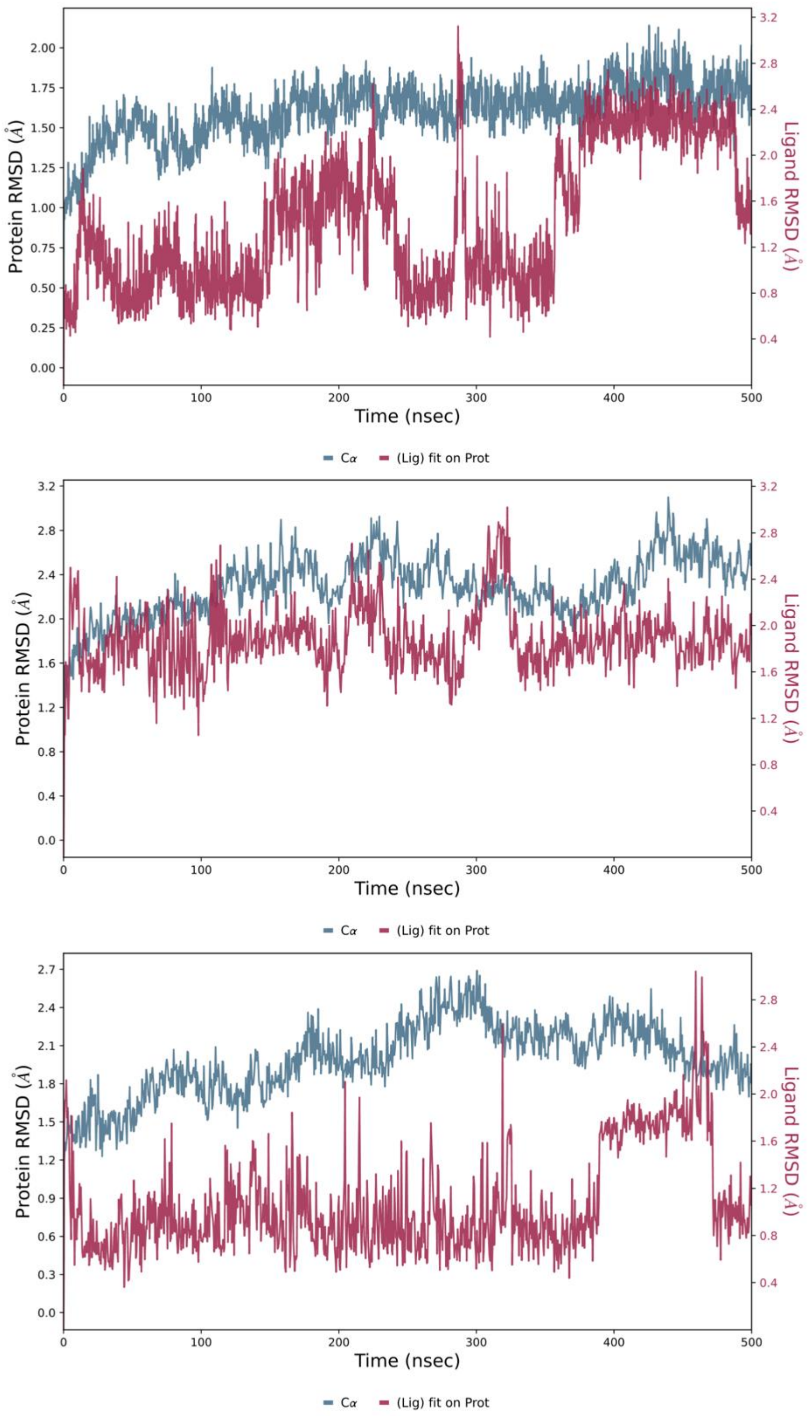

Figure S24. RMSD plots for protein and ligand for MD simulations of ligand **38** run 1 (top), run 2 (middle) and run 3 (bottom).

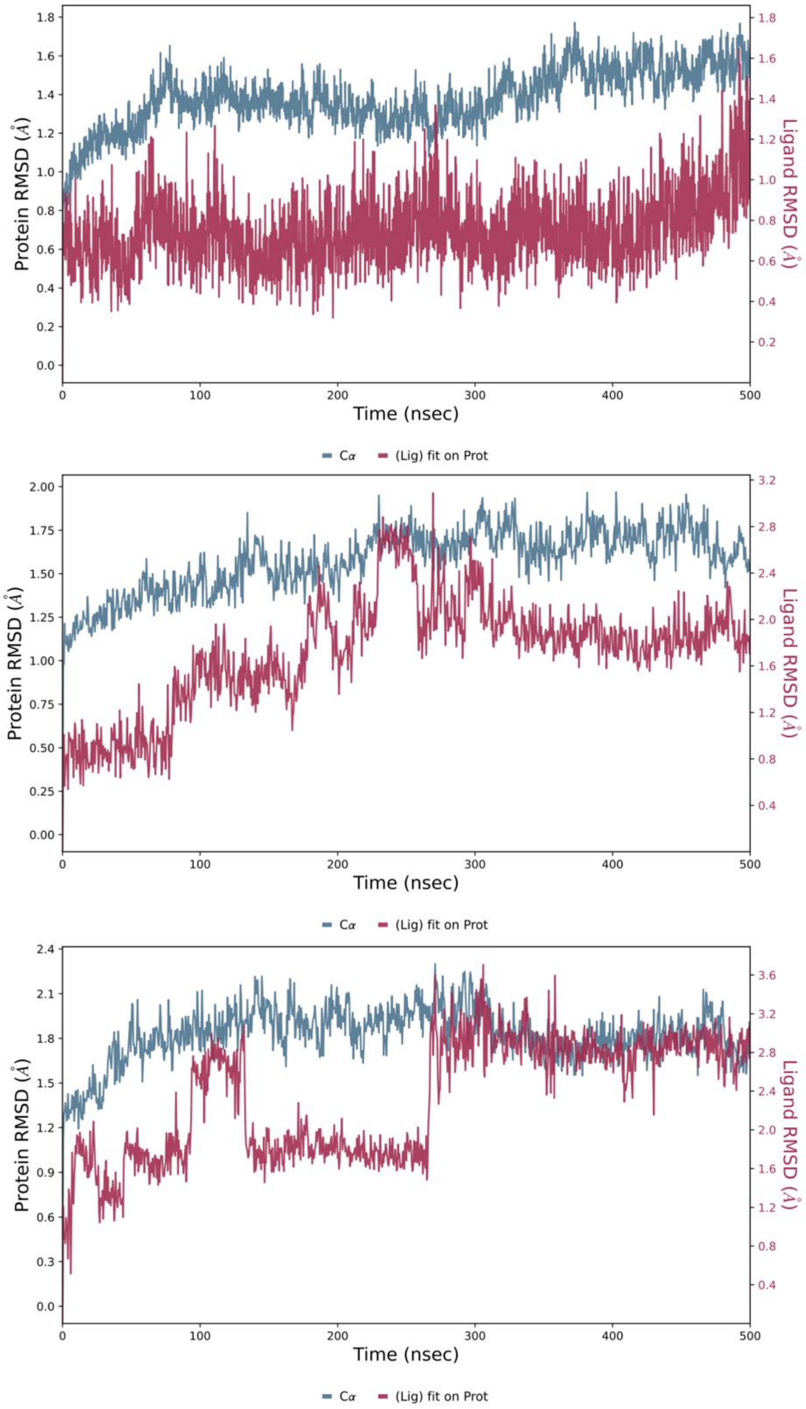

Figure S25. RMSD plots for protein and ligand for MD simulations of ligand **39** run 1 (top), run 2 (middle) and run 3 (bottom).

References

(1) Motiwala, H. F.; Charaschanya, M.; Day, V. W.; Aubé, J. Remodeling and Enhancing Schmidt Reaction Pathways in Hexafluoroisopropanol. *Journal of Organic Chemistry* **2016**, *81* (4), 1593–1609. https://doi.org/10.1021/ACS.JOC.5B02764/SUPPL_FILE/JO5B02764_SI_003.PDF.

(2) Clark, R. D.; Jahangir. Effects of Remote N-(Tert-Butoxycarbonyl) Groups on Heteroatom Directed Lithiation at Benzylic Positions. *Tetrahedron* **1993**, *49* (7), 1351–1356. https://doi.org/10.1016/S0040-4020(01)90188-7.

(3) Pitcher, N. P.; Harjani, J. R.; Zhao, Y.; Jin, J.; Knight, D. R.; Li, L.; Putsathit, P.; Riley, T. V.; Carter, G. P.; Baell, J. B. Development of 1,2,4-Oxadiazole Antimicrobial Agents to Treat Enteric Pathogens within the Gastrointestinal Tract. *ACS Omega* **2022**, *7* (8), 6737–6759. https://doi.org/10.1021/ACSOMEGA.1C06294/SUPPL_FILE/AO1C06294_SI_002.XLSX.

(4) Nhu, D.; Duffy, S.; Avery, V. M.; Hughes, A.; Baell, J. B. Antimalarial 3-Arylamino-6-Benzylamino-1,2,4,5-Tetrazines. *Bioorg Med Chem Lett* **2010**, *20* (15), 4496–4498. https://doi.org/10.1016/J.BMCL.2010.06.036.
